# Safety interventions for the prevention of accidents at work: A systematic review

**DOI:** 10.1002/cl2.1234

**Published:** 2022-06-01

**Authors:** Johnny Dyreborg, Hester Johnstone Lipscomb, Kent Nielsen, Marianne Törner, Kurt Rasmussen, Karen Bo Frydendall, Hans Bay, Ulrik Gensby, Elizabeth Bengtsen, Frank Guldenmund, Pete Kines

**Affiliations:** ^1^ National Research Centre for the Working Environment Copenhagen Denmark; ^2^ Division of Occupational and Environmental Medicine Duke University Medical School Durham North Carolina USA; ^3^ Department of Occupational Medicine—University Research Clinic Danish Ramazzini Centre, Goedstrup Hospital Herning Denmark; ^4^ School of Public Health and Community Medicine Institute of Medicine, University of Gothenburg Gothenburg Sweden; ^5^ Team Working Life Copenhagen Denmark; ^6^ Institute for Work and Health Toronto Ontario Canada; ^7^ Safety Science & Security Group Centre for Safety in Health Care Delft University of Technology Delft The Netherlands

## Abstract

**Background:**

Limited knowledge regarding the relative effectiveness of workplace accident prevention approaches creates barriers to informed decision‐making by policy makers, public health practitioners, workplace, and worker advocates.

**Objectives:**

The objective of this review was to assess the effectiveness of broad categories of safety interventions in preventing accidents at work. The review aims to compare effects of safety interventions to no intervention, usual activities, or alternative intervention, and if possible, to examine which constituent components of safety intervention programs contribute more strongly to preventing accidents at work in a given setting or context.

**Date Sources:**

Studies were identified through electronic bibliographic searches, government policy databanks, and Internet search engines. The last search was carried out on July 9, 2015. Gray literature were identified by searching OSH ROM and Google. No language or date restrictions were applied. Searches done between February and July of 2015 included PubMed (1966), Embase (1980), CINAHL (1981), OSH ROM (NIOSHTIC 1977, HSELINE 1977, CIS‐DOC 1974), PsycINFO (1806), EconLit (1969), Web of Science (1969), and ProQuest (1861); dates represent initial availability of each database. Websites of pertinent institutions (NIOSH, Perosh) were also searched.

**Study Eligibility Criteria, Participants, and Interventions:**

Included studies had to focus on accidents at work, include an evaluation of a safety intervention, and have used injuries at work, or a relevant proxy, as an outcome measure. Experimental, quasi‐experimental, and observational study designs were utilized, including randomized controlled trials (RCTs), controlled before and after (CBA) studies, and observational designs using serial measures (interrupted time series, retrospective cohort designs, and before and after studies using multiple measures). Interventions were classified by approach at the individual or group level, and broad categories based on the prevention approach including modification of:

- Attitudes (through information and persuasive campaign messaging).
- Behaviors (through training, incentives, goal setting, feedback/coaching).
- Physiological condition (by physical training).
- Climate/norms/culture (by coaching, feedback, modification of safety management/leadership).
- Structural conditions (including physical environment, engineering, legislation and enforcement, sectorial‐level norms).

When combined approaches were used, interventions were termed “multifaceted,” and when an approach(es) is applied to more than one organizational level (e.g., individual, group, and/or organization), it is termed “across levels.”

**Study Appraisal and Synthesis Methods:**

Narrative report review captured industry (NACE), work setting, participant characteristics, theoretical basis for approach, intervention fidelity, research design, risk of bias, contextual detail, outcomes measures and results. Additional items were extracted for studies with serial measures including approaches to improve internal validity, assessments of reasonable statistical approaches (Effective Practice of Organization of Care [EPOC] criteria) and overall inference. Random‐effects inverse variance weighted meta‐analytic methods were used to synthesize odds ratios, rate ratios, or standardized mean differences for the outcomes for RCT and CBA studies with low or moderate levels of heterogeneity. For studies with greater heterogeneity and those using serial measures, we relied on narrative analyses to synthesize findings.

**Results:**

In total 100 original studies were included for synthesis analysis, including 16 RCT study designs, 30 CBA study designs, and 54 studies using serial measures (ITS study designs). These studies represented 120 cases of safety interventions. The number of participants included 31,971,908 individuals in 59 safety interventions, 417,693 groups/firms in 35 safety interventions, and 15,505 injuries in 17 safety interventions. Out of the 59 safety interventions, two were evaluating national prevention measures, which alone accounted for 31,667,110 individuals. The remaining nine safety interventions used other types of measures, such as safety exposure, safety observations, gloves or claim rates. Strong evidence supports greater effects being achieved with safety interventions directed toward the group or organization level rather than individual behavior change. Engineering controls are more effective at reducing injuries than other approaches, particularly when engineered changes can be introduced without requiring “decision‐to‐use” by workplaces. Multifaceted approaches combining intervention elements on the organizational level, or across levels, provided moderate to strong effects, in particular when engineering controls were included. Interventions based on firm epidemiologic evidence of causality and a strong conceptual approach were more effective. Effects that are more modest were observed (in short follow‐up) for safety climate interventions, using techniques such as feedback or leadership training to improve safety communication. There was limited evidence for a strong effect at medium‐term with more intense counseling approaches. Evidence supports regulation/legislation as contributing to the prevention of accidents at work, but with lower effect sizes. Enforcement appears to work more consistently, but with smaller effects. In general, the results were consistent with previous systematic reviews of specific types of safety interventions, although the effectiveness of economic incentives to prevent accidents at work was not consistent with our results, and effectiveness of physiological safety intervention was only consistent to some extent.

**Limitations:**

Acute musculoskeletal injuries and injuries from more long‐time workplace exposures were not always clearly distinguished in research reports. In some studies acute and chronic exposures were mixed, resulting in inevitable misclassification. Of note, the classification of these events also remains problematic in clinical medicine. It was not possible to conduct meta‐analyses on all types of interventions (due to variability in approach, context, and participants). The findings presented for most intervention types are from limited sources, and assessment of publication bias was not possible. These issues are not surprising, given the breadth of the field of occupational safety. To incorporate studies using serial measures, which provide the only source of information for some safety interventions such as legislation, we took a systematic, grounded approach to their review. Rather than requiring more stringent, specific criteria for inclusion of ITS studies, we chose to assess how investigators justified their approach to design and analyses, based on the context in which they were working. We sought to identify measures taken to improve external validity of studies, reasonable statistical inference, as well as an overall appropriate inferential process. We found the process useful and enlightening. Given the new approach, we may have failed to extract points others may find relevant. Similarly, to facilitate the broad nature of this review, we used a novel categorization of safety interventions, which is likely to evolve with additional use. The broad scope of this review and the time and resources available did not allow for contacting authors of original papers or seeking translation of non‐English manuscripts, resulting in a few cases where we did not have sufficient information that may have been possible to obtain from the authors.

**Conclusions and Implications of Key Findings:**

Our synthesis of the relative effectiveness of workplace safety interventions is in accordance with the Public Health Hierarchy of Hazard Control. Specifically, more effective interventions eliminate risk at the source of the hazard through engineering solutions or the separation of workers from hazards; effects were greater when these control measures worked independently of worker “decision‐to‐use” at the worksite. Interventions based on firm epidemiological evidence of causality and clear theoretical bases for the intervention approach were more effective in preventing injuries. Less effective behavioral approaches were often directed at the prevention of all workplace injuries through a common pathway, such as introducing safety training, without explicitly addressing specific hazards. We caution that this does not mean that training does not play an essential function in worker safety, but rather that it is not effective in the absence of other efforts. Due to the potential to reach large groups of workers through regulation and enforcement, these interventions with relatively modest effects, could have large population‐based effects.

## 1 PLAIN LANGUAGE SUMMARY

### 1.1 Occupational safety interventions directed at the group or organizational level are more effective in preventing accidents than individual‐level measures

Occupational safety interventions directed at the group or organizational level are more effective at improving safety and behavior and reducing accidents at work than interventions directed solely at the individual level.

Multifaceted measures are particularly effective when they include elimination, substitution or other engineering controls. Safety regulation and enforcement contribute to the prevention of accidents at work, but with lesser effect.

### 1.2 What is this review about?

Accidents at work are responsible for considerable morbidity and mortality, with an estimated 380,000+ fatalities a year worldwide. There are over 3700 fatalities in the European Union annually, while reported nonfatal accidents at work amount to approximately 3.2 million cases annually.

The evidence base regarding what works in preventing accidents at work is limited, which inhibits informed decision‐making by policy makers, occupational health and safety practitioners, business owners, managers, and workers in selecting the most effective approaches to reduce accidents at work.

This systematic review fills this gap by focusing on the main types of occupational safety interventions directed at the individual, group or organizational level. This includes attitude, behavior and physiological modifications, changes in structural conditions, such as legislation and engineering controls (such as barriers, or measures that remove hazardous conditions), and multifaceted approaches combining two or more safety interventions.

### 1.3 What is the aim of this review?

The aim of this Campbell systematic review is to assess the effectiveness of broad classes of safety interventions in preventing accidents at work, and to examine which intervention components are most effective.

### 1.4 What studies are included?

This review includes studies that evaluate the effectiveness of interventions to improve safety and reduce accidents at work. A total of 100 studies, containing 120 safety interventions, were of sufficient methodological quality to be included in the analyses.

The studies use experimental, quasi‐experimental or observational study designs, with about one‐third being of high quality, one‐fourth of moderate quality, and the remaining being low‐quality studies.

### 1.5 What are the main findings of this review?

Strong evidence supports greater effects being achieved with safety interventions directed toward the group or organizational level rather than at the individual level. Engineering controls, including elimination and substitution, are more effective at reducing accidents at work than other approaches, particularly when engineered changes are introduced independently of workplace practices and “decision‐to‐use” by workers.

Multifaceted approaches combining intervention elements at the organizational level, or across levels, provide moderate to strong effects, particularly when engineering controls are included. The evidence supports safety regulation and enforcement, but with lower effect sizes.

Effects are modest for safety climate interventions, for example, leadership safety communication. Intensive group discussions are effective approaches (at medium‐term follow‐up). No effects are found for various physical training methods on reducing accidents at work. Behavioral approaches, such as general coaching and feedback or safety training, are less effective. This does not mean that safety training is not relevant, but rather it is ineffective in the absence of other efforts.

### 1.6 How have these interventions worked?

The relative effectiveness of workplace safety interventions is in accordance with the Public Health Hierarchy of Hazard Control, whereby interventions that are more effective in preventing accidents eliminate risks at the source of the hazard through engineering solutions or the separation of workers from hazards.

### 1.7 What do the findings of this review mean?

Occupational safety intervention efforts should foster safer working environments, machines, tools and working conditions rather than solely focusing on how workers can mitigate the risks. The latter approach should be a last resort, exercised only when other more effective measures are not feasible.

Even though effects are modest for legislation and enforcement, their population‐based effects can potentially be quite large, as they are often applied to broad groups of workers.

For some types of safety interventions, the level of evidence is insufficient or limited: safety campaigns and training, behavioral‐based safety interventions, interventions directed at changes in safety climate and administrative controls, and soft regulation such as audits and certification systems. Here further research should be encouraged.

There is a need for clarification as to how various types of safety intervention are classified. The review authors propose a classification of safety interventions, which they hope can be a starting point in more clearly defining safety interventions in future studies.

### 1.8 How up‐to‐date is this review?

The review authors searched for studies up to July 2015.

## 2 BACKGROUND

### 2.1 Description of the condition

Accidents at work are estimated to kill more than 380,000 workers worldwide every year (Concha‐Barrientos et al., 2005; EU‐OSHA, 2017). In the European Union, the number of fatalities amounts to over 3700 cases annually, and nonfatal accidents at work amount to over 3.2 million cases annually involving at least four calendar days absence from work (Eurostat, 2017). Results from the European Labor Force Survey 2015 indicated that 2.9% of workers in the EU‐28 had an accident at work during a 1‐year period, which corresponds to almost seven million workers (Eurostat, 2015). Aside from the human cost, workplace accidents also represent a significant economic burden to society (EU‐OSHA, 2017; Eurostat, 2004a). Although the risks of accidents at work have been reduced over the last 30 years, the number of accidents remains unacceptably high and continues to receive much attention from a wide spectrum of policy and decision‐makers.

In this review, an “accident at work” is understood as an accident which causes physical harm (injury) to people at work; that injury may be minor, major or fatal. We use the term “accident” for the causal event(s) leading to the harmful exposure of an individual, whereas we reserve the term “injury” for physical harm as the consequence(s) of such an event. In the health and safety field it is important to distinguish “accidents” from chronic injuries related to long‐term exposures, as the etiologies are different. We found the European Commission definition of a work accident the most exhaustive and clear (European Commission, 1999). In this review an accident at work is thus defined as “a discrete, sudden and unexpected occurrence in the course of work which leads to physical harm (injury).” This includes cases of acute poisoning and willful acts of other persons, but excludes deliberate self‐inflicted injuries, and accidents on the way to and from work (commuting accidents). The phrase “in the course of work” is taken to mean whilst engaged in an occupational activity, or during the time spent at work and includes road traffic accidents occurring in the course of work.

However, we exclude accidents where the resulting injury is mental harm alone. Occupational diseases, such as occupational dermatitis and repetitive strain injuries, are not included in this review, as they have longer exposure periods, are not discrete, sudden and unexpected, and thus fall outside the definition of an accident. However, harmful exposures, such as acute poisoning and chemical burns are included, as the exposure period is short, and the event is usually discrete, sudden and unexpected, and thus accidental. It should be emphasized, that in this review the term “accidental” does not mean unpredictable or unpreventable, even though the specific event may be unforeseen by the victim. In fact, we contend, most accidents and their precipitating events are predictable and preventable (Davis & Pless, 2001; Haddon, 1968; Zwetsloot et al., 2017).

The safety science literature has identified several measures influencing risk, safety behavior, and accidents at work (Guldenmund, 2010b; Haddon, 1968; Heinrich, 1931; Kjellén, 2000; Rivara & Thompson, 2000b; Spangenberg, 2010; Tuncel et al., 2006; Zohar, 2010). The development in the research field has improved the predictive power of theories and conceptual models over the last four decades, and these have informed authorities, companies, and employees in developing more reliable and efficient measures for the prevention of accidents at work (Hale, 2006).

Over the last about 30 years, the multidimensional characteristics of risk to workers, and not least the understanding of how to prevent accidents at work, have been widely emphasized in the safety science literature (Guastello, 1993; Kjellén, 2000; Lund & Aarø, 2004; Reason, 1997; Robson et al., 2001; Shannon et al., 2001). This development is referred to as the “third age of safety” (Hale & Hovden, 1998). Whereas accidents previously were seen from a technical, legal, or human factors perspective, in recent years cultural and organizational factors have become important additional perspectives included in accident prevention programs at work (Grote, 2007; Mearns et al., 2003; Parker et al., 2006; Spangenberg, 2010), and so represent a key perspective to understanding the complex and multifaceted approaches to reduce harm to workers (Rasmussen, 1997; Spangenberg et al., 2002). At least seven main categories of approaches can be identified:

*Attitudinal* approaches focus on the modification of attitudes and beliefs and their consequences for behavior and accidents (Lund & Aarø, 2004), which mainly explain behavior in terms of internal mental states and cognitive processes (e.g., knowledge‐attitudes‐behavior).

*Physiological modifications* focus on improving the physiological capacity of individuals through various training methods, such as, endurance training (running, cycling, swimming, etc.); strength and resistance training, such as push‐ups, pull‐ups, weight training, interval training etc.; flexibility exercises, such as stretching to improve joint flexibility; which all aim to reduce the risk of injury.

*Behavior based* approaches is about modifying behavior by use of environmental antecedents and consequences, such as incentives for safe behavior or punishment for unwanted behavior (Luthans & Kreitner, 1985) and have been described widely by practitioners and are supported by organizational and psychological theory (Krause & Russel, 1994; Saari, 1998; Scott Geller, 2011).

*Safety climate* approaches are about modifying the shared perceptions among employees in an organization or group to influence the relative priority of safety enacted within the organization or the group, for example, what kinds of behavior are being rewarded and supported with regard to a specific strategic focus, such as safety at work (Zohar, 2010).

*Safety culture* approaches represent a central approach to safety intervention in theory and practice (Guldenmund, 2000, 2010b; Hale, 2010; Nielsen, 2014). Safety (organizational) culture can be briefly defined as the shared basic assumptions, values, and beliefs concerning safety that characterize a work setting and are taught, often informally, to newcomers as the proper way to think and feel about safety (Zohar & Hofmann, 2012). Safety culture is thus, compared to safety climate, changes in the deeper (tacit) taken for granted assumptions and values that govern organizational life (Schein, 2004; Schneider et al., 2013).

*Structural* approaches comprise varied modifications of the physical, organizational, or regulatory environment, including *engineering control*, for example, introduction of machine safeguards, walkways, elimination of hazardous substances or materials and other changes in the physical environment; *organizational approaches*, such as introduction of occupational health and safety management systems (OHSMSs), including various forms of incentive programs, for example, economic incentives issued by insurance bodies or the use of ranking, benchmarking, or other approaches (soft regulation); and *legislative approaches* involving enforcement of rules and safety standards (Castillo et al., 2006; Haddon, 1968; Heinrich, 1931; Herrick & Dement, 1994; Lingard & Holmes, 2001; Robson et al., 2007).

*Multifaceted* approaches usually integrates several components in the prevention of accidents at work and are characterized as a complex intervention. Research has emphasized the importance of integrating these various components to achieve a higher level of safety at work (DeJoy, 2005; Guastello, 1993).

### 2.2 Description of the intervention

In this review we focus on primary safety interventions, defined as any attempt deliberately applied to promote safety and decrease the frequency or severity of accidents at work (Robson et al., 2001). Such accidents may subsequently have consequences in terms of work absence, disability, and other personal or economic costs.

As safety interventions are often not based on one component alone, it is expected that some of the studies included in this review will consist of evaluations of more than one type of intervention component (multifaceted), such as safety training, a safety campaign, goal setting, safety feedback, or machine or tool safeguarding. It is recognized in the field that there can be a lag phase (latency period) in the implementation of safety interventions, and that this is dependent on the type of safety intervention and the context (Robson et al., 2007). For example, the introduction of a new provision or safety legislation will be expected to have a longer lag phase compared to the introduction of a new safeguard on a machine, which could have immediate effects. This will be taken into consideration when effects of interventions are evaluated. A safety intervention may consist of one or more components, and can run for a shorter or longer period of time, or involve a permanent change, such as new regulations or orders.

A safety intervention can be initiated at work, for example, by the employer or the employees, or initiated externally by public authorities, social partners or other stakeholders. Interventions aimed at the prevention of accidents at work can operate at different levels, namely at the micro‐, meso‐, or macro‐level, that is, individual, group or organizational, or the broader societal‐industry level, respectively (Dyreborg, 2011; Haslam et al., 2005; Hofmann et al., 1995; Landeweerd et al., 1990; Lund & Aarø, 2004; Spangenberg, 2010).

In this review an inclusive approach to the meaning of a safety intervention was adopted that recognizes various theoretical approaches and distinctions made in the scientific literature and in practice in this field. It is not expected that all studies evaluating safety interventions in general are explicit about how the intervention may work, but based on the information we will seek to classify theoretical underpinnings and the types of interventions. Lund and Aarø (2004) have suggested that safety interventions for the prevention of accidents at work may work by applying three main types of measures: attitude modification, behavior modifications and structural modifications. Additionally, a fourth type, safety climate modifications, is suggested by a large body of research (Christian et al., 2009; Nahrgang et al., 2007; Spangenberg, 2010; Zohar, 2002; Zohar & Luria, 2003) and a fifth type, namely safety culture (Guldenmund, 2000, 2010b; Zohar & Hofmann, 2012). Finally, we identified studies on physiological approaches representing a new type of safety intervention. In practice, these types of measures can involve many different components to form multifaceted safety interventions.

In this review we define a safety intervention component as an independently operating entity in the intervention, which can stand‐alone. This means, for example, that the *instruction* in use of a new specific item of fall protection equipment is not a component in itself, but is part of the component “introduction of fall protection equipment,” since the introduction to the use of the new equipment would be meaningless without this instruction. However, in the case where a general introduction or campaign about fall risks at a workplace is given to raise awareness among workers, besides the introduction of fall protection equipment, it will be classified as two separate components, as both components can be considered stand‐alone interventions.

Below is listed the main types of components of safety interventions directed at the individual or the group/organizational level, respectively.

#### 2.2.1 Main types of components directed at the individual level

*Attitude modification*: This may be achieved by means of information and persuasive messages in campaigns, leaflets, booklets, films, posters, direct mail, guidelines, or by teaching or various counseling approaches. Educational approaches that attempt to change attitudes—typical in classroom based safety introduction, are included here and should be distinguished from training of skills.

*Behavior modification:* This may be achieved by means of training, incentives, goal setting, feedback, or individual coaching etc. This category includes skill‐based training, for example, proficiency or dexterity of doing a manual work task, and other types of skill based accomplishments (not just attempts to change attitudes)

#### 2.2.2 Main types of components directed at the group or organizational level

*Safety climate/culture modifications:* Climate, culture, or social norms may be changed through leadership‐based interventions, goal setting, feedback, or other approaches to modify values or norms related to safety at work. Safety climate reflects the shared priority of safety in an organization/group compared to other competing goals, such as productivity or quality. Safety climate/culture work on how people at work are expected to act under different circumstances. This mechanism thus differs from merely introduction of safety standards and safety management systems, as the core of the mechanism in safety climate is the priority of safety compared to other competing goals, and that it is a group level (shared among group members) phenomenon (Zohar, 2010).

*Structural modification:* Contextual factors are changed through legislation, regulation, enforcement and economical or other incentive systems, including benchmarking, ranking, or other measures that work through the reputation and legitimacy of the company (soft regulation). Structural modifications also include changes in the internal organization of safety management systems in the enterprise, such as workers' rights and obligations (employee involvement), involvement of workers in decisions, safety management systems, and certification regimes; the physical environment and engineering controls, such as modification of machines, equipment, and products.

*Multifaceted safety interventions*: Combination of components across the main types of safety interventions.

The review *excludes* secondary and tertiary interventions, such as on‐site injury treatment, rehabilitation and return to work programs. Public safety campaigns directed at the general population and community‐based safety interventions are also excluded from the review, as they are not primarily implemented at workplaces.

### 2.3 How the intervention may work

Even though the working environment, the nature of work, and the workforce vary from one industry to another, we assume that the different types of safety interventions work in similar ways across various settings, although the effect may be modified by contextual factors, such as whether the industry is static or dynamic (Cooper, 2007; Sadayappan & Moaued, 2011). Work settings that experience transient workforces, such as in construction work, can present barriers or challenges to the implementation of safety interventions, as steps taken are easily lost when staff disappear and new ones come in. In addition, peer pressures resulting from the social dynamics within a group could be weaker in dynamic settings with unstable workgroups, thus making safety interventions more difficult to maintain, such as improvement in safety climate in construction work (Dedobbeleer & Béland, 1991; Kines et al., 2010).

Below we describe how the main types of safety interventions may work with references to the most relevant theory in the field.

*Attitude and belief modification*: The underlying assumption of this type of approach is that attitudes and beliefs can be modified by the provision of information and knowledge to the relevant persons, and that this in turn can influence behavior. The theoretical or conceptual support of such approaches is the KAP (Knowledge‐Attitudes‐Practices) model (Lund & Aarø, 2004). According to this model, safety‐related behavior is determined by an individual's beliefs and attitudes. Within this perspective, safety information, for example, provided by pamphlets, safety campaigns, or safety courses, would change behaviors, through providing workers with “the right” information or knowledge about the hazards at the workplace, and the consequences these may have on their safety and health, which in turn will alter their attitudes and beliefs.

Social psychology research has provided theoretical knowledge of the relationship between attitudes and beliefs and human behavior (Hofmann & Tetrick, 2003). This attitude–behavior relationship has provided the basis for a number of practical approaches for the prevention of accidents at work, where knowledge and information related to risk and safety at work have been disseminated or taught to workers with the intention to modify their attitudes and beliefs and in turn promote safer behavior (Burke et al., 2006). However, this approach is not without critics.

Wicker (1969) reviewed research on the relationship between attitudes and behavior, and concluded that attitudes or beliefs probably not predict behavior. Since this review, social psychology researchers have sought to develop models that increase the predictive power of attitudes. The main approach in this study area has been to build more complex models of the relationship between attitudes and behavior, by inclusion of many additional components assumed to affect behavior (Armitage & Conner, 2001). The two most known models are the Theories of Reasoned Action (TRA) (Ajzen & Fishbein, 1980; Fishbein & Ajzen, 1975) and the Theory of Planned Behavior (TPB) (Ajzen, 2012). The assumption is that behavioral intention is the best predictor of behavior. Behavioral intention is regarded as a result, not only of attitudes, but also of social influences (including social norms) and self‐efficacy (or perceived behavioral control). With this development in social psychology theories, an increasing number of factors related to the environment is included to improve the predictability of the theories, and thus comes closer to the environmental approach, as seen in behavior modification theories.

*Physiological modifications:* Aim to modify the physiological capacity of individuals through various training methods, for example: endurance training (running, cycling, swimming, etc.); strength and resistance training, such as push‐ups, pull‐ups, weight training, interval training etc.; and flexibility exercises, such as stretching to improve joint flexibility. The underlying assumption of these training methods is that a stronger body can better resist loads on the body thus avoiding a potential accidental injury. These training approaches can also be combined into an integrated training program.

*Behavior modifications* are based on an environmental approach, for example, incentives for safe behavior. Attitude modification approaches mainly explain behavior in terms of internal mental states and cognitive processes (e.g., knowledge‐attitudes‐behavior), whereas behavior modification approaches represent an external focus that explains behavior in terms of environmental consequences, such as incentives or punishment (Luthans & Kreitner, 1985).

The theoretical framework for the behavior modification programs is based on *behaviorism*, specifically operant (learned) conditioning that can be traced back to B. F. Skinner (1969), who contributed with the distinction between learned and unlearned behavior. B. F. Skinner suggested that humans choose various responses to receive a particular consequence. This contingency is framed as the Antecedent‐Behavior‐Consequence (A‐B‐C) model, where both antecedents and consequences are responsible for affecting the behavior of an individual. Where antecedents serve to define or signal the desired behavior, the consequences of behavior serve in influencing and reinforcing the behavior more directly and encourage the occurrence of a desired behavior (Krause et al., 1999). Skinner's theoretical framework has been expanded by inclusion of the mediating role of cognition, and the term organizational behavior modification has been suggested for this approach (Luthans & Kreitner, 1985). Another important expansion of the Skinnerian approach is the social learning theory (Bandura, 1971). These theoretical frameworks have informed safety intervention research and practice. Some commonly used components in behavior based safety (BBS) interventions are safety training, goal setting and feedback, observation and feedback, verbal feedback, data analysis, and problem solving (Krause, 1999; McAfee & Winn, 1989). A multitude of BBS studies have been conducted since the 1970s (Tuncel et al., 2006), which include a number of various models and components (Cooper, 2007; Laitinen & Ruohomäki, 1996). One drawback is that the possible effect of behavior based approaches seems to disappear when the incentives are no longer present, and that the sustainability of the effect rests in the hands of the adopting organizations and their leaders (Cox & Jones, 2006).

*Structural modifications* include varied approaches that change the physical, organizational or regulatory environment. A common feature of the structural approaches is that environmental factors are changed, often over longer time periods or permanently, consequently with more profound effect. One type of structural modification is *engineering control*, for example, introduction of machine safeguards, walkways, elimination of hazardous substances or materials and other changes in the physical environment that directly influences individuals' safety without necessarily affecting their behavior.

Preference for *engineering control* is based on the public health hierarchy of hazard control (Herrick & Dement, 1994; Lingard & Holmes, 2001). This approach follows the basic tenet of industrial hygiene, which is control of health hazards in working environments; it has been applied to the control of physical hazards responsible for energy transfer and subsequent accidents and injuries in the workplace (Castillo et al., 2006). Emphasis in this model is given to the most effective and efficient preventive measures that eliminate risk at the source of the hazard. Lower tiered approaches in the hierarchy control risk through barriers or use of personal protective equipment or training efforts. Of note, engineering controls typically focus on control of a specific hazard in marked contrast to safety climate or culture approaches that often do not, but rather address safety more broadly.

Other types of approaches in this group are based on simple linear models, where a chain of multiple events culminate in an injury. Safety prevention is then directed at removing one or more of the elements involved in this chain of events, to prevent the occurrence of the injury. One such model is Heinrich's “Domino theory” (Heinrich, 1931), which has had a tremendous effect on practical safety interventions, and still is much in use despite numerous pitfalls (Johnson, 2011; Manuele, 2014). A further development of these models is the complex linear models, such as the Swiss cheese model (Reason, 1997), that illustrate that even though there are several barriers between hazards and accidents there can be flaws (holes in the slices of cheese) in these barriers that co‐incidentally can be aligned and result in accidents.

Another type of structural modification is *social control*, which introduces coercive power or incentives for people or organizations to change behavior. This is related to compliance with rules and regulation on a nonvoluntary basis, for example, by use of enforcement and legal sanctions, as well as compliance on a voluntary basis, for example, by use of marketing, economic incentives, reputations, and benchmarking, which involves a voluntary exchange, for example, insurance‐related benefits for low risk companies. Regulation may serve as a potentially powerful institutional force to promote the adoption of occupational health and safety policies and practices (Chambers et al., 2013). The basic idea is that such instruments provide an incentive for companies or people at work to stick to certain (legal) standards, either due to the risk of penalties in case of noncompliance, or because a benefit can be achieved in exchange for an appropriate behavior (Rothschild, 2000).

Currently, reports of the effect of legislation as a structural approach to occupational safety are conflicting, which is not surprising given the complexities involved in such evaluations for the most part. While effect variation may reflect differences in the actual implementation of the legislative approaches to prevention, a number of other factors may also be of potential importance. These include the very nature—or strength—of the legislation and thus the requirements it is intended to impose. For example, a call for training would be expected to have a different effect than a requirement for safer equipment that removed a dangerous exposure.

The methods and approaches to the evaluation of these effects also play important roles in our understanding. Legislative efforts typically influence the entire population of interest at one point in time, meaning that the use of an experimental design, such as an randomized controlled trial (RCT), to assess effectiveness, is typically not possible. This makes the identification and selection of appropriate comparisons for quasi‐experimental designs particularly important. Interest is largely in assessment of longer‐term effectiveness, which increases the risk of having results confounded by maturation effects. Failure to appropriately identify, or explore, the necessary latency of effect of any intervention will make it more difficult to discern effects that do exist, and effects may manifest early after promulgation, but wane later.

Regulatory activity can be precipitated by untoward events, and may include activities that business has already found palatable and adopted in some part by the time the legislation is passed, which can make the discernment of effect more difficult. Regulatory efforts are preceded by a rule‐making process which is typically one of negotiation, such that the final product of legislation may not be evidence‐based. Furthermore, the passing of legislation does not necessarily equate with full enforcement activities, making it difficult to identify differences in failures of theory versus failure of implementation.

*Safety climate and culture modifications*: Safety culture has long been a subject of interest for safety science, and particularly so following the Chernobyl nuclear meltdown in 1986. However, the theoretical framework for safety culture is generally underdeveloped, and the link to research on organizational culture has been weak or even non‐existent (Choudhry et al., 2007; Guldenmund, 2000). There is, for instance, no widely accepted definition of an organization's safety culture or any consensus on how to change a safety culture to improve safety. Therefore, the concept of safety culture is vague and not easily translated into safety prevention efforts, which may explain why there is a noticeable lack of (safety) culture change intervention studies in the safety literature (DeJoy, 2005; Hale et al., 2010; Nielsen, 2014). The most elaborated theory of safety culture is based on Edgar Schein's Theory of organizational culture (Schein, 2004), where the essence of culture is its core of basic assumptions that manifest as values, and in turn defines behavioral norms, for example, norms that influence safety behavior. The basic assumptions and values are taken for granted and maintained by members of a group, and will be taught to new members as the correct way of thinking and feeling in relation to specific aspects, such as safety. Following this, safety culture may be defined as those aspects of the organizational culture which will impact on attitudes and behavior related to increasing or decreasing safety or risk (Guldenmund, 2010a).

A related concept of culture is climate, which describes the shared perceptions of organizational policies, practices and procedures, both formal and informal (Reichers & Schneider, 1990). The level of analysis in safety climate studies is the organization or the group, and thus differs from individual‐level attitudinal research, even in cases where the latter uses climate survey items. Safety climate approaches include, for example, leadership based safety interventions, as leadership is seen as a safety climate antecedent, which has consequences for safety behavior and safety at work (Kines et al., 2010; Zohar & Luria, 2003).

Since the seminal safety climate article by Zohar (1980), a multitude of safety climate studies have emerged (Dedobbeleer & Béland, 1991; Flin et al., 2000; Glendon & Litherland, 2001; Zohar, 2002, 2010; Zohar & Luria, 2003). This type of intervention seems to be supported by a consistent theoretical framework, relating to organizational sensemaking processes (Weick, 1993, 1995; Zohar & Luria, 2004), social interactions (Morgeson & Hofmann, 1999), and social exchange and climate theory (Christian et al., 2009; Mearns et al., 2010; Nahrgang et al., 2007; Zohar, 2003; Zohar & Luria, 2004).

Although there is no general consensus on a definition of safety climate, and the literature has been plagued by conceptual ambiguity (Zohar, 2010), meta‐analyses have identified some common ground and reveal that safety climate is a robust predictor of safety performance (Nahrgang et al., 2007). The assumption underlying this approach is that the safety climate of a group or an organization, which can be understood as a socially constructed phenomenon, as it emerges as a group‐level property through shared cognitions and social consensus, informs workers on how they are expected to act under different circumstances. Thus, safety climate reflects the shared priority of safety in an organization/group compared to other competing goals such as productivity or quality. It has been suggested that safety climate can be understood as a surface manifestation (espoused values) of the deeper cultural levels (Guldenmund, 2000, 2010b; Schein, 2004). Furthermore, the culture and climate approaches have brought focus on the role of leaders and leadership in creating general organizational change, and in the prevention of accidents at work. These approaches are often centered on the commitment to and priority of safety demonstrated by supervisors and top management (Beus et al., 2010; Hofmann & Tetrick, 2003; Zohar, 2002).

### 2.4 Why it is important to do this review

Accidents at work are prevalent in society, but there are important gaps in our knowledge of the effectiveness and efficiency of various safety prevention efforts. This lack of a clear base of evidence creates challenges for policy makers, business owners, worker advocates, and public health practitioners as they seek approaches to prevent accidents at work.

Despite earlier attempts to summarize the evidence, the effectiveness of varied approaches for preventing accidents at work remains unclear. Previous reviews have typically restricted their focus to one type of injury, for example, eye injuries, or one type of prevention measure, such as BBS, OHSMS etc. (Cameron & Duff, 2007; Robson et al., 2005, 2007; Tuncel et al., 2006), one type of event, for example, falls (Hsiao & Simeonov, 2001; Rivara & Thompson, 2000a), or on one industry, for example, agriculture or construction (Lehtola et al., 2008; Lisa & Risto, 2000; Rautiainen et al., 2008). Specificity of focus can decrease the likelihood of misclassification and may therefore be useful when addressing very narrow review questions. Furthermore, a very specific focus may answer a very specific question, but also produces results that may not be useful in broadly addressing the most effective types of safety interventions, that is, identifying a “best” approach to safety intervention in a given context. For these reasons this review considered all work settings and the contextual factors reported, and considered the relevant follow‐up times, as the latency period for when to expect an effect differs depending on the type of safety intervention.

#### 2.4.1 A brief summary of previous studies and reviews

A recent update of a Cochrane systematic review (van der Molen et al., 2007) assessed the effectiveness of preventing injuries in the construction industry (van der Molen et al., 2013). Of the 17 studies included in the review, 12 were interrupted time series (ITS) studies, and 1 was a controlled before‐and‐after (CBA) study. The authors reported limited evidence for the effectiveness of a multifaceted safety campaign (Spangenberg et al., 2002), and a multifaceted drug program (Wickizer et al., 2004) in preventing injuries, and no evidence was found that legislation is effective in preventing nonfatal or fatal injuries in the construction industry (Lipscomb et al., 2003). The methodology of this review has been criticized for a lack of sensitivity to context and a lack of flexibility in using the Effective Practice of Organization of Care (EPOC) review criteria, making it difficult to address realistic challenges faced in evaluating workplace interventions (Lipscomb et al., 2009). To avoid such criticism in the present review, we have taken some important methodological steps:

- first, we do not restrict the review to any one industry;
- second, we implement a grounded approach to studies using serial measures (including ITS designs) that includes a narrative review and synthesis of contextual detail provided by investigators;
- third, we consider the relevant follow‐up times, as the latency period for when to expect an effect will differ depending on the type of safety intervention;
- fourth, we consider the contextual factors reported;
- finally, we distinguish between various types of safety interventions by classifying them theoretically and conceptually.

Previous research related to the seven types of safety interventions is reported below.

*Attitudinal approaches:* Modification of attitudes and beliefs and its consequences for behavior and accidents has been researched in various settings outside and inside the workplace. Attitudes seem to be related to behavior and accidents, even though the evidence is not clear (Guastello, 1993; Lund & Aarø, 2004; Rundmo, 2000; Williamson et al., 1997).

*Physiological modifications:* The underlying assumption of these training methods is that a stronger body can better resist loads on the body thus avoiding a potential accidental injury. These training approaches can also be combined into integrated training programs. The idea of physical training or exercise as a way to prevent accidents represent a relatively new approach in an OHS context. A limited number of studies is known and provides inconclusive evidence (Costa et al., 2008; Stojanovic & Ostojic, 2012). This approach could also include weight loss programs and the like.

*Behavioral approaches:* Behavior based interventions have in previous studies shown a consistent positive effect on the reduction of the reporting of accidents at work (Cooper, 2007; Krause et al., 1999; Stajkovic & Luthans, 2001; Tuncel et al., 2006). A systematic review and meta‐analysis was carried out on behavioral safety interventions in 2006 (Tuncel et al., 2006). The study shows evidence for the effect of behavioral safety interventions. However, the review excluded training interventions, such as those addressing the antecedents of behavior.

*Safety Climate approaches:* Three recent meta‐analytic studies revealed that safety climate offers robust prediction of safety related outcomes across industries and countries (Beus et al., 2010; Christian et al., 2009; Nahrgang et al., 2007), and thus demonstrate the strength of an inverse relationship between safety climate and safety outcome, such as work accidents (Zohar, 2002, 2010). A fourth recent meta‐analytic study showed that a supportive workplace environment was consistent in explaining safety outcomes and other variables across industries (Nahrgang et al., 2011).

*Safety culture approaches:* Reviews of safety culture interventions have mainly been conducted as qualitative assessments and primarily based on a theoretical evaluation of the effectiveness (Farrington‐Darby et al., 2005; Gadd & Collins, 2002; Guldenmund, 2000; McDonald et al., 2000; Vaughan, 1996), and in some cases include organizational aspects (Guldenmund, 2010a; Hale & Hovden, 1998; Hale et al., 2010; Parker et al., 2006; Weick, 1987). As safety culture represents a central approach to safety intervention theory and practice, it is important to evaluate the existing knowledge, and assess the effect of safety culture on behavior and accidents at work, if the quality of the available studies allows for such an evaluation.

*Structural approaches:* A review covering literature up until July 2004 (Robson et al., 2005, 2007) concluded that the body of evidence was insufficient to make recommendations either in favor or against OHSMSs. A recent review covering literature up until January 2013 concluded that there is evidence that labor inspections decrease occupational diseases and/or accidents in the long term, but not in the short term (Mischke et al., 2013). The review thus included both the effect on either health and safety hazards or rates of occupational diseases and injuries. The quality of the evidence was however considered low. The study excluded studies on the effects of voluntary consultations and legislation, and was thus restricted to studying the effects of enforcement. The present review will only consider effects on accidents, but also includes the effects of legislation and voluntary consultations, such as soft regulations, occupational health service systems, and safety audits. We acknowledge that in some cases it may be difficult to distinguish between the effect of legislation and the effect of the enforcement of the legislation.

*Multifaceted approaches:* A review of accident prevention programs by Lund and Aarø (2004) concluded that the greatest effect is obtained in a combination of attitudinal, behavioral, and structural approaches (multifaceted interventions). Even though the review is quite comprehensive, and provides a very useful categorization and modeling of the level and type of intervention, it did not establish summarized effect sizes for the various prevention measures. Moreover, the study also included nonoccupational accidents such as those occurring during leisure time, in traffic and at homes. The review suggested that it may not be possible to influence an organization's safety culture directly, which has also been supported by other studies (Richter & Koch, 2004; Grote, 2007). A review by Guastello (1993) compared reductions in accidents for 10 types of workplace safety interventions, and showed that individual approaches had smaller effect sizes compared to more comprehensive (multifaceted) programs. However, the review did not assess the statistical significance for effect sizes; thorough and systematic assessment of the methodological quality of the included studies was lacking; and finally, an appropriate conceptual categorization of the workplace safety programs was missing.

Even though several reviews and evaluations of safety interventions have been conducted, reviews of workplace safety interventions using systematic approaches are limited in number, not up to date, and not comprehensive, particularly in systematic reviews that include programs covering different levels and types of components.

**Figure 1:**
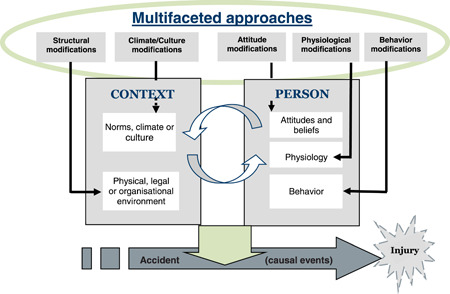
Possible pathways for promoting safety and decreasing the frequency or severity of accidents causing injury to people at work (logic model)

This systematic review summarizes the most recent scientific evidence on the effectiveness of the main types of safety interventions and their components in preventing accidents at work; the process was based on the conceptual model of Lund and Aarø (2004). A main type of safety intervention could be, for example, attitude modification at the individual level or structural modification at the organizational level (Figure 1). The review aims to fill the gap in the extant knowledge of safety interventions at work by evaluating the effects of the main types of interventions for preventing injuries at work and to synthesize best practices that are widely applicable.

## 3 OBJECTIVE OF THE REVIEW

The objective of this review was to assess the effectiveness of broad categories of safety interventions in preventing accidents at work (SIPAW). The review aims:

- to compare the effects of safety interventions to no intervention, usual activities or alternative intervention, and if possible,
- to examine which constituent components of safety intervention programs contribute more strongly to preventing accidents at work in a given setting or context.

## 4 METHODS

### 4.1 Criteria for considering studies for this review

A variety of research designs have been used to evaluate workplace safety interventions. Random allocation is not always feasible for all types of safety interventions in workplace settings due to of several practical issues. Moreover, workplaces are often highly dynamic and complex social entities and accidents are rare events, thus not lending workplace safety interventions to be evaluated using highly controlled studies. For the same reasons, observational studies were included in the review as they allow for the assessment of longer‐term effects and opportunities to incorporate larger samples at times. They provide alternative means of evaluating interventions when experimental designs are not appropriate or feasible. Thus, for the purposes of this review we included a broader range of studies than is typically found in Campbell reviews. In the review we had interest in differentiating effects of interventions focused on individuals with those focused more broadly on groups such as in organizational approaches. However, even though safety interventions may focus on individuals or at group levels, the effects are typically measured at the workplace level. The protocol for this review includes further details on these issues (Dyreborg et al., 2015).

#### 4.1.1 Types of studies

In this review the following types of studies were eligible for inclusion:

RCTs, including studies with cluster randomization.

Quasi randomized study designs (where participants are allocated by means such as alternate allocation, person's birth date, the date of the week or month, case number or alphabetical name order).

Controlled before and after study designs (quasi‐experimental designs) such as controlled two group study designs, and study designs using observational data where statistical methods such as modeling of differences in differences are used.

Studies utilizing serial measures including, but not limited to, ITS, which use observations at multiple time points before and after an intervention (the “interruption”) and retrospective cohort designs. In safety science studies, the ITS design which uses multiple time points before and after an intervention (the “interruption”), can be useful for the evaluation of the effect of legislative changes, changes in safety procedures, changes in the use of new types of machinery, etc., when long‐term effects are of interest, and when randomization is not feasible. For our purposes, we included studies with at least three measures, if at least one measure was before the intervention.

Single group study designs with before and after measures (BA designs). The simple BA study is a type of nonexperimental design that is commonly used in safety science studies. Although it suffers from serious threats to internal validity, it can provide preliminary evidence for intervention effectiveness, when it is supplemented with complementary information (Robson et al., 2001). We also recognize that BA designs are often the first designs used to assess effectiveness of new interventions. As such, they were considered to potentially provide important preliminary information, as well as providing a more complete picture of the components included in safety interventions for the prevention of accidents at work. Simple BA designs were not included in any meta‐analysis, but were simply reviewed as a source of additional information about interventions.

The comparison conditions were either no intervention, such as attention/placebo controls or wait list controls (*absolute effects*), or usual or alternative control conditions (*relative effects*). Analyses of the absolute effects of safety interventions were conducted separately from safety interventions evaluating the relative effects.

Studies with serial measures (including ITS studies) often compared the same work group over time, looking for changes reflecting the effects of the intervention. Experiences of external comparison groups were utilized at times to strengthen the design, as were internal comparisons within the study population using a different injury event than the one targeted by the intervention. Another approach is the use of stratified analyses within the group exploring differences in groups who were more likely to have been affected by an intervention to those who were less likely to be affected. For our purposes studies utilizing serial measures were categorized as: (1) simple serial measures (no comparison); (2) serial measures with external comparison(s); (3) serial measures with internal comparison(s); (4) serial measures with both external and internal comparisons; (5) serial measures with stratified analyses; and (6) combinations of the above comparisons.

##### 4.1.1.1 Types of studies not eligible for inclusion

1. Studies measuring effects only after the intervention is applied
2. Single‐subject designs or case studies
3. Case‐crossover studies, which are typically used to assess injury etiology
4. Simple cross‐sectional and case control studies
5. Laboratory studies

#### 4.1.2 Types of participants

The population of interest in this review was limited to working populations. Accidents at work are thus limited to those engaging in the work, including voluntary as well as unpaid employees. This also includes any subsets or special populations of participants, such as studies that select females or young employees at worksites for interventions. The review was not confined by the geographical location of the study, nor by the age or gender of participants.

The review considered only accidents at work, and did not consider accidents which occur in the home, or during leisure activity; similarly, road traffic accidents not related to actual work, such as commuting, and accidents involving third parties (such as hospital patients or pedestrians passing a construction site) were omitted.

#### 4.1.3 Types of interventions

All types of safety interventions addressing accidents at work were of interest for this review. We identified whether efforts were focused on individuals or group levels, or whether efforts were multifaceted. The safety interventions were classified into the following main categories:

*Attitude modification*: Aim to modify individual attitudes and beliefs by means of information and persuasive messages in campaigns, leaflets, booklets, films, posters, direct mail, or various counseling approaches etc.

*Behavior modification*: Aim to modify individual behavior through approaches such as, training, incentives, goal setting, feedback, and coaching. Behavior modification approaches represent an external focus that explains behavior in terms of environmental consequences, such as incentives or punishment.

*Physiological modifications:* Aim to modify the physiological capacity of individuals through various training methods, such as, endurance training (running, cycling, swimming, etc.); strength and resistance training, such as push‐ups, pull‐ups, weight training, interval training etc.; flexibility exercises, such as stretching to improve joint flexibility, which can reduce the chance of injury.

*Modifications of climate, social norms, and culture*: Aim to change the shared perceptions among employees in an organization or group of the relative priority of safety, the safety norms, or the taken for granted assumptions and values that guide safety priorities. Climate, social norms, and culture are modified through various approaches such as, coaching, feedback, and modification of safety management and leadership.

*Structural modification*: These approaches seek to modify contextual factors through legislation, regulation, enforcement, economic incentives and/or other types of nonlegal modification that influence the organization of work safety, physical environment, engineering, modification of equipment and products, or the influence of sectorial‐ or societal‐level norms, and expectations that impact organizational preferences and world views.

*Multifaceted approaches*: where a mix of the above approaches was used, focused on individual or groups only, or across both domains.

Usually, more than one type of safety intervention is included in a safety intervention program (multifaceted approach). Each type of safety intervention described above may consist of one or more components (multifaceted). A conceptual model, based on Lund and Aarø (2004), is presented in Figure 1, which indicates some possible pathways to prevent accidents. The organizational and physical environment, as well as behaviors of members of the organization, provides the main *risk factors* for accidents at work. Attitudes and beliefs at the individual level, and social norms, climate and culture at the group or organizational level are *process factors* that may influence the presence of risk factors at work. Guidance on the classification of safety interventions are presented in Supporting Information Appendix 12.3.

The model with the possible pathways for preventing accidents at work, (Figure 1), provides an overall framework (logic model), which has been divided into sub‐categories representing more specific types of safety interventions (Section 13.3). These specific types of safety interventions have been the subject of analysis in this review.

#### 4.1.4 Types of outcomes

A series of primary and secondary outcomes of interest were identified for inclusion in the review. Both organization level outcomes and individual level outcomes were included. All sources of outcome data, including self‐reports, were utilized.

##### 4.1.4.1 Primary outcomes

The primary outcomes of interest included:

1. Incidence of accidental work injuries causing physical harm
2. Number of lost working days due to injury events and cases of work disability
3. Proxy measures of injury incidence, such as changes in safety behaviors and/or changes in injury risk factors (proximal risk factors)

We also included safety behavior and relevant injury risk factors as primary outcomes, as they are considered proxies for accidents at work (Laitinen et al., 2003; Laitinen & Päivärinta, 2010). Outcomes of mental or psychological harm resulting from an accident were excluded from the review.

##### 4.1.4.2 Secondary outcomes

The secondary outcomes of interest included changes in knowledge and attitudes as well as changes in workplace norms, climate, or culture

Accidents at work are relatively rare events, from a statistical perspective. Safety‐related behavior and workplace risk factors are, consequently, often used in the evaluation of safety interventions. In cases where we might identify changes in intermediate measures, such as knowledge levels, safety behaviors or climate, as well as in injury rates, this would add significantly to the knowledge of work injury prevention efforts.

##### 4.1.4.3 Outcome measures

(**NOTE:** *Effect measures are described more specifically in Section* 4.3.5)

The primary outcomes of safety interventions are typically measured as dichotomous (binary) data on an individual basis (e.g., an individual is either injured or not in the timeframe of interest). However, on the group or organizational level, injury incidence rates are of primary interest. The rate may be expressed in many ways typically taking the form of the *number of injuries/population size in a given time period*. Cases of work disability and number of lost working days can be expressed similarly. It is not uncommon for these rates to be calculated based on a denominator of person‐time, such as hours of work. Prevalence rates (%) are acceptable, given a stable work population over time. Changes in a relevant injury risk factor or safety behavior may also be measured as a binary variable (exposed—yes or no, or safe/correct behavior—yes or no) or as a percentage of improvement.

Measures of knowledge, attitudes, climate, and culture are typically expressed continuously where data can take any value in a specified range, for example, a safety climate scale. Measurement of safety attitudes as well as climate and culture in the context of safety intervention research are evolving constructs (Flin et al., 2000; Kines et al., 2011; Seo et al., 2004). We expect to see them measured in a variety of ways depending on the context of the work and the specific research questions. Consequently, we did not restrict our assessments to any pre‐specified scales or indices.

The duration of follow‐up was extracted for each study, allowing us to examine outcomes across a variety of time points. We assumed that the lag from intervention to effect would vary depending on the main types of intervention. Usually, interventions directed at the individual level would have a shorter lag than interventions directed at the group or organizational level, as for the latter there is usually a more complex relationship between the intervention and the outcome, and thus requiring a longer timeframe for implementation. Also we assume that there is a differential effect of interventions over time.

For all types of safety interventions we examined outcomes at the following time points: post‐test (immediately after the intervention ends); short‐term (up to 12 months); medium‐term (from 1 to 3 years); and long‐term (with a follow‐up longer than 3 years). For example, we usually expect a longer time frame before legislation and enforcement will effect on accidents and that there may be differential effect at different time points.

### 4.2 Search methods for identification of studies

#### 4.2.1 Electronic searches

Relevant studies were identified through electronic searches of bibliographic databases, government policy databanks, and Internet search engines. We included gray literature by, for example, searching OSH ROM and Google. No language or date restrictions were applied to the searches. All searches were done between February and July of 2015.

PubMed 1966–to 4th March 2015 (includes MEDLINE)
Embase 1980–to 30th April 2015
CINAHL 1981–to 9th July 2015
EI Compendex (no access to database)
OSH ROM (we searched in NIOSHTIC 1977–present, HSELINE 1977–present, CIS‐DOC 1974– For all: 24 April 2015)
PsycINFO 1806–20th February 2015
EconLit 1969–9th July 2015
Web of Science 1969–18th Marts 2015
ProQuest (dissertations: http://dissexpress.umi.com/dxweb/search.html). 1861–26 June 2015 (full text from 1997).

The websites of the following organizations were searched for relevant documents (between February and July of 2015):

World Health Organization (WHO)
European Agency for Safety and Health (OSHA)
European Agency for the Improvement of Living and Working Standards (Eurofound)
International Labor Organization (ILO)
Safetylit.org
Organization for Economic Co‐operation and Development (OECD)
National Institutes of Occupational Safety and Health (NIOSH)
Cochrane Central Register of Controlled Trials

#### 4.2.2 Search terms

The search strategy that was used for MEDLINE is provided in Supporting Information Appendix 12. It was modified and adjusted, where necessary, for the other databases. We used trial filters that allow non‐randomized studies and simple BA studies to be included in the review. The filters were based on the “The Cochrane Highly Sensitive Search Strategy for identifying randomized trials in MEDLINE” (Higgins & Green, 2008, chapter 6). However, its ability to identify ITSs and CBAs is not so well known in terms of sensitivity and specificity (Fraser & Thomson‐O'Brien, 2000). The search strategy in this review considered both sensitivity and specificity of searches (Verbeek et al., 2005), and the resources allocated to the project.

The retrieved reviews in the searches will be kept in a separate database for further search of relevant studies. The database will be screened for relevant reviews, and relevant studies will be selected from the reviews, following the procedure described in 4.3.1.

#### 4.2.3 Searching other resources

Literature searching in the field of accidents at work cannot be limited to database searches, as much of the literature is not well‐indexed. We therefore used supplementary search methods to capture relevant literature in the field.

We used the Google search engine (google. com) with selected terms from the above strategy to search the gray literature and to attempt to identify further unpublished studies. The first 100 hits of the Google search were included in the search. We also examined the reference lists of any relevant review we identified. We did not conduct hand searches, but did search the OSH ROM, which covers a wide range of publications, including gray literature.

### 4.3 Data collection and analysis

#### 4.3.1 Selection of studies

Literature screening was done at three levels (on the basis of title, abstract, and full text). At the first level, pairs of reviewers (JDY, KBF, HJL, ADZ, PKI, KNI, PEP, AHR, SSP, NNH, and KRA) independently read titles of reports and articles identified in the search to exclude reports that were clearly irrelevant. A report only moved on to the second screening level if the answer was “yes” or “uncertain” to the question of whether the study reports on accidents at work.

At the second screening level, reviewers in pairs (PKI, JDY, HJL, KNI, ADZ, SSP, UGE, PEP, AHR, NNH, KBF, MTÖ, and KRA) independently evaluated the report on the basis of the abstract. Uncertain reports from level one were evaluated again based on the abstract, and only retained if they were about accidents at work. At the second level the eligibility inclusion criteria were extended, and a report only moved to the third level of screening if the answer was “yes” or “uncertain” as to the question of whether the study involved evaluating of a safety intervention aimed at preventing accidents at work.

At the third screening level, studies were evaluated on the basis of the full text by reviewers in pairs (KNI, KRA, HJL, FWG, JLU, MTÖ, KJM, DZO, KBF, UGE, PKI, and JDY). Uncertain reports from level two were evaluated again based on the full text, and only retained if they were about the evaluation of a safety intervention at work. At the third level the eligibility inclusion criteria were extended to the following; the study meets the study design inclusion criteria (see Sections 2.3 and 12.3, Q20 Guidance box).

In the event of disagreements, between the reviewers in the pairs, a third reviewer and content specialist (KNI, KRA, HJL, FWG, JLU, MTÖ, KJM, DZO, and PKI) was consulted and consensus was sought through discussion. Exclusion reasons for studies that otherwise might be expected to be eligible were documented and presented in Table 9.2 and studies awaiting classification are presented in Table 9.3. The overall search and screening procedures are presented in the study protocol (Dyreborg, 2015). The inclusion coding questions for levels 1, 2, and 3 screening were piloted and adjusted if required.

#### 4.3.2 Data extraction and management

Pairs of reviewers (HJL, FWG, JLU, MTÖ, KRA, KNI, DZO, OOL, UGE, KBF, SCO, LPO, ALS, PKI, and JDY) independently extracted and coded data from the included studies. The data extraction sheet was piloted on several studies and revised as necessary. Extracted data were stored and managed electronically (JDY, ADM). Disagreements that could not reach consensus through discussion were resolved by consulting an independent reviewer with extensive content and methods expertise (HJL, DZO, OOL, and JDY). Data and information were extracted on: type of industry (NACE), type of work settings, the characteristics of participants (age, gender, other), types of intervention component(s) included (by use of classification), theoretical basis for approach, contextual detail provided by investigators, fidelity of intervention, control conditions, research design, risk of bias (RoB) and potential confounding factors, outcomes, and results. Additional items were extracted for studies with serial measures including approaches taken by investigators to improve internal validity, assessments of reasonable statistical approaches and overall inference, and whether the study could have been conducted using an experimental controlled design. Additional text was extracted to capture potential “lessons learned” in the narrative review process (see overview in Supporting Information Appendix 12.4).

#### 4.3.3 Assessment of RoB and overall quality in included studies

We assessed the methodological quality of RCTs, quasi‐randomized RCTs, and CBA (quasi‐experimental studies), by using the RoB model in the Cochrane Handbook for Systematic Reviews of Interventions (Higgins & Green, 2008). We assessed the methodological quality of serial measures, by using the seven‐standard RoB criteria for ITS studies based on the Cochrane EPOC Review group (EPOC, 2016).

RoB assessment of RCTs, quasi‐randomized RCTs, and CBA study designs were based on six main dimensions, using assessment questions with a rating of low risk, high risk, and uncertain RoB/not reported, that was piloted and modified (RoB table in Supporting Information Appendix 12.5). Pairs of reviewers (HJL, JLU, MTÖ, KNI, KRA, SCO, LPO, ALS, OOL, PKI, and JDY) independently assessed the RoB for each of the included studies using a consensus approach. Disagreements were resolved by a third reviewer with content and/or statistical expertise (KNI, HJL, HBA, and JDY) if consensus could not be reached. We have reported the RoB assessment for each study included in the review.

We judged the overall quality of an RCT or CBA study to be *high* if minimum eight out of the following eleven items were rated, as low RoB: sequence generation; allocation concealment; equivalent groups; blinding of participants; blinding of outcome assessors; statistical analysis; incomplete outcome data; selective reporting; other potential sources of RoB; the intervention has been adequately implemented (intervention fidelity); it has been clearly stated why the intervention should work (intervention rationale: theoretical concepts or description of intervention). If a minimum of six out of the eleven dimensions were rated low RoB, we judged the overall quality of an RCT or CBA study to be of *moderate* quality, otherwise, *low* quality.

For serial measures we used the seven‐standard RoB criteria for ITS studies based on the Cochrane EPOC Review group (EPOC, 2016): History (maturation); pre‐specified shape of the intervention effect; intervention affect data collection; knowledge of the allocated interventions (detection bias); incomplete outcome data (attrition); selective outcome reporting (reporting bias); and other RoB. In addition to these seven standard RoB criteria, we evaluated: whether external or internal comparison conditions were utilized to strengthen the internal validity or the use of stratified analyses within the group (statistical methods); that intervention has been adequately implemented (intervention fidelity); and that it is clearly stated why the intervention should work (theoretical concepts or description of intervention). With serial measures (ITS studies) we judged the overall quality to be *high* if a minimum eight out of the ten items were rated as low RoB/or “yes” that external or internal comparison conditions were used.

#### 4.3.4 Level of evidence

For each type of safety interventions, we assessed the level of evidence for the effect of this intervention, based on the assessment of quality for each safety intervention included (see previous section). We adjusted the methodology suggested by Tompa, Trevithick, et al. (2007) to evaluate the *level of evidence* for the effect size of each type of safety interventions: We judged *strong evidence* if the effect size was supported by a minimum of three studies with high‐quality, and reporting consistent findings. We judged *moderate evidence* if the effect size was supported by at least two high‐quality studies or three studies of medium and high‐quality, with consistent findings. We judged *limited evidence* if the effect size was supported by at least one high‐quality study or two studies of medium and/or high‐quality, with consistent findings. If findings from medium and high‐quality studies did not have consistent findings, we judged that there was *mixed evidence* for the effect of a safety intervention. If a safety intervention was only supported by one moderate quality study or any number of low quality studies, we judged the safety intervention to have *insufficient evidence* for an effect. Consistent findings were reached when point estimates from the included studies favor the intervention or the control.

#### 4.3.5 Measurement of the effect of safety interventions

The main part of the studies used dichotomous outcome data, either injury rates (80%) for the outcome measure or proxy outcomes, such as changes in safety behavior and/or changes in more proximal risk factors (17%). A smaller part of the studies used continuous outcome data (3%), such as safety climate scales.

For dichotomous outcome data, such as having an accident or not having an accident, we used risk ratios (RRs) or odds ratios (ORs) with 95% confidence intervals (CIs). These effect measures may compare the same population in different periods (before and after the intervention) or different populations (exposed vs. not exposed). Time‐to‐event (survival data) measures using person‐time in the rate denominator were included as well. Reported effect measures were plotted as point estimates, or described in detail when they could not be calculated or included in the meta‐analysis.

For the continuous data we used the mean difference (MD) or the standardized MD (SMD) for different scales (Higgins & Green, 2008), with their standard deviations (SD). In cases where means and SDs were not available, we used the methods suggested by Lipsey and Wilson (2001) to calculate SMDs from, for example, *F*‐ratios, *t*‐values, *χ* ^2^ values, and correlation coefficients. The differences in sample sizes were considered by using Hedges' (adjusted) “g” (inverse variance weight). The direction of a scale or other types of measures were adjusted by multiplying the mean values from one set of studies by “–1.”

Nearly all safety interventions were at group or organizational level, and we assumed some time before changes in outcomes could be expected. For this reason, we categorized follow‐up as short‐term up to 1 year, medium‐term from 1 to 3 years, and long‐term with follow‐up longer than three years. Only one study used the immediate effect (Jensen et al., 1997), and follow‐up for this intervention was Posttest (immediately after intervention period).

#### 4.3.6 Unit of analysis issues

It is common in the safety science field that interventions are directed at the workplace or organizational level. We found no studies where the unit of analysis was at the individual level. In cases where the clustering effect was not controlled for, that is, when unit of analysis was wrongly set at the individual level (Cheng & Chan, 2009; Daltroy et al., 1997; Porru et al., 2011; van der Molen et al., 2011), we estimated the intra‐cluster correlation coefficient from similar studies, and entered these data (design effect) into Review Manager (RevMan, 2014) to analyses effect sizes and CIs using the generic inverse variance method (Higgins & Green, 2008: section 16.3.3).

Where applicable we pooled multiple intervention groups (with different individuals, but the same intervention) within a study with one control group. No RCT or CBA studies used multiple control groups, and we identified no studies with overlapping samples for these two study designs. This was not the case for serial designs, where multiple control/comparison groups were sometimes used; these sometimes included both internal and external comparisons (Dyreborg, 2015). We performed separate analyses for control groups representing usual or an alternative intervention comparison group including one or more prevention program components.

#### 4.3.7 Dealing with missing data and incomplete data

We assessed the level of missing data and the degree of attrition (dropouts) for each of the included studies. Attrition rates and reasons for attrition were noted and included in the RoB evaluation, and considered where possible. We have not imputed any outcome data.

We noted information on intention to treat analysis (ITT), and in studies where ITT analysis was not used (Jinnah et al., 2014; Kines et al., 2013; van der Molen et al., 2011), we only included a study if we considered that the lack of intention to treat analysis was not affecting results (Higgins & Green, 2008).

#### 4.3.8 Assessment of heterogeneity

Heterogeneity in effects of safety intervention included in the meta‐analysis were assessed visually (Forest plots) along with the *χ* ^2^ (Q) statistic and *p*‐values, and the *I* ^2^ statistics and the *τ*‐squared statistics (Higgins, 2008), which are included in the Review Manager standard analyses. Heterogeneity is here understood as any kind of variability among studies in a systematic review, caused by variability in the participants, outcomes, type of safety intervention, the setting and intervention fidelity, or caused by the methodological approach, such as study designs and RoB.

The *I* ^2^ computes approximately the proportion of variation due to heterogeneity rather than sampling error. Percentages over 75%–80% may suggest heterogeneity concerns. In particular, we found high levels of heterogeneity for multifaceted safety interventions, where the number and types of components vary between studies.

#### 4.3.9 Assessment of publication bias

Publication bias can be assessed given adequate numbers of studies with appropriate data. We refrained from assessing publication bias, as we did not find enough studies with appropriate data for the various types of safety interventions.

### 4.4 Data synthesis

A subset of studies with RCT and CBA designs has been included in meta‐analyses. Studies using serial measures have not been considered for meta‐analysis. Two or more studies that we considered similar in terms of “type of safety intervention,” control conditions, outcome and follow‐up time, were combined in meta‐analyses. We used the safety intervention classification in Supporting Information Appendix 12.3 that groups safety interventions with the same type of mechanisms and theoretical foundations, which are expected to provide similar effects across settings. Only studies with similar control conditions (relative or absolute effects) were combined, and studies with similar outcome measures (injuries or safety and behavior).

As we assume that safety interventions have different lag phases and differential effects over time, we only combined outcomes at similar time points (follow‐up size bands). We are aware of the problem of repeated measures between different time points, and we have performed separate analyses for each time point in a study. Only one study had more follow‐up times, but we only included one of them in the analysis (Levine et al., 2012).

Due to the expected variation in types of safety prevention programs, combination of program components (complex interventions), implementation of programs (fidelity), and that interventions take place in a natural setting (workplaces), we have used a random effects analysis when synthesizing average effect sizes, as stated at protocol stage (Dyreborg et al., 2015).

However, if the combined effect measures provided high heterogeneity (*I* ^2^ > 80), we did not perform a meta‐analysis. We checked if some studies added more to the heterogeneity, and considered to exclude the studies (see also section on sensitivity analysis below).

The following types of safety interventions were combined, all using injuries as outcome:

1. Counseling approaches versus usual intervention with short‐term follow‐up (analysis A3.1);
2. Counseling approaches versus usual intervention at medium‐term follow‐up (analysis A4.1);
3. Teaching and education versus usual intervention at short‐term follow‐up (analysis A6.1);
4. Individual physical training versus usual intervention at short‐term follow‐up (analysis A11.1);
5. Enforcement of legislation versus no intervention at medium‐term follow‐up (analysis A17.1);
6. Engineering controls versus usual intervention at short‐term follow‐up (analysis A23.1);
7. Multifaceted interventions across levels versus usual intervention at short‐term follow‐up (analysis A32.1).

For the studies that we did not include in the meta‐analyses we synthesized the data by using a narrative approach. In all of the analyses, both meta‐analysis and narrative analysis, we interpreted the data with caution, and each pair of reviewers evaluated whether a reasonable inferential process was described by the investigators, including theoretical constructs, the epidemiology of the injury that is aimed to be prevented, time period of the intervention itself, the length of follow‐up, the fidelity and/or adoption of the intervention, contextual information provided by investigators to help in the interpretation of findings, and finally the statistical inference.

Information on each included study were extracted and recorded in Microsoft Excel 2010. We used Revman 5.3 and SAS 9.4, for the calculation of effect sizes.

#### 4.4.1 Subgroup analysis, moderator analysis and investigation of heterogeneity

As the number of studies is limited for each type of safety intervention, we refrained from doing subgroup and moderator analyses. We assessed the heterogeneity of the combined effect measures by eye‐balling the funnel‐plots, and tested whether some studies contributed more to the heterogeneity measures (*χ* ^2^ and *I* ^2^) and considered whether they were in fact a different type of safety intervention by re‐assessing the type of safety intervention.

#### 4.4.2 Sensitivity analysis

We conducted an overall sensitivity analysis for the types of outcome (injury outcome vs. safety and behavior outcome). We also conducted sensitivity analyses for the subset of safety interventions used in the meta‐analyses to see how robust the estimates were and to assess which studies contributed to a higher heterogeneity. For other types of safety interventions not included in the meta‐analysis we did not perform sensitivity analysis, but restricted assessment to “eye‐ball” analysis, if some studies had effect sizes that differed from the effect sizes of the remaining studies.

#### 4.4.3 Narrative analysis

##### 4.4.3.1 Studies using RCTs or controlled before and after designs

Effect size statistics was used for the various types of safety interventions. In addition, a narrative analysis of studies was conducted to capture relevant knowledge of the effects of safety interventions from studies not included in meta‐analysis. We reported these studies in accordance to intervention characteristics, intervention components and contextual factors (Lehtola et al., 2008), to evaluate the summarized effect of the various types of safety interventions.

##### 4.4.3.2 Studies using serial measurements

Rather than beginning our assessment of studies using serial measures with a fully pre‐specified assignment of categories of studies and quality criteria, we were more interested in how investigators sought to improve quasi‐experimental or nonexperimental intervention studies across a broad range of occupational safety intervention efforts designed to prevent fatal and nonfatal work injuries. To make this presentation more transparent, we reported separately on these studies that were not combined in meta‐analyses, in a manner focused on intervention characteristics and contextual factors (Lehtola et al., 2008).

For this report we included intervention evaluations with at least three measures before and three after the intervention, as well as studies with at least three measures, where one should be a premeasure, if the investigator described an approach that improved the internal validity of the study. We also included serial measures even if the interventions did not occur at a precise moment in time. Simple BA studies were not included in the analysis.

We took a grounded approach to this review activity to more clearly describe how investigators have used studies with serial measures in workplace injury intervention evaluations. This included attention to ways in which potential threats to validity were addressed. Each report was read by two members of the review team and data were independently extracted. Impressions were then compared and discrepancies discussed to reach consensus. In rare circumstances a third opinion was sought.

We attempted to capture descriptions of why investigators believe a given intervention should work, based on theoretical constructs or the epidemiology of the injury that is aimed to be prevented, and when that should be the case. We looked at the time period of the intervention itself and the length of follow‐up, how the fidelity and/or adoption of the intervention were measured or described, and the analytical approaches and tools used. We also extracted contextual information provided by investigators to justify an approach and/or help in the interpretation of findings.

Approaches taken by investigators that addressed internal validity concerns in these nonexperimental designs and improved study quality were noted. Using the information from this text review we classified the study designs into categories that captured whether comparisons or other analytical strategies were employed. Furthermore, we noted additional design or analytical features used in the overall inferential process that were considered to strengthen the work.

Along with assessment of bias (as described earlier), each reviewer assessed whether a reasonable inferential process was described by the investigators, that included, but was not limited to, statistical inference. Ways the evaluation could have been further improved were noted and consideration was given as to whether the research questions could have been addressed using an experimental approach.

## 5 RESULTS

### 5.1 Results of the search

The literature search resulted in 60,460 references and six additional references were identified through other resources. After merging of databases and removing duplicates 42,927 references were retained for title and abstract relevance screening. After screening for eligibility 485 references included for full text assessments. After full‐text assessment, 219 articles fulfilled the inclusion criteria. Of those, 25 articles were excluded with reasons or put on a waiting list for language translations. 194 studies were data extracted. The 94 identified single group studies were excluded for the synthesis analysis. These single group studies are presented in Table 16, and not further discussed in the report.

**Figure 2:**
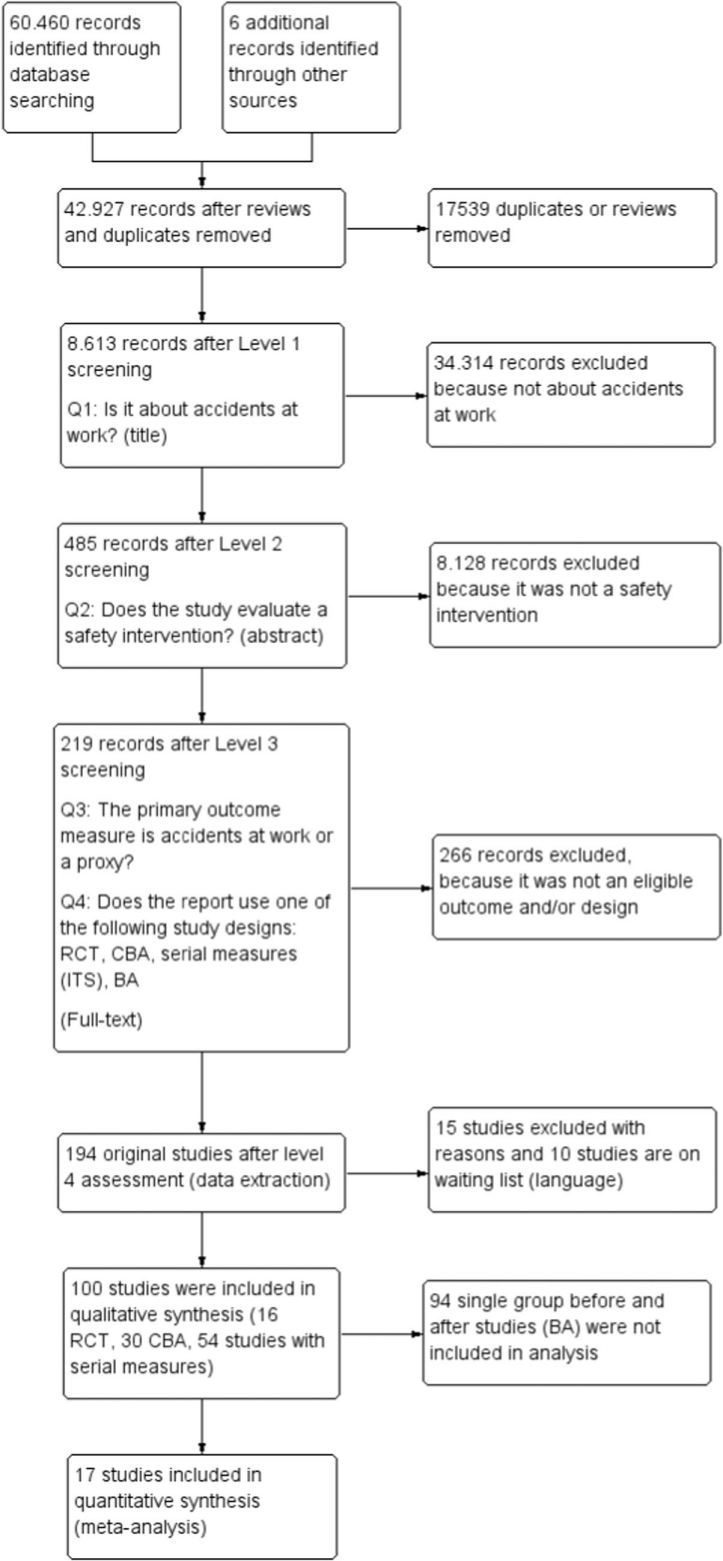
Flow diagram of the data search and screening procedure

In total 100 original studies were included for synthesis analysis, including 16 RCT study designs, 30 CBA study designs, and 54 studies using serial measures (ITS study designs) (Figure 2). These studies represented 120 reports of safety interventions. The number of participants included 31,971,908 individuals in 59 safety interventions, 417,693 groups/firms in 35 safety interventions, and 15,505 injuries in 17 safety interventions. Out of the 59 safety interventions, two were evaluating national prevention measures, which alone accounted for the 31,667,110 individuals. The remaining nine safety interventions used other types of measures, such as safety exposure, safety observations, gloves, or claim rates.

### 5.2 Description of the studies

#### 5.2.1 Included studies

##### 5.2.1.1 Overall characteristics of included studies

Section 9.1 presents the characteristics of each of the 100 included original studies. The included studies are mainly representing the western societies, as only few studies came from Africa and Asia (Table 1).

**Table 1:** Number of studies for each study design for five continents of the world

Original study continent	Study design: RCT	CBA	ITS	Total
Africa		1		1
Asia	4	2	1	7
Australia	1	2	4	7
Europe	5	8	14	27
North America	6	17	35	58
Number of studies	16	30	55	100

Abbreviations: CBA, controlled before‐and‐after; ITS, interrupted time series; RCT, randomized controlled trial.

##### 5.2.1.2 Study design

Of the 100 original studies included in the analyses 16 studies were using randomized controlled design (RCT), 30 were CBA studies, and 54 studies used serial measures (ITS).

The included RCT studies were randomized at group level, some using stratified or pair matched random samples and others simple random sampling. The 30 included CBA studies used various forms of control, such as historical controls and matched controls. The included 54 studies with serial measures (ITS) cover several different approaches. Simple serial measures taken from one group over time were more commonly used (*n* = 24). Other approaches included serial measures with internal comparison (*n* = 12), serial measures with external comparison (*n* = 10), serial measures with stratified analyses (by job, type of injury, focus of intervention, etc.) (*n* = 2), and serial cross‐sectional measures (*n* = 2). Finally, we found hybrid designs using combinations of the above (*n* = 4).

The 100 studies used for the analysis of effectiveness of safety interventions, included 120 safety interventions in total (Table 2).

**Table 2:** Number of studies included and number of safety interventions covered by these studies, for each study design

Study design	Studies	Safety interventions
RCT	16	20
CBA	30	43
Serial (ITS)	54	57
Total	100	120

Abbreviations: CBA, controlled before‐and‐after; ITS, interrupted time series; RCT, randomized controlled trial.

In the following we refer to these 120 safety interventions, if we are not specifically referring to the included number of original studies.

##### 5.2.1.3 Participants, types of work, and types of injuries

The 120 safety interventions included 31,971,908 individuals in 59 safety interventions, 417,693 groups/firms in 35 safety interventions, and 15,505 injuries in 17 safety interventions. Out of the 59 safety interventions, two were evaluating national prevention measures which alone accounted for the 31,667,110 million individuals. The remaining nine safety interventions used other types of measures, such as safety exposures, safety observations, gloves, or claim rates.

Tables 10, 11, 12, 13, 14, 15 (Section 12) provides an overview of the nature of included safety interventions using RCT, CBA, or serial measures (ITS), by type of safety intervention, including information on Participants, Intervention characteristics, Comparisons conditions, Outcome measures and Study design (PICOS).

Half of the 120 included safety interventions evaluated “all types of injuries” in the workplace (*n* = 61). The two largest specific types of injuries assessed were needlestick injuries (*n* = 20) and overexertion injuries (*n* = 20), such as dislocation, sprains, and strains, mainly in hospitals or health care institutions. Overexertion injuries were related to acute exposure in the workplace, such as the transfer or support of patients or clients. Safety interventions to prevent overexertion injuries were mainly studied in human health and social work activities, such as health care institutions. Five studies evaluated the effect of interventions on eye injuries. The effect of safety interventions on fatal injuries were only evaluated in four studies (Bulzacchelli et al., 2007; Derr et al., 2001; Menendez et al., 2012; Suruda et al., 2002), of which two were in the construction industry.

More than one‐third of the 120 included safety interventions evaluated “all types of accidents” in the workplace (*n* = 46). The two largest specific types of accidents are contact with sharp or pointed materials or tools (*n* = 23) and overexertion of the musculoskeletal system (*n* = 19), mainly in hospitals or health care institutions. Collision and other horizontal impact on body (*n* = 10) were a type of accident involving transport and vehicles, and were mainly seen in transport and storage (*n* = 7). Few safety interventions evaluated assault or violence at work (*n* = 4), where persons were exposed to assaults from patients, prisoners or customers in retail.

The main groups of participants in the included safety interventions came from health and social activities (*n* = 35), manufacturing (*n* = 22), agriculture (*n* = 14), transport and storage (*n* = 11), and construction (*n* = 10). These groups made up 77% of the included safety interventions. These five sectors are generally considered as high‐risk sectors. Mining and quarrying industry is also a high‐risk sector, but only three of the included studies evaluated safety interventions in this sector.

Most of the included safety interventions used firms as the analytical unit, sometimes worksites, such as in the construction sector, or organizational units. Nearly half of the safety interventions were evaluated in large firms or public institutions with more than 250 employees (*n* = 53). Micro firms were mainly from the agricultural sector, evaluating safety interventions on smaller (family) farms (*n* = 5). For several safety interventions the company sizes were mixed, often evaluated with an ITS design, for example, as in the evaluation of legislation and enforcement. For about 9% of the safety interventions the size of enterprise was unclear (*n* = 11).

##### 5.2.1.4 The theoretical basis or rationale for the interventions

This review used a conceptual model of safety interventions including five specific types of safety interventions and combinations of these (multifaceted safety interventions), thus providing six main types of approaches to reduce accidents at work (see chapter 2.3). The safety interventions directed at the individual level included attitude and beliefs modifications (*n* = 11), behavior modifications (*n* = 6), and modifications of physiological strengths and resistance (*n* = 5).

The interventions directed at the group or organizational level included culture, climate and normative changes (*n* = 11), and structural modifications (*n* = 51), where the latter included legislative changes and enforcement (*n* = 23), engineering controls (*n* = 19), and administrative controls (*n* = 2). Only two safety interventions included economic incentives. A large group of safety interventions investigated the combined effect of two or more safety interventions (*n* = 36), where the largest group involved safety interventions investigating the combined effects of components across individual and organizational levels (*n* = 21). In one study the type of safety intervention evaluated was unclear (Lanoie, 1992).

Most safety interventions provided some information about the rationale for the interventions, some more explicit than others. However, only a smaller subset of the studies provided a more integrated theoretical basis for why the safety intervention should work, and which was reflected in the design and measurements of intermediate outcomes.

The studies on attitudinal approaches used a theoretical or conceptual basis for the safety intervention to a limited extent. Most studies referred to common sense (Adams, 2013; Gadomski et al., 2006; Johnson & Owoaje, 2012) or following recommendations from authorities (Wang, 2003). One exception was Gregersen's (1996) study of drivers in transport, who used the insights from Kurt Lewin's (1947) theoretical and practical research on how to change habits by use of group discussions.

Evaluation of safety interventions based on changes in individual strengths and physiological readiness used references to physiological theory and principles, such as Physical Readiness Training (PRT) (Knapik et al., 2003), or knowledge about how connective tissues in tendons, ligaments, joints etc., become shortened and dense when not exercised, then limiting the range of motion, and thus risk of overexertion of muscles and tendons (Hilyer et al., 1990; Leffer, 2010).

In the 1980s and early 1990s we saw early work evaluating behavioral approaches to safety in manufacturing in the UK, Finland, and the US (Cooper et al., 1994; Fellner & Sulzer‐Azaroff, 1984; Saari, 1998; Sulzer‐Azaroff & de Santamaria, 1980; Sulzer‐Azaroff et al., 1990). The reported safety interventions had follow‐ups lasting from 4 months up to 3 years, with only one safety intervention lasting 36 month (Saari & Näsänen, 1989). Other variations of behavior based theories were referred to in the included safety interventions (Bena et al., 2009; Daltroy et al., 1997; Quintana, 1999). Jinnah et al. (2014) used a 16‐item instrument to measure farm safety behavior of fathers and youth, guided by the TPB (Ajzen, 2012). Cheng and Chan (2009) combined behavior based theories with attitudinal changes guided by the linear KAP model. Other variants of the behavior based theories included social marketing approaches, as well as Rogers's theory of diffusion of innovation (Rogers, 1995), which was used by Chapman et al. (2011) to increase awareness and encourage adoption of safer dairy farming work practices in a 7‐year follow‐up study.

After more than 20 years of behavioral safety research, safety climate interventions attempt better integration with other domains of management research. From a theoretical standpoint, safety climate created a link to important constructs of management theory, for example, leadership (Zohar, 2002). Safety climate theory or components of safety climate were investigated in a number of studies (Cooper et al., 1994; Cunningham & Austin, 2007; Fellner & Sulzer‐Azaroff, 1984; Kines et al., 2010; Moore‐Ede et al., 2004; Sulzer‐Azaroff et al., 1990; Zohar & Luria, 2002, 2003).

In the early 1990s studies focusing on reducing needlestick and/or sharps injuries appeared in correspondence to growing concern about the AIDS epidemic and later hepatitis concerns (Birnbaum, 1993; Lawrence et al., 1997; Zafar et al., 1997); these have continued (Cunningham & Austin, 2007; Phillips, 2012; Reddy & Emery, 2001; Rogues et al., 2004; Smollen, 2004; Sossai et al., 2010; Whitby et al., 2008). The latest of these is a report on effectiveness of the US Needlestick Protection Act passed in 2000. The rationale for the prevention of needlestick injuries is predominantly various forms of engineering controls, initiated by policies of the health care system or legislation (Grimmond et al., 2010; Jensen et al., 1997; Lawrence et al., 1997; Prunet et al., 2008; Reddy & Emery, 2001; Rogues et al., 2004; Smollen, 2004; Sossai et al., 2010; van der Molen et al., 2011; Whitby et al., 2008). Also teaching and educational efforts (Mehrdad et al., 2013; Wang, 2003), counseling approaches (van der Molen et al., 2011), and goal setting and feedback (Cunningham & Austin, 2007) were used to improve knowledge and attitudes and in turn safer behavior in handling needles. And finally these efforts were also combined in multifaceted approaches to prevention (Gershon, 1999; Srikrajang et al., 2005; Valls et al., 2007; Zafar et al., 1997). In these studies, investigators began making use of ongoing injury surveillance systems to address intervention evaluation.

By the 2000s studies focused on preventing injuries from lifting (transfer of patients) in health care appeared using engineering controls (Alamgir, 2008; Schoenfisch et al., 2013) or multifaceted safety interventions (Black, 2011; Chhokar et al., 2005; Evanoff et al., 1999; Fujishiro et al., 2005; Garg, 1999; Martin et al., 2009; Park et al., 2009; Passfield, 2003).

*Structural modifications* include varied approaches that change the physical, organizational, or regulatory environment. A common feature of the structural approaches is that environmental factors are changed, often over longer time periods or permanently, consequently with more profound effect. One type of structural modification is *engineering control*, for example, introduction of machine safeguards, walkways, elimination of hazardous substances or materials, and other changes in the physical environment that directly influences individuals' safety without necessarily affecting their behavior.

Preference for *engineering control* is based on the public health hierarchy of hazard control (Herrick & Dement, 1994; Lingard & Holmes, 2001). This approach follows the basic tenet of industrial hygiene regarding control of health hazards in working environments; it has been applied to the control of physical hazards responsible for energy transfer and subsequent accidents and injuries in the workplace as well (Castillo et al., 2006). Emphasis in this model is given to the most effective and efficient preventive measures that eliminate risk at the source of the hazard. Lower tiered approaches in the hierarchy control risk through barriers or use of personal protective equipment or training and education efforts. Of note, engineering controls typically focus on control of a specific hazard, in marked contrast to safety climate or culture approaches that often do not, but rather address safety more broadly.

Other types of approaches in this group are based on simple linear models, where a chain of multiple events culminate in an injury. Safety prevention is then directed at removing one or more of the elements involved in this chain of events, to prevent the occurrence of the injury. One such model is Heinrich's “Domino theory” (Heinrich, 1931), which has had a tremendous effect on practical safety interventions, and still is much in use despite numerous pitfalls that have been described (Johnson, 2011; Manuele, 2014). A further development of these models is the complex linear models, such as the BowTie model and the Swiss cheese model (Reason, 1997), that illustrate that even though there are several barriers between hazards and accidents there can be flaws (as holes in the slices of a cheese) in these barriers that co‐incidentally can be aligned and then result in accidents.

Another type of structural modification is *social control*, which introduces coercive power or incentives for people or organizations to change behavior. This is related to compliance with rules and regulations on a non‐voluntary basis, for example, by use of enforcement and legal sanctions, as well as compliance on a voluntary basis, for example, by use of marketing, economic incentives, reputations, benchmarking, and insurance‐related benefits for low risk companies. Regulation may serve as a potentially powerful institutional force to promote the adoption of occupational health and safety policies and practices (Chambers et al., 2013). The basic idea is that such instruments provide an incentive for companies or people at work to adhere to certain (legal) standards, either due to the risk of penalties in case of noncompliance, or because a benefit can be achieved in exchange for an appropriate behavior (Rothschild, 2000).

Currently, reports of the effect of legislation as a structural approach to occupational safety are conflicting, which is not surprising given the complexities involved in such evaluations for the most part. While effect variation may reflect differences in the actual implementation of the legislative approaches to prevention, a number of other factors may also be of potential importance. These include the very nature—or strength—of the legislation, and thus the requirements it is intended to impose. For example, a call for training would be expected to have a different effect than a requirement for safer equipment that removed a dangerous exposure.

In early 2000s we also began seeing studies focused on assessing effectiveness of efforts to reduce fatalities and injuries in the high‐risk construction industry, including teaching, coaching, feedback and climate interventions (Bena et al., 2009; Cheng & Chan, 2009; Derr et al., 2001; Kines et al., 2010; Lipscomb et al., 2003; Suruda et al., 2002), evaluation of regulatory acts (Derr et al., 2001; Farina et al., 2013; Lipscomb et al., 2003; Suruda et al., 2002), and multifaceted approaches (Lipscomb et al., 2008, 2010; Spangenberg et al., 2002). The first study using serial measures to assess legislative effectiveness in this industry was published in 2001 by Derr. We found no studies evaluating engineering controls in the construction industry.

##### 5.2.1.5 Comparison conditions

Most of the controlled safety intervention studies (RCT and CBA) used treatment as usual (TAU) as the main type of comparison condition (*n* = 46). However, the content of TAU varied across studies, reflecting the contextual conditions and how work and prevention are managed in particular settings (Andrée Löfholm et al., 2013). In most cases there were already some activities going on at a workplace, which were directed at the prevention of a certain problem, for example, needlestick injuries or overexertion injuries. Therefore, comparison conditions were in most cases usual activities (TAU), in only few cases (*n* = 5) no other intervention (Hilyer et al., 1990; Jinnah et al., 2014; Johnson & Owoaje, 2012; Quintana, 1999), and alternative types of safety interventions (*n* = 5) (Adams, 2013; Forst, 2004; Parker et al., 2009; Rautiainen et al., 2004). For the latter it could be cases where safety glasses were provided and only the educational components were different and thus evaluated (Adams, 2013). In some cases, it was difficult to evaluate the precise comparison condition, for example, when a historical cohort group was used (Peate et al., 2007).

##### 5.2.1.6 Types of outcomes

The main types of outcomes used for evaluation of effects of safety interventions were injuries (80%), and to a less degree “risk or behaviors” (18%). Four studies (2%) investigated the effect on fatal injuries, 92 studies (44%) investigated the effect on nonfatal injuries. The remaining reported safety interventions (55%) mixed fatal and nonfatal injuries in their evaluation of effects.

#### 5.2.2 Excluded studies

Reported safety interventions were mainly excluded from the present review due to data being insufficiently reported, or due to authors only reporting after‐only data (Azar‐Cavanagh et al., 2007; Hall et al., 2013; Jagger & Bentley, 1997; Miller et al., 2006). In addition, some reported safety interventions were excluded as in the end we did not consider them eligible, for example, as they were in fact not addressing acute accidents at work, but rather injuries caused by longer‐term exposures (Lavender et al., 2007). In other cases, the report included too little information about the study, for example, abstracts and poster presentations (Lim, 2011; Markovic‐Denic et al., 2011; Mobasherizadeh et al., 2011).

#### 5.2.3 Studies awaiting classification

A few studies that appeared relevant from abstract review were not analyzed (Bena et al., 2009; Benavides, 2007; Hernández Navarrete, 2010; Lanoie & Streliski, 1996; López‐Rojas et al., 2013; Porru et al., 2009; Urban et al., 2012); these were all in a language that none of the authors managed to a sufficient level to allow a reasonable review.

### 5.3 RoB in included studies

Various types of RoB were evaluated for RCT, CBA and serial measures study designs (ITS). For the serial measures we have used the EPOC criteria (EPOC, 2016).

#### 5.3.1 Types of bias assessed

##### 5.3.1.1 Selection sample bias

For the dimension *selection sample bias* three domains were assessed that each can introduce sample bias: sequence generation, allocation concealment and equivalent groups. Both randomly and non‐randomly, selection can introduce bias in estimation of effects of safety interventions.

###### 5.3.1.1.1 Sequence generation

We considered low RoB from sequence generation for all RCT studies, apart from Cheng and Chan (2009) and Parker et al. (2009), where participants for both studies in practice were engaged on a voluntary basis, and for the latter we questioned whether the randomization had worked. The CBA studies were considered high‐risk if it was not clear whether the procedure could produce comparable groups and the risk of confounding factors were considered high. Only a smaller number of CBA studies met this criterion (Gregersen, 1996; Hilyer et al., 1990; Johnson & Owoaje, 2012; Levine et al., 2012; Peate et al., 2007; Santaweesuk et al., 2014). We did not evaluate the serial measures study designs (ITS studies) for this type of bias, but used the EPOC criteria (EPOC, 2016).

###### 5.3.1.1.2 Allocation concealment

We assessed that the RoB due to allocation concealment was high for the majority of the CBA safety interventions; only in a few cases did we judge the RoB low (Harms‐Ringdahl, 1987; Levine et al., 2012; Quintana, 1999), despite allocation was not concealed. In half of the safety interventions based on RCT design we judged high risk that allocation was not concealed.

###### 5.3.1.1.3 Equivalent groups

We evaluated the baseline differences between intervention and comparison groups for all studies with a control group (RCT and CBA), and judged whether authors had adequately accounted for differences. For two of the RCT studies and seven of the CBA we assessed high RoB, due to non‐equivalent groups that had not been taken into account in the effect assessment. In one RCT study (Rautiainen et al., 2004), the farms did not represent the general Iowa farm in size, as they were larger farms, and in another (Srikrajang et al., 2005) the groups were different on several items. For three CBA studies types of work and pre‐intervention injury rates were higher in intervention groups than in the control groups (Black, 2011; Harms‐Ringdahl, 1987; Lopez‐Ruiz et al., 2013). In one CBA study there were significant gender and work‐time differences (Carrivick et al., 2002); in another there were differences in the fitness of control and intervention groups, as intervention soldiers were less fit on entry compared with their historical control counterparts (Knapik et al., 2004). In one case it was difficult to know whether it was equivalent groups, when no information on the historical control group was provided (Peate et al., 2007).

##### 5.3.1.2 Performance bias

For most of the safety interventions (*n* = 114) we judged a high RoB that the participants were not blinded, including all safety interventions using RCT study design. Only in few cases we assessed low RoB that the participants knew about the safety intervention. Clearly, the social context of work makes it very difficult to blind participants. There are also ethical reasons for informing participants of the safety interventions they are involved in.

##### 5.3.1.3 Detection bias

Detection bias is concerned with systematic differences between groups in relation to how outcomes are determined, including blinding of outcome assessors and the statistical analysis. Variation in reporting of accidents over time is a concern in evaluating the effects of safety intervention, as safety interventions could influence reporting propensity in the intervention group. We considered this a problem in several studies (Cheng & Chan, 2009; Mehrdad et al., 2013; Rasmussen et al., 2006). In Wang (2003), students reported their behavior and their number of injuries. This is likely to cause underreporting of accidents in both groups.

Blinding of outcome assessors was judged as a high RoB in the following studies (Forst, 2004; Harms‐Ringdahl, 1987; Hilyer et al., 1990; Kines et al., 2010; Mattila & Hyoedynamaa, 1988; Mehrdad et al., 2013). In cases where the intervention, data collection and management of data were done by the same researchers, we considered detection bias as a high RoB.

Some studies were judged at high RoB as appropriate methods were not used to take into account the appropriate unit of analysis (Black, 2011; Cheng & Chan, 2009), or censoring time to event data (Evanoff et al., 1999).

##### 5.3.1.4 Attrition bias

As safety intervention studies often require participants to change the way they usually work, this can result in their dropping out. Consequently, the characteristics of the intervention group can change, if there are systematic differences between dropouts and completers. Some studies provided useful information on employee turnover, dropouts and percentage of completers, but if this information is not available it can be difficult to judge the attrition bias, and there is a risk of underestimating the RoB.

If loss to follow‐up was not taken into account in the analysis we considered this a high RoB, which was the case for two RCT studies (Kines et al., 2013; van der Molen et al., 2011) and unclear in one RCT study (Zohar, 2002). In four CBA studies we considered this a high RoB (Black, 2011; Evanoff et al., 1999; Forst, 2004; Mehrdad et al., 2013), and unclear for two studies (Hilyer et al., 1990; Ray et al., 1997). Three studies did not use intention to treat analysis, and we did not have data to correct this (Evanoff et al., 1999; Kines et al., 2013; van der Molen et al., 2011).

##### 5.3.1.5 Reporting bias

Selective reporting of outcome data and results can be difficult to assess if there is no protocol published beforehand. Only in recent years, have investigators even considered publishing workplace study protocols in advance. We checked that pre‐specified primary outcomes were reported. We also checked whether the pre‐specified severity of accidents or the pre‐specified types of accidents were considered. We identified four CBA studies (Haviland et al., 2012; Mattila & Hyoedynamaa, 1988; Parker et al., 2009; Wang, 2003) and one RCT study (Adams, 2013), where we judged high RoB caused by selective reporting of study outcomes. In one of these (Parker et al., 2009) not all results from the intervention companies were presented in the estimation of effects.

##### 5.3.1.6 Fidelity of intervention

For assessment of the fidelity of intervention we judged whether the target population was given the safety intervention and/or the target population adopted the safety intervention (implementation of intervention). If one of these two criteria were not met we judged the fidelity of intervention as high RoB. We judged high RoB for 25% of the studies due to the intervention not being clearly provided and/or adopted by the target population. In 30% of the studies this information was unclear from the study report.

In some cases we were informed that the intervention groups received the intervention, but no information on whether it was adopted (Daltroy et al., 1997; Kim et al., 2004; Mehrdad et al., 2013; Peate et al., 2007; Zohar, 2002). In other cases we had no information on the degree of the actual implementation, for example, use of eyewear (Forst, 2004); whether firefighters maintained exercises throughout the intervention period (Hilyer et al., 1990); enforcement was not documented (López‐Ruiz et al., 2013); whether older non‐safe devices were removed in engineering approaches (Valls et al., 2007); or whether provided equipment were in fact being used (Black, 2011; Santaweesuk et al., 2014). In one case improvement of safety knowledge was measured, but the intervention was about change in behavior, and this was not documented (Wang, 2003). Following this we judged the fidelity of interventions as unclear RoB. In a study of the influence of weight loss on reduction of work injuries, there was no documentation on whether weight loss was maintained in the follow‐up year (Morgan, 2012), which we considered a high RoB.

##### 5.3.1.7 Intervention rationale

For the assessment of the intervention rationale we evaluated whether it has been clearly stated why the intervention should work, including a reasonably description of the theoretical or conceptual elements. We also accepted plain arguments for why an intervention should work, if it was reasonably clear. If one of these two criteria were not met we judged the intervention rationale as high RoB. We only judged high RoB for 8% of the studies where it was not clearly stated why the intervention should work. In 3% of the studies this information was unclear from the study report. This means that most studies have some reasonably description of why the intervention should work. However, in 30% of the safety interventions neither a theoretical nor a conceptual approach were used as a background for the intervention. In some cases the effect was taken for granted, as in the case of the effect of legislative efforts (Derr et al., 2001; Farina et al., 2013; Haviland et al., 2012; Levine et al., 2012).

##### 5.3.1.8 Other potential sources of bias

In half of the safety interventions risk of other potential sources of bias was judged to be high. In one study we judged that the “Hawthorne effect” was a RoB, as strong involvement of researchers could have influenced outcomes (Cheng & Chan, 2009). We did not consider self‐report a bias in itself, but in some cases we judged that it was likely that the intervention contributed to an increased reporting of cases of injuries in the intervention group (Daltroy et al., 1997), or that outcome data, such as self‐reported injuries (Gadomski et al., 2006; Rasmussen et al., 2003; Rautiainen et al., 2004) were not a reliable measure, or that different seasons for data collection in the control and intervention groups were a threat (farming), even though statistical adjustments for time at risk were made (Rasmussen et al., 2003). Knowledge of intervention in control group (bleed over) can bias results toward null, in particular when the control and intervention groups are in the same location (Harms‐Ringdahl, 1987; van der Molen, 2011). In some other cases, we judged that the intervention likely influenced self‐reported outcome data (Mattila & Hyoedynamaa, 1988), including self‐reported intermediate measures (Wang, 2003). In the latter case, students reported their behavior and their injuries, and we judged that this is likely to have caused underreporting of both. In one study, we judged that economic incentives for participation likely influenced the reporting of injuries (Rautiainen et al., 2004). In a study on effects of health education on the riding habits of commercial motorcyclists in Uyo, southern Nigeria (Johnson & Owoaje, 2012), we judged a high RoB, as self‐reported riding behavior in face‐to‐face interviews is likely to cause underreporting of cases.

In Knapik et al. (2003) we considered it a high RoB that the control group had more than double the number of running miles compared to the intervention group, as an increase in running miles would increase the risk of traumatic overuse injuries, and this was not taken into consideration in the statistical model.

Another important aspect was potential for regression‐to‐the‐mean, as intervention activities usually were initiated as a result of high injury rates. We judged high RoB due to regression to the mean in four studies (Black, 2011; Kim et al., 2004; Levine et al., 2012; López‐Ruiz et al., 2013).

#### 5.3.2 Assessment of bias by study design

##### 5.3.2.1 RCTs

**Figure 3:**
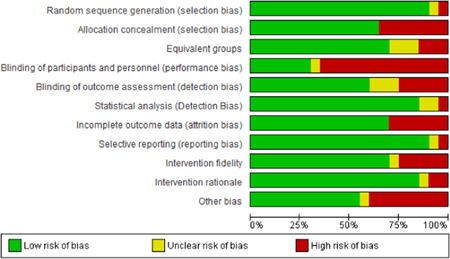
Risk of bias graph in percentage for each item across all RCT studies, based on review authors' evaluation of each study. RCT, randomized controlled trial

The RoB assessments for the 20 included RCT safety interventions are shown in Figure 3.

**Figure 4:**
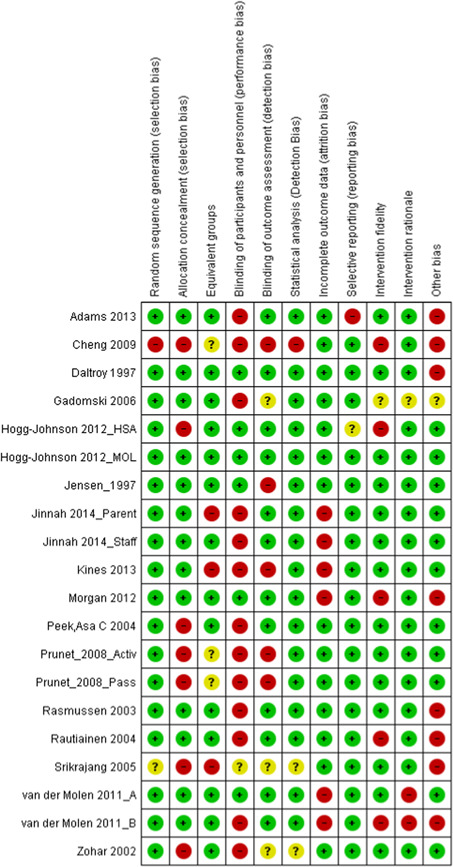
Risk of bias for each study using randomized controlled trials (RCT) study design

The most common RoB identified was “other potential sources of risk of bias,” where nearly half of the studies were judged to have high RoB (Adams, 2013; Cheng & Chan, 2009; Daltroy et al., 1997; Morgan, 2012; Parker et al., 2009; Rasmussen et al., 2003; Rautiainen et al., 2004; Srikrajang et al., 2005; van der Molen et al., 2011), rather than those sources of bias that were specifically listed and assessed. Examples of other risks of bias are: that intervention has contributed to increase reporting of claims in the intervention groups; bleed‐over between groups; the Hawthorne effect, external validity concerns, and selection bias caused by the intervention. RoB for the 20 RCT study designs is shown in Figure 4.

##### 5.3.2.2 Controlled before and after (CBA) study designs

**Figure 5:**
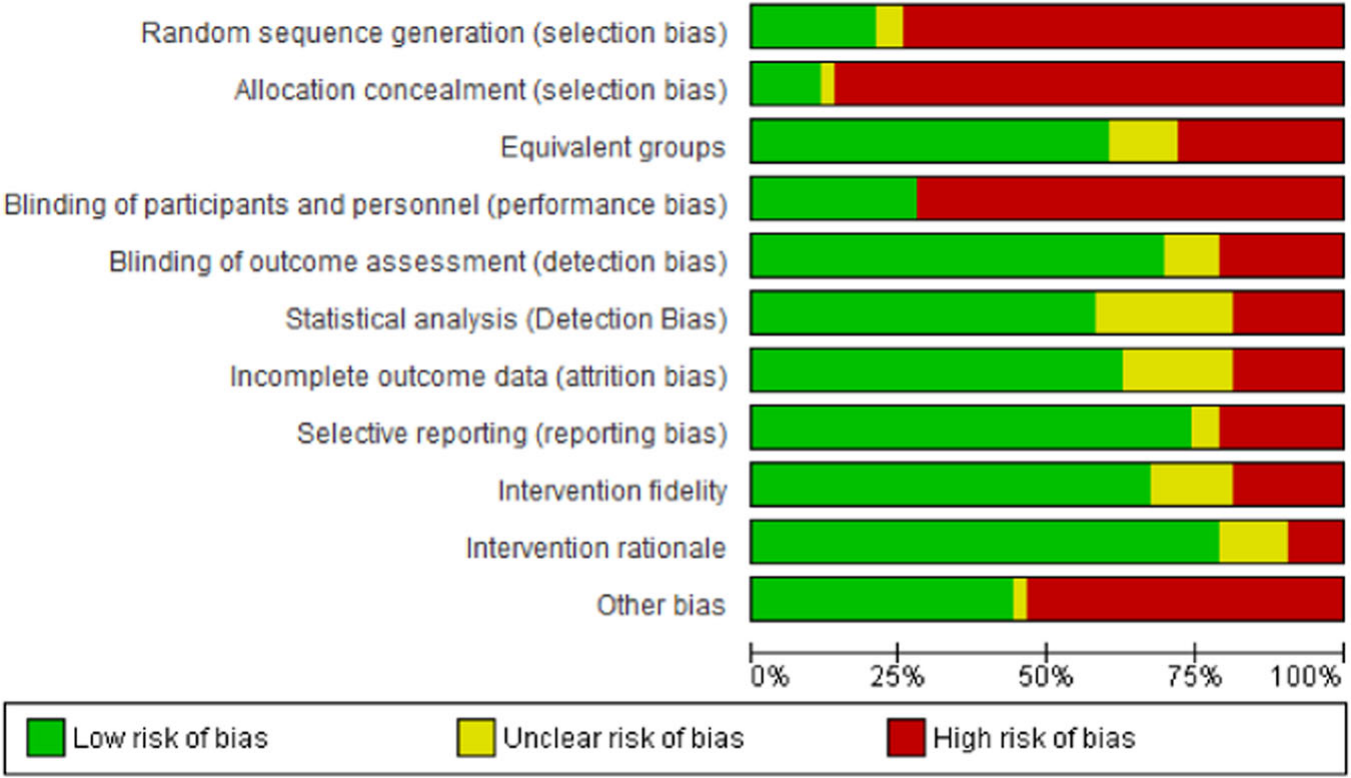
Risk of bias (RoB) for Controlled Before and After (CBA) study designs: RoB graph in percentage for each item across all CBA studies, based on review authors' evaluation of each study

As safety interventions using CBA study designs do not use randomization, they will in general be assessed with higher RoB compared to RCT designs. However, in a few of the studies we judged that a non‐randomized selection was not likely to be significantly compromised (Gregersen, 1996; Hilyer et al., 1990; Johnson & Owoaje, 2012; Levine et al., 2012; Peate et al., 2007; Santaweesuk et al., 2014). In 10 out of the 39 CBA safety interventions we judged that there was a high RoB due to nonequivalent groups, some due to differences in injury rates at baseline (Black, 2011; Carrivick et al., 2002; Harms Ringdahl, 1987; Lopez‐Ruiz et al., 2013), or because the historical control group had unknown risk at baseline (Peate et al., 2007) (Figure 5).

**Figure 6:**
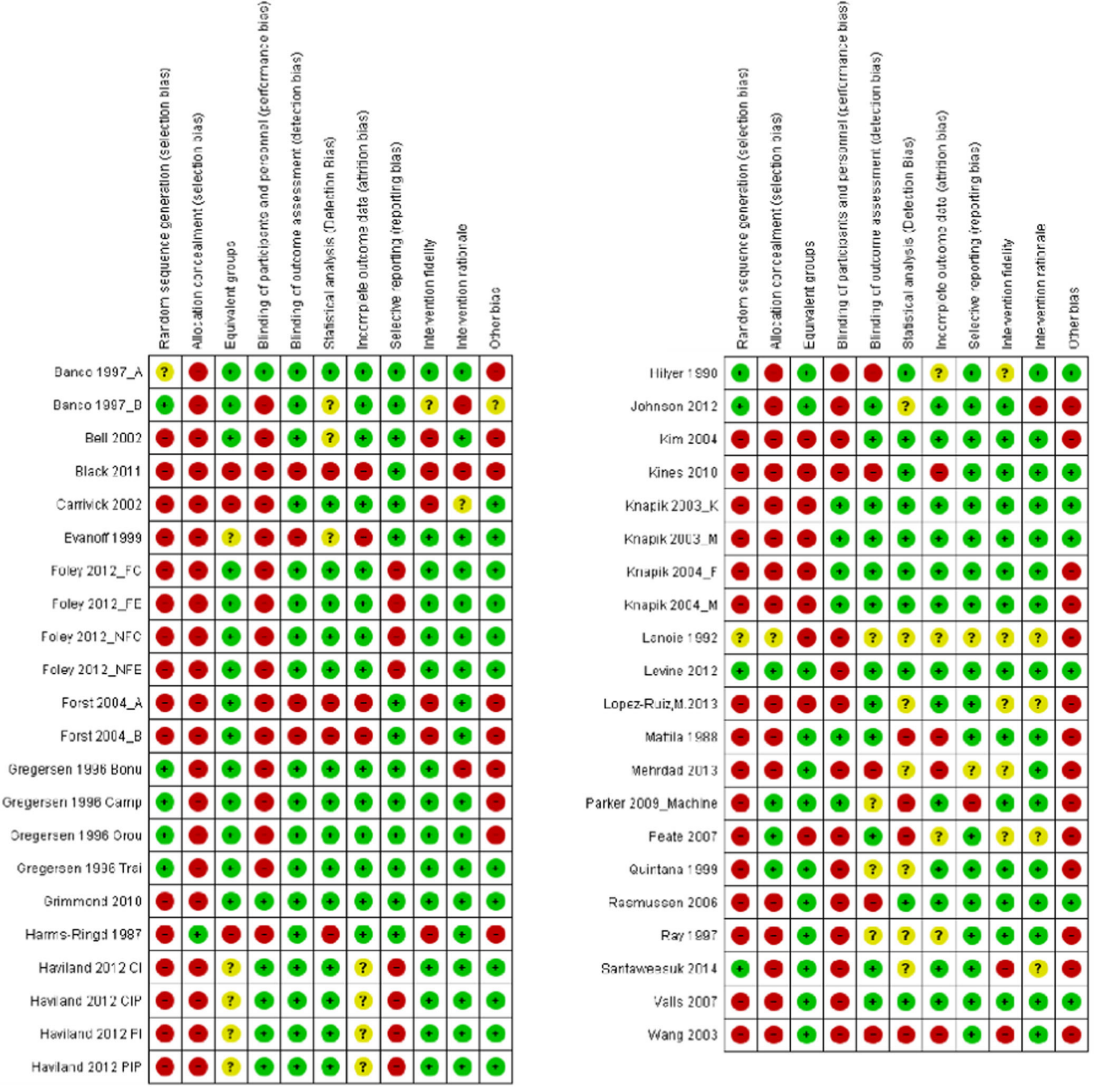
Risk of bias for each study using Controlled Before‐After (CBA) design

Risk of attrition was only judged to be high in four cases (Black, 2011; Evanoff et al., 1999; Forst, 2004; Mehrdad et al., 2013), where loss to follow‐up were not accounted for in analyses, or where high turnover questioned the representation of the sample (Black, 2011). Half of the CBA studies suffered from other potential sources of bias, where regression to the mean was an important RoB for some studies (Black, 2011; Harms Ringdahl, 1987; Kim et al., 2004; Lopez‐Ruiz et al., 2013), whereas others were likely to have serious risk of reporting failures (Mehrdad et al., 2013; Rasmussen et al., 2006). RoB for each CBA study design is shown in Figure 6.

##### 5.3.2.3 Serial measures (ITS) studies

The more common concerns regarding systematic error, or bias, in the studies with serial measures included maturation and possibilities that the intervention affected reporting of outcomes. Based solely on longer observation, potential maturation risks were not unexpected. However, some investigators effectively countered this issue through the use of internal and/or external comparisons or through the analytical methods chosen. Based on our assessments of RoB and any other validity concerns, an overall appraisal was made and studies of lower quality were noted.

Lower quality studies for which we considered the overall inferential process was problematic included (Birnbaum, 1993; Chhokar et al., 2005; Cooper et al., 1994; Fellner & Sulzer‐Azaroff, 1984; Kuehl et al., 2013; Reddy & Emery, 2001; Saari & Näsänen, 1989; Sossai et al., 2010; Sulzer‐Azaroff et al., 1990; Sulzer‐Azaroff & de Santamaria, 1980; Zafar et al., 1997). While we had concerns about aspects of the Cunningham & Austin study (2007), it was noted that it was presented as a pilot study of an intervention focused on behavior of teams in operating rooms. It is of note that the studies considered to be of lower quality were more likely to have included older work that was published before 2000; 7 of 10 (70%) studies with serial measures published before 2000, but only 5 of 32 (16%) were published from 2000 forward. The lower quality studies were often small and used informal internal comparisons rather than direct statistical adjustment of outcome data.

Studies using serial measures were commonly used in the evaluation of legislative interventions. Blinding of participants and prevention of knowledge of the *legislative interventions* is not possible. Furthermore, based solely on length of the observation periods (four years and up to more than 30 years) all of the studies using serial measures were theoretically at high risk of being influenced by other changes. However, in large part, the investigators had tailored their approach/analyses to compensate for concerns. High risk of maturation effects was assessed for three studies (Derr et al., 2001; Lopez‐Ruiz et al., 2014; Suruda et al., 2002). While both Derr et al. (2001) and Suruda et al. (2002) studies in the US construction industry observed declining fatalities, it is difficult to clearly assign attribution to the legislative effects. Construction injury and fatality rates were declining in general in the study periods, and the investigators made only informal comparison to other fatality patterns in the industry. Lopez‐Ruiz et al. (2014) reported several potential factors that could have influenced traffic injury patterns that would not have been addressed through their use of an internal control with non‐traffic work‐related injuries.

Given the use of the secondary data for retrospective analyses in the ITS studies, concern regarding the intervention affecting outcome data is minimized. It is still possible that concerns about penalties, particularly if they increased after the legislation went into effect, could influence reporting by employers. Little information was available in the study reports about the enforcement of the legislative efforts. No concerns were identified regarding selective outcome reporting (which is hard to identify). No concerns were noted about specification of the point of intervention; investigators who did not use time periods immediately following the intervention explained their approach clearly.

**Figure 7:**
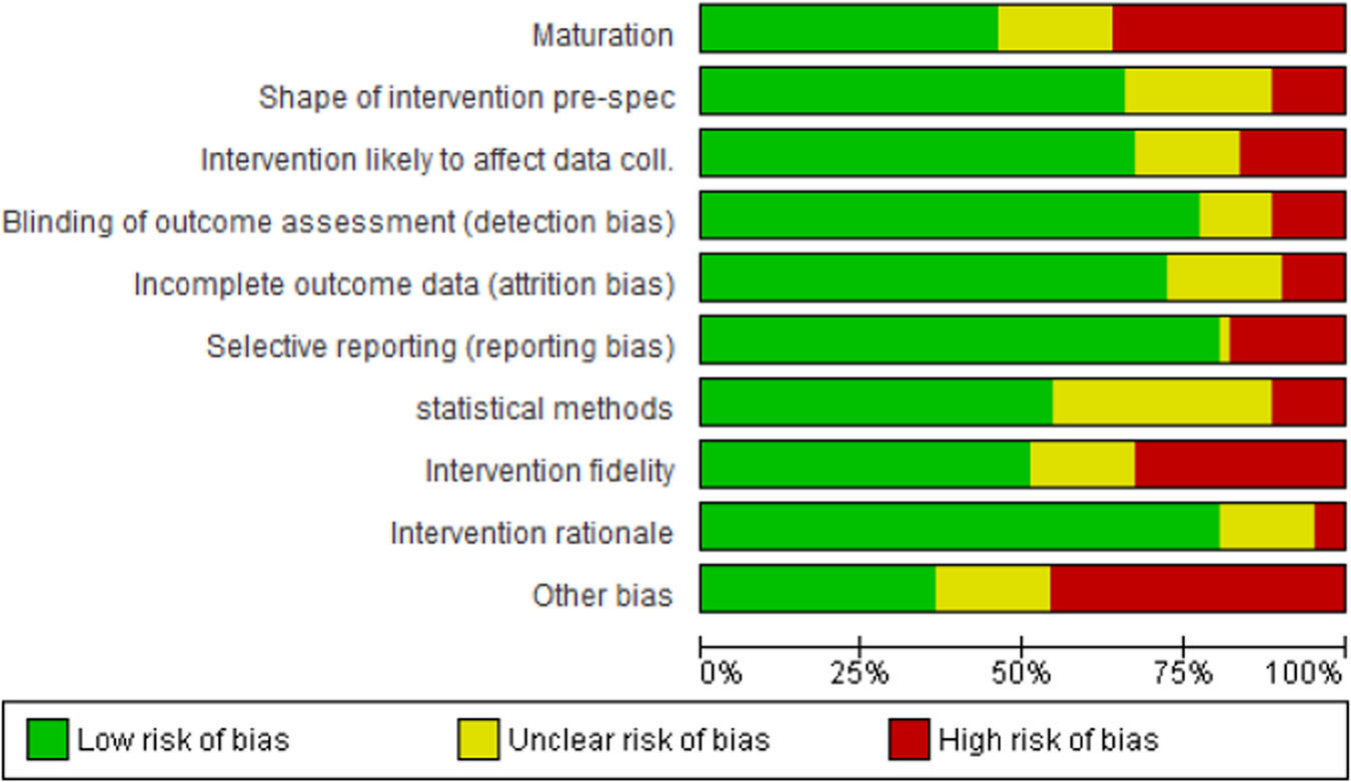
Risk of bias (RoB) for Serial measures (ITS) study designs: RoB graph in percentage for each item across all serial measures, based on review authors' evaluation of each study

**Figure 8:**
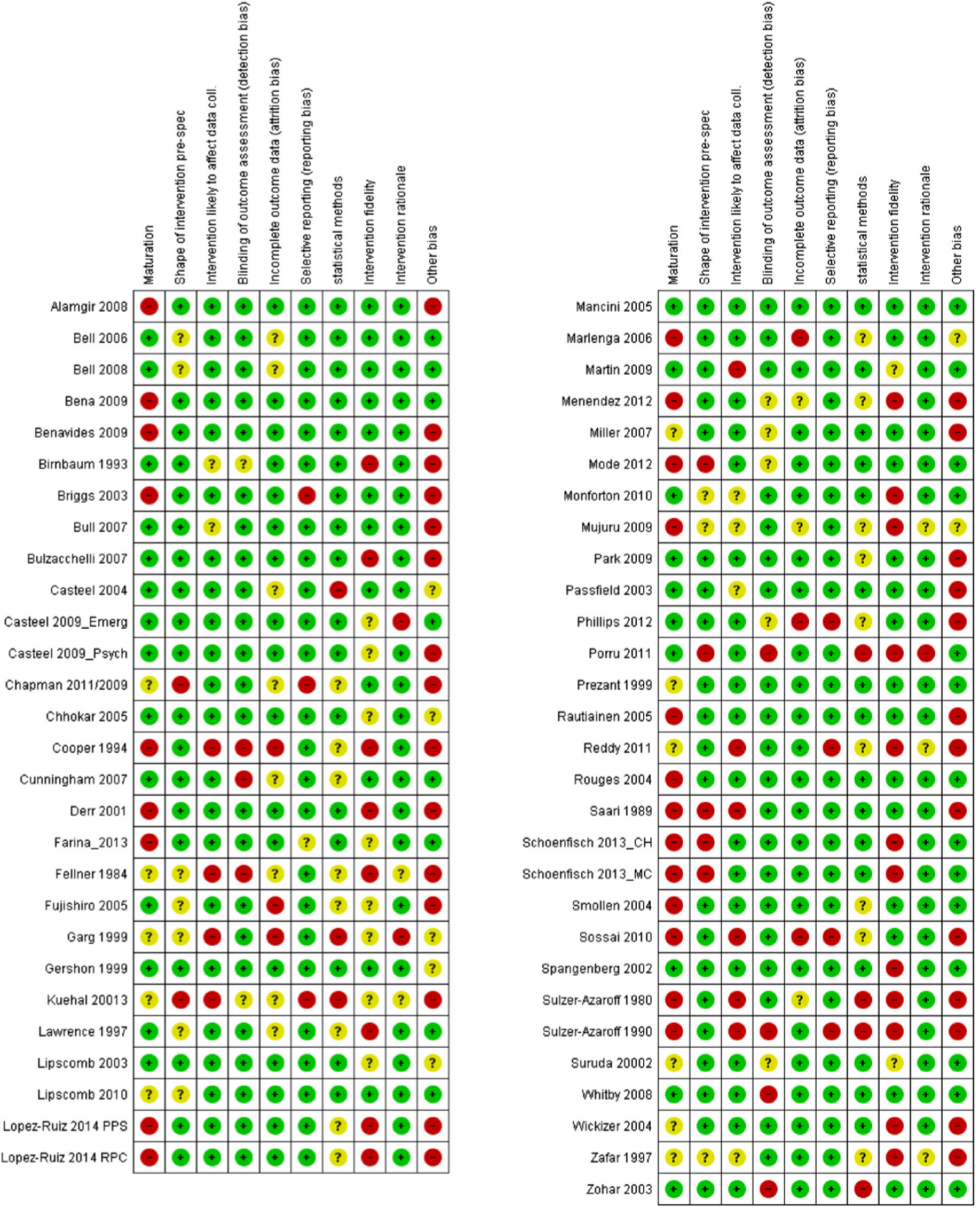
Risk of bias (RoB) for each SERIAL MEASURES (ITS) STUDIES

Aside from these more common concerns regarding bias in ITS studies, several other concerns regarding bias or threats to validity were noted as well (see Figures 7 and 8).

### 5.4 Effects of the interventions

In the following sections each of the six main types of safety interventions are described along with their expected effects, based on the analyses included in the review. In the Supporting Information Appendix (Section 11.1) we have provided Forest plots, and where applicable we have conducted meta‐analyses. In Section 11.1.1 forest plots are provided for RCT and CBA studies, including Comparisons A1–A30, presented for comparisons with injury outcome and/or risk and behavior outcomes, respectively. In Section 11.1.2 Forest plots are provided for serial measures, including Comparisons B1–B17, presented for comparisons with injury outcome and/or risk and behavior outcomes, respectively.

The first of the three main types of safety interventions reported below are directed at the *individual level*.

#### 5.4.1 Safety interventions aimed at modifying attitudes and beliefs

The approaches assessed in this type of safety intervention were primarily concerned with safety campaigns (Forst et al., 2004; Gregersen et al., 1996), group discussions (Adams et al., 2013; Gadomski et al., 2006; Gregersen et al., 1996; van der Molen et al., 2011), and educational approaches (Bena et al., 2009; Johnson & Owoaje, 2012; Mehrdad et al., 2013; Mujuru et al., 2009; Wang et al., 2003).

##### 5.4.1.1 Safety campaigns (Type 1.1.1, Supporting Information Appendix 12.3)

The basic rationale in safety campaigns is that peoples' behaviors are changed through providing them with the right information or knowledge about the hazards in the workplace, and the consequences these can have on their safety at work. This is to be accomplished by use of persuasive messages in posters, pamphlets, videos, or spots on television and the like. We retrieved two studies evaluating safety campaigns, where both studies were using a CBA design. We judged that meta‐analysis was not possible for these safety interventions, as the interventions were different in nature and also the outcome measures used (injuries and behavior).

The studies came from the agriculture (Forst, 2004), and transport and storage (Gregersen et al., 1996) sectors. Safety information in the included studies was provided through various means. In neither of the two studies the campaigns were restricted to media sources, but were more intensive. For example, the campaigns were directed at specific target groups such as drivers in transport and workers on farms and the workers were addressed directly face‐to‐face with safety information.

The agricultural campaign elements were very specific and related to actual work on the farms where the evaluation took place, and evaluated the effect of safety campaigns focused on eye injuries faced by Latino farm workers in southeastern Michigan and northern Illinois (Forst, 2004). Together with visits among Latino workers and provision of campaign materials in English and Spanish, a variety of tinted and clear safety glasses were also provided to the Latino farm workers. The study found an effect in the short term of safety campaigns to change the behavior and risk of the Latino workers with a SMD of −0.62, 95% CI: −1.13, −0.11. However the study had several limitations (see Figure 8, RoB of included RCT and CBA studies) (Comparison A1, chapter 11.1.1.).

In the study among drivers at the Swedish tele‐operator company, “Televerket” (Gregersen et al., 1996), the drivers were targeted at meetings with very specific messages relating to actual work and seasonal problems and road conditions. For example, during the autumn, drivers were informed about driving in darkness, stopping distances and warning. A spring meeting included awareness of other road users, as well as company specific problems such as loading tools and equipment. The safety information was provided by specially trained employees from within the company, during working hours. A number of video films with road safety themes were shown and campaign material and pamphlets were handed out to the drivers. A nonsignificant increase in accidents was reported from this intervention at medium‐term follow‐up (OR: 1.39, 95% CI: 0.96 to −2.01) (Comparison A2, chapter 11.1.1.).

The evidence base was not strong enough to conclude on safety campaigns.

##### 5.4.1.2 Counseling approaches (two‐way approaches) (Type 1.1.2, Supporting Information Appendix 12.3)

We retrieved four safety interventions, three with RCT study designs (Adams, 2013; Gadomski et al., 2006; van der Molen et al., 2011) and one with CBA study design (Gregersen, 1996), that used counseling approaches. The included studies used group discussions (counseling), alone or in combinations, to increase knowledge and changes in attitudes and beliefs. Counseling and group discussions are a two‐way communication process and considered to be an approach with more interaction and involvement than a one‐way educational approach. We conducted a meta‐analysis to evaluate the overall effect of the counseling approach (Comparison A3 and A4, in chapter 11.1.1.).

One RCT study in health wards in hospitals indicated no significant effect of group discussions among health care workers (HCW) (van der Molen et al., 2011). They used a 1‐h interactive Power Point slide presentation beyond usual information given, where participants of the wards were informed, and where information among participants was exchanged about the causes, consequences and prevention of needlestick injuries on their ward. The study showed a non‐significant effect on injuries at short‐term follow‐up (OR: 0.45, 95% CI: 0.15–1.31) of this short intervention effort.

Adams (2013) compared an enhanced interactive education package to a standard education already being provided. The enhanced education consisted of the initial standard education plus education in the form of short street‐plays and messages; group motivational sessions; and individual counseling provided by health workers to those who were not regularly using protective eyewear. These sessions occurred over 1–2 h every week in the 1st month, twice in the second and 3rd month, and so on, and in total 11 educational sessions were conducted. Compared to the van der Molen et al. (2011) study above, the doses provided is much stronger. However, there was no significant effect of this safety intervention at short‐term follow‐up (OR: 0.80, 95% CI: 0.40–1.60).

Another study assessed whether active dissemination and in‐depth communication and counseling on the North American Guidelines for Children's Agricultural Tasks (NAGCAT) reduced childhood agricultural injuries (Gadomski et al., 2006). The intervention also included in‐depth communication between educators and intervention group participants. The core set of guidelines included a chart of recommended ages for tractor operation by size of tractor and task, and guidelines on driving a farm tractor with no implement attached, and several other specific guidelines. Farm visits were conducted and a parent resource booklet containing 52 guidelines was provided to the farm families. Several booster information interventions followed these farm visits. Possible co‐interventions were also assessed and this was taken into account in the analysis. The length of follow‐up was 2 years and 10 months (medium‐term). The study showed a significant effect on injuries (OR: 0.52, 95% CI: 0.29–0.93).

In the Swedish “Televerket study” each driver participated in three meetings of small groups of 8–15 drivers, discussing problems of road safety and what to do about them. Each meeting lasted approximately 1 h. The method used an approach that has been used in Japan in a study of bus drivers (Misumi, 1978, 1982) which showed that accident involvement decreased sharply following group discussions. The intervention arm was compared with a matched control group.

The discussions were led by drivers from their own working unit who had attended a special introduction course. The company had agreed to follow‐up on suggestions from the drivers about measures that should be implemented by the company. There was a significant effect of this intervention at medium‐term follow‐up (OR: 0.53, 95% CI: 0.36–0.77). We had some concern that reporting of accidents may have been influenced by the intervention. There may have been an additional component to the counseling as such, as there seemed to be strong management involvement, and further incentives to the work of the groups as measures would subsequently have been implemented by the company. However, this was not clear from the manuscript, and we decided to classify this intervention as counseling approaches and not multifaceted.

The counseling interventions are homogeneous, though with small effect sizes favoring the intervention. The high level of homogeneity in these interventions may be explained by quite similar interventions focusing on personal protective devices in primarily dynamic work settings. The meta‐analyses showed limited evidence for no effect at short‐term follow‐up (OR: 0.67, 95% CI: 0.38–1.21; *I* ^2^ = 0%). We judged limited evidence for an effect of intensive and interactive counseling approaches including group discussions at medium‐term follow‐up (Meta‐analyses in Comparison A3, in chapter 11.1.1.).

##### 5.4.1.3 Teaching and educational approaches (Type 1.1.3, Supporting Information Appendix 12.3)

Teaching and education, as means for modifying attitudes and beliefs, are common approaches to the prevention of accidents at work. Usually this is a one‐way information approach. Three CBA studies (Johnson & Owoaje, 2012; Mehrdad et al., 2013; Wang, 2003) and two serial measures studies (Bena et al., 2009; Mujuru et al., 2009) were included.

Wang (2003) evaluated the effect of a 60‐min lecture and a 20‐min video on the prevention of needlestick injuries among student nurses in Changsha, China. A non‐significant effect was seen of this intervention (OR: 0.29, 95% CI: 0.07–1.20), after we took the design effect into account. The study had several limitations such as attrition (22 out of 56 students lost to follow‐up), intervention fidelity (intermediate outcome indicate no effect of intervention), and that behavioral intervention might have influenced self‐reported injuries to the teacher. In another study by Mehrdad et al. (2013) in Iran the focus was also the effect of teaching and educational activities on needlestick injuries, showing a non‐significant increase in injuries in the intervention group (OR: 1.08, 95% CI: 0.77–1.51). Serious RoB was also judged for this study (Figure 6), including lack of intervention fidelity and detection bias, and we also considered that the intervention was likely to have influenced reporting of injuries.

The third CBA study evaluating teaching and educational approaches (Johnson & Owoaje, 2012) assessed the effect of health education on the riding habits of commercial motorcyclists in southern Nigeria. The motorcyclists in the intervention group received safety education with health and safety lectures and interactive sessions, and the controls had no intervention (placebo treatment, received HIV health education). No significant effect of the intervention was observed (OR: 0.93, 95% CI: 0.46–1.85) (See Comparison A5 for the three CBA studies in chapter 11.1.1.).

These studies of teaching and education safety interventions are moderately homogeneous, though with no effect at short‐term follow up. The diverse settings (Iran, China, and Nigeria), variations in the work context (health establishments and mail delivery by means of motorcycle), and one study with alternative control group, are likely to be sources of heterogeneity. We conducted a meta‐analysis for the effect of these three teaching and education safety interventions at short‐term follow up that showed no significant effect (OR: 0.90, 95% CI: 0.56–1.46; *I* ^2^ = 37%) (Comparison A5 in chapter 11.1.1.).

In the safety interventions using serial measures, there were observed minimal (Bena et al., 2009) or no impact (Mujuru et al., 2009), in the absence of other intervention efforts, for safety teaching and educational approaches (Comparison B1, chapter 11.1.2.). One study in the construction trades aimed to decrease overall injury rates through education (Bena et al., 2009); only 29% of the workforce were able to be recruited to participate on this large Italian construction project. By use of a time‐series regression model to assess the effect at medium‐term follow‐up, the study demonstrated a very modest and non‐significant effect. Mujuru et al. (2009) evaluated the effect at long‐term follow‐up (8 year) of a single session that included a 14‐min video focused on prevention of causes of fatalities in logging (US), specifically struck by injuries and caught in, under or between events. No significant effects were seen in these studies.

These latter two safety interventions using serial measures represent different intensities and doses of training. However, the analyses showed limited evidence for no effect of the safety interventions, and thus supported the meta‐analysis of the controlled studies. We conclude that there is limited evidence for no effect of teaching and educational approaches, without any other simultaneous intervention efforts (Comparison B1 in chapter 11.1.2.).

##### 5.4.1.4 Summary of the effect of attitude and beliefs modification

No or modest effects were found across the various means directed at attitude and belief modification as an approach to reducing injuries or improve safety and behavior. Thus, it is somewhat uncertain whether attitude and belief modification as a safety intervention approach, by itself, has any effect on work injuries. There seems to be limited evidence for no effect of the KAP model, as a one‐way communication strategy in a workplace setting.

There could be seen some promise in the group discussions, as the nature of this approach may represent stronger interaction and involvement than one‐way educational approaches or campaigns. Future work should distinguish between campaigns disseminated through media and other channels of influence, and attitude modifications that take place “face‐to‐face” in group discussions; the latter approach among Swedish telephone installers was the only study that showed a significant positive effect on the number of work‐related road traffic accidents at medium term follow‐up.

We conclude that the link between attitude modification and accidents seems to be uncertain, and that the level of evidence is limited for no effect of one‐way communications in preventing accidents. However, attitude shaping as a one‐way approach may be relevant in the context of other types of safety interventions, and will also be present as components in the multifaceted safety interventions analyzed below. Counseling approaches representing two‐way communication showed limited evidence for a strong effect at medium‐term follow‐up, but no effect at short‐term follow‐up.

#### 5.4.2 Safety interventions aimed at behavioral modifications (Main type 1.2.0., Supporting Information Appendix 12.3)

The behavioral approaches in our material primarily include safety training (Type 1.2.2), where results are presented in Comparisons A6–A7, and individual goal setting and feedback (Type 1.2.4), presented in Comparisons A8–A10, in chapter 11.1.1. We included three RCT studies (Cheng & Chan, 2009; Daltroy, 1999; Jinnah et al., 2014) and three CBA studies (Banco et al., 1997; Gregersen, 1996; Quintana, 1999). The study by Jinnah et al. (2014) included two intervention arms where either a parent or a member of staff provided individual safety feedback to the youths.

##### 5.4.2.1 Safety training

A study to reduce cut injuries among young and inexperienced workers (Banco et al., 1997) used training in use of case‐cutters. Each employee attended a 15‐min training session specific for the type of cutter being used over a 4‐week period. The content of the training sessions included instruction and practice of opening and closing of case cutters, changing blades, top, tray‐pack, and mid‐cut cutting techniques, blade disposal, cutter storage, and re‐supply of case cutters and blades. The study demonstrated a modest (small) non‐significant change in reducing injuries (OR: 0.91, 95% CI: 0.39–2.12) (Comparison A6, in chapter 11.1.1.).

Gregersen's (1996) study on Swedish telephone repair workers showed an effect at medium‐term follow‐up (2 years) of training on the reduction of accidents (OR: 0.60, 95% CI: 0.43–0.83). The training was comprised of three blocks: maneuvering training, skid training and “commentary driving.” Apart from the skid training, where a skid simulation car was used, the training was carried out in real traffic. Each of the blocks lasted 2 h and 30 min, and thus the whole training program lasted nearly 8 h. A special driver training team from the National Society for Road Safety provided the instructors for all the drivers (Comparison A7, in chapter 11.1.1.).

##### 5.4.2.2 Individual goal setting and feedback/coaching

A study (Cheng & Chan, 2009) of the effect of individual job coaching and use of a job‐specific occupational health education program (protection motivation theory), on prevention of work‐related musculoskeletal back injury among construction workers, did not give any significant effect (OR: 0.37, 95% CI: 0.09–1.56). This study also had several shortcomings in the design and statistical analyses, and is thus of low quality (Comparison A8, in chapter 11.1.1.).

Jinnah et al. (2014) reported on interventions to improve tractor safety as part of a larger youth‐on‐farm safety intervention, where efforts focused on knowledge transfer, active learning, and skill building. A 16‐item survey was used to measure farm safety behavior of fathers and youth (mainly to prevent tractor overturns), guided by the TPB(Ajzen, 2012). Youth 10–19 years of age were randomly assigned to either one of two intervention groups (parent‐led or staff‐led), or a control group; Training and information surrounding tractor safety including use of Roll‐Over Protection Structure (ROPS) were provided to the farms, and either staff (intervention arm one) or fathers (intervention arm two) provided individual feedback to the youths.

We evaluated the effect of the interventions by measuring the SMD of youth's unsafe behaviors for the intervention farms compared to the control farms (Comparison A8, for outcome 8.2: risk and behavior, in chapter 11.1.1.). For the parent‐lead intervention there was a significant effect (SMD: −0.60, 95% CI: −0.12, −1.08), and a non‐significant effect for the staff lead intervention (SMD: −0.41, 95% CI: −0.85, 0.04).

A controlled trial of a training program, equal to the so‐called “back schools,” among 4000 US postal workers, was evaluated to see whether it reduced injuries (Daltroy, 1999). This behavior based intervention included education through classroom sessions, safety training and individual safety coaching, and did not provide any significant effect (OR: 1.11, 95% CI: 0.90, 1.37) (Comparison A9, in chapter 11.1.1.).

Quintana (1999) evaluated the effect of task‐delineated safety (TDS)—a BBS management scheme—on slip, trip, and fall (STF) accidents. The outcome was the number of hazards observed which may induce in a STF accidental injury. The TDS approach combined behavior based principles of clear task delineation, individual accountability, and continuous performance feedback. They reported a reduced risk of hazards, but it was not possible to estimate a point estimate on the basis of the data provided (Comparison A10, in chapter 11.1.1.).

##### 5.4.2.3 Summary of the effect of behavioral modifications (Type 1.2.0., Supporting Information Appendix 12.3)

We judged that the included behavioral safety interventions were too diverse in types of interventions and settings to allow for meta‐analytic estimates. The effects of the behavioral safety interventions point in different directions, where interventions both favor control and intervention.

Neither of the two RCT studies aiming to reduce low back injuries related to lifting and handling of goods in construction and in postal service work provided any significant effect of the behavioral approach on injuries. The four CBA studies pointed in different directions. The one study arm in Gregersen (1996) using training of drivers had an effect on road traffic accidents, whereas the Banco et al. (1997) study among young and inexperienced workers did not reduce injuries.

Apart from Gregersen's (1996) study of drivers, the statistical power is not strong in the safety interventions evaluating safety training. The level of evidence is limited for no effect at short‐term follow‐up and moderate effect at medium‐term follow‐up. We conclude that the included studies indicate limited evidence for an effect of safety training. The level of evidence is moderate for little or no effect of individual feedback or coaching at medium‐term follow up, whereas there is limited evidence for no effect at long‐term follow‐up.

#### 5.4.3 Safety interventions aimed at physiological modifications (Type 1.3.0, Supporting Information Appendix 12.3)

We identified four studies evaluating nine safety interventions aimed at physiological modifications, that is, human physiology is modified through various training methods, which can reduce the risk of injury, either through improved endurance, higher strength, improved flexibility of joints, reduced body weight, or a combination of these approaches. One study used an RCT design (Morgan, 2012), two studies used a CBA design (Hilyer et al., 1990; Knapik et al., 2003), and one used serial measures (Kuehl et al., 2013) to evaluate the long term effect of physiological modification.

Safety interventions aimed at physiological changes can be carried out through changes in physiology, such as training of the working population in scope, or weight loss, to increase an individual's resistance to physiological loads.

Morgan et al. (2012) assessed effectiveness of weight loss as a way to increase resistance to accidents, based on the concept that personal fitness can decrease injury. The workplace‐based weight loss program (Workplace POWER) for male shift workers reported decreased accidents at short‐term follow‐up. However, overall the Workplace POWER weight loss program did not provide a clear picture of an intervention effect. The relationship between weight loss and accidents was not clear from the manuscript, and no theoretical basis was provided. The theoretical link to weight loss of the magnitude observed (mean weight loss was 4.4 kg compared to treatment), creating a change in injury rates of nearly 60%, was very weak. Weight measures were assessed at 14 weeks of follow‐up, while injury outcomes were assessed at 12 months. This was problematic, as no documentation was provided that weight loss was maintained in the 1 year follow‐up period. The level of evidence supporting weight loss as an injury prevention intervention was therefore judged to be insufficient and was not included in the meta‐analysis.

One study (Knapik et al., 2003) examined injury outcomes in Basic Combat Training during implementation of PRT among male and female army recruits. PRT differs from usual training practices in that it de‐emphasizes running, and incorporates procedures and principles designed to reduce injuries and increase functional fitness. We considered the study at high RoB given that the control group had more than double the number of running miles compared to the intervention group; running can increase the risk of traumatic injuries and overuse injuries. The study found no significant effect for male recruits (OR: 0.97, 95% CI: 0.58–1.62) or for female recruits (OR: 1.09, 95% CI: 0.71–1.67). The meta‐analytic effect of individual PRT at short‐term follow‐up was none (OR: 1.04, 95% CI: 0.75–1.44; *I* ^2^ = 0%) (Analysis A11 in chapter 13.1). The two meta‐analyzed CBA studies were in similar setting (military) and by same authors, which might explain that the interventions are highly homogeneous (Comparison A11, in chapter 11.1.1.).

Hilyer et al. (1990) evaluated the medium‐term effect of a flexibility intervention to reduce the incidence of joint injuries among municipal firefighters in the southeast US. The basic idea was to evaluate whether better fitness would protect against injuries in the firefighters' work. A non‐significant 25% reduction in injuries was seen (OR: 0.75, 95% CI: 0.48–1.18) (Comparison A12, in chapter 11.1.1.).

Kuehl et al. (2013; PHLAME study) assessed the long‐term effect (5‐year follow‐up) of a program focused on nutrition and physical activity using serial measures. Although, the exact intervention was not clear from the paper, we interpreted it as being mainly physical activities. The authors reported an immediate change in reported injuries, which they attributed to the intervention. Given lack of a physiological basis, the conclusion seems spurious. It was not possible to calculate an exact point estimate based on the provided information, but the graph provided indicated an effect of the intervention that does not appear to be justified in the report (Comparison B2, in chapter 11.1.2.).

##### 5.4.3.1 Summary of the effect of safety interventions aimed at physiological modifications (Type 1.3.0., Supporting Information Appendix 12.3)

Overall, we judged the level of evidence to be limited for safety interventions targeting strength and endurance of human physiology, and that these safety interventions tend to have no effect on reducing injuries at work. While one RCT study (Morgan, 2012) reported a positive effect of personal fitness in the form of weight loss, we judged the evidence of the relationship between weight loss and injuries to be unfounded. Further work in this area should explicitly define the mechanisms through which such interventions are intended to work, and when the effects may be expected to be seen, for which types of accidents.

The first of the three main types of safety interventions reported above, were directed at the individual level, whereas the following approaches, are directed at the group or organizational level.

#### 5.4.4 Safety interventions aimed at climate, norms and cultural changes (Type 2.1.0., Supporting Information Appendix 12.3)

Zohar (1980) developed the first theoretical and practical approach to the concept of safety climate. The included studies in this area lasted from 4 months to those with a 3 years follow‐up. We retrieved one RCT (Zohar, 2002), three CBA studies (Kines et al., 2010; Mattila & Hyoedynamaa, 1988; Ray et al., 1997) and six studies using serial measures (Cooper et al., 1994; Cunningham & Austin, 2007; Fellner & Sulzer‐Azaroff, 1984; Sulzer‐Azaroff et al., 1990; Sulzer‐Azaroff & de Santamaria, 1980; Zohar, 2003). Primary data collection of numerous measurements obtained through observation of workers was used in all of these studies (Comparisons A13–A14, in chapter 11.1.1. and Comparisons B3–B4 in chapter 11.1.2.). No studies on modification of safety culture were included.

A subset of these studies (Kines et al., 2013; Zohar, 2002, 2003) used leadership based safety interventions that allow for the modification of all subordinate safety behaviors, as antecedents and consequences are based on continual supervisory monitoring in ever‐changing situations (Type 2.1.7., Supporting Information Appendix 12.3), and thus impact the safety climate of a work unit. Zohar (2002) investigated the effect of a leadership‐based intervention model to modify supervisory practices to improve subunit safety and safety climate, and thus decrease “micro‐accidents” in an industrial plant (i.e., minor injuries incurred due to unsafe behavior, suffered during work activities, and of sufficient severity to discount the possibility of an unjustified visit to the infirmary). Participants were assigned quasi‐randomly to intervention and control (about 190 line workers and 18 supervisors in each group). The study showed a significant effect on the rate of “micro‐accidents,” that is, less serious injuries, (OR: 0.25, 95% CI: 0.11–0.58) (Comparison A14, in chapter 11.1.1.). This result was supported by improved safety climate (repeated measures of variance for climate scores) and improved behavior (increased rate of safe behavior in organizational subunits).

Another study by Zohar (2003) using serial measures reported evidence of increased safety behaviors and safety climate scores through safety feedback to work groups, leaders and workplaces (intervention type 2.1.5) in a follow‐up period of 4 months. Supervisory safety‐oriented interactions increased and safety climate scores improved, resulting in improved worker safety behavior. We could not establish effect sizes on the basis of the provided correlation measures (Comparison B3, in chapter 11.1.2.).

The studies of Kines et al. (2010, 2013) aimed to improve worksite safety through the use of goal setting, safety coaching and feedback to work groups, upper management and leaders. In the study of Kines et al. (2010) construction foremen in two intervention groups were given bi‐weekly feedback about their daily verbal safety communications with their workers during a 26‐week intervention period. A safety climate measure was used to assess as the outcome measure, but only one aspect of the climate measure improved in only one intervention group (“attention to safety”). At follow‐up (from 8 to 16 weeks) the intervention favored the control group (SMD: 0.05, 95% CI: 0.02, 0.08).

In the other study by Kines et al. (2013) four owner/manager lead dialogue meetings with workers and coaching sessions with leaders were provided at six intervention metal industry sites during a 16‐week intervention period. Compared to the previously mentioned study by Kines et al. (2010), more emphasis was given to involve upper management and leaders in a problem solving and culture change process, based on Dejoy's (2005) integrated safety management approach. An increase was observed in six of eight safety‐perception‐survey factors in the intervention sites, however, seven of the factors also increased in the control group. The mean safety index improved at the six intervention sites compared to the eight control sites, although not significantly at 17–26 weeks follow‐up (SMD: −0.32, 95% CI: −0.70, 0.07) (Comparison A14, outcome 14.2: risk and behavior, in chapter 11.1.1.).

We did not conduct meta‐analysis for the leadership based climate interventions, as the heterogeneity was high, and we judged that this was due to the diversity in their approaches to changing safety climate, and differences in outcome measures. We judged limited evidence that leadership based safety interventions have little to moderate effects.

The following studies used classic goal setting and feedback methods directed at group level (type 2.1.1). Mattila and Hyoedynamaa (1988) used group discussions for the goal setting and feedback mechanisms, and with duration of intervention between 20 and 22 weeks. Targets for safe handling and conditions at the construction site were discussed and the target was posted at various places at the workplace. Weekly inspections evaluated the targets and assessed whether they were attained. A safety index was created, and feedback on how the targets were attained was given to the site and to the construction workers. We recalculated the OR based on the data provided in the paper, and could not establish a reduction in accidents rates compared to the control site, as the paper indicated, even there was a slightly decrease of severity of injuries at the intervention site. The safety index measure increased over time (20 weeks follow‐up), and a non‐significant increase in accidents was observed (OR: 1.13 95% CI: 0.23–5.57) (Comparison A13, in chapter 11.1.1.).

Ray et al. (1997) also investigated the effect of goal‐setting and performance feedback by using a safety index. They reported that goal setting in addition to feedback can enhance the beneficial impact of a BBS program in the workplace. However, by comparing the mean safety index of the intervention to the control group, we found only a small and non‐significant effect of the intervention (SMD: −0.03, 95% CI: −0.13, 0.07) (Comparison A13, outcome 13.2: risk and behavior, in chapter 11.1.1.).

Some other studies were of lower quality, with little information provided about the fidelity of the interventions, and lack of intermediate variables and sound statistical approaches, and we assessed the overall inferential process to be problematic (Cooper et al., 1994; Cunningham & Austin, 2007; Fellner & Sulzer‐Azaroff, 1984; Sulzer‐Azaroff et al., 1990; Sulzer‐Azaroff & de Santamaria, 1980) (Comparisons B4, in chapter 11.1.2.). It is of note that the lower quality studies more often included older work that was published before 2000. Some of these studies are among the first attempts to assess goal setting and feedback interventions, and they provide some useful arguments for this approach.

##### 5.4.4.1 Summary of the effect of climate, norms and culture (Type 2.1.0., Supporting Information Appendix 12.3)

There are a few high‐quality studies in this group of safety interventions, which indicate an effect on behavior and accidents. However, taken as a whole, the effect pattern in the included studies is inconsistent, and in particular there is no clear demonstration of the relationship between the intermediary measures (climate scores, safety index scores, and behavior), and reduction in injuries in all of the studies. Of those studies we considered higher quality, only Zohar (2002) used occupational micro‐accidents as outcome (minor accidents—see above). We conclude that there is limited evidence that leadership based safety interventions have from little to moderate effect.

There was limited evidence that safety interventions using classic goal setting and feedback methods directed at group level have no effect at short term follow‐up.

#### 5.4.5 Structural modification (Main type 2.2.0., Supporting Information Appendix 12.3)

Structural modifications comprise several models and components related to changes in the physical, organizational, or regulatory environment. Some of these changes are initiated from external bodies and some other internal processes and decisions by organizations, or a combination of these. Organizational or regulatory environment can be modified by introducing legislation, enforcement, legal sanctions of rules and regulations, or economic incentives, or by changes in the administrative routines or changes in machinery and engineering solutions in companies.

We retrieved eight studies evaluating the introduction of legislation, and 14 studies evaluating the introduction of enforcement and compliance approaches. We also identified some studies evaluating effects of voluntary social controls: Two studies evaluating economic incentives; three studies evaluating internal administrative controls in companies; one study evaluating soft regulation; and one study using serial measures to evaluate the effect of social marketing.

Finally, we retrieved 19 studies that evaluated engineering controls, such as, introduction of machine safeguards, walkways, elimination of hazardous substances or materials, and other changes in the physical environment.

##### 5.4.5.1 Legislative changes and enforcement (Type 2.2.1, Supporting Information Appendix 12.3)

Structural interventions such as regulation and enforcement may serve as a potentially powerful institutional force to promote the adoption of occupational health and safety policies and practices. The basic idea is that changes in laws and regulations provide coercive power or incentives for people or organizations to change behavior, and stick to certain (legal) standards that can impose effects on large working populations, as they are compulsory.

Fifteen studies were identified that sought to evaluate the effects of legislative intervention on accident at work through regulation and/or enforcement activities. The majority were from the US; (Bulzacchelli et al., 2007; Casteel et al., 2009; Derr et al., 2001; Foley et al., 2012; Haviland et al., 2012; Levine et al., 2012; Lipscomb et al., 2003; Marlenga, 2006; Monforton & Windsor, 2010; Phillips, 2012; Suruda et al., 2002) two were from Spain (Benavides et al., 2009; Lopez‐Ruiz et al., 2014); and there were single studies from Italy (Farina et al., 2013) and Canada (Hogg‐Johnson et al., 2012).

All but four of the studies were classified as serial measures (ITS) (Benavides et al., 2009; Bulzacchelli et al., 2007; Casteel et al., 2009; Derr et al., 2001; Farina et al., 2013; Foley et al., 2012; Lipscomb et al., 2003; Lopez‐Ruiz et al., 2014; Marlenga, 2006; Monforton & Windsor, 2010; Phillips, 2012; Suruda et al., 2002). One involved a CBA study design (Haviland et al., 2012), one was a simulated RCT (Levine et al., 2012), and one was an RCT (Hogg‐Johnson et al., 2012). All of the studies that focused on legislative efforts, or rule making, used ITS study designs; while three of the five studies exploring effects of activities designed to increase compliance with existing regulations such as consultation, inspections or enforcement—included the more experimental designs (Haviland et al., 2012; Hogg‐Johnson et al., 2012; Levine et al., 2012).

Investigators typically provided important contextual detail regarding the intervention that was used in guiding the analytical approach and/or in the interpretation of findings (Table 14, structural safety interventions).

In the following, the effects of legislation and the effects of enforcement/compliance efforts will be treated separately.

###### 5.4.5.1.1 Effects of legislation

All legislative safety interventions used serial measures (ITS) studies. Eight studies representing nine safety interventions focused on evaluating effects of specific pieces of legislation designed to reduce workplace injury morbidity and/or mortality (Bulzacchelli et al., 2007; Casteel et al., 2009; Derr et al., 2001; Lipscomb et al., 2003; Marlenga, 2006; Monforton & Windsor, 2010; Phillips, 2012; Suruda et al., 2002). The legislative intervention evaluations targeted overall injuries through mandated training requirement in surface mining (Monforton & Windsor, 2010); mortality and morbidity from falls from heights (Derr et al., 2001; Lipscomb et al., 2003); fatalities from trenching accidents in construction (Suruda et al., 2002); fatalities from hazardous energy (lockout/tagout) in manufacturing and construction (Bulzacchelli et al., 2007); violence and needlestick injuries in healthcare (Casteel et al., 2009; Phillips, 2012); and farm tractor‐related accidents on roadways among teens (Marlenga, 2006). All of these were in the US and involved longer‐term evaluation; observation time ranged from 6 to 21 years (mean 11.1 years, median 11 years) with follow‐up periods after the intervention from 4 to 11 years (mean 6.3 years, median 6 years).

In the studies evaluating legislative efforts, not all effects could be attributed to the interventions. Derr et al. (2001), Suruda et al. (2002), Bulzacchelli et al. (2007) all reported some decrease in fatality rates, but attribution could not be made to the interventions. Inferences on Derr et al. (2001) (not estimable) and Suruda et al. (2002) (OR: 0.55, 95% CI: 0.46–0.67) were limited by their informal comparisons to other construction deaths, while Bulzacchelli et al. (2007) adjusted her analyses leading to non‐significant effects (OR: 1.05, 95% CI: 0.87–1.27). The authors explain that the lack of a decline in the rate of machinery‐related fatalities in manufacturing after the standard took effect, may be due to a low level of compliance with the standard (Comparison B5, outcome 5.1: injuries, in chapter 11.1.2.).

Casteel et al. (2009), Lipscomb et al. (2003), and Phillips (2012) reported patterns of injury decline consistent with the legislation. Casteel found a significant effect in emergency departments (OR: 0.52, 95% CI: 0.31–0.87), but no significant effect on psychiatric units (OR: 0.63, 95% CI: 0.26–1.53). Phillips' analyses and results are the most clear‐cut, demonstrating a significant reduction (OR: 0.68, 95% CI: 0.66–0.70). The needlestick problem was also the most discrete, and the legislation mandated an engineering solution. Lipscomb's analyses were limited by a short “pre‐intervention” period, requiring overall inferences to be made from contrasts to non‐fall injuries over time, and indicated an effect, although not significant.

Monforton & Windsor (2010) found a decreased level of permanently disabling injuries (OR: 0.59, 95% CI: 0.53–0.66). However, inconsistency in the results with the other injury‐severity categories precluded attributing the observed outcome to the US Department of Labor's Mine Safety and Health Administration (MSHA) regulation, which was not consistent with an intervention effect. The evidence‐base for the intervention was also questionable.

Marlenga (2006) found no significant effect on tractor events on roadways among farm youth (OR: 0.58, 95% CI: 0.21–1.60). The methods are not strong (no denominator data and proportional analyses), and the legislation that mandated an educational approach that has been deemed an ineffective approach for promoting farm safety, lacked any evidence‐base by the time it was passed.

###### 5.4.5.1.2 Summary of the effect of legislation

The analyses of the safety interventions helped identify patterns of effectiveness over time that are important in understanding the process through which regulations may work (Casteel et al., 2009; Lipscomb et al., 2003). We also find evidence that in some cases regulation does not reduce accidents, as the preventive component in the legislative approach has no effect. Monforton and Windsor (2010) work provides quite convincing arguments that legislative requirements for training alone in surface mining did not create a meaningful reduction in injuries. Although a causal relationship between the regulatory intervention and the decline in the rate of permanently disabling injuries is plausible, inconsistency in the results with the other injury‐severity categories, preclude attribution of the observed outcome to the US Department of Labor's Mine Safety and Health Administration (MSHA) regulation.

The Marlenga's (2006) study of tractor incidents on roadways among youth and the Monforton & Windsor (2012) study on training requirements for surface miners, to some degree, demonstrate legislation passed with little evidence‐base, and for which, enforcement would be unlikely to substantively affect injury outcomes.

Attribution of declining fatalities to legislation is not consistently demonstrated in these studies. Bulzacchelli (2007), Derr et al. (2001) and Suruda et al. (2002) all demonstrated declining fatalities, but the findings do not clearly allow attribution to the standard in question. Methodological constraints and history surrounding these legislative efforts may contribute to the inability to identify effects. The long and evolving rule‐making processes surrounding both the revision of trenching and lockout/tagout regulations, demonstrate that it may be difficult to establish a well‐defined intervention period (Bulzacchelli et al., 2007). These fatality studies also have less statistical power due to the relatively rare incidence of death, even when looking at all deaths in the US over fairly long periods of time.

Overall, from this review process we judge that there is limited evidence that regulation has from little to moderate effect on the prevention of accidents at work.

##### 5.4.5.2 Effects of enforcement (Type 2.2.7, Supporting Information Appendix 12.3)

Seven studies, representing 12 interventions, assessed effects of inspections and enforcement of laws and regulations (Benavides et al., 2009; Farina et al., 2013; Foley et al., 2012; Haviland et al., 2012; Hogg‐Johnson et al., 2012; Levine et al., 2012; Lopez‐Ruiz et al., 2014). Four of the six studies assessing enforcement efforts demonstrated declining patterns of injury rates consistent with the enforcement efforts. Further information on the studies can be seen in Table 14 on *the nature of included Studies Evaluating structural modifications, under the subheading 2.2.1 legislative change and 2.2.7 enforcement of laws and regulations*. Funnel plots for study effects can be seen in chapter 11.1.1. (Comparisons: A15–A18).

Enforcement efforts targeted overall and serious injuries in construction in Italy (Farina et al., 2013); overall reportable injuries in manufacturing in the US state of Pennsylvania and Ontario, Canada (Haviland et al., 2012; Hogg‐Johnson et al., 2012); and injuries in mixed industries in the states of Washington and Pennsylvania in the US (Foley et al., 2012; Levine et al., 2012). The observation period for these studies ranged from 21 months to 12 years (mean 7.8 years, median 10 years) with follow‐up periods after the intervention from 21 months to 6 years (mean 4.2 years, median 4 years).

The Hogg‐Johnson study (2012), the only RCT, reported no significant effects of enforcement (OR: 0.99, 95% CI: 0.90– 1.09) for non‐fatal injuries compared to no intervention (Comparisons: A16, in chapter 11.1.1.).

The Levine study (2012), a simulated RCT (study design classified as CBA), compared inspections to no intervention for all types of injuries at short‐time follow‐up (OR: 0.86, 95% CI: 0.77–0.95) and long‐term follow‐up (but data not included because we could not rule out risk of repeated measures). The Levine study did not find any significant effect at medium term follow‐up (but data not included, for same reason as above). For the Haviland et al. (2012) study we took the sum of year one and two for complaint inspections (based on workers' complaints about a hazard) with or without penalty, to compare with programmed inspections (based on industry and firm hazardousness), and to have the same time points for the follow‐up (Comparisons: A16, in chapter 11.1.1.).

We calculated the standard error from the *P* values given in the article according to the methods described in the Cochrane Handbook for Systematic Reviews of Interventions (Higgins & Green, 2008). In addition, Haviland et al. (2012) study did not find significant effects of complaint inspections at medium‐time follow‐up, with citations or penalties imposed compared to no inspections/penalties (OR: 0.99, 95% CI: 0.89–1.05). However, the programmed inspections showed a modest effect at medium‐time follow‐up, with citations or penalties imposed compared to no inspections/penalties (OR: 0.95, 95% CI: 0.91–0.98). Businesses without inspections served as the control, and they excluded multi‐site organizations to ensure linkage between facility, intervention, and injury, and thus strengthened the internal validity.

Foley et al. (2012) incorporated a mix of internal and external comparisons and stratified analyses that facilitated interpretation. Foley compared companies (accounts) that had enforcement activities and companies that had consultation activities, respectively, to companies without any consultation or enforcement. Foley found that inspections with or without citations were more effective than consultations, although with no or little effect. This was the case for both fixed‐site industries (OR: 0.96, 95% CI: 0.92–0.99), such as manufacturing, and non‐fixed site industries (OR: 0.97, 95% CI: 0.95–0.99), such as construction work. However, Foley did not find any significant effect of consultations, either on fixed‐site industries (OR: 0.97, 95% CI: 0.93–1.01) or on non‐fixed sites (OR: 0.92, 95% CI: 0.85–1.00) (Comparisons: A17, in chapter 11.1.1.).

Three ITS studies were included (with eight study arms in total) (Comparison: B6); Farina et al. (2013) report was based on external comparisons for which greater detail would have been helpful. Farina et al. (2013) investigated the formalized programs to implement minimum health and safety requirements in construction work, comprising inspection, training and information plans, and found a modest effect (RR: 0.81, 95% CI: 0.67–0.99).

Benavides et al. (2009) reported decreasing injury rates but the declines could not be attributed to the “Preferential Action Plans” (PAP) that focused on regional manufacturing and private industry. Lopez‐Ruiz et al. (2014) reported mixed outcomes of the effectiveness of the “Penal Point System” and “Reformed Spanish penal code” initiatives on traffic‐related occupational injuries. It was also difficult in this study to attribute observed patterns to the roadway safety interventions, and furthermore, the two interventions were treated as if they were independent, even though they overlapped in time. It may be that company internal policies and practices are more important in managing employee working conditions, thereby reducing road traffic accidents, as was seen in the study by Gregersen (1996) (Comparisons: A4, in chapter 11.1.1.).

###### 5.4.5.2.1 Summary of the effect of enforcement and compliance

In contrast to legislative effects, the evidence that enforcement activities can work was rather consistent (Farina et al., 2013; Foley et al., 2012; Haviland et al., 2012; Levine et al., 2012) but with lower effect sizes, overall. The Canadian study, the only straightforward RCT in this group (Hogg‐Johnson et al., 2012), did not demonstrate any significant effect of enforcement or consultation activities as delivered in the province of Ontario. Though a strong experimental study, it was not without potential error that may have biased results toward the null.

The meta‐analysis shows that there was moderate evidence for no or little effects of enforcement at medium‐term follow‐up (OR: 0.93, 95% CI: 0.93–0.97; *I* ^2^ = 0%) (Analysis A17 in chapter 13.1), whereas there was limited evidence of a little effect at short‐term (OR: 0.86, 95% CI: 0.77–0.95; *I* ^*2*^ = not appl.) and long‐term follow‐up (OR: 0.77, 95% CI: 0.64–0.92; *I* ^2^ = 51%) (Comparison: A15, A16, and A18). The four interventions using serial measurers indicated a moderate evidence for no or little effect of enforcement at long‐term follow‐up (Comparison: B6, chapter 11.1.2.).

Theoretically, legislation may have effects without enforcement, for example, if changes are made in anticipation of possible penalties. It seems these effects may be short‐lived. However, it seems reasonable that longer‐term effects would be enhanced by enforcement, but this is only true if the preventive regulations being enforced actually prevent injuries. The legislative rule‐making process often involves negotiation and compromises, such that a promulgated standard in varying degrees will consider both the epidemiologic evidence and political decision‐making processes.

##### 5.4.5.3 Effects of economic incentives (Type 2.2.2, Supporting Information Appendix 12.3)

We retrieved one controlled before and after study (Gregersen, 1996), and one with serial measures (Rautiainen et al., 2005). Marketing and economic incentives can be seen as a voluntary exchange, for example, insurance related benefits for low risk companies (Rautiainen et al., 2005), or economic incentives for group level behavior (Gregersen, 1996). The basic idea is that such instruments provide an incentive for companies or groups of people at work to stick to certain (legal) standards, as a benefit can be achieved in exchange for appropriate (more safe) behavior.

Another approach used for behavior change is the use of bonus systems. In another study arm of Gregersen's work (1996) the effect of a group‐based bonus system on the reduction of traffic‐related accidents at work among drivers was assessed. The group‐based reward system included the whole working unit that resulted in peer pressure in each group (and among groups). At the outset, the group was given a money level, based on the number of vehicles (SEK 200 per vehicle). An average group with 30 cars started with SEK 6000. For each accident caused by a driver in the group, the amount of money was reduced by SEK 100 or 200 depending on seriousness—for all drivers in the group. After 1 year, the remaining money was given to the drivers for a group activity such as a party, a pleasure trip or buying something together, such as physical training equipment. This group level bonus system showed a significant effect on reduction of work related road traffic accidents (OR: 0.72, 95% CI: 0.53–0.96). However, we cannot fully rule out underreporting here, as it is likely that the peer pressure of the group will work both on the driving behavior and on the reporting behavior (Comparison A19).

A single study from Finland (Rautiainen et al., 2005), which took advantage of a natural experiment to assess effects of providing economic incentives (type 2.2.2) to farmers for lower injury claim rates, reported decreased rates of workers' compensation injury claims, but not for injuries with 30 or more days of work disability. The pattern of decreased claims by severity levels was observed in each category with up to 29 lost days (0 days, 16.3% decline; 1–6 days, 14.1% decline; 7–13 days, 19.5% decline; and 14–29 days, 8.4% decline), and is consistent with some underreporting. However, the authors pointed out that while underreporting would be expected to be greatest in the zero lost days' category, this was not the case. This would seem to indicate that underreporting cannot explain the full estimated effect of the intervention; however, no effects were observed on more serious events (Comparison B7, chapter 11.1.2.).

###### 5.4.5.3.1 Summary of the effect of economic incentives

The studies reporting on economic incentives were from transport and agriculture, both industries representing dynamic work environment. The two studies provide limited evidence of effects of economic incentives, at medium and long‐term follow‐up.

##### 5.4.5.4 Effects of engineering controls (Type 2.2.4, Supporting Information Appendix 12.3)

We included 17 studies evaluating engineering controls, three RCT study designs (Jensen et al., 1997; Prunet et al., 2008; van der Molen et al., 2011), four CBA designs (Banco et al., 1997; Bell, 2002; Grimmond et al., 2010; Harms‐Ringdahl, 1987), and 10 studies using serial measures (Alamgir, 2008; Briggs et al., 2003; Lawrence et al., 1997; Prezant et al., 1999; Reddy & Emery, 2001; Rogues et al., 2004; Schoenfisch et al., 2013; Smollen, 2004; Sossai et al., 2010; van der Molen et al., 2011; Whitby et al., 2008). A common characteristic of safety interventions based on engineering control is the focus on elimination or substitution of the hazardous substances or materials at the source. Another strategy is to separate the persons from the hazardous conditions by introduction of machine safeguards, safer hand tools, walkways, or other changes in the physical environment, which reduce safety's dependence on human behavior. Even though the studies evaluated different approaches to engineering controls in different contexts, they all built on the same basic principle of directly influencing workers' safety by eliminating hazards or separating the workers from the hazards, thus reducing dependence on human behavior (Comparison A21–A24 and Comparison B8–B10).

Jensen et al. (1997) found that double gloving reduced the rate of perforation of glove barriers during abdominal surgery, and thereby the number of episodes in which transmission of disease from patient to surgeon would be possible. The outcome measure was perforations of glove barriers. This classical approach to engineering control, where the double gloving provided a barrier between disease and surgeon, resulted in significant reduction in the risk of exposure to bodily fluids and thus the risk of transmission of diseases from patients to surgeon (OR: 0.33, 95% CI: 0.21–0.51) (Comparison A21, chapter 11.1.1.).

van der Molen et al. (2011) evaluated the effect of the introduction of injection safety needles provided by a commercial supplier (another arm of the study evaluated the effect of group discussions). The existing injection needles were replaced by the new injection needles with the safety device. A non‐significant 28% reduction in injuries was observed with this intervention (OR: 0.72, 95% CI: 0.29–1.83).

Grimmond et al. (2010) investigated whether enhanced container engineering increases disposal safety and reduces injuries in health care. Sharps safety devices were developed following the principle that enhanced container engineering increases disposal safety by reducing dependence on human behavior. This resulted in a relatively large reduction in container‐associated sharps injuries at short‐term follow‐up (OR: 0.18, 95% CI: 0.11–0.29). The reduction in sharps injuries in general was also reduced, but at a more modest rate (OR: 0.85, 95% CI: 0.74–0.99).

Banco et al. (1997) evaluated the effect of a new and safer case cutter on injuries among young unexperienced workers, compared to the use of a traditional case cutter. The results showed a reduction of accidents at short‐term follow‐up, but it was not significant (OR: 0.51, 95% CI: 0.19–1.37) (Comparison A22, chapter 11.1.1.).

Evidence of effect, as well as lack of effect, was demonstrated for engineering controls in four studies using serial measures that were conducted in healthcare. Smollen et al. (2004) found evidence that the introduction of a safety butterfly device (Type 2.2.4) resulted in a large decrease in needlestick injury rates (RR: 0.20, 95% CI: 0.07–0.55) that was sustained over 1.5 years of follow‐up (medium‐term). Similarly, Whitby et al. (2008) found a 51% reduction of needlestick injuries following replacement of conventional needles with safety devices (RR: 0.49, 95% CI: 0.43–0.56), at medium‐term follow‐up. In both cases the older devices were removed after the staff was trained in use of the new safety device. Reddy and Emery (2001) evaluated the effect of medium‐term availability of engineering controls on needlestick injuries among HCW. The engineering controls was a hospital‐wide implementation of safety syringes and needleless‐intravenous systems in an 800 bed hospital in Texas. However, HCW also had access to older devices, which may be the reason that the effect was more modest, compared to the results of Smollen (2004) and Whitby et al. (2008) (RR: 0.78, 95% CI: 0.65–0.93).

Analyses by Rogues et al. (2004) demonstrate evidence of an effect (OR: 0.62, 95% CI: 0.51–0.75) consistent with other reports of multifaceted safety interventions to prevent needlestick injuries using a campaign, training, and technological modifications (engineering controls) to reduce exposure. The intervention required action on the part of workers to trigger a sheath to cover needles' post‐phlebotomy, but old devices were removed from the workplace, guaranteeing use of the newer devices. While injury rates for all needlestick injuries were declining during this period, and compliance with universal precautions could contribute to the reduction in injuries, the decrease of the incidence of needle‐related injuries not related to phlebotomy was not significant. The analyses could have been improved through a more rigorous approach to analyses that formally used non‐phlebotomy needlestick injuries as an internal control (Comparison B9, chapter 11.1.2.).

In the same vein, Prunet et al. (2008) evaluated the effect of respectively passive (automatically triggered) and active safety catheters compared to conventional (non‐safety) catheters among health care personal. The two types of catheters should protect the HCW from contamination from the patients, by separating the HCW from the bodily fluids. The outcome was cases of blood splashes, but no information on number of staff were provided, and thus it was not possible to calculate effect sizes of the two interventions. However, the results indicated a better protection with safety catheters, with lower exposure to HCW, and the passive safety catheter provided better protection against splashes to the environment than the two other types. Automatically triggered passive safety triggers provided better protection in general (Comparison A22, for outcome 22.2: Risk and behavior, chapter 11.1.1.).

Bell (2002) evaluated whether West Virginia (WV) logging companies experienced a reduction in injuries after beginning to use feller‐bunchers, which are tree‐cutting machines, which replace some of the more hazardous work done with a chainsaw during harvesting operations in forestry work. This substitution of manual tree cutting methods using chainsaws with tree cutting machines had a strong protective effect (OR: 0.27, 95% CI: 0.14–0.52), when compared to the remaining logging industry not using feller‐bunchers (usual practice), at long‐term follow‐up (Comparison A24, chapter 11.1.1.).

Another study also took advantage of a natural experiment where a production line in a paper mill was redesigned. The investigators evaluated the effect on injuries after the introduction of the re‐designed machinery, using another department as control (Harms‐Ringdahl, 1987). Before the re‐designed machinery was introduced, a systems approach was used to analyze safety at the paper mill plant, including job safety analysis, energy analysis, and deviation analysis, as a basis for re‐design of the production system. The energy model derives its idea from early engineering prevention, which indicates that the injury of a person is caused from an uncontrolled flow of energy. The deviation analysis built on the assumption that accidents often are preceded by deviations from the normal and planned functions of a system. If deviations are eliminated or controlled, then accidents can be reduced. We considered that the introduction of the redesigned production line was the key component of the safety intervention. Like the feller‐buncher study above, a rather strong effect was observed (OR: 0.44, 95% CI: 0.26–0.74) at medium‐term (Comparison A23, chapter 11.1.1.). However, the frequency of accidents was about six times higher in the intervention unit compared to the control unit, and there is thus a serious risk of regression to the mean. Even though there are several limitations in these study designs, such studies are important, as it is not easy to acquire access to these types of safety interventions where very expensive re‐designed machinery is introduced. The intervention was not designed as a research study, and it is understandable—and not inappropriate—that high‐risk groups may be selected for early intervention efforts.

In other types of engineering interventions (2.2.4), Alamgir et al. (2008) reported a gradual decline in musculoskeletal injuries in the 4 years following installation of ceiling lifts in three long‐term care facilities (RR: 0.56, 95% CI: 0.47–0.67). In contrast, Schoenfisch et al. (2013) observed an immediate decline in musculoskeletal injuries associated with patient handling in a community hospital following placement of mobile lift equipment and introduction of a “minimal manual lift” policy (RR: 0.56, 95% CI: 0.46–0.87). In another study arm (medical center) no effect was seen (RR: 1.01, 95% CI: 0.79–1.29). Overall, there appeared to be little effect of the intervention. The changes observed were more consistent with suppression of reporting caused by an institutional policy change that called on payment for work‐related injuries by each clinical unit; adoption of equipment use was demonstrated to be low.

All four of these studies involved engineering changes in health care settings, yet they have important distinctions. In two of the needlestick interventions (Smollen, 2004; Whitby et al., 2008), once the staff were trained, the older devices were removed essentially assuring use of the new safety devices. Sossai et al. (2010) reported on the long‐term effect of safety catheter use on the annual incidence rate of needlestick injuries 2003–2007. The introduction of catheters remained in place throughout 2 years of the follow‐up; a campaign was also launched, which might have increased the reporting of cases. It was not possible to estimate an effect size for this study.

In these cases, the use of the engineering solution did not require any active decision making on the part of staff, and no alternative was available, that is, reducing dependence on human behavior. The lift equipment can reduce hazardous exposures, but it is dependent on staff adoption to be effective.

Briggs et al. (2003) reported mixed findings regarding the effectiveness of Supermax prisons' effect on officers' safety. For the purposes of such a drastic move to isolation of prisoners, the authors considered the findings “did not provide the necessary onus of evidence” to support the use of Supermax prisons. This study involved retrospective analyses of data taking advantage of a natural experiment (Comparison B10, chapter 11.1.2.).

Prezant et al. (1999) demonstrated a decline in lower extremity burns among firefighters in NYC, following requirements for a new protective uniform across the entire department that was designed to prevent extremity burns, compared to the traditional uniform. The modified uniform significantly decreased incidence and severity of lower extremity burns (by 85%) in structural fires. Analyses controlled for number of structural fires and serious fires (OR: 0.36, 95% CI: 0.34–0.39). Head burns, which would not be affected by the altered uniform, decreased 40% as well; this may reflect differences in the fires, and full effects on the lower extremity burns may not be attributable solely to the uniform (Comparison B9, chapter 11.1.2.).

###### 5.4.5.4.1 Summary of the effect of engineering controls

Engineering approaches provided in general high reductions in injuries. The effects were particularly greater in cases where the safety intervention was able to be made independent of human behavior (Bell, 2002; Grimmond et al., 2010; Jensen et al., 1997; Smollen, 2004; Whitby et al., 2008). In particular for engineering solutions, there is moderate evidence that safety needle systems yield high reduction in incidents for HCW when the traditional needles are removed from the workplace. In other cases, where worker behavior changes were required, effect sizes were lessened (Reddy & Emery, 2001; Schoenfisch et al., 2013).

Meta‐analysis (Comparison A22) demonstrated that engineering approaches provided strong evidence of strong effects at short‐term follow‐up (OR: 0.28, 95% CI: 0.10–0.75; *I* ^2^ = 70%), for CBA studies. van der Molen et al. (2011) RCT study provided a non‐significant decline in injuries. There was insufficient evidence for effects at medium‐term and long‐term follow‐up. There was limited evidence for strong to very strong effect of engineering controls at post‐test (OR: 0.33, 95% CI: 0.21–0.51, *I* ^2^ = not appl.). This was partly backed up by the studies using serial measures, even though they only indicated a moderate effect at short‐term or long‐term follow up (Comparisons B8–B10).

Evaluations of engineering solutions, that require active rather than passive adoption by staff, benefit from the incorporation of process evaluation efforts and intermediate measures of adoption to understand if an intervention failure is of an engineering nature, or one of lack of adoption, as it appeared to be in the Schoenfisch et al. (2013) and Reddy and Emery (2001) studies. Passive engineering controls tend to have higher effects than active. A meta‐analysis of passive engineering controls with RCT and CBA designs showed a strong effect (OR: 0.31, 95% CI: 0.24–0.42, *I* ^2^ = 63%). For the serial measures the meta‐analysis showed a non‐significant effect (OR: 0.55, 95% CI: 0.30–1.01) for active engineering controls, and an odds ratio (OR: 0.58, 95% CI: 0.46–0.71) for passive engineering controls. The CIs are overlapping, but indicate a stronger effect of passive engineering controls (tables not presented).

##### 5.4.5.5 Effects of administrative controls (Type 2.2.5, Supporting Information Appendix 12.3)

Administrative controls comprise organizational policies and procedures, such as the introduction or modification of safety policies (e.g., new lifting policies) or Safety Management Systems, including modifications of monitoring, feedback and learning systems (e.g., procedures for incident or injury reporting, internal audit systems and inspections). Administrative controls exert some sort of coercive power on organizational members. However, they are dependent on being monitored, and that organizational procedures are in place to ensure a sustained effect of the controls.

One study examined the effect of administrative controls (2.2.5) and provided evidence of effectiveness. Crime levels and assault rates in smaller liquor stores (Casteel et al., 2004) decreased through business participation in Crime Prevention Through Environmental Design (CPTED) that included safer measures to handle cash, lighting, signs etc.; effectiveness was greater among stores with higher compliance with recommended components.

##### 5.4.5.6 Effects of soft regulation (Type 2.2.3, Supporting Information Appendix 12.3)

Soft regulation is a group of safety interventions where firms or organizations accept voluntary measures, such as agreements among social partners, corporate social responsibility policies, benchmarking, or voluntary external controls (e.g., ISO/OHSAS or other safety standards or guidelines). We identified one study (RCT) evaluating the effect of soft regulation (one study arm of the Hogg‐Johnson et al., 2012 study, the other study arm was enforcement).

Hogg‐Johnson et al. (2012) evaluated consultation where interested firms could consult with an sector specific Health & Safety Association (HSA) consultant to review their current practices, conduct gap analyses, or develop OHS plans, compared to no intervention. HSAs were able to provide a range of products and services including training and OHS certification. There was no significant effect (OR: 1.05, 95% CI: 0.92–1.20) (Comparison A20, chapter 11.1.1.).

This one RCT study provided no evidence for the effect of soft regulation. We judge the level of evidence to be limited, as only one high‐quality study was included in the analysis.

##### 5.4.5.7 Effects of social marketing (Type 2.2.8, Supporting Information Appendix 12.3)

One study using serial measures (Chapman et al., 2011; Chapman et al., 2013) investigated the effect of social marketing by use of existing and trusted channels of information to increase awareness and encourage adoption. The aim of this approach was to increase the information flow on measures that were not widely used, in this case effort to improve use of three safer and more profitable production practices, including barn lights, silage bags, and calf feed mixing sites. We used the updated version of the study from 2013 with a 7‐year follow‐up to measure the long‐term effect. The study used adoption of a practice as outcome (behavior), and found an effect of the intervention (SMD: −1.72, 95% CI: −2.22, −1.22) (Comparison B17, chapter 3.1.2.).

However, the study had some limitations. Comparisons between different states were used in the two reports. There was no difference in adoption of any of the three measures between the Wisconsin and the Maryland farmers after 4 years, while there was indeed a difference in adoption between the Wisconsin and NY farmers after 7years. However, we know nothing about the baseline in NY. Both the intervention group and the comparison group reached similar levels of adoption points toward a secular trend. Since the intervention was also partly implemented in the comparison group, and this group had a delayed response, the conclusion that the intervention was effective is not unreasonable. The shape of the intervention was not described, and there was an initial effect but no effect after 1–2 years. In addition, we considered some risk of reporting bias, as data for all years was not included in the final analysis.

##### 5.4.5.8 Summary of the effect of structural safety interventions

Overall, from this review process we judge that there is some evidence demonstrating that regulation can contribute to the prevention of accidents at work at long‐term follow‐up, even though effect sizes were little to moderate. We also find evidence that in some cases regulation does not work. Specifically, if there is no scientific evidence for the legislative requirements. It is therefore not surprising that injury reductions were not observed (Marlenga, 2006; Monforton & Windsor, 2010). In contrast to legislative effects, the evidence that enforcement activities can work was more consistent, but with even lower effect sizes, compared to legislative effords.

The engineering approaches in general provided modest to high reductions in injuries. The effects seem to be particularly high in cases where the safety intervention was made independent of human activities (Alamgir, 2008; Jensen et al., 1997; Bell, 2002; Grimmond et al., 2010; Smollen, 2004; Whitby et al., 2008). In other cases, the human factor seemed to reduce effects to a degree (Reddy & Emery, 2001; Schoenfisch et al., 2013).

Relatively strong effects were observed for engineering controls aimed at reducing needlestick injuries in hospitals, in particular when the required human behavior component could be reduced. Engineering controls are classical approaches where equipment with high‐risk, such as traditional syringes, are replaced with equipment with less risk, such as needleless intravenous injection systems. Such initiatives often have a major impact, as they will reach all employees concerned because they are independent of human behavior. These studies are not only relevant in the specific work context of health care, as it must be assumed that the substitution principle (eliminating the risk at the source) of injury prevention, applies more broadly to safety prevention at work. Based on this, we judged that engineering controls have medium to strong effects, and that the quality of the evidence is strong. Larger effects were seen with passive engineering controls, that is, safety works independently of “decision‐to‐use” by workers at the worksite.

The results overall show that effect sizes of the structural measures range from modest to strong effects, with higher effects when safety interventions to a lesser degree are independent of human activities. This is well in accordance with theoretical (conceptual) knowledge related to the Public Health Hierarchy of Hazards Control (PHHHC).

#### 5.4.6 Multifaceted safety interventions (Main type 3.0, Supporting Information Appendix 12.3)

The following types of safety interventions combine two or more components directed at the individual level (type 3.1), at the organizational level (type 3.2), or across levels (type 3.3).

##### 5.4.6.1 Effect of combinations of components at the individual level (type 3.1)

This group includes interventions directed at the individual level that combine attitude modification and behavioral modifications. We retrieved two RCT studies (Rasmussen et al., 2003; Rautiainen et al., 2004) and four CBA studies (Forst, 2004; Kim et al., 2004; Knapik et al., 2004; Peate et al., 2007). These interventions each combined a variety of intervention elements to achieve an effect, including both attitudinal and behavioral components. We found two safety interventions that integrated physiological changes with attitude modifications (Knapik et al., 2004, Peate et al., 2007). We found no studies in this group using a serial measures design to assess effects (Comparisons A25–A30, chapter 11.1.1.).

Two studies evaluated effects of a combination of approaches directed at the individual level on injuries in farming. Rautiainen et al. (2004) study investigated the Iowa Certified Farm Safety (CFS) program in a sample of 316 farms in the US and evaluated the effect at 3 years. The size of the farms was not clear from the paper. The CFS program comprised onsite safety reviews, health screening for farmers (farm operators), as well as education and monetary incentives for participation. No significant difference was found between intervention and control groups (OR: 0.99, 95% CI: 0.63–1.56) (Comparison A27, chapter 11.1.1.). The multifaceted intervention in Rasmussen et al. (2003) study included 201 small farms in Denmark with 6 month follow‐up. The intervention program comprised some of the same elements as in Rautinen's (2004) study, excluding monetary incentives. Additionally, focus group discussions were conducted on accident occurrence and prevention, and individual feedback on problems, risks, and hazards was provided. Rasmussen found a difference between intervention and control that was not significant for the outcome “all injuries” (OR: 0.57, 95% CI: 0.29–1.12); also no significant effects were observed for more serious injuries requiring medical care (Comparison A25, chapter 11.1.1.).

Knapik et al. (2004) evaluated the effect of a multifaceted safety intervention that included modified physical training, injury education, and injury surveillance in two groups of soldiers. There was a significant difference in injury outcome between male soldiers attending United States Army Ordnance School Advanced Individual training and a historical control group (OR: 0.66, 95% CI: 0.47–0.93). For the female soldiers no significant difference was seen in injury outcome (OR: 0.73, 95% CI: 0.30–1.78) (Comparison A26, chapter 11.1.1.).

Peate et al. (2007) evaluated a multifaceted intervention including group seminars and a firefighter competency training program with individual training. The authors mention that the intervention reduced the number of injuries by 42% over a 12 month period as compared to a historical control group (Peate et al., 2007), but this could not be justified by the data, and thus a clear effect estimate was not possible to establish from this study. In addition, the group level analyses were not tied to any measure of fitness intervention that was applied at the individual level. For this reason we do not know if those with better fitness scores are the ones who had fewer injuries.

Kim et al. (2004) evaluated a back education program (the Back Informed Program) to reduce injuries to firefighters in Toronto, Canada. It offered employees job‐specific education and ergonomic advice, exercises and hands‐on sessions. The study was not strong and the data presented were unclear. The number of lost time injuries was two in the intervention group, and “about the same size” in the control (not reported) (Comparison A27, chapter 11.1.1.).

Forst (2004) evaluated the Community Health Worker (CHW) “promotor de salud” model as a tool for reducing eye injuries in Latino farm workers. The effort included safety training and safety promotion related to use of protective eyewear, as well as information on preventing eye injuries and illnesses in Latino farm workers. An effect of the program was found on the self‐reported use of eyewear among the Latino workers (SMD: −0.58, 95% CI: −1.04, −0.13). However, the study had several shortcomings, and there was considerable loss to follow‐up, also related to the difficulties of evaluating this among Latino farm workers (Comparison A27, chapter 11.1.1.).

###### 5.4.6.1.1 Summary of the effect of multifaceted interventions including components at the individual level

The two RCT studies included did not report significant results on safety interventions combining both attitudinal and behavioral components. The CBA studies indicate some effect, but only one study had significant results. We did not perform meta‐analysis, because of high heterogeneity, probably due to different combinations of components.

Taken together we judge that safety interventions combining attitudinal and behavioral components seem to have no or a low effect on reported work injuries, and the level of evidence is moderate.

##### 5.4.6.2 Multifaceted safety interventions combining approaches at the group or organizational level (type 3.2)

Safety interventions directed at the group or organizational level may combine various approaches, such as modifications of climate, norms, engineering, or legal initiatives. We found eight studies exploring effects of approaches at the group or organizational level, two studies with a CBA design (Carrivick et al., 2002; Lopez‐Ruiz et al., 2013) and four studies with a serial measures (ITS) design (Chhokar et al., 2005; Fujishiro et al., 2005; Gershon, 1990; Park et al., 2009) (Comparison A30 and Comparisons B13–B14).

Lopez‐Ruiz et al. (2013) evaluated the effectiveness of an occupational injury prevention program, known as the PAP that focused on companies with high incidence rates of occupational injuries. The intervention combined official visits by a technician of the Safety and Health authority of the Valencia Region to companies with an action protocol; the technician writing a report describing the preventive situation of the companies, evaluating whether companies followed legal rules concerning preventive measures—mainly related to safety aspects of machinery, equipment, tools, devices, clean spaces, etc.; offering solutions and technical support and establishing deadlines to correct detected faults; carrying out a second official visit to check on the advances and changes produced; and finally, even proposing possible sanctions by the labor authority, after finding that recommendations had not been fulfilled. They reported a reduction in injuries at long‐term follow‐up (OR: 0.46, 95% CI: 0.33–0.64) (Comparison A29, chapter 11.1.1.).

Carrivick et al. (2002) evaluated the effectiveness of a consultative workplace risk assessment team in reducing the rate and severity of injury among cleaners in a 600‐bed hospital. The intervention included an iterative injury risk identification, assessment, and control process, and feedback/recommendations to leaders. The study applied a participatory approach, where a workplace risk assessment team including employees supported the risk identification and control process, and resulted in a reduction in injuries (OR: 0.35, 95% CI: 0.23–0.55). This study had serious RoB, and thus low quality (Comparison A28, chapter 11.1.1.).

Two studies on the same initiative (but different populations) in the US State of Ohio focused on reducing musculoskeletal injuries in long‐term health care establishments (Fujishiro et al., 2005; Park et al., 2009). The state policy initiative allowed institutions to seek consultation and training as well as funds for the purchase of lifting devices and assistance with initiation. The institutions could select what aspects of the overall program they wanted for their own needs. Fujishiro et al. (2005) reported that their results suggested an effect of consultation and support to purchase ergonomic interventions (RR: 0.77, 95% CI: 0.69–0.86). The Park study that followed several years later reported that the program expenditures were equivalent to savings from fewer workers' compensation claims (RR: 0.59, 95% CI: 0.41–0.85); they further concluded that injury rates did not decline with consultation activities alone, and findings regarding ergonomics alone were inconclusive (Comparison B13, chapter 3.1.2.).

Chhokar et al. (2005) reported that long‐term follow‐up of the introduction of overhead ceiling lifts in a long‐term care facility was effective in reducing the risk of injury to workers, as claims continued to decline for 3 years post‐intervention. This was a small study (involving only 50 injury events) in one institution, and the authors described the work as a longitudinal case series. Injury counts, rather than rates, were reported, and there were not stable numbers of events before the intervention, raising some concern about possible regression to the mean. We recalculated the number of claims by use of regression analysis based on three points before and three points after (the slopes b2‐b1), and calculated the SE from the p‐value. The effect was of borderline significance (OR: 0.96, 95% CI: 0.93–1.00) (Comparison B13, chapter 3.1.2.). However there was a gradual decline in lift transfer injuries that was not observed for repositioning injuries, providing some support for an intervention effect.

Gershon et al. (1999) reported needlestick injury reductions (OR: 0.29, 95% CI: 0.19–0.46) in one hospital associated with a multifaceted approach to prevention that included engineering modifications (safety devices, new disposal systems) and administrative controls in the workplace. Reductions were sustained over a 6 year follow‐up (Comparison B14, chapter 3.1.2.). The last study in this group (Bell & Grushecky, 2006) found no evidence of effect of the West Virginia Loggers Safety Initiative (Comparison B14, chapter 3.1.2.). The initiative included 8 h of training provided to company representatives and site visits to assess safety practices with associated incentives (OR: 1.00, 95% CI: 0.44–2.27). This was the only study in the group (multifaceted at group/organizational level) where engineering controls were not included in the safety intervention. High risk of injury persisted in the industry even in companies participating in the initiative. In this respect, it is interesting to compare with the other logger study (Bell, 2002), where manual tree cutting methods were replaced by tree cutting machines (engineering control), which had a strong effect (OR: 0.27, 95% CI: 0.14–0.52).

###### 5.4.6.2.1 Summary of the effect of multifaceted interventions at the group/organizational level

We refrained from doing meta‐analysis of multifaceted interventions at the group/organizational level, as the heterogeneity was rather high (*I* ^2^ > 85%). The high heterogeneity can partly be explained by the variation in components when multifaceted approaches are evaluated. Serious RoB was assessed for two studies using serial measures. Considering the quality of studies and the estimated effect sizes for each study, we judged moderate evidence that combining components at the group/organizational level provide strong effects at medium‐term follow‐up and limited evidence for a moderate effect at long‐term follow‐up. Multifaceted components using engineering controls indicate a higher effect.

##### 5.4.6.3 Effect of multifaceted safety interventions across levels (type 3.3)

This group includes interventions directed at both the individual level and the group or organizational level, and may include various components across levels, such as attitude modifications or modification of behavior, combined with climate intervention, engineering controls, or changes in legislation. These interventions thus have in common that various approaches at the individual and group or organizational level are combined to achieve an effect. Integration of individual level and group or organizational level studies, was the largest group of multifaceted safety intervention evaluations we identified, with 20 safety interventions. Two interventions with RCT design (Peek et al., 2004; Srikrajang et al., 2005), five with CBA design (Black, 2011; Evanoff et al., 1999; Parker et al., 2009; Rasmussen et al., 2006; Valls et al., 2007), and 13 interventions using serial measures (Bell et al., 2008; Bull, 2007; Garg, 1999; Lipscomb et al., 2008; Lipscomb et al., 2010; Mancini, 2005; Miller et al., 2007; Mode, 2012; Passfield, 2003; Saari & Näsänen, 1989; Spangenberg et al., 2002; Wickizer et al., 2004; Zafar et al., 1997) (Comparison A30 and Comparisons B15–B16, chapter 13.1).

Peek et al. (2004) evaluated a multifaceted safety prevention approach, CPTED, which included environmental, administrative and behavioral approaches. The included intervention businesses made their own decisions about which recommendations to implement. We note that there is an increased level of crime reporting for both intervention and control, and no significant effect of the interventions (OR: 0.88, 0.67, 1.17) at short‐term follow‐up (Comparison A30, chapter 11.1.1.).

The outcome of the other RCT study (Srikrajang et al., 2005) was risk or behavior. This multifaceted intervention integrated the use of posters, educational activities, problem solving in work groups, and technological modifications to promote safe nursing practices. There was a significant effect at short‐term follow‐up (12 months) of this multifaceted approach to change nursing practices related to the prevention of needlestick and sharp injuries (SMD: −1.43, 95% CI: −1.67, −1.19). Parker et al. (2009) evaluated the effect of the Minnesota Machine Guarding Program that consisted of inspection of businesses for machinery problems, feedback on problems and remediation. There was no effect reported. It was concluded that simple and easy‐to‐use assessment tools allowed businesses to significantly improve their safety practices, and safety committees facilitated this process. However, the conclusions are based on before and after and risk factor analysis, and not the data used for the RCT study design. It is therefore reasonable to say that results of the randomized evaluation had no effect. We calculated the SMD of the intervention using the provided machine safety scores before and after the intervention, and by using the management only group as control, and found a non‐significant effect (SMD: −0.26, 95% CI: −0.90, 0.39) (Comparison A30, outcome: Risk and behavior, chapter 11.1.1.).

Black et al. (2011) evaluated the effectiveness of a multifaceted Transfer, Lifting and repositioning (TLR) program to reduce musculoskeletal injuries among direct HCW. The study integrated safety training and the implementation of a standardized patient handling needs assessment and management system. They found about a 30% decrease in injuries at short‐term follow‐up (12 months) (OR: 0.69, 95% CI: 0.67–0.72) (Comparison A30, chapter 13.11.).

Passfield (2003) investigated a multifaceted safety intervention including safety training and safety policy (NO‐LIFT Policy) and provision of lifting equipment in a public hospital. It was unclear whether equipment was used, (not reported), but probably the policy was established and the population participated in the training. The effect was significant (OR: 0.38, 95% CI: 0.16–0.93).

Rasmussen et al. (2006) evaluated a multifaceted intervention with safety training, safety coaching of workers and managers, and employee participation. There was a decrease in accidents from falls over time, but the accidents in the control group decreased further, and overall there was no significant effect of the intervention (depicted data from graphs, OR: 1.31, 95% CI: 0.62–2.77).

Valls et al. (2007) evaluated a multifaceted intervention including a slide show on risk factors and consequences of blood borne pathogens, posters and safety training on correct use of phlebotomy systems, and technological modifications in the form of implementation of new phlebotomy systems (engineering control). They observed a decrease in the rate of accidents after 6 month follow‐up (short‐term) (OR: 0.10, 95% CI: 0.02–0.49) (Comparison A30, chapter 13.11.).

Evanoff et al. (1999) evaluated a multifaceted intervention including safety training and feedback, and implementation of new lifting procedures including a participatory ergonomics program in health care among hospital orderlies. There was a significant effect of this intervention (OR: 0.50, 95% CI: 0.35–0.71) after 2 years follow‐up (medium‐term). However, employment turnover was high in the intervention group, and no information was provided as to whether the control and intervention group had similar characteristics at the outset (Comparison A30, chapter 13.11.).

Effectiveness of interventions that included more than one component across levels (3.3) was more likely for those with an engineering or administrative control. Spangenberg et al. (2002) studied a natural experiment in the construction industry, specifically a major bridge and tunnel project, which aimed to decrease overall injury rates on this project. The study found that the safety campaigns and economic incentives (award given twice a year to be shared by the employees at the safest site), including feedback to workers and workplace (site results and total results) did not result in significant behavior change, even though most had awareness of the effort; only 10% of the workers indicated that they had changed their working habit during this intervention. It showed a borderline significant drop in injury rates of 25% after control for periods of heavy and light construction work (OR: 0.75, 95% CI: 0.57–0.99).

Bell et al. (2008) demonstrated a 58% reduction (OR: 0.42, 95% CI: 0.33–0.54) of STF accidents in hospitals using a multifaceted intervention that included assessment of causes, hazard assessments, housekeeping changes, awareness campaigns, ice removal, flooring changes, slip resistant footwear for high‐risk subgroups of workers, etc. This was a well‐informed intervention; the approach was supported by the epidemiology of these injuries, which included a multi‐causal problem. An ITS design with an external comparison (to US Bureau of Labor Statistics data reported by hospitals over a 10‐year period) was used to measure effect.

Martin et al. (2009) demonstrated a non‐significant reduction (OR: 0.79, 95% CI: 0.59–1.06) in patient handling injuries, through a program that included attitude modification, training, engineering controls (lift equipment), administrative controls, and employee participation in the intervention process. Back injury rates declined in the implementation period, but not as much afterward. The authors reported intermediate measures, and reported threats to sustained activity including fewer funds, storage issues for equipment, no designated resource people; they stated that waning levels of interest over time required constant or more sustained activity to maintain effects. There were no changes seen in patterns of injury in control injuries, that would not be affected by the program and lift equipment, which added strength to their findings (Comparison B15, chapter 11.1.2.).

Lipscomb et al. (2008/2010) demonstrated effectiveness of a safer trigger design on pneumatic nailguns and training in prevention of residential construction injuries at long‐term follow‐up through serial cross‐sectional analyses based on a natural experiment. Over 4‐years of observation, crude injury rates based on hours of tool use declined 43% as safer trigger use and prevalence of training increased. The test for trend approached statistical significance (*p* = 0.056); the authors discussed the loss of power over time as injury rates declined resulting in a less robust measure in the final year of follow‐up. Data on exposure (type of trigger used and training) and injuries were collected on an individual basis allowing calculation of population attributable risk measures. In each year of follow‐up, the engineering intervention was more effective from a population perspective. The training measurement was whether having had any prior training or not, and as such, this intervention could be considered largely one of engineering controlled for effects of training (Comparison B16, chapter 11.1.2.).

Mode (2012) and Porru (2012) reported on public health practice efforts to respectively decrease air crashes in Alaska, and to prevent injuries among foundry workers in Italy. Both safety interventions included structural components, such as competitions, benchmarking, legislative initiative with congressional funding (Mode, 2012), technical and organizational procedures (Porru, 2012). It appears from these reports that public health practice over a period of time can be effective in preventing injuries; the authors made no attempts to disaggregate elements involved, but rather evaluated the process of surveillance, risk factor, identification, intervention, and evaluation as an ongoing process over several years (Comparison B16, chapter 11.1.2.).

Bull (2007) and Mancini (2005) demonstrated effects of prevention of eye injuries at long‐term in metal workers in Norway and Italy, respectively, in efforts that included use of appropriate PPE and administrative controls that required and/or fostered use. Bull reported on the experiences in one setting using presentation of simple rates over time. The intervention involved an enforced policy requiring use of eye protection; workers were given two warnings and then fired for lack of compliance. Injury rates immediately declined markedly (6.09 to 0.42 injuries per million working hours) after eye protection became mandatory, and the decline was sustained at 10 year follow‐up. The Italian intervention (Mancini et al., 2005) focused on multiple small facilities in one region of Italy. The intervention was based on considerable input from a needs assessment and follow‐up of reported injuries. Interviews were conducted with workers about barriers to eye protection, unions were consulted about approaches and worksites were visited. A tailored educational brochure was developed, as were messages for TV and radio, and physicians provided workers with individual consultations. A 42% sustained reduction in eye injuries was reported in analyses of this ITS design with both internal and external controls (Comparison B16, chapter 11.1.2.).

Miller et al. (2007) at long‐term follow‐up (Comparison B16, chapter 11.1.2.), and Wickizer et al. (2004) at medium‐term (Comparison B15, chapter 11.1.2.), both found evidence of effect of peer‐based substance abuse prevention and federally mandated drug and alcohol testing in a transportation company, as well as a multifaceted Drug Free Workplace program in various industries, respectively. Results varied by occupational group and for Wickizer et al. (2004), regression to the mean was a bias, as large differences in injury rates at baseline.

###### 5.4.6.3.1 Summary of the effect of combining components across levels (type 3.3)

Moderate effects can be seen when intervention components are combined across levels and the patterns are rather consistent. Effectiveness of interventions that included more than one component across levels was more likely for those studies including an engineering control. All RCT and CBA studies that combined components across levels included at least one engineering control as one element. The heterogeneity for the CBA studies was moderate (*I* ^2^ = 74% for short‐term follow‐up studies). There is moderate level of evidence that multifaceted safety interventions across levels have moderate effect at short‐term follow‐up (OR: 0.62, 95% CI: 0.40–0.96; *I* ^2^ = 74%) (Meta‐analysis of CBA studies in Comparison A30, outcome 32.1 injuries, chapter 11.1.1.).

However, the heterogeneity for the serial measures (ITS) study design, for this type of interventions at medium‐term follow‐up, was considerable (Heterogeneity: *I*² = 94%). If studies that did not include engineering or administrative control components were excluded, then the heterogeneity became moderate. This indicates that studies including engineering or administrative control components are more homogeneous in their approach and yield more similar effect sizes, or that engineering approaches override the other included components.

#### 5.4.7 Other approaches outside designated interventions [type 9]

##### 5.4.7.1 Serial measures (ITS) studies

The following intervention did not fall into one of the designated intervention classifications demonstrated some evidence of effectiveness in prevention injuries.

Menendez et al. (2012) found some indication in the Fatality Assessment and Control Evaluation (FACE) program in the US—and associated follow‐up activities—may decrease fatal injuries in targeted areas (falls from height and electrocution); these multiple state run programs involved considerable variation in program development and follow‐up. Evidence was not conclusive, and not over‐stated, with the investigators simply reporting “some indications” that the programs may be effective.

## 6 DISCUSSION

### 6.1 Summary of the main results

In this review we examined broad classifications of safety interventions to provide an overview of the effectiveness of the main types of safety interventions, and to compare the various approaches with regard to their relative effectiveness in preventing accidents, reduce injury risk factors or improving safety behavior at work (proximal risk factors). The goal was to provide an informed basis for stakeholders to select more effective approaches to reduce accidents at work.

The review took into account the variety of safety prevention activities initiated by companies or by external bodies for which assessments of effectiveness could be found. The process was framed in the conceptual model of Lund and Aarø (2004), as presented in Figure 1. Using this framework, safety interventions may be directed at the individual level, the group or organizational level, or across levels at the workplace. Furthermore, interventions may use one component or be multifaceted, and thus combine two or more main types of safety intervention components. Multifaceted approaches may combine intervention components at the individual level or at the organizational level, or combine components across levels. The Lund and Aarø model is a generalized model (logic model), that provided an understanding of the nature of safety interventions and possible pathways for prevention. To conceptualize the nature of some types of safety interventions we also make use of the PHHHC. Nevertheless, this approach cannot integrate well climate and culture or legislative safety interventions, and for this reason, we did not find the PHHHC useful as a generalized model. In this review, the specific types of safety interventions have been the subject of analysis. The types of safety interventions referred to by a number in the following (e.g., type 1.1.2) can be found in Supporting Information Appendix 12.3.

In the following, we present a summary of the main results. We carried out meta‐analyses for a sub‐set of safety interventions (Tables 3, 6, and 8) and supplemented these analyses with narrative analyses combining information on the intervention mechanisms and the effects of the included safety interventions (Tables 4, 5, and 7). For the main part of the evaluated types of safety interventions data only allowed to use a narrative approach for the evaluation. Section 12 includes Table 19 that defines ranges for the strength of effects, Table 20 with an overview of results based on meta‐analyses, and Table 21 with an overview of results based on narrative analyses.

**Table 3:** Summary of meta‐analysis for a subset of safety interventions directed at the individual level, by type of safety intervention, quality assessment, level of evidence and strength of effect

Number of safety interventions	Quality assessment				Level of evidence	Strength of effect	Meta‐analysis (injury outcomes)		
Types of safety interventions and follow‐up periods	High quality	Moderate quality	Low quality	Total	RCT and CBA	RCT and CBA	RCT	CBA	*I* ^2^, RCT/CBA
**1.1.0.: Attitude and belief modification:**									
**1.1.1** Safety campaign, by use of various means	**1**		**1**	**2**					
2 = Short‐term (−12 months)			1	1	Insufficient	*Not estimable*			
3 = Medium‐term (12–36 months)	1			1	Insufficient	*Not estimable*			
**1.1.2** Counseling approaches	**1**	**3**		**4**					
2 = Short‐term (−12 months)	1	1		2	Limited	None	OR: 0.67 [0.38, 1.21]		0%
3 = Medium‐term (12–36 months)		2		2	Limited	Strong	**OR: 0.52 [0.29, 0.93]**	**OR: 0.53 [0.36, 0.77]**	NA/NA
**1.1.3** Teaching, education to increase awareness	**1**	**1**	**1**	**3**					
2 = Short‐term (−12 months)	1	1	1	3	Limited	None		OR: 0.90 [0.56, 1.46]	37%
**1.3.0.: Physiological modification:**									
**1.3.1** Individual physical training	**1**	**2**	**1**	**4**					
2 = Short‐term (−12 months)	1	2		3	Limited	None		OR: 1.04 [0.75, 1.44]	0%
3 = Medium‐term (12–36 months)			1	1	Insufficient	*Not estimable*			/

Abbreviations: CBA, controlled before‐and‐after; *I* ^2^, Heterogeneity; NA, not applicable; RCT, randomized controlled trial.

**Table 4:** Summary of narrative assesment of safety interventions directed at the individual level *not included* in meta‐analysis, by quality assessment, level of evidence and estimated strength of effect

Number of safety interventions	Quality assessment				Level of evidence	Strength of effect
Type of safety intervention and follow‐up periods	High quality	Moderate quality	Low quality	Total	RCT, CBA, and serial measures (ITS)	RCT, CBA, and serial measures (ITS)
**1.1.0.: Attitude and belief modification, not specified:**			**1**	**1**		
**1.1.3 Teaching, education to increase awareness**	**1**		**1**	**2**		
3 = Medium‐term (12–36 months)	1		1	2	Limited	None
**1.2.0.: Behavior modification**						
**1.2.2 Safety training**	**2**			**2**		
2 = Short‐term (−12 months)	1			1	Limited	None
3 = Medium‐term (12–36 months)	1			1	Limited	Moderate
**1.2.4 Individual feedback or coaching**	**3**		**2**	**5**		
0 = Not reported or unclear			1	1	Insufficient	*Not estimable*
2 = Short‐term (−12 months)	2		1	3	Moderate	**None to little**
4 = Long‐term (36‐month)	1			1	Limited	None
**1.3.0.: Physiological modification:**						
**1.3.1 Individual physical training**			**1**	**1**		
4 = Long‐term (36‐month)			1	1	Insufficient	*Not estimable*

Abbreviations: CBA, controlled before‐and‐after; ITS, interrupted time series; RCT, randomized controlled trial.

**Table 5:** Summary of narrative analyses of safety interventions directed at group or organizational level, *not included* in meta‐analysis, by quality assessment, level of evidence and evaluated strength of effect

Number of safety interventions	Quality assessment				Level of evidence	Strength of effect
Type of safety intervention and follow‐up periods	High quality	Moderate quality	Low quality	Total	RCT, CBA, and serial measures (ITS)	RCT, CBA, and serial measures (ITS)
**2.1.0. Climate, norms or culture modifications:**						
**2.1.1 Goal setting and FB at group or org. level**		**2**	**5**	**7**		
2 = Short‐term (−12 months)		2	5	7	Limited	None
**2.1.7 Leadership‐based safety interventions**	**1**	**2**	**1**	**4**		
2 = Short‐term (−12 months)	1	2	1	4	Limited	**Little to moderate**
**2.2.0. Structural modifications:**						
**2.2.1 Legislative changes**	**1**	**0**	**8**	**9**		
4 = Long‐term (36‐month)	1	0	8	9	Limited	**Little to moderate**
**2.2.2 Economic incentives**	**2**			**2**		
3 = Medium‐term (12–36 months)	1			1	Limited	**Little to moderate**
4 = Long‐term (36‐month)	1			1	Limited	*Not estimable*
**2.2.3 Soft regulation**	**1**	**2**		**3**		
3 = Medium‐term (12–36 months)	1			1	Limited	None
4 = Long‐term (36‐month)		2		2	Limited	None
**2.2.4 Engineering controls**	**3**	**3**	**5**	**11**		
2 = Short‐term (−12 months)			1	1	insufficient	Moderate
3 = Medium‐term (12–36 months)	3	1	1	5	Strong	Moderate
4 = Long‐term (36‐month)		2	3	5	Limited	Little
**2.2.5 Administrative controls**		**1**	**1**	**2**		
2 = Short‐term (−12 months)		1		1	Insufficient	*Not estimable*
3 = Medium‐term (12–36 months)			1	1	Insufficient	*Not estimable*
**2.2.7 Enforcement of laws and regulations**	**1**	**2**	**3**	**6**		
4 = Long‐term (36‐month)	1	2	3	6	Moderate	**None to little**
**2.2.8 Social marketing and other approaches**			**1**	**1**		
4 = Long‐term (36‐month)			1	1	Insufficient	**Very strong**

Abbreviations: CBA, controlled before‐and‐after; ITS, interrupted time series; RCT, randomized controlled trial.

**Table 6:** Summary of meta‐analysis for a subset of structural safety interventions, directed at the organizational level, by type of safety intervention, quality assessment, level of evidence and strength of effect

Number of safety interventions	Quality assessment				Level of evidence	Strength of effect	Meta‐analysis (injury outcomes)		
Types of safety interventions and follow‐up periods	High quality	Moderate quality	Low quality	Total	RCT and CBA	RCT and CBA	RCT	CBA	*I* ^2^, RCT/CBA
**2.2.0.: Structural safety interventions:**									
**2.2.4** Engineering controls	**4**	**2**		**6**					
1 = Posttest	1			1	Limited	**Strong to very strong**	**OR: 0.33 [0.21, 0.51]**		NA
2 = Short‐term (−12 months)	3			3	Strong	Moderate	**OR: 0.72 [0.29, 1.83]**	**OR: 0.28 [0.10, 0.75]**	NA/70%
3 = Medium‐term (12–36 months)		1		1	insufficient	Strong		**OR: 0.44 [0.26, 0.74]**	NA
4 = Long‐term (36‐month)		1		1	insufficient	**Very strong**		**OR: 0.27 [0.14, 0.52]**	NA
**2.2.7** Enforcement of laws and regulations	**7**		**4**	**11**					
2 = Short‐term (−12 months)	1			1	Limited	Little		**OR: 0.86 [0.77, 0.95]**	NA
3 = Medium‐term (12–36 months)	2		4	6	Moderate	**None to little**	**OR: 0.99 [0.89, 1.10]**	**OR: 0.95 [0.93, 0.97]**	NA/0%
4 = Long‐term (36‐month)	4			4	Strong	Little		**OR: 0.96 [0.93, 0.98]**	0%
**2.2.7** Enforcement of laws w/penalties	**2**			**2**					
3 = Medium‐term (12–36 months)	2			2	Moderate	**None to little**		**OR: 0.95 [0.92, 0.98]**	0%

Abbreviations: CBA, controlled before‐and‐after; *I* ^2^, Heterogeneity; NA, Not applicable; RCT, randomized controlled trial.

#### 6.1.1 Safety interventions directed at the individual level

Individual level safety interventions include three main types of safety interventions, which are modifications of attitudes and beliefs, modifications in physiology to increase individual's resistance to acute physical exposures, and BBS interventions.

##### 6.1.1.1 Effectiveness of attitude and belief modifications (Main type 1.1.0) by use of three main types of safety interventions

We included 12 safety interventions (three using RCT, six using CBA, and three safety interventions using serial measures) as shown in Table 3 (meta‐analyses) and Table 4 (narrative analyses).

**Safety campaigns (type 1.1.1)**: We assessed two studies evaluating safety campaigns, where both studies were using a CBA design; one high‐quality study (Gregersen, 1996) evaluated the effect on injuries, and the other low quality study measured the effect on safety behavior through the use of preventive tools and eyewear (Forst, 2004).

*We conclude that* the evidence base was not strong enough to conclude on the effect of safety campaigns (Table 3, meta‐analyses, and Table 4, narrative analyses).

**Counseling approaches (type 1.1.2)**: We assessed four safety interventions, (two using RCT design and two using CBA design) that evaluated effects of counseling approaches, one of high quality and three with moderate quality. The key methodological limitations in the studies were that allocation concealment was not possible, and that the CBA study was not randomized. In addition, we judged that self‐reported outcomes in two of the studies as a RoB. A meta‐analysis was carried out (Table 3).

*We conclude that the level of evidence is limited that counseling approaches has no effect at short‐term follow‐up and strong effect at medium‐term follow‐up*.

**Teaching and educational approaches (Type 1.1.3):** We assessed three CBA studies and two studies using serial measures which evaluated teaching and education as means for modifying belief and attitudes, and in turn accidents.

Two CBA studies evaluating the effect of teaching and educational approaches on needlestick injuries had several methodological limitations, such as incomplete outcome data and unclear implementation fidelity (Mehrdad et al., 2013; Wang et al., 2003). Another study evaluating teaching and educational approaches (Johnson & Owoaje, 2012) used self‐reported behavior in face‐to‐face interviews, that we considered a RoB. We conducted a meta‐analysis for these studies (Table 3). Two studies using serial measures found no or modest effect (Table 4).

*We conclude that the level of evidence is limited for no effect of teaching and education at short‐term follow‐up and at medium‐term follow‐up*.

Thus there appears to be limited support for the KAP model in a workplace setting (Lund & Aarø, 2004), which assumes knowledge leads to changes in attitudes or beliefs, which in turn leads to changes in safety practices and injury. Limitations to this model in an occupational safety context have been described previously (McDonald et al., 2009).

There could be a potential for effects of group discussions or counseling approaches, which involve stronger interaction and involvement than one‐way educational approaches or safety campaigns through media, pamphlets, or other ways. Attitude shaping may be relevant in the context of other types of safety interventions, for example, multifaceted safety interventions (see below).

Overall, we did not find clear evidence that safety interventions based on attitude and belief modification as one‐way communications are effective in changing behaviors or injury risk. Meta‐analysis (Table 3) showed limited evidence of a strong effect of more interactive counseling approaches to reduce accidents at work at medium‐term follow‐up.

##### 6.1.1.2 Effectiveness of behavior based approaches (Main type 1.2.0)

We included six studies, representing seven behavior based interventions (four interventions using RCT design: Cheng & Chan, 2009; Daltroy et al., 1997; Jinnah et al., 2014 with two study arms; three safety interventions using CBA design: Banco et al., 1997; Gregersen, 1996; Quintana, 1999).

**Safety training (Type 1.2.2):** We evaluated two high quality CBA studies evaluating safety training. This category includes skill based training, for example, proficiency or dexterity of doing a manual work task, and not just attempts to change attitudes. Gregersen's (1996) study on Swedish telephone repair workers showed an effect at medium‐term follow‐up (2 years) of training on the reduction of accidents. A study to reduce cut injuries among young and inexperienced workers (Banco et al., 1997) used training in use of case‐cutters. The study demonstrated a modest change in reducing injuries, but was not significant.

*We conclude that the level of evidence is limited for no effect of safety training at short‐term follow‐up, and moderate effect at medium‐term follow‐up* (Table 4, narrative analyses).

**Individual feedback or coaching (Type 1.2.4):** Two studies judged to be of higher quality (Daltroy et al., 1997; Jinnah et al., 2014) pointed in different directions, where one study favored control and the other the intervention. Jinnah et al. (2014: study arm parent) found a significant effect of the behavioral approach on injuries, but did not use intention to treat analysis, and the outcome is measured as changes in behavior (seat belt use in ROPS tractors). The other RCT study of higher quality favored the control (Daltroy et al., 1997), even though it was not at a statistically significant estimate.

The RCT study of individual job coaching (Cheng & Chan, 2009) had some limitations, and we considered it low quality. The low quality was mainly due to the Hawthorne effect and that possible underreporting of injuries in the control group was likely to have biased the results, as management was keen on limiting work related muscular injuries. In addition, there was serious RoB for several other RoB dimensions.

*We conclude that the level of evidence is moderate for no effect of individual feedback or coaching at short‐term follow‐up and limited evidence for no effect at medium‐term follow‐up*.

All in all, the results indicate that behavioral approaches have no or little effect on prevention of injuries at work. Only safety training at medium term follow‐up provided limited evidence for a moderate effect (Table 4, *narrative analyses)*.

##### 6.1.1.3 Effectiveness of physiological modifications (Main type 1.3.0)

Safety interventions based on modification of physiology to increase individuals' resistance to accidental risks are a relatively new category of safety intervention. We included four studies (one RCT, three CBA and one serial measures study) evaluating five safety interventions. The studies used heterogeneous approaches, mainly mixed physical training methods, and one intervention was about weight loss. None of these safety interventions demonstrated significant effects on safety behavior or accidents, apart from one study (Morgan, 2012) that reported a positive effect of personal fitness, in the form of weight loss (type 1.3.9, other types). However, we judged the evidence of the relationship between weight loss and injuries to be unfounded.

Data allowed for a meta‐analysis for individual mixed physical training at short‐term follow up, and showed limited evidence for no effect on injuries of safety interventions using physiological modification. Analysis of medium‐term follow‐up gave insufficient evidence (Table 3, *meta‐analyses, and* Table 4, *narrative analyses*).

*We conclude that there was limited evidence for no effect of mixed physical training on reducing injuries at work at short‐term follow‐up, and insufficient evidence at medium‐term follow‐up*.

##### 6.1.1.4 Summary

Overall, individual level approaches appear to have little or no impact on reducing accidents or improve safety and behavior at the workplace, and the evidence range from insufficient to limited evidence for the interventions, apart from individual feedback or coaching providing moderate evidence for no effect.

#### 6.1.2 Safety interventions directed at the group or organizational level

Group and organizational level safety interventions include changes in climate, norms and culture, introduction of legislation and enforcement, engineering controls, administrative controls, soft regulation, and introduction of economic incentives.

##### 6.1.2.1 Effectiveness of climate, norms or culture modifications (main type 2.1.0)

We included no studies that investigated safety culture as a means to improve safety and reduce accidents. We included 11 studies under this main heading (two RCT, three CBA, and six studies with serial measures) assessing effectiveness of safety interventions to improve climate and safety norms, that is, improving the (shared) priority of safety in an organization/group, compared to other competing goals, as a means to improve safety and reduce accidents.

Seven studies used goal setting and feedback at group or organizational level (Cooper et al., 1994; Cunningham & Austin, 2007; Fellner & Sulzer‐Azaroff, 1984; Mattila & Hyoedynamaa, 1988; Ray et al., 1997; Sulzer‐Azaroff & de Santamaria, 1980; Sulzer‐Azaroff et al., 1990). These studies had several methodological limitations, such as no blinding of outcome assessors, regression to the mean, serious risk of spillover, and attrition.

*We conclude that there was limited evidence for no effect of goal setting and feedback at group or organizational level at short‐term follow‐up* (Table 5, narrative analyses).

Four studies using leadership based approaches to improve safety climate (Kines et al., 2010, 2013; Zohar, 2002; Zohar & Luria, 2003). Of those studies we considered higher quality, only one (Zohar, 2002) used occupational injuries as the outcome. This study showed a significant effect on injuries.

However, the quality of the evidence overall is limited that leadership based safety interventions can improve safety and reduce accidents. The effect pattern in the included studies was inconsistent and, in particular, there was no clear relationship between intermediary measures (climate scores, safety index scores and behavior) and reduction in injuries. There is a need for a better empirical basis in support of the well‐developed theoretical and conceptual approach used within safety climate research (Zohar, 1980; Zohar & Luria, 2003).

*We conclude that there is limited evidence for a little or moderate effect of leader based safety interventions on safe behavior and decrease in injuries at short term follow‐up* (Table 5, narrative analyses).

##### 6.1.2.2 Effectiveness of legislation (Type 2.2.1)

We included eight studies evaluating nine long‐term effects of legislation, which were all serial measure studies. These legislative efforts, or rule making, all used serial measures and all were long‐term evaluations. Methodological quality was limited by informal comparisons to other data, by a short “pre‐intervention” period, and inconsistency in effects measured by various types of outcome. However, several investigators justified their approaches well, used techniques to improve the internal validity of their work including internal and external comparisons, and stratified results documenting changes in groups more consistent with an intervention effect. Of note, we found evidence from two studies that legislation or rule‐making that lacks an evidence‐base for the intervention did not appear to affect injury rates (Marlenga, 2006; Monforton & Windsor, 2010). Attribution of declining fatalities to legislation was not consistently demonstrated in some of the studies (Bulzacchelli et al., 2007; Derr et al., 2001; Suruda et al., 2002), as they all demonstrated declining fatalities, but the findings did not clearly allow attribution to the standard in question. This weakens the overall evidence base for legislative safety interventions.

*We conclude that the level of evidence is limited that legislative efforts have little to moderate effects on prevention of injuries at work at long‐term follow‐up* (Table 5, narrative analyses).

Even though effect sizes were modest, population‐based impact can be considerable, given the number of work sites and workers that potentially can be affected through legislative interventions.

##### 6.1.2.3 Effectiveness of enforcement and compliance efforts (Type 2.2.7)

The policy initiatives implementing the enforcement of legislation were quite diverse. Still, the three studies looking at effects of enforcement in three different US states, respectively, demonstrated some consistent findings (Foley et al., 2012; Haviland et al., 2012; Levine et al., 2012). Of particular note were the findings of larger effects when penalties or citations were imposed (Foley et al., 2012; Haviland et al., 2012), rather than consultation visits or inspections without penalty (Haviland et al., 2012; Hogg‐Johnson et al., 2012; Levine et al., 2012). However, information on the level of penalties, and whether penalties were consistently imposed if violations were found, were lacking in the included studies.

Three of the six studies (16 study arms) exploring effects of activities designed to increase compliance with existing regulations such as consultation, inspections or enforcement included the more experimental designs. In contrast to legislative effects, the evidence that enforcement activities can work was more consistent, however, with lower effect sizes, overall. While legislation theoretically could have effects without enforcement, it seems reasonable that longer‐term effects would be enhanced by enforcement. The only RCT study (Hogg‐Johnson et al., 2012) did not show any effect. Two other high‐quality studies did show effect (Benavides et al., 2009; Levine et al., 2012), but no significant effect at medium‐term follow‐up. Some studies had problems with selective reporting (Haviland et al., 2012), as subgroup analysis was not specified (size of enterprises), and there was overlap of intervention periods in two study arms (Lopez‐Ruiz et al., 2014).

Meta‐analysis of the RCT and CBA studies showed that there was limited evidence for a little effect of enforcement and compliance at short‐term follow‐up, moderate evidence of from none to a little effect at medium‐term follow‐up, and limited evidence of a little effect at long‐term follow‐up. For the studies using serial measures to evaluate enforcement and compliance we assessed that there was moderate evidence for no or little effects of enforcements and compliance at long‐term follow‐up. Studies with control showed lower effect sizes, and RCT studies the lowest effect size (Hogg‐Johnson et al., 2012).

*We conclude limited evidence for a little effect of enforcement and compliance efforts at short‐term follow‐up and strong evidence for a little effect at long‐term follow‐up and moderate evidence of no or little effect at medium‐term follow‐up* (Table 6).

A cautious interpretation indicate a differential effect of enforcement over time, and that this effect is lower at medium‐term follow‐up. We cannot explain the mechanism of this pattern of differential effect over time on the basis of these studies.

##### 6.1.2.4 Effectiveness of economic incentives (Type 2.2.2)

Two studies reporting on economic incentives, one study using serial measures to evaluate economic incentives in agriculture (Rautiainen et al., 2005), and one study using CBA design evaluating economic incentives in the transport industry (Gregersen, 1996). Evidence of effectiveness is mixed with a weak link between economic incentives and work injuries.

The use of insurance premiums seems to influence the propensity of reporting accidents at work, rather than clearly improving safety on farms (Rautiainen et al., 2005). The study reported decreased rates of workers' compensation injury claims, but not for injuries with 30 or more days of work disability. This pattern was consistent with some underreporting.

The CBA study by Gregersen (1996) investigated economic incentives for workers in a transport company and found an effect of this.

*We conclude that the level of evidence is limited that economic incentives have from little to moderate effect on the reduction of accidents at medium‐term follow‐up* (Table 5).

We could not estimate the effect of economic incentives at long‐term follow‐up (Rautiainen et al., 2005).

##### 6.1.2.5 Effectiveness of engineering controls (Type 2.2.4)

We included 17 studies evaluating 19 safety interventions using engineering controls, four evaluations of engineering controls using RCT study designs, four using CBA designs and eleven using serial measures (ITS) designs. The engineering approaches in general provided modest to strong effects on improving safety and reducing accidents (Table 5, narrative analyses, and 5.4 meta‐analyses). The effects were particularly high in cases where the safety intervention was made independent of human behavior (Alamgir, 2008; Bell, 2002; Grimmond et al., 2010; Jensen et al., 1997; Smollen, 2004; Whitby et al., 2008). In other cases the human factor was reduced to a lesser degree, requiring decisions or allowing options on the part of workers potentially exposed to hazards (Reddy & Emery, 2001; Schoenfisch et al., 2013), which was reflected in lower effect sizes.

The reduction of needlestick injuries in hospitals provides a particularly strong example of the effectiveness of engineering changes that design out risk and thus reduce the opportunity for human decision making to influence effects. This type of engineering control works by eliminating the risk, and refers to a higher level of the hierarchy of controls.

Meta‐analyses were performed on evaluations of engineering controls using RCT and CBA designs, and showed limited evidence for a strong to very strong effect of engineering controls at post‐test, strong evidence for a strong effect at short‐term follow‐up, and insufficient evidence for medium and long‐term follow‐up, respectively (Table 6). On the basis of the serial measures we judged that there was insufficient evidence at short‐term follow‐up; strong evidence for a moderate effect at medium‐term follow‐up; and limited evidence for a little effect at long‐term follow‐up (Table 5).

*We conclude that there is limited evidence for a strong to very strong effect of engineering controls at post‐test, strong evidence for a moderate effect at short‐term follow‐up, strong evidence for a moderate effect at medium‐term follow‐up, and limited evidence for a little effect at long‐term follow‐up* (Tables 5 and 6).

Overall engineering controls provide moderate to strong effects on reducing accidents, however, with varying levels of evidence depending on the follow‐up. Strong effects were in particular seen in cases where the safety intervention works independently of individual human decision making or work practices, or where the risks were “designed out.”

##### 6.1.2.6 Administrative controls (Type 2.2.5)

One study examined crime levels and assault rates in smaller liquor stores (Casteel et al., 2004) participating in a CPTED that included safer measures to handle cash, lighting, signs, etc. Injury patterns were compared to non‐participating businesses, controlling for crime trends in neighborhoods. Robbery and shoplifting were lower among participating stores, and even greater among stores with higher compliance with recommended components.

Another study evaluated whether the adoption of Universal Precautions (UP) or Body Substance Isolation (BSI) resulted in decreased needle recapping (risk factor) or injury rates among critical care nurses. Findings suggest that UP and BSI did not have significant impact on healthcare workers' greatest source of exposure to blood borne pathogens. We were not able to calculate effect measures for this study.

*We found insufficient evidence for the effect of administrative controls at short and medium‐term follow‐up* (Table 5).

##### 6.1.2.7 Summary of structural measures

The results overall show that effect sizes of the structural measures range from modest to strong effects, apart from legislation and enforcement that had somewhat lower effect sizes overall (Table 5, narrative analyses, and Table 6, meta‐analyses). This contrasts individual level safety interventions that mainly provided no to little effects overall. Structural safety interventions yielded higher effects when safety interventions were independent of human decision making. This is well in accordance with theoretical knowledge related to the PHHHC.

#### 6.1.3 Multifaceted safety interventions

Multifaceted safety interventions integrate different types of components at the individual level, group or organizational level, or across these levels. In this review, multifaceted safety interventions is when more component are combined to change the immediate object of the intervention, such as the use of PPE, cleaner and tidy workplaces, and improved safety behavior. In the following, we present the three types of multifaceted safety interventions that we have included, and summary tables of results are presented below (Table 7, narrative analyses, and Table 8, meta‐analyses of multifaceted safety interventions).

**Table 7:** Summary of narrative analyses of multifaceted safety interventions, *not included* in meta‐analysis, by quality assessment, level of evidence and estimated strength of effect

Number of safety interventions	Quality assessment				Level of evidence	Strength of effect
Type of safety intervention and follow‐up periods	High quality	Moderate quality	Low quality	Total	RCT, CBA, and serial measures (ITS)	RCT, CBA, and serial measures (ITS)
**3.0. Multifaceted safety interventions:**						
**3.1 Multifaceted at the individual level**	**1**	**5**	**2**	**8**		
2 = Short‐term (−12 months)	1	2	1	4	Moderate	None
3 = Medium‐term (12–36 months)		3	1	4	Limited	**Little to moderate**
**3.2 Multifaceted at the group or org. level**	**2**	**4**	**3**	**9**		
3 = Medium‐term (12–36 months)	2	1	2	5	Moderate	Strong
4 = Long‐term (36‐month)		3	1	4	Limited	Moderate
**3.3 Multifaceted across individ. and org. levels**	**5**	**4**	**4**	**13**		
3 = Medium‐term (12–36 months)	3	2	1	6	Strong	None
4 = Long‐term (36‐month)	2	2	3	7	Moderate	Strong
**3.9 Multifaceted safety interventions, other**			1	1		
4 = Long‐term (36‐month)			1	1	Insufficient	*Not estimable*

Abbreviations: CBA, controlled before‐and‐after; ITS, interrupted time series; RCT, randomized controlled trial.

**Table 8:** Summary of meta‐analysis for a subset of multifaceted safety interventions, by quality assessment, level of evidence and strength of effect

Number of safety interventions	Quality assessment				Level of evidence	Strength of effect	Meta‐analysis (injury outcomes)		
Types of safety interventions and follow‐up time points	High quality	Moderate quality	Low quality	Total	RCT and CBA	RCT and CBA	RCT	CBA	*I* ^2^, RCT/CBA
**3.0. Multifaceted safety interventions:**									
**3.3 Multifaceted across levels**	**2**	**1**	**2**	**5**					
2 = Short‐term (−12 months)	2	1	2	5	Moderate	None to moderate	**OR: 0.88 [0.67, 1.17]**	**OR: 0.62 [0.40, 0.96]**	NA/74%

Abbreviations: CBA, controlled before‐and‐after; *I* ^2^, Heterogeneity; NA, not applicable; RCT, randomized controlled trial.

##### 6.1.3.1 Effectiveness of multifaceted safety interventions at the individual level (Type 3.1)

This type of safety intervention combines components directed at the individual level, such as, attitude modification and behavioral modifications. We identified eight studies using combinations of components at the individual level (Table 7).

Safety interventions combining attitudinal and behavioral components seem to point in the direction of a small non‐significant effect. It seems that this type of safety intervention follows the results we found for safety interventions directed at the individual level. There is moderate evidence that combining various approaches at the individual level has little effect on improving safety or reducing accidents at work.

We included one RCT study and six CBA studies, representing eight safety interventions. Two safety interventions used RCT study design, but did not provide significant results on safety interventions combining both attitudinal and behavioral components. The CBA studies indicated some effect, but only one study demonstrated significant results.

*We conclude that multifaceted interventions at the individual level have moderate evidence of no effect at short‐term follow‐up, and limited evidence of little to moderate effect at medium‐term follow‐up on reduction of accidents and improvement of safety* (Table 7).

##### 6.1.3.2 Effectiveness of multifaceted safety interventions at the group/organizational level (Type 3.2)

We identified nine studies (two CBA and seven studies using serial measures) that evaluated multifaceted safety interventions directed at the group or organizational level. The two CBA studies combined interventions of consultation, advice and feedback, and sanctions in a group of mixed industries and in a hospital. Both of these studies had serious RoB.

The multifaceted interventions using serial measures combined various combinations of intervention components. One study combined skills training, workers' compensation premium rate reductions and administrative procedures at company level to encourage companies to maintain safety in the logging industry (Bell & Grushecky, 2006). Four studies combined engineering and administrative controls and sometime including training in use of new devices (Chhokar et al., 2005; Fujishiro et al., 2005; Gershon, 1999; Passfield 2003). The last study combined safety training, administrative controls, employee participation and feedback in an industrial setting (Park et al., 2009).

Effect sizes in the individual studies range from little to strong effect of the intervention, and thus the heterogeneity in outcomes is substantial, indicating that differences between studies are not due to pure sampling error. These differences can be related to the variation in combinations of components in a safety intervention when multifaceted approaches are evaluated, but also differences in study populations, as nurses, cleaners and industry workers are represented in various degrees in the interventions. Multifaceted components using engineering controls provide higher effects.

*We conclude that safety interventions combining group or organizational level components provide moderate evidence of a strong effect at medium‐term follow‐up, and limited evidence of moderate effect at long‐term follow‐up* (Table 7).

##### 6.1.3.3 Effectiveness of multifaceted safety interventions across levels (type 3.3)

Multi‐level safety interventions integrating individual level and group or organizational level components are the largest group of multifaceted safety interventions, including two RCT studies, five CBA studies, whereof two studies used risk and behavior as outcomes. Furthermore 13 studies using serial measures were included in this group of safety interventions, all using injury as outcomes.

Two CBA studies evaluating training and administrative controls or engineering controls provided significant and non‐significant effects on injuries in health care and grocery store workers, respectively. The three other CBA studies found significant effects of combining individual and organizational level components, mainly safety training, worker involvement, engineering controls and administrative controls. The two RCT studies (Peek et al., 2004); Srikrajang et al., 2005) provided borderline and significant results, respectively, of the safety interventions. In the studies using serial measures, the effectiveness of interventions that included more than one component across levels was more likely for those with an engineering or administrative control. The heterogeneity was high for safety interventions at medium‐term follow‐up, but not for interventions at long‐term follow‐up. Overall, the multifaceted safety interventions using serial measures were favored over the control, apart from one study.

Meta‐analyses were performed on evaluations of multifaceted safety interventions across levels using RCT and CBA study designs with injury outcomes. We found moderate evidence of none to moderate effect of the multifaceted safety intervention, as only the CBA studies provided significant effect (Table 8). The heterogeneity for the CBA studies was moderate (*I* ^2^ = 74% for short‐term follow‐up studies). The single RCT study did not provide a significant effect (Table 8). For the serial measures we judged a strong evidence of a moderate effect of multifaceted safety interventions across levels at medium term follow‐up, and moderate evidence for a strong effect at long‐term follow‐up. One serial measure provided a lower estimate, and the components of the interventions differed somehow, as it mainly consisted of a multifaceted Drug Free Workplace program that varied from the other components combined in this group of multifaceted safety intervention studies. If we excluded this latter study in the evaluation, then the heterogeneity became lower and the effect sizes higher and statistically significant.

*We conclude that there is moderate evidence that safety interventions combining individual level and group or organizational level components have none to moderate effects at short‐term follow‐up* (Table 8); *strong evidence for a moderate effect at medium‐term follow‐up; moderate evidence for a strong effect at long‐term follow‐up* (Table 7).

##### 6.1.3.4 Summary

Based on the studies reviewed, the greatest effects were achieved with safety interventions directed toward the group or organizational level, whereas safety interventions directed at the individual level provided smaller effect sizes overall. For the latter group of safety interventions there was in general limited evidence for an effect of the safety interventions. In particular, evidence is strong that safety interventions based on engineering controls, alone or in combination with other types of interventions, were more effective than the other approaches.

These results of the relative effectiveness of the main types of intervention are well in accordance with the Public Health Hierarchy of Hazard Control (Castillo et al., 2006; Herrick & Dement, 1994).

### 6.2 Overall completeness and applicability of evidence

To capture the most relevant studies, search criteria were built to allow for a high degree of sensitivity. A broad number of databases were searched as were other sources, such as homepages of relevant institutions to identify appropriate gray literature. Additionally, we asked a group of experts in the field to provide relevant intervention studies in the safety science field (“must have studies”). We identified 60,666 studies that were reduced to 196 studies after the screening procedure. We further excluded 85 single group studies from the evaluation of the effects of safety interventions. The remaining 100 studies include 16 studies with an RCT design, 30 studies with a CBA design, and 54 studies using serial measures. The 16 RCT studies included 20 study arms; the 30 CBA studies included 43 study arms, and the 54 studies with serial measured included 58 study arms, thus providing 120 study arms in total. The single group studies were not considered for further analyses of effectiveness.

Of 37 suggestions for “must have” studies identified by experts, 23 of those met the inclusion criteria, and we successfully retrieved 21 studies (90%) with the search strategy. As the sensitivity of the search strategy is high, we consider relevant studies in the field were likely to be well captured and sufficient to answer the research questions. Obviously, the specificity of the search was quite low (about 0.4%).

The 100 included studies covered all high‐risk industries. Half of the studies were in large firms or public institutions, such as hospitals, social and health care institutions, but all classes of firm size were represented. The review covered all main types of safety interventions, and all specific types of safety intervention components as pre‐specified in the review protocol (Section 13.3). We did not pre‐specify safety interventions using physiological modifications, but included these when they appeared in the material. Some intervention components were only evaluated in a few studies, which are reflected in their level of evidence assessments.

While the review did not focus on prevention of any specific types of accidents or accidents, a wide variety were included in the review. An “accident” is the causal event(s) leading to the harmful exposure of an individual. Thirty‐eight percent (*n* = 46) of the studies assessed the effects of safety interventions for all types of accidents. The more common groups of accidents included “contact with sharp or pointed materials or tools” (19%) and “acute overexertion events” (16%), which are recognized as common accidents at work. Only one included study investigated the effect of safety interventions on accidents related to “contact with electrical voltage.”

“Accidental injury” is the consequence(s) of an accident. The largest groups of accidental injuries were “needlestick injuries” and “overexertion injuries.” Only one included study investigated the effect of safety interventions on “burns, scalds and frostbite” injuries. Four studies evaluated the effect of safety interventions on fatal accidents (3%), and the remaining studies evaluated the effect on non‐fatal accidents (50%), or events of mixed severity (44%). Most studies used “injury” as the outcome measure (82%); risk and behavior were used as the other main outcome measures (16%), and the remaining 2% used other types of outcome measures, such as violent crime and assault rates.

To be sure to cover all relevant types of safety interventions, our initial inclusion criteria included randomized controlled studies (RCT), controlled before and after studies (CBA), studies using serial measures even if they did not meet stricter criteria for ITS, and single before and after study designs. The simple before and after studies, which are recognized to have validity limitations, did not provide any new types of safety interventions not captured in the other designs; therefore we did not include these in the analyses. The studies using serial measures included important types of interventions, such as legislative changes and enforcement, engineering controls, and administrative controls, which were not well covered by RCT and CBA study designs. We included the serial designs using narrative analyses. While we provided point estimates where applicable, we did not include them in the meta‐analyses.

The review covers all sectors where workers are at high risk for work accidents, apart from fishing and offshore. Furthermore, all main types of safety interventions are included that reflect, to our knowledge, the current practices in the prevention of accidents at work. All relevant types of accidents and types of injuries are covered by this review, but only two studies covered safety intervention evaluating accidents related to “contact with high electrical voltage” and injuries related to “burns, scalds, and frostbite.” Accordingly, we judge that the review covers relevant types of participants, safety interventions, and relevant outcomes, and that the external validity is high and relevant for practice.

### 6.3 Quality of the evidence

The most frequently used design to evaluate safety interventions was serial measures (ITS) (*n* = 57), followed by controlled before and after studies (CBA) (*n* = 43). Only a smaller part of the safety interventions were evaluated used RCT designs (*n* = 20). The ITS design is mainly used for studies with long follow‐up periods (85%), such as legislative changes, multifaceted safety interventions and engineering controls. RCT designs are mainly used for safety interventions using shorter follow‐up periods (75%), such as attitude modifications, behavior modifications, or needlestick injuries in hospitals. CBA studies are placed in‐between these two designs, and mainly used in evaluations of safety interventions with shorter follow‐up periods (47%) and medium‐term follow‐up periods.

We assessed the level of evidence by using a modification of the methodology suggested by Tompa, Trevithick, et al. (2007). Even though we followed the overall methodology suggested by Tompa, Trevithick, et al. (2007), we did not use their suggested quality criteria, but used the RoB assessment following the recommendation of the Cochrane handbook (Higgins & Green, 2008) for RCT and CBA study designs (Risk of Bias table, chapter 12.5). For the serial measures we used the seven‐standard RoB criteria for ITS studies based on the Cochrane Effective Practice and Organization of Care Review group (EPOC, 2016). The level of evidence for the effect of a safety intervention thus takes into consideration both the included numbers of safety interventions and the RoB of these safety interventions.

We judged a safety intervention to have *strong evidence* if it was supported by a minimum of three studies with high‐quality (low RoB) reporting of consistent findings. We judged a safety intervention to have *moderate evidence* if it was supported by at least two high‐quality studies, or three studies of medium and high quality, with consistent findings. We judged a safety intervention to have *limited evidence* if it was supported by at least one high‐quality study, or two studies of medium and/or high‐quality, with consistent findings. If findings from medium and high quality studies did not have consistent findings, we judged that there was *mixed evidence* for the safety intervention. If a safety intervention was only supported by one moderate quality study or any number of low quality studies, we judged the intervention to have *insufficient evidence*. Findings were considered to be consistent when the majority of point estimates from various studies favored the intervention, or favored the control.

In cases where we conducted meta‐analysis we did not use the “mixed evidence,” but calculated the pooled effect estimate of a particular outcome.

About 32% of the evaluated safety interventions were judged as high quality studies, 27% medium quality, and 42% low quality studies (Table 9).

**Table 9:** Number of included safety interventions, by level of quality and study design

Number of safety interventions				
	Study design			
Level of quality	RCT	CBA	Serial measures (ITS)	Total
High quality	11	13	14	38
Moderate quality	5	9	18	32
Low quality	4	21	25	50
total	20	43	57	120

Abbreviations: CBA, controlled before‐and‐after; ITS, interrupted time series; RCT, randomized controlled trial.

The quality of studies was used as a basis for establishing the level of evidence for each type of safety intervention. The levels of evidence varied from insufficient level of evidence to a strong level of evidence, depending on the type of safety intervention being considered.

For the group of safety interventions included in meta‐analysis the assessment of level of evidence showed that safety interventions directed at the individual level, such as counseling approaches, teaching and education, and individual physical training had insufficient and limited evidence for the findings. Moderate evidence was found for enforcement of laws and regulation at medium‐term follow‐up, as well as for multifaceted safety interventions across levels at short‐term follow‐up. We found strong evidence for engineering controls at short‐term follow up (Tables 5 and 6, previous section).

For safety interventions evaluated with serial measures and RCT and CBA study designs, where we judged that meta‐analysis were not possible, we identified, by and large, the same picture regarding level of evidence as with the meta‐analysis results. Interventions directed at the individual level, such as counseling approaches, teaching and education, and individual physical training had insufficient and limited evidence for the findings. Only individual feedback and coaching at short‐term follow‐up had moderate evidence for the findings.

Interventions directed at the group or organizational level had somewhat higher levels of evidence for the findings. Strong evidence for the effect was found for: engineering controls at medium‐term follow‐up; multifaceted interventions at medium‐term follow‐up. Moderate evidence of the findings was found for: enforcement of laws and regulations at long‐term follow‐up; multifaceted interventions at the individual level at short‐term follow‐up; multifaceted interventions at the organizational level at medium‐term follow‐up; and multifaceted interventions across levels at long‐term follow‐up.

Limited evidence was found for: soft regulation, economic incentives and legislative changes. For soft regulations and economic incentives there were few studies. The main reason for the limited evidence of legislative efforts was that attribution of declining accidents and fatalities to legislation is not consistently demonstrated in these studies. The inability to establish better evidence for this type of safety intervention may be due to methodological constraints and history surrounding legislative efforts. Insufficient evidence was found for social marketing and administrative controls.

The robustness of effect sizes for the various types of safety interventions was investigated, where possible, by removing studies with high RoB, and see changes in effect estimates. Also, in cases where review authors judged that outcomes could not be attributed to the safety intervention, we excluded them in the effect size calculations.

Inevitably, various safety interventions brought together in a systematic review will differ due to differences in participants, included components and outcomes, RoB, and study designs. The heterogeneity of the effects is particularly high for multifaceted safety interventions, where different numbers and types of safety intervention components are combined. In these cases the heterogeneity ranges from 73% to 90%, apart from long‐term follow‐up of multifaceted safety interventions across levels, which had a much lower heterogeneity (*I* ^2^ = 16%).

### 6.4 Potential biases in the review process

We sought to minimize bias in study selection and data extraction by developing a coding book that we tested before launching the project. In particular, the type of safety interventions can be difficult to determine, and we developed a detailed guideline to assist coding personal and researchers. A challenge was to distinguish between acute accidents, and injuries from chronic and long‐time exposure in the workplace, as reports are not always clear about this. The review team assigned two experts with medical knowledge to assist in the evaluation of such cases, in particular cases related to overexertion injuries (KRA and HJL). The team also had members with expertise in biological and chemical exposure, who assisted in the coding and classification of studies relating to poisoning, chemical burns etc. (KBF and KRA). However, in some cases acute and chronic exposures were mixed in the studies, and we could not fully rule out misclassification in these studies, even with the precautions taken. Concerns are greatest surrounding acute musculoskeletal injuries, which may have resulted from longer‐term exposures. It is of note that the absolute classification of these events remains problematic in clinical medicine as well.

The number of studies included and the time and resources at our disposal to carry out the review did not allow for contacting authors of original papers. This implied that in a few cases we did not have sufficient information on a study that may have been possible to obtain from the authors. Some members of the review team had own original studies included in the review. To avoid unintended bias, authors were never assigned to screen their own papers. Before sending out a packet of studies for screening, the administrator checked whether any of the reviewers had a study included in the packet (searching their name in the Reference Manager file).

To incorporate studies using serial measures, which provide the only source of information for some interventions such as legislation, we took a systematic, grounded approach to their review. Rather than requiring more stringent, specific criteria for inclusion of ITS studies, we chose to assess how investigators justified their approach to design and analyses based on the context in which they were working. We specifically sought to identify measures taken to improve internal validity of studies. Each study was assessed for reasonable statistical inference, as well as an overall appropriate inferential process. Two reviewers independently assessed each study, and then discussed the studies where differences were noted, or disagreements existed. We found the process to be not only useful, but also enlightening, as we learned from each other's perspectives. Given this approach was new, we may have failed to extract points others will find of relevance at a later date that could have influenced our assessments of these studies.

We sought to provide an overall assessment for each of the main types of safety interventions, to aid stakeholders in making informed decisions when considering safety interventions in the workplace. It was not possible to carry out meta‐analyses on all of the different types of interventions, as the content of the interventions, the context and the participants differed too much. Furthermore, the data we present for most intervention types are from limited sources, and assessment of publication bias was not always possible. Given the breadth of the field of occupational safety, these issues are not surprising.

### 6.5 Agreements and disagreements with other reviews or studies

#### 6.5.1 Methods used to assess agreements or disagreements with other reviews

We know of no other review that has addressed effectiveness of occupational safety interventions across broad classifications of interventions as we did here. However, other investigators have conducted reviews that cover various components of our review. To capture these, we systematically searched for relevant reviews by using the same search methods as described in Section 4.2 for original studies for this review. In total we found 2894 reviews. We restricted our search to include only “systematic reviews,” and we identified 459 systematic reviews. These reviews were then screened with the methodology described in Section 4.3.1 and Supporting Information Appendix 12, for original studies. After screening of these reviews we excluded 420, leaving 29 relevant reviews. We then conducted a quality assessment of these 29 reviews using the AMSTAR methodology (Shea et al., 2007), and excluded eight reviews with a quality score below four. From the remaining 21 reviews we extracted key information about interventions and effects. The types of interventions included in the selected reviews were then coded by using the classification in Section 12.3 in Supporting Information Appendix 12.3. In the following, we discuss whether there are agreements or disagreements between the present reviews and other reviews for each of the main types of interventions. We have additionally included two studies of non‐published working papers from the National Bureau of Economic Research (Gray & Mendeloff, 2002; Gray & Scholz, 1991) that provide in‐depth accounts of previous work on the effect of enforcement.

#### 6.5.2 Comparisons to our findings

##### 6.5.2.1 Effectiveness of attitude and belief modification

Five previous reviews looked at the effects of attitude modification on accidents at work. In general, they found no evidence or weak evidence for the effectiveness of interventions aimed at attitude modifications in preventing occupational injuries. Attitude modification by means of educational efforts has been found to be ineffective in general (Robson et al., 2012). Likewise, two reviews of the effectiveness of back school programs in preventing back injuries found limited evidence that education is not effective in preventing back injuries (Hogan et al., 2014; Van Poppel et al., 1997). Although slightly more positive findings have been seen for farm safety educational programs (DeRoo & Rautiainen, 2000), and educational efforts aimed at preventing falls in the construction industry (Rivara, 2000), methodological limitations and inadequate study designs restrict the conclusions that can be drawn from these latter two reviews.

The results from these reviews thus support the findings of this review, in which attitude modification has a weak link to the improvement of safety and behavior and to reduction of accidents at work. While these previous reviews found limited evidence for no or little effect of attitude and belief modification, the present review found insufficient evidence.

##### 6.5.2.2 Effectiveness of behavior modification

Reviews of behavioral interventions to prevent injuries have generally found weak, insufficient or no evidence of effect. Reviews on training interventions have shown either no effect in preventing nonfatal injuries in the construction industry (Molen et al., 2012), or insufficient evidence regarding training in general (Robson et al., 2012), or in reducing injuries among HCW due to violence (Runyan et al., 2000).

Insufficient evidence was also found for the use of drug and alcohol testing of professional occupational drivers for preventing injuries (Cashman et al., 2009), although a later review, not limited to occupational drivers, concluded that the evidence of the effectiveness of drug testing is weak in general, due to poor quality research. However, some evidence for the efficacy of random alcohol testing in the road transport industry was identified (Pidd & Roche, 2014).

The more classical BBS interventions, based on monitoring of behavior and feedback, have also shown limited effect and methodological problems. Although a meta‐analysis of BBS interventions in the workplace revealed a statistically significant reduction in injuries after conducting a BBS intervention, the authors recommended that the results should be interpreted with caution, due to the poor marginal methodological quality of studies included in the meta‐analysis (Tuncel, 2006). Similarly, a review of interventions for the prevention of eye injuries found that a low quality study using behavior modification techniques to improve the use of safety equipment showed a non‐significant decrease in injury rates (Shah et al., 2009), while another review based on two studies concluded that behavioral interventions had an effect on eye injuries in manufacturing settings (Lipscomb, 2000).

Overall, the results of these reviews are consistent with the present review. BBS interventions, such as monitoring of behavior and feedback, have also shown limited effect in these studies, and methodological problems in the studies is also a limitation. Random alcohol testing among drivers, as a BBS intervention approach, indicated some effect in road transport industry, and thus provided some evidence of this type of behavior based approach.

##### 6.5.2.3 Effectiveness of physiological modification

Physiological modification is a safety intervention that works either by exercising and thus increasing the flexibility and strength of the worker's body, or by using different forms of personal supportive equipment. The evidence for the effectiveness of personal protective or supportive equipment is mixed, and dependent on the type of equipment and injury.

A review of interventions toward physical training related injuries primarily within a military setting found strong evidence for mouth guards, semi rigid ankle braces and synthetic socks as effective interventions (Bullock et al., 2010). Two other reviews looked at the effectiveness of lumbar supports in the prevention of back injuries among hospital workers, and among managers and shop floor workers in industry. These two studies identified the same original studies, and reached the same conclusions, that there is no conclusive evidence for or against lumbar support (Hogan et al., 2014; Van Poppel et al., 1997).

The idea of physical training or exercise as a way to prevent accidents is relatively new in an occupational context, thus not many studies have been carried out. Therefore, the reviews in this area often end up being inconclusive. Mixed findings and low quality studies led to inconclusive evidence in a review of stretching to reduce work‐related musculoskeletal injuries (Costa et al., 2008). Likewise, no firm conclusion could be drawn on the possible effectiveness of multifaceted physical training programs in preventing anterior cruciate ligament injury, although moderate evidence was found among female athletes engaged in team sports (Stojanovic & Ostojic, 2012). However, one review found strong evidence for agility‐like training and prevention of overtraining as prevention approaches toward physical training related injuries (Bullock et al., 2010).

The reviews thus support our findings to some extent, but with a more mixed level of evidence. There seems to be some effect on athletes in relation to overtraining that are included in the present review. However, the latter seems to cover injuries in professional sports, and thus is not similar to the occupational groups that we have included in the present review. In this sense it does not directly contradict our findings concerning the effects of modification of employee physiology in a workplace setting.

##### 6.5.2.4 Effectiveness of structural modifications

Structural interventions can either be initiated at the company level or at the societal level, and can be either “hard,” as is the case with legislation and engineering controls, or “soft” as is the case with economic incentives, corporate social responsibility, industry agreements and other more voluntary approaches. Generally the reviews evaluating the effectiveness of structural changes have found mixed results at both the societal and company level. However, there is a tendency that the results are more positive the “harder” the intervention.

###### 6.5.2.4.1 Effectiveness of legislation and enforcement

At a societal level a review found low to very low quality evidence that workplace inspections decrease (accidental) injuries in the long term, but not in the short term (Mischke, 2013). A review by Tompa, Trevithick, et al. (2007) demonstrated limited to mixed evidence that inspections offer general and specific deterrence, and that citations and penalties aid general deterrence; and strong evidence that actual citations and penalties reduce injuries. Another review limited to the construction industry found no evidence that introduction of regulation, inspections or the introduction of occupational health services is effective in preventing non‐fatal and fatal injuries (van der Molen et al., 2012). Slightly more positive results were found in a review on fall prevention in the construction industry, where some evidence was identified that regulations with enforcement may decrease falls. However, this was based on a single low quality study (Rivara et al., 2000). Yet more positive result were found within agriculture, where legislation focused on either the use of roll‐over protection on new tractors, or banning of pesticides; both of which were found effective in decreasing accidents (Rivara et al., 2000).

Two (non‐published) working papers (not systematic reviews) from the National Bureau of Economic Research (Gray & Mendeloff, 2002; Gray & Scholz, 1991) addressed similar issues. Gray and Scholz (1991) reported effects of OSHA inspections in decreasing injuries in the period 1979–1985, but they did not examine effects of penalties. Gray and Mendeloff (2002) subsequently reported declining effects of OSHA inspections over time, particularly in the last period they examined, 1992–98, which is not consistent with the more recent, published findings in the US. In line with this we found small but consistent effect sizes for enforcement of legislation.

In contrast, the included Canadian study by Hogg‐Johnson et al. (2012) designed to assess effectiveness of regulatory enforcement and consultation (rather than citations and penalties) did not demonstrate any effect. Possible explanations include differences in policy enforcement across countries, as well as methodological differences in the evaluations. Another important issue was that the study ignored any general effects of the health and safety legislation, that is, the threat of inspections and control on yet uninspected workplaces, and consequently bias would be toward the null.

All in all, other reviews present somewhat mixed evidence on the effect of legislation and enforcement. Introduction of legislation seems to provide some effect, even though the assessment of the level of evidence differs from review to review. This is in line with our conclusions that legislative effectiveness is highly contextual, including evidence that under certain conditions (such as in the face of little evidence base for ruling) legislation does not work. The sources of the mixed results were only revealed through careful narrative review. Quite consistent with our report are the reviews that point to small but consistent effects of enforcement, particularly when penalties are introduced.

###### 6.5.2.4.2 Effectiveness of economic incentives

Financial incentives from insurance companies were found to be effective in reducing injury rates (Rautiainen et al., 2008). Another review focused specifically on the prevention incentives of insurance and regulatory mechanisms, and found that there was moderate evidence that the degree of experience rating reduces injuries (Tompa, Trevithick, et al., 2007). These reports contrast to the lack of clear evidence we observed. It is of note, that we did not capture all of the studies included in these two other reviews.

###### 6.5.2.4.3 Effectiveness of administrative controls

Mixed results have also been identified related to structural changes at the company level. One review concluded that policy changes had an effect on eye injuries in manufacturing settings (Lipscomb, 2000), while another deemed the results of administrative approaches' effect in reducing the risk of violence inconclusive, due to low quality studies (Runyan et al., 2000). The same conclusion was reached in a review of the effectiveness of introducing an OSH management system, where the evidence was insufficient to make recommendations either in favor of or against these systems (Robson et al., 2007). Within health care, one review found that a hands‐free passing technique (policy) in the clinical setting had no effect on surgeon scalpel injuries (Watt et al., 2010).

Our results were assessed based on only one study, and as such our conclusions are not inconsistent with these reports of mixed results. We suspect that the effectiveness of administrative controls is likely to be highly contextual, as is legislation. For example, such controls that are enforced in the workplace would likely be more effective than those that are not, as would ones put in place with a reasonable level of supporting evidence.

###### 6.5.2.4.4 Effectiveness of engineering control

One review found that blunt suture needles reduced needlestick injuries related to surgical procedures, and concluded that engineering control research would produce the most effective preventive approaches (Rogers & Goodno, 2000). Two other reviews, also within the hospital sector, showed that protective equipment, such as surgeons using either cut‐resistant gloves (Watt et al., 2010) or double gloves (Rogers & Goodno, 2000), are effective in preventing scalpel and needlestick injuries.

These reviews are well in line with the conclusion of the present review, and we consider they support the findings of this review that engineering controls provides an effective approach to improve safety and prevent accidents at work. However, the present review also included other types of engineering controls not covered by previous reviews, and potentially disagreement could not be determined for these types of safety interventions.

##### 6.5.2.5 Effectiveness of multifaceted safety interventions

Multifaceted interventions are often more complex and less comparable across studies than other types of interventions. Thus, reviews of these types of interventions seldom aggregate across studies due to the great heterogeneity between studies. Overall, mixed results have been found in previous reviews, although most reviews demonstrate a positive effect of the multifaceted safety interventions (DeRoo & Rautiainen, 2000; Lipscomb, 2000; van der Molen et al., 2012).

Other reviews have found no evidence that multifaceted educational interventions are effective in decreasing injury rates among agricultural workers (Rautiainen et al., 2008), or that regional safety campaigns consisting of a contest and inspections have any effect on nonfatal injuries within construction (Molen et al., 2012). These multifaceted safety interventions are mainly combining components at the individual level, and their conclusions are in line with the present review. Safety interventions combining components at the individual level have no or limited effect on the prevention of accidents at work.

Reviews have identified evidence of efficacy of multifaceted interventions across levels, including safety campaigns within farm safety (DeRoo & Rautiainen, 2000), eye injuries in manufacturing (Lipscomb, 2000), and on non‐fatal injuries within the construction industry (van der Molen et al., 2013).

## 7 AUTHORS' CONCLUSION

### 7.1 Implications for practice

In this review we assessed the effectiveness of broad classifications of safety interventions intended to reduce accidents at work. Through this process we sought to provide information that would allow more informed decision making as resources were allocated for intervention efforts to reduce accidents at work. Such information should be useful to business owners, worker representatives, OSH experts, authorities and policy makers.

Findings provide strong evidence that greater effects are achieved with safety interventions directed toward the group or organizational level rather than those directed at individuals. In particular, safety interventions based on engineering controls are more effective at reducing injuries than other approaches; this is particularly the case when the engineered intervention can be introduced without requiring decision‐making on the part of exposed workers. In other words, engineering interventions that directly remove the hazard for physical injury seem to be more effective than ones that require workers to choose to use the intervention. An example of the former would be safer syringes with retractable needles, when all older devices are removed from the workplace (Smollen, 2004).

In contrast, patient lift equipment can reduce biomechanical loads among hospital staff that cause acute musculoskeletal injuries, but workers have to decide whether they will use them or not, and how often (Alamgir, 2008; Schoenfisch et al., 2013). Furthermore, injury reductions would be expected sooner when the “decision‐to‐use” is removed, thus alleviating choices regarding adoption of the intervention. Multifaceted approaches that combined intervention elements on the organizational level or across levels provided moderate to strong effects, in particular when engineering controls were integrated with other components. It should also be noted, that engineering controls are not always possible to introduce for all types of workplace accidents, and thus other approaches need to be introduced.

More modest effect was observed for safety climate interventions using techniques such as feedback or leadership training to improve safety communication. Only one study assessed injuries as the outcome, and these were non‐serious events (Zohar, 2002). Furthermore, follow‐up time was short, with insufficient evidence of any longer‐term effects for this classification of interventions.

Safety interventions directed at the individual level, in general, provided no effect or only very modest effect on the outcomes. Safety campaigns, teaching and introduction to safety had no effect without any other simultaneous intervention efforts. We caution that this does not mean that workers should not be trained in how to do their jobs safely. In fact, in most jurisdictions this is required by law. However, such training efforts should not be expected to result in reduced injury rates in the absence of other more effective efforts. There is some evidence of effect with more intense counseling approaches, such as group discussions; these studies were also limited to short‐term evaluation.

We found reasonable evidence demonstrating that regulation can contribute to the prevention of accidents at work, but with lower effect sizes. As legislation/regulation often applies to broad groups of workers, population‐based effects can potentially be quite large even in the face of relatively low effect measures such as those we observed. Regulation often involves a lengthy process, and negotiations are common among stakeholders during that process. The final product is not guaranteed to include the original provisions designed to promote safety. Thus, it is not at all surprisingly that we also found evidence that in some cases, regulation does not work, specifically, when legislative requirements were passed with little evidence base, and for which enforcement would be unlikely to substantively affect injury outcomes. In the process of promulgation of any legislation or regulatory effort this is worth bearing in mind.

The example presented by Marlenga et al. (2006) demonstrates a rule that made little sense by the time it was approved; after negotiations, the legislation failed to address what was known about the problem of tractor accidents in teens. The fact that no evidence of effect was found is not surprising, and if an effect had been found it would have been hard to attribute the changes to the legislation.

In contrast to legislative effects, the evidence that enforcement activities can work was more consistent, but with smaller effects. While legislation theoretically could have effects without enforcement, it seems reasonable that longer‐term effects would be enhanced by enforcement.

Overall, the results of this review support careful attention to the Public Health Hierarchy of Hazard Control when considering workplace safety interventions. This model which shares public health and engineering safety principles emphasizes that more effective preventive measures involve eliminating risk at the source or separating workers from hazards. Efforts delivered at the level of the group or organization are more likely to be effective than individual level measures. A focus on training workers to deal with dangerous tasks, for example, use of personal protective equipment, should be a last resort, exercised only when other more effective measures are not feasible. However, it should be recognized that such efforts enacted alone are unlikely to effectively prevent injuries. In short, occupational safety intervention efforts should foster safer work environments, tools, and conditions rather than focusing on how workers can mitigate the risks.

### 7.2 Implications for research

The ultimate goal of occupational injury science is the long‐term prevention of workplace injuries. This goal is supported through epidemiologic studies that define causes of injuries, development of control measures, and evaluation of effects. However, to be most useful, evaluations need to provide more information than simply if an intervention worked or not; they should also enlighten us as to why that is the case, and under what conditions this is likely to be true.

#### 7.2.1 Improving the conduct of injury intervention research

Basic epidemiologic principles inform us of the importance of clear definitions of outcomes and exposures (in this case the interventions) in discerning effects. Interventions that focus on a specific problem with a firm evidence base may be easier to develop, enforce, and consequently demonstrate effects. It is notable that the bulk of theories in the safety science literature are not used very well in the development or assessment of safety interventions. In this review 44% of the studies appraised the effects of interventions on the prevention of all types of work injuries. Such an approach implicitly, and perhaps falsely, implies a common cause of workplace injuries rather than exposure specific causes. This approach makes it more difficult to demonstrate effects when this is not the actual case. Such interventions might prevent some types of injuries, but this would not be apparent in analyses that included all injuries due to a type of outcome misclassification. Furthermore, these studies often lack a clear theoretical or conceptual basis for why the safety intervention should work.

We believe it is of note that we found the strongest evidence of effects for engineering solutions which focus on the proximal causes of specific types of injury. A number of other interventions with more tenuous effects on injury prevention (campaigns, safety climate, physical strengthening, or training of workers) did not share this focus, and the studies often lacked detail on exactly how the intervention being delivered should reduce injuries.

Just as clear and appropriate outcome definitions are needed, there is also a need for clarification as to how various types of safety intervention are classified, including the combination of components included in the intervention. If we are not clear about how to define a particular safety intervention, then comparison of effect measures becomes much less meaningful, and perhaps even inappropriate, from a theoretical perspective. We have suggested a classification of components (chapter 12.3), that includes the levels at which the intervention, or its varied components, are delivered. In this broad review, the classification scheme was useful in demonstrating relative effectiveness of different types of interventions delivered across varied levels (individual, group, organization). The process allowed us to identify areas where few studies have been conducted and evidence of effectiveness is limited. These areas included safety campaigns and training, behavioral based safety interventions, and interventions directed at changes in safety climate.

We hope the classification process used here will be a starting point in more clearly defining safety interventions, and we recommend consideration of this classification system in future studies. We acknowledge the ways our method could be improved, for example, legislative efforts often involve multiple components, yet we made no attempt to dissect the involved elements. Based on our findings, we would expect greater impact from legislation that included engineering controls that were enforced (e.g., legislation and enforcement combining engineering and administrative control), but this has not been assessed. In addition, some categories may include subcategories in future studies, for example, elimination and substitution should be separate from engineering controls.

RCTs have long been considered the “gold standard” for intervention evaluation studies, and yet in this review we saw increasing numbers of studies over time that used serial measures in more quasi‐experimental approaches. We think there are good reasons for this pattern. RCTs are not feasible for all questions asked in occupational injury science. Randomized trials are rarely, if ever, able to be used to evaluate legislative interventions. Laws are passed and scientists interested in assessing their effectiveness must find ways to do that. Several studies we evaluated took advantage of the opportunity to evaluate natural experiments. Furthermore, workplaces are highly dynamic settings, so much so, that the validity of randomization (designed to equalize unmeasured confounders at the outset) can be questioned, and contamination of study arms can be problematic, particularly in long‐term evaluations.

We believe these issues call for reflection on the differences in efficacy and effectiveness; the former being whether an intervention is capable of actually preventing injuries and the latter reflecting whether the intervention works in real world situations—where workers work. RCTs clearly have a role, as their strengths from experimental design are well‐recognized. However, we believe they are best suited, in large part, to studies exploring efficacy of interventions or shorter‐term effectiveness. They are not particularly useful in occupational injury science for assessment of long‐term effectiveness. Consequently, we believe there is a need to embrace observational studies more. We recognize that they are not perfect, but neither are experimental designs, particularly in some contexts.

In using longitudinal data for evaluation of injury prevention interventions, ARIMA models provide a reasonable analytical approach when the available data are robust enough (in number and precision of measurements) to allow such analyses. Most of the data from the studies we reviewed did not allow such an approach, yet several other very reasonable statistical methods were used (mathematical models using Poisson or negative binomial regression with generalized estimating equations allow control for autocorrelation) in analyses of serial measures. Some of the studies we reviewed provided clear examples of work that could have been improved through a more rigorous analytical plan. Accounting for serial dependence could be improved, and doing so would make requirements for statistical significance more conservative. However, this adjustment will not change point estimates, and is of less concern in cases where the pattern of injury rates is of primary interest, rather than simply statistical significance. Such would be the case in looking at periods when the legislation may be more effective, or when effects might wane after an initial improvement. Many of these ITS type studies do not even lend themselves to a single point estimate for inferential purposes.

Studies evaluating effects of legislation provide a good example. Legislation does not occur in a vacuum; it may be pushed forward due to devastating events, and it can follow a long rulemaking process that involves negotiation. Activities leading up to the effective date of a piece of legislation have the potential to influence changes in the workplace in advance of an officially effective date. The investigators for these reports, in large part, recognized challenges in the longer‐term evaluation of policy initiatives. To counter threats to internal validity, a variety of analytical approaches were used. The final decisions regarding utility of the interventions were often not based on a single point estimate, but rather on a more complex inferential process based on a series of analyses.

The use of internal and external comparisons adds significant strength to longitudinal analyses. External control populations identical to targeted ones can be hard to identify and access.

Use of internal comparison conditions or injuries has several advantages. The same underlying population is used, alleviating the need to control for demographic factors that may be associated with occupational injury such as age, experience, perhaps even union status; secondary data sources used in the analyses of legislation often provide a readily available source of comparison injuries. While there are well‐recognized problems in underreporting of occupational injuries, the use of the same data source is more likely to assure comparable accuracy in reporting. In this case, there may be reporting error, yet ratio measures of effect should be quite accurate, as long as the underreporting remains at the same magnitude. Selection of appropriate internal comparisons is just as important as selection of external ones. Internal control conditions should not be related to the intervention being evaluated, to allow control for overall secular trend. However, numerous efforts may be underway simultaneously, making careful consideration and knowledge of the population being studied important. Investigators facilitate assessment of overall inferential strength of their work when they provide details about the population they are studying, similarities and differences to comparison groups, and clear rationale for the selection of internal control conditions.

Future occupational intervention safety studies should strive to include the collection of measures that reflect the fidelity of interventions, and to provide this information along with the results. We often found that investigators reported on the delivery of an intervention, but provided no information as to whether the intervention was adopted by the target population. We believe it is a fallacy to assume that this is always the case. It is strongly recommended that information on both the delivery and the adoption of safety interventions are reported in intervention evaluations. When clearly reported, such information provides a basis for the evaluation of the influence of context, and can help differentiate failures in theory from failures in delivery or adoption. Such information is crucial for more informed interpretation of our results. Where applicable this can be done as a process evaluation that is also assessing the expected mechanisms through which the safety intervention is working; qualitative data can be a useful adjunct. Particularly, this is important in cases of complex interventions, and in cases where more intervention components are included.

Efforts to improve occupational safety intervention research evaluation processes should be based on theoretical approaches that clearly define the specific type of safety intervention and when, and why, an intervention should work and for what conditions it is likely to be more effective. Innovative study designs and analytical plans should be embraced when they are clearly justified and follow a strong inferential process.

To improve occupational safety intervention research through a transparent process, we recommend several things. Throughout, the adherence to basic principles that improve epidemiological studies beyond simple issue of study design will lead to stronger work, including clear definitions of outcomes of interest, exposures or conditions you are seeking to control, and the intervention being tested. We ask investigators to attempt to clearly articulate several issues including the following:

- What is the current understanding of the basic epidemiology of events of interest, specifically, what do we know about the proximal cause(s) of these injuries? What else do we know beyond proximal cause, such as what factors may influence dangerous exposure or unsafe behavior?
- What exactly is the intervention, including all necessary components? How is each of the necessary components to be delivered to the targeted audience?
- Conceptually, how do you see the intervention working, and does it have any theoretical basis? Is latency of effect expected, and if so what influences the latent period? Can these be measured (such as adoption of a new device)?
- How may fidelity of the intervention and adoption be assessed as intermediate measures? Given that interventions are sometimes triggered by untoward events, what factors lead to the decision to intervene? Could these influence observations reflecting regression to the mean, or alternatively, could they reflect reporting of injuries? Are there relevant contextual phenomena that should be considered? Help the reader understand things that influenced your research approach and that may facilitate more appropriate interpretation of results.
- What changes occurred in the research environment over time? Obviously, this becomes more important the longer an observation period is for effectiveness with well‐recognized trade‐offs between maturation effects and length of follow‐up. However, given the dynamic nature of occupational settings, changes are common, and consideration should be given to whether those that occur may influence findings.

#### 7.2.2 Improving systematic reviews in occupational safety

Whether occupational safety interventions are effective in preventing injuries is likely to be highly contextual, depending on exactly what the intervention is, how it is delivered or enforced, and upon what theoretical or epidemiologic evidence it was based on. In this review, considerable insight was gained from careful narrative review of the studies. The process was time consuming, yet important. We often found that investigators using more quasi‐experimental designs gave more attention to contextual detail that justified their approach. From our review, we found it is important to carefully consider the conceptual information provided by investigators, rather than limiting data extraction to more common review aspects, such as study design and bias.

Investigators more often than not provided some level of important contextual detail that should be considered in assessing study quality and/or in the overall inferential process. From this detailed information it is clear that the requirements that interventions must start at one point in time to be valid for inclusion in systematic reviews is fraught with problems. We believe that the better question to assess is whether the evaluation design, analytical plan, and overall inferential process took this into account. While such a requirement may make analyses and interpretation a more clear‐cut process, it is not how many safety interventions are actually delivered.

The most common RoB identified was “other potential sources of risk of bias,” where nearly half of the studies were judged to have high RoB, rather than those sources of bias that were specifically listed and assessed. These calls for a rethinking of what is important bias in relation to occupational safety interventions.

The studies reviewed here clearly demonstrate how difficult it can be to establish a clear pre‐intervention period when assessing change related to legislation. Even though legislative efforts officially go into effect at one point in time, they have the potential to influence the workplace through publicity about untoward events, negotiations in the rule‐making process, and through early adoption of anticipated regulatory changes. It is thus difficult to establish a well‐defined intervention period and a clear pre‐intervention time period. The process is not necessarily a clean one, but it is the reality in which these evaluations must be done, and that is unlikely to change. Recognizing this complexity documents the importance of observational studies, as well as a process of inferential thought that cannot be limited to statistical inference alone.

The main types of interventions evaluated were not homogeneous by any means. Legislation and enforcement activities provided a good example of this, as they all had some form of regulative component in common, but the nature of the interventions and what changes they were calling for in the workplace varied tremendously, including ones that are targeted the same industry. Intensity of regulation is not the same. The “construction falls safety standard” was based on considerable epidemiologic data, and the final rulings could be considered a multifaceted intervention, contrasting to the regulation calling for training of surface miners without any attention to direct hazard control.

Studies of fatalities have limited statistical power even in large high‐risk industries, and even when looking at a decade of data on one type of fatal injury across the US. Foley et al. (2012) and Lipscomb et al. (2003) in their studies in Washington demonstrated difficulties evaluating the effect of legislation and regulation. Even when using a large US state fund over a 10‐year period, statistical power became an issue in some of the analyses. As mentioned earlier, the final decisions regarding utility of the interventions were often not based on a single point estimate, but rather on a more complex inferential process based on a series of analyses. Yet, we believe some of the studies of legislation or enforcement, using quasi‐experimental designs and innovative analytical strategies, represent not only “best evidence” examples, but examples of studies that are about “as good as they are going to get” for addressing the questions as to whether these interventions are effective in preventing injuries at work. Our evidence base in this field needs to include such studies, but careful consideration is warranted before we go about combining single effect measures in formal meta‐analyses.

In this review, we did not find the gray literature to be useful. The quality of reports lacked scientific rigor, and none of that literature was used in our analyses. However, considerable time was spent by the review team to collect such reports and screen them. In fact, we found very little literature that had not been peer‐reviewed that was used in this report. Consideration should be given to allowing exclusion of gray literature for occupational safety intervention reviews.

Based on our experience with the current review we suggest the following considerations to improve occupational safety systematic reviews:

- A wider range of study designs and research methods, including hybrid designs, should be embraced and evaluated for their merit, based not on study design, but on an appropriate inferential process that allows for contextual considerations. Observational studies are not without limitations, but they can be more robust than sometimes portrayed if principles are carefully followed. Even when there is error in measurements, comparable accuracy over time will facilitate evaluation, and ratio measures should still be useful appraisals of effect. Transparency in the review process is just as important as having a protocol.
- Use of careful narrative review to capture important contextual detail provided by the investigative team is useful and should be utilized, which can also support the interpretation of the heterogeneity of safety interventions.
- Incorporation of process review and qualitative methods could help considerably in improving our capture of intermediate measures, and improve the inferential process about effectiveness considerably. If an intervention appears to have an effect, but it is never being adopted, it is hard to attribute change to the intervention.
- Length of follow‐up warrants careful consideration and reporting. Effectiveness of short‐term trial does not mean the same thing as effectiveness of a 10‐year old legislative standard. All intervention evaluations are not the same, and should not be treated as such. This is important in synthesizing results for practical use as well.

## 8 DEVIATIONS FROM THE PROTOCOL

### 8.1 Searches

The authors did not have access to the searches in the electronic databases EI Compendex and in business elite. Our funding did not allow us buying access to these databases.

### 8.2 Methodology

A new type of safety intervention was seen in the included studies—physiological modifications—that had not been anticipated. We included studies where modification of persons' strength and agility were sought to increase their resistance to acute exposures to the body, such as acute physical loads.

Due to limited resources, including time and finances, we did not attempt to contact authors for clarification or more exact data than presented in the manuscripts.

Publication bias was not assessed due to limited numbers of studies in each category of intervention.

## 9 CHARACTERISTICS OF STUDIES

### 9.1 Characteristics of included studies

#### 9.1.1 Included RCT studies

*The characteristics of the studies with a RCT design are listed below ordered by first author*.

**Table:** 

**Adams, JSK (2013)** *Increasing compliance with protective eyewear to reduce ocular injuries in stone‐quarry workers in Tamil Nadu, India: a pragmatic, cluster randomized trial of a single education session versus an enhanced education package delivered over 6 months*.	
Country	IN
Aim	Evaluate whether participants allocated to intensive and less intensive educational strategies differed in the incidence of detected eye injuries.
Target population	**Occupation**: Stone quarry workers
	**Industry**: Mining
	**Setting**: 100 Underground
	**Firm size**: 10–49 (small)
Study design	**RCT** with comparison conditions: Alternative type of intervention
	**Unit of analysis**: Group/organizational level
	**Sample size**: 103 workers (cases with enhanced education program; 92/103 at follow‐up). 101 workers (controls standard education program; 91/101 at follow‐up) = 204.
Type of intervention	1.1.2 Counseling approaches
Evaluation design	**Duration of intervention**: 6 months
	**Duration of follow‐up**: 2 = Short‐term (≤12 months)
	**Type of outcome measure**: Injury
Study quality	High quality

**Table:** 

**Cheng, AS (2009)** *The effect of individual job coaching and use of health threat in a job‐specific occupational health education program on prevention of work‐related musculoskeletal back injury*.	
Country	HK
Aim	To examine the effect of individual job coaching and use of health threat in a job‐specific occupational health education program in preventing work‐related musculoskeletal back injuries during manual materials handling in construction laborers.
Target population	**Occupation**: Construction work (71: Extraction and building trades workers)
	**Industry**: F—Construction
	**Setting**: 020 Construction site
	**Firm size**: Unclear/not reported
Study design	**RCT** with comparison conditions: TAU (Treatment as usual)
	**Unit of analysis**: Group/organizational level
	**Sample size**: 101 laborers intervention + 81 laborers control = 182
Type of intervention	1.2.4 Individual feedback or coaching
Evaluation design	**Duration of intervention**: 1 Half day **Duration of follow‐up**: 2 = Short‐term (≤12 months) **Type of outcome measure**: Injury
Study quality	Low quality

**Table:** 

**Daltroy, LH (1997)** *A controlled trial of an educational program to prevent low back injuries*.	
Country	US
Aim	To evaluate an educational program designed to prevent low back injury.
Target population	**Occupation**: Postal and courier activities
	**Industry**: H—Transporting and storage
	**Setting**: 060 Public area
	**Firm size**: 250+ (large)
Study design	**RCT** with comparison conditions: TAU (Treatment as usual)
	**Unit of analysis**: Group/organizational level
	**Sample size**: approximately 4000 US postal workers. Unit of analysis was the work unit.
Type of intervention	1.2.4 Individual feedback or coaching
Evaluation design	**Duration of intervention**: Not reported or unclear
	**Duration of follow‐up**: 4 = Long‐term (>36 months)
	**Type of outcome measure**: Injury
Study quality	High quality

**Table:** 

**Gadomski, A (2006)** *Efficacy of the North American guidelines for children's agricultural tasks in reducing childhood agricultural injuries*.	
Country	US
Aim	To assess whether active dissemination of the North American Guidelines for Children's Agricultural Tasks (NAGCAT) reduced childhood agricultural injuries.
Target population	**Occupation**: Crop and animal production, hunting and related service activities
	**Industry**: A—Agriculture, forestry, and fishing
	**Setting**: 030 Farming and forestry
	**Firm size**: Unclear/not reported
Study design	**RCT** with comparison conditions: TAU (Treatment as usual)
	**Unit of analysis**: Group/organizational level
	**Sample size**: 462 farms intervention + 469 farms control = 931 total
Type of intervention	1.1.2 Counseling approaches
Evaluation design	**Duration of intervention**: 40 min (mean) and range (5–90 min). Apart from that intervention was boosted with information (by mail, e.g., Postcard and safety calendar)
	**Duration of follow‐up**: 3 = Medium‐term (12–36 months)
	**Type of outcome measure**: Injury
Study quality	Low quality

**Table:** 

**Hogg‐Johnson, S (2012)** *A randomized controlled study to evaluate the effectiveness of targeted occupational health and safety consultation or inspection in Ontario manufacturing workplaces*. Study arm: Legal enforcement	
Country	CA
Aim	To determine the relative effectiveness in improving work injury claim and disability day rates of targeting firms for HSA consultation versus targeting firms for Ministry of Labour (MOL) priority inspection versus no targeted services.
Target population	**Occupation**: Mixed
	**Industry**: C—Manufacturing
	**Setting**: 010 Industrial site
	**Firm size**: Mixed firm size
Study design	**RCT** with comparison conditions: TAU (Treatment as usual)
	**Unit of analysis**: Group/organizational level
	**Sample size**: Health & Safety Association (HSA): 600, Ministry of Labour (MOL): 619, Controls: 934 = 1553
Type of intervention	2.2.7 Enforcement of laws and regulations
Evaluation design	**Duration of intervention**: 1 year (April 1, 2006 to March 31, 2007), but the specific intervention, that is, control and inspection, lasted on average 6.9 h (Mean), see Table 3
	**Duration of follow‐up**: 3 = Medium‐term (12–36 months)
	**Type of outcome measure**: Injury
Study quality	High quality

**Table:** 

**Hogg‐Johnson, S (2012)** *A randomized controlled study to evaluate the effectiveness of targeted occupational health and safety consultation or inspection in Ontario manufacturing workplaces*. Study arm: Voluntary consultation services	
Country	CA
Aim	To determine the relative effectiveness in improving work injury claim and disability day rates of targeting firms for Health & Safety Association (HSA) consultation versus targeting firms for MOL priority inspection versus no targeted services.
Target population	**Occupation**: Mixed
	**Industry**: C—Manufacturing
	**Setting**: 010 Industrial site
	**Firm size**: Mixed firm size
Study design	**RCT** with comparison conditions: TAU (Treatment as usual)
	**Unit of analysis**: Group/organizational level
	**Sample size**: Health & Safety Association (HSA): 600 and Controls: 934 = 1534
Type of intervention	2.2.3 Soft regulation
Evaluation design	**Duration of intervention:** 1 year (April 1, 2006 to March 31, 2007), but the specific intervention last 3 h on average (Mean), see Table 3
	**Duration of follow‐up:** 3 = Medium‐term (12–36 months)
	**Outcome measure:** Injury
Study quality	High quality

**Table:** 

**Jensen, SL (1997)** *Double gloving as self‐protection in abdominal surgery*.	
Country	DK
Aim	To investigate if double gloving can reduce the rate of perforation of glove barriers during abdominal surgery.
Target population	**Occupation**: Human health activities
	**Industry**: Q—Human health and social work activities
	**Setting**: 050 Health establishment
	**Firm size**: 250+(large)
Study design	**RCT** with comparison conditions: TAU (Treatment as usual)
	**Unit of analysis**: Group/organizational level
	**Sample size**: 200 pairs of double gloves (barriers) intervention + 200 pairs of gloves (barriers) control = group
Type of intervention	2.2.4 Engineering controls
Evaluation design	**Duration of intervention**: Not reported or unclear
	**Duration of follow‐up**: 1 = Post‐test
	**Type of outcome measure**: Risk or behavior
Study quality	Low quality

**Table:** 

**Jinnah, HA (2014)** *Involving fathers in teaching youth about farm tractor seatbelt safety—a randomized control study*. Study arm: Staff lead intervention	
Country	US
Aim	To assess effectiveness of involving fathers in teaching youth about farm tractor seatbelt use (treating farm safety as a family issue and building on central parental role).
Target population	**Occupation**: Farm work
	**Industry**: A—Agriculture, forestry and fishing
	**Setting**: 030 Farming and forestry
	**Firm size**: 1–9 (micro)
Study design	**RCT** with comparison conditions: No other intervention
	**Unit of analysis**: Group/organizational level
	**Sample size**: 34 parent‐led, 45 staff‐led, 35 controls = 80
Type of intervention	1.2.4 Individual feedback or coaching
Evaluation design	**Duration of intervention**: Short‐term training varied a bit by farm site
	**Duration of follow‐up**: 2 = Short‐term (≤12 months)
	**Type of outcome measure**: Risk or behavior
Study quality	High quality

**Table:** 

**Jinnah, HA (2014)** *Involving fathers in teaching youth about farm tractor seatbelt safety—a randomized control study*. Study arm: Parent lead intervention	
Country	US
Aim	To assess effectiveness of involving fathers in teaching youth about farm tractor seatbelt use (treating farm safety as a family issue and building on central parental role).
Target population	**Occupation**: Farm work
	**Industry**: A—Agriculture, forestry and fishing
	**Setting**: 030 Farming and forestry
	**Firm size**: 1–9 (micro)
Study design	**RCT** with comparison conditions: No other intervention
	**Unit of analysis**: Group/organization
	**Sample size**: 34 parent‐led, 45 staff‐led, 35 controls = 69 farms = families
Type of intervention	1.2.4 Individual feedback or coaching
Evaluation design	**Duration of intervention**: Short‐term training varied a bit by farm site
	**Duration of follow‐up**: 2 = Short‐term (≤12 months)
	**Type of outcome measure**: Risk or behavior
Study quality	High quality

**Table:** 

**Kines, P (2013)** *Improving safety in small enterprises through an integrated safety management intervention*.	
Country	DK
Aim	To test the applicability of a participatory behavior‐based injury prevention approach integrated with safety culture initiatives.
Target population	**Occupation**: Metal processing/Dynamic and static
	**Industry**: C—Manufacturing
	**Setting**: 010 Industrial site
	**Firm size**: 10–49 (small)
Study design	**RCT** with comparison conditions: TAU (Treatment as usual)
	**Unit of analysis**: Group/organizational level
	**Sample size**:16 metal industry enterprises; 8 treatment with 2 dropouts and 8 control = 16
Type of intervention	2.1.7 Leadership‐based safety interventions
Evaluation design	**Duration of intervention**: 4 dialogue meetings of 30–45 min between on‐site owner/manager and at least 2 owner/manager led dialogue meetings of 30–60 min with workers under the presence of an on‐site research team member over the 26‐week period.
	**Duration of follow‐up**: 2 = Short‐term (≤12 months)
	**Type of outcome measure**: Risk or behavior
Study quality	Moderate quality

**Table:** 

**Morgan, PJ (2012)** *The impact of a workplace‐based weight loss program on work‐related outcomes in overweight male shift workers*.	
Country	AU
Aim	To evaluate the impact of a workplace‐based weight loss program (Workplace POWER) for male shift workers on a number of outcomes (including injuries).
Target population	**Occupation**: Shift workers at metal factory
	**Industry**: C—Manufacturing
	**Setting**: 010 Industrial site
	**Firm size**: 250+(large)
Study design	**RCT** with comparison conditions: TAU (Treatment as usual)
	**Unit of analysis**: Mixed levels
	**Sample size**: 110 total; 65 to program, 45 to wait‐list control group; recruited from any employees overweight or obese between 18 and 65 years old = 110
Type of intervention	1.3.1 Individual physical training
Evaluation design	**Duration of intervention**: 14 weeks
	**Duration of follow‐up**: 2 = Short‐term (≤12 months)
	**Type of outcome measure**: Other
Study quality	High quality

**Table:** 

**Peek‐Asa, C (2004)** *Compliance to a workplace violence prevention program in small businesses*.	
Country	US
Aim	Evaluate a workplace violence prevention program implemented from August 1997 through August 2000.
Target population	**Occupation**: Sales persons
	**Industry**: G—Wholesale and retail trade
	**Setting**: 040 tertiary activity area (such as office, teaching establishment, restaurant etc.), incl retail
	**Firm size**: Mixed firm size
Study design	**RCT** with comparison conditions: TAU (Treatment as usual)
	**Unit of analysis**: Group/organizational level
	**Sample size**: intervention 345; control 96 = 441
Type of intervention	3.3 Multifaceted across individual and organizational levels
Evaluation design	**Duration of intervention**: Varied depending on the actual shop. Some educational training could have been shorter, like hours or day, and some interventions, such as changing environment is lasting. The total implementation period for the whole program (all interventions) was three
	**Duration of follow‐up**: 2 = Short‐term (≤12 months)
	**Type of outcome measure**: Injury
Study quality	High quality

**Table:** 

**Prunet, B (2008)** *A prospective randomized trial of two safety peripheral intravenous catheters*. Study arm: Passive engineering control	
Country	FR
Aim	In this prospective randomized survey, we compared a passive safety catheter with an active safety catheter and a nonsafety classic catheter.
Target population	**Occupation**: Human health activities
	**Industry**: Q—Human health and social work activities
	**Setting**: 050 Health establishment
	**Firm size**: 250+(large)
Study design	**RCT** with comparison conditions: TAU (Treatment as usual)
	**Unit of analysis**: Group/organizational level
	**Sample size**: Staff's exposure to blood during use of different intravenous catheters. Only secondary outcome in terms of 73 exposures in 759 procedures (no injuries measured).
Type of intervention	2.2.4 Engineering controls
Evaluation design	**Duration of intervention**: 5 months
	**Duration of follow‐up**: 2 = Short‐term (≤12 months)
	**Type of outcome measure**: Injury
Study quality	Moderate quality

**Table:** 

**Prunet, B (2008)** *A prospective randomized trial of two safety peripheral intravenous catheters*. Study arm. Active engineering control	
Country	FR
Aim	In this prospective randomized survey, we compared a passive safety catheter with an active safety catheter and a nonsafety classic catheter.
Target population	**Occupation**: Human health activities
	**Industry**: Q—Human health and social work activities
	**Setting**: 050 Health establishment
	**Firm size**: 250+(large)
Study design	**RCT** with comparison conditions: TAU (Treatment as usual)
	**Unit of analysis**: Group/organizational level
	**Sample size**: Staff's exposure to blood during use of different intravenous catheters. Only secondary outcome in terms of 73 exposures in 759 procedures (no injuries measured).
Type of intervention	2.2.4 Engineering controls
Evaluation design	**Duration of intervention**: 5 months
	**Duration of follow‐up**: 2 = Short‐term (≤12 months)
	**Type of outcome measure**: Injury
Study quality	Moderate quality

**Table:** 

**Rasmussen, K (2003)** *Prevention of farm injuries in Denmark*.	
Country	DK
Aim	To examine the effect of a 4‐year randomized intervention program that combined a safety audit with safety behavior training in the prevention of farm injuries.
Target population	**Occupation**: Crop and animal production, hunting and related service activities
	**Industry**: A—Agriculture, forestry and fishing
	**Setting**: 030 Farming and forestry
	**Firm size**: 1–9 (micro)
Study design	**RCT** with comparison conditions: TAU (Treatment as usual)
	**Unit of analysis**: Group/organizational level
	**Sample size**: The intervention group contained 99 farms, and the control group had 102 farms. = 201
Type of intervention	3.1 Multifaceted at the individual level
Evaluation design	**Duration of intervention**: 1.5 days
	**Duration of follow‐up**: 2 = Short‐term (≤12 months)
	**Type of outcome measure**: Injury
Study quality	High quality

**Table:** 

**Rautiainen, RH (2004)** *Injuries in the Iowa Certified Safe Farm Study*.	
Country	US
Aim	To assess injury characteristics and risk factors in the Iowa Certified Farm (CSF) program and to evaluate the effectiveness of CSF for reducing injuries.
Target population	**Occupation**: Crop and animal production, hunting and related service activities
	**Industry**: A—Agriculture, forestry and fishing
	**Setting**: 030 Farming and forestry
	**Firm size**: Unclear/not reported
Study design	**RCT** with comparison conditions: Alternative type of intervention
	**Unit of analysis**: Group/organizational level
	**Sample size**: 152 farms intervention + 164 farms control (1999) (2000: drop‐outs replaced to maintain at least 125 farms in each group) = 316 ‐
Type of intervention	3.1 Multifaceted at the individual level
Evaluation design	**Duration of intervention**: 3 years
	**Duration of follow‐up**: 3 = Medium‐term (12–36 months)
	**Type of outcome measure**: Injury
Study quality	High quality

**Table:** 

**Srikrajang, J (2005)** *Effectiveness of education and problem solving work group on nursing practices to prevent needlestick and sharp injury*.	
Country	TH
Aim	To examine the effect of an education program and problem solving group on nursing practices for prevention of needlestick and sharp injury.
Target population	**Occupation**: Nursing work
	**Industry**: Q—Human health and social work activities
	**Setting**: 050 Health establishment
	**Firm size**: Unclear/not reported
Study design	**RCT** with comparison conditions: TAU (Treatment as usual)
	**Unit of analysis**: Group/organizational level
	**Sample size**: 12 healthcare workers intervention + 12 healthcare workers control = 24
Type of intervention	3.3 Multifaceted across individual and organizational levels
Evaluation design	**Duration of intervention**: 1 month
	**Duration of follow‐up**: 2 = Short‐term (≤12 months)
	**Type of outcome measure**: Risk or behavior
Study quality	Low quality

**Table:** 

**Van der Molen, HF (2011)** *Better effect of the use of a needle safety device in combination with an interactive workshop to prevent needle stick injuries*. Study arm: Introduction of needles with safety devices	
Country	NL
Aim	Comparing the effectiveness of two types of interventions with no intervention on the prevention of needle stick injuries.
Target population	**Occupation**: Nurses/static
	**Industry**: Q—Human health and social work activities
	**Setting**: 050 Health establishment
	**Firm size**: 250+(large)
Study design	**RCT** with comparison conditions: TAU (Treatment as usual)
	**Unit of analysis**: Group/organizational level
	**Sample size**: 267 cases, 266 control = 533
Type of intervention	2.2.4 Engineering controls
Evaluation design	**Duration of intervention**: 12 months with new needles
	**Duration of follow‐up**: 2 = Short‐term (≤12 months)
	**Type of outcome measure**: Injury
Study quality	High quality

**Table:** 

**Van der Molen, HF (2011)** *Better effect of the use of a needle safety device in combination with an interactive workshop to prevent needle stick injuries*. Study arm: 1 h work shop	
Country	NL
Aim	Comparing the effectiveness of two types of interventions with no intervention on the prevention of needle stick injuries.
Target population	**Occupation**: Nurses/static
	**Industry**: Q—Human health and social work activities
	**Setting**: 050 Health establishment
	**Firm size**: 250+(large)
Study design	**RCT** with comparison conditions: TAU (Treatment as usual)
	**Unit of analysis**: Group/organizational level
	**Sample size**: 263 case, 266 control = 529
Type of intervention	1.1.2 Counseling approaches
Evaluation design	**Duration of intervention**: 1‐h workshop offered two to three times per ward during a 4‐month period February–May 2007
	**Duration of follow‐up**: 2 = Short‐term (≤12 months)
	**Type of outcome measure**: Injury
Study quality	Moderate quality

**Table:** 

**Zohar, D (2002)** *Modifying supervisory practices to improve subunit safety: a leadership‐based intervention model*.	
Country	IL
Aim	To present a leadership‐based intervention model designed to modify supervisory monitoring and rewarding of subordinates' safety performance.
Target population	**Occupation**: Repair and installation of machinery and equipment
	**Industry**: C—Manufacturing
	**Setting**: 010 Industrial site
	**Firm size**: 250+(large)
Study design	**RCT** with comparison conditions: TAU (Treatment as usual)
	**Unit of analysis**: Group/organizational level
	**Sample size**: 190 line workers and 18 supervisors intervention + 191 line workers and 18 supervisors control = 417.
Type of intervention	2.1.7 Leadership‐based safety interventions
Evaluation design	**Duration of intervention**: 8 weeks
	**Duration of follow‐up**: 2 = Short‐term (≤12 months)
	**Type of outcome measure**: Injury
Study quality	Moderate quality

#### 9.1.2 Included CBA studies

*The characteristics of the studies with CBA design are listed below ordered by main type of safety intervention*.

**Table:** 

**Banco, L (1997)** *The Safe Teen Work Project: a study to reduce cutting injuries among young and inexperienced workers*. Study arm: Engineering control	
Country	US
Aim	Evaluate whether the frequency of cutting injuries among youth and inexperienced workers could be reduced by the use of safety case cutters plus appropriate education, when compared to workers using old case cutters without safety education.
Target population	**Occupation**: Retail trade, except of motor vehicles and motorcycles
	**Industry**: G—Wholesale and retail trade
	**Setting**: 040 tertiary activity area (such as office, teaching establishment, restaurant etc.), incl retail
	**Firm size**: Unclear/not reported
Study design	**CBA** with comparison conditions: TAU (Treatment as usual)
	**Unit of analysis**: Group/organizational level
	**Sample size**: 152 cutting injuries; 54 intervention group A (48 baseline + 6 F/U), 98 control group (79 baseline + 19 F/U)
Type of intervention	2.2.4 Engineering controls
Evaluation design	**Duration of intervention**: 3 months
	**Duration of follow‐up**: 2 = Short‐term (≤12 months)
	**Type of outcome measure**: Injury
Study quality	High quality

**Table:** 

**Banco, L (1997)** *The Safe Teen Work Project: a study to reduce cutting injuries among young and inexperienced workers*. Study arm: Safety training	
Country	US
Aim	Evaluate whether the frequency of cutting injuries among youth and inexperienced workers could be reduced by use of old case cutters plus instruction in their safe use, when compared to workers using old case cutters without safety education.
Target population	**Occupation**: Retail trade, except of motor vehicles and motorcycles
	**Industry**: G—Wholesale and retail trade
	**Setting**: 040 tertiary activity area (such as office, teaching establishment, restaurant etc.), incl retail
	**Firm size**: Unclear/not reported
Study design	**CBA** with comparison conditions: TAU (Treatment as usual)
	**Unit of analysis**: Group/organizational level
	**Sample size**: 145 cutting injuries; 47 intervention group B (39 baseline + 8 F/U), 98 control group (79 baseline + 19 F/U)
Type of intervention	1.2.2 Safety training
Evaluation design	**Duration of intervention**: 3 months
	**Duration of follow‐up**: 2 = Short‐term (≤12 months)
	**Type of outcome measure**: Injury
Study quality	High quality

**Table:** 

**Bell, JL (2002)** *Changes in logging injury rates associated with use of feller‐bunchers in West Virginia*.	
Country	US
Aim	To determine whether West Virginia (WV) logging companies experienced a reduction in injuries after beginning to use feller‐bunchers (tree cutting machines, which replace some of the work done with a chainsaw) during harvesting operations.
Target population	**Occupation**: Agriculture, forestry, and fishing
	**Industry**: A—Agriculture, forestry, and fishing
	**Setting**: 030 Farming and forestry
	**Firm size**: 250+(large)
Study design	**CBA** with comparison conditions: TAU (Treatment as usual)
	**Unit of analysis**: Group/organizational level
	**Sample size**: 11 logging companies w 19.4 ‐ 5.2 injury claims/100 workers. Number of workers unknown.
Type of intervention	2.2.4 Engineering controls
Evaluation design	**Duration of intervention**: 2–5 years
	**Duration of follow‐up**: 4 = Long‐term (>36 months)
	**Type of outcome measure**: Injury
Study quality	Moderate quality

**Table:** 

**Black, TR (2011)** *Effect of transfer, lifting, and repositioning (TLR) injury prevention program on musculoskeletal injury among direct care workers*.	
Country	CA
Aim	To evaluate the effectiveness of a Transfer, Lifting and Repositioning (TLR) program to reduce musculoskeletal injuries (MSI) among direct health care workers.
Target population	**Occupation**: Residential care activities
	**Industry**: Q—Human health and social work activities
	**Setting**: 050 Health establishment
	**Firm size**: 250+(large)
Study design	**CBA** with comparison conditions: TAU (Treatment as usual)
	**Unit of analysis**: Group/organizational level
	**Sample size**: Musculoskeletal injuries, 411 Intervention (3 hospitals) + 365 control (3 hospitals) = 776
Type of intervention	3.3 Multifaceted across individual and organizational levels
Evaluation design	**Duration of intervention**: Not reported or unclear
	**Duration of follow‐up**: 2 = Short‐term (≤12 months)
	**Type of outcome measure**: Injury
Study quality	Low quality

**Table:** 

**Carrivick, PJW (2002)** *Effectiveness of a workplace risk assessment team in reducing the rate, cost, and duration of occupational injury*.	
Country	AU
Aim	Evaluate the effectiveness of a consultative workplace risk assessment team in reducing the rate and severity of injury among cleaners within a 600‐bed hospital.
Target population	**Occupation**: Services to buildings and landscape activities
	**Industry**: N—Administrative and support service activities
	**Setting**: 050 Health establishment
	**Firm size**: 250+(large)
Study design	**CBA** with comparison conditions: TAU (Treatment as usual)
	**Unit of analysis**: Group/organizational level
	**Sample size**: 137 cleaners (intervention) + 128 orderlies (comparison) = 265
Type of intervention	3.2 Multifaceted at the group or organizational level
Evaluation design	**Duration of intervention**: 3 years
	**Duration of follow‐up**: 3 = Medium‐term (12–36 months)
	**Type of outcome measure**: Injury
Study quality	Low quality

**Table:** 

**Evanoff, BA (1999)** *Effects of a participatory ergonomics team among hospital orderlies*.	
Country	US
Aim	To study the effects of a participatory worker–management ergonomics team among hospital orderlies.
Target population	**Occupation**: Human health activities
	**Industry**: Q—Human health and social work activities
	**Setting**: 050 Health establishment
	**Firm size**: 250+(large)
Study design	**CBA** with comparison conditions: TAU (Treatment as usual)
	**Unit of analysis**: Group/organizational level
	**Sample size**: Hospital orderlies—100–110 average, questionnaires: 67 baseline + 87 follow‐up = 105
Type of intervention	3.3 Multifaceted across individual and organizational levels
Evaluation design	**Duration of intervention**: 8 h training session/15 months
	**Duration of follow‐up**: 2 = Short‐term (≤12 months)
	**Type of outcome measure**: Injury
Study quality	Low quality

**Table:** 

**Foley, M (2012)** *The impact of regulatory enforcement and consultation visits on workers' compensation claims incidence rates and costs, 1999–2008*. Study arm: Non‐fixed sites with enforcement	
Country	US
Aim	Examine changes in workers compensation claim rates (CIRs) and costs for Washington employers having either an inspection, with or without citation, or a voluntary consultation activity.
Target population	**Occupation**: mixed, all
	**Industry**: All or mixed industries
	**Setting**: 888 All or mixed setting
	**Firm size**: Mixed firm size
Study design	**CBA** with comparison conditions: TAU (Treatment as usual)
	**Unit of analysis**: Group/organizational level
	**Sample size**: 86,314 different businesses observed over 10 year period, all are not included in every year. 4 years of followup for each account allowed
Type of intervention	2.2.7 Enforcement of laws and regulations
Evaluation design	**Duration of intervention**: Ongoing process of inspections; on individual level short term inspections and reports
	**Duration of follow‐up**: 4 = Long‐term (>36 months)
	**Type of outcome measure**: Injury
Study quality	High quality

**Table:** 

**Foley, M (2012)** *The impact of regulatory enforcement and consultation visits on workers' compensation claims incidence rates and costs, 1999–2008*. Study arm: Non‐fixed sites with consultation	
Country	US
Aim	Examine changes in workers compensation claim rates (CIRs) and costs for Washington employers having either an inspection, with or without citation, or a voluntary consultation activity.
Target population	**Occupation**: mixed, all
	**Industry**: All or mixed industries
	**Setting**: 888 All or mixed setting
	**Firm size**: Mixed firm size
Study design	**CBA** with comparison conditions: TAU (Treatment as usual)
	**Unit of analysis**: Group/organizational level
	**Sample size**: 86,314 different businesses observed over 10 year period, all are not included in every year. 4 years of followup for each account allowed
Type of intervention	2.2.3 Soft regulation
Evaluation design	**Duration of intervention**: Ongoing process of inspections; on individual level short term inspections and reports
	**Duration of follow‐up**: 4 = Long‐term (>36 months)
	**Type of outcome measure**: Injury
Study quality	High quality

**Table:** 

**Foley, M (2012)** *The impact of regulatory enforcement and consultation visits on workers' compensation claims incidence rates and costs, 1999–2008*. Study arm: Fixed sites with enforcement	
Country	US
Aim	Examine changes in workers compensation claim rates (CIRs) and costs for Washington employers having either an inspection, with or without citation, or a voluntary consultation activity.
Target population	**Occupation**: mixed, all
	**Industry**: All or mixed industries
	**Setting**: 888 All or mixed setting
	**Firm size**: Mixed firm size
Study design	**CBA** with comparison conditions: Unknown
	**Unit of analysis**: Group/organizational level
	**Sample size**: 86,314 different businesses observed over 10 year period, all are not included in every year. 4 years of followup for each account allowed
Type of intervention	2.2.7 Enforcement of laws and regulations
Evaluation design	**Duration of intervention**: Ongoing process of inspections; on individual level short term inspections and reports
	**Duration of follow‐up**: 4 = Long‐term (>36 months)
	**Type of outcome measure**: Injury
Study quality	High quality

**Table:** 

**Foley, M (2012)** *The impact of regulatory enforcement and consultation visits on workers' compensation claims incidence rates and costs, 1999–2008*. Study arm: Fixed sites with consultation	
Country	US
Aim	Examine changes in workers compensation claim rates (CIRs) and costs for Washington employers having either an inspection, with or without citation, or a voluntary consultation activity.
Target population	**Occupation**: mixed, all
	**Industry**: All or mixed industries
	**Setting**: 888 All or mixed setting
	**Firm size**: Mixed firm size
Study design	**CBA** with comparison conditions: TAU (Treatment as usual)
	**Unit of analysis**: Group/organizational level
	**Sample size**: 86,314 different businesses observed over 10 year period, all are not included in every year. 4 years of followup for each account allowed
Type of intervention	2.2.3 Soft regulation
Evaluation design	**Duration of intervention**: Ongoing process of inspections; on individual level short term inspections and reports
	**Duration of follow‐up**: 4 = Long‐term (>36 months)
	**Type of outcome measure**: Injury
Study quality	High quality

**Table:** 

**Forst, L. (2004)** *Effectiveness of community health workers for promoting use of safety eyewear by Latino farm workers*. Study arm: Introduction of safety training and safety information	
Country	US
Aim	To evaluate The Community Health Worker “promotor de salud” (CHW) model as a tool for reducing eye injuries in Latino farm workers.
Target population	**Occupation**: Crop and animal production, hunting and related service activities
	**Industry**: A—Agriculture, forestry and fishing
	**Setting**: 030 Farming and forestry
	**Firm size**: 50–249 (medium)
Study design	**CBA** with comparison conditions: Alternative type of intervention
	**Unit of analysis**: Group/organizational level
	**Sample size**: Latino farm workers: 256 intervention farms and 149 control farms. Used observations of safety (use of eyewear table IV, in the study) = 405. Block assignment was done at the level of “farm.”
Type of intervention	3.1 Multifaceted at the individual level
Evaluation design	**Duration of intervention**: 16 week
	**Duration of follow‐up**: 2 = Short‐term (≤12 months)
	**Type of outcome measure**: Risk or behavior
Study quality	Low quality

**Table:** 

**Forst, L. (2004)** *Effectiveness of community health workers for promoting use of safety eyewear by Latino farm workers*. Study arm: Introduction of safety information	
Country	US
Aim	To evaluate The Community Health Worker “promotor de salud” (CHW) model as a tool for reducing eye injuries in Latino farm workers.
Target population	**Occupation**: Crop and animal production, hunting and related service activities
	**Industry**: A—Agriculture, forestry and fishing
	**Setting**: 030 Farming and forestry
	**Firm size**: 50–249 (medium)
Study design	**CBA** with comparison conditions: Alternative type of intervention
	**Unit of analysis**: Group/organizational level
	**Sample size**: Latino farm workers: 298 intervention farms and 149 control farms. Used observations of safety (use of eyewear table IV) = 447. Block assignment was done at the level of “farm.”
Type of intervention	1.1.1 Safety campaign, by use of various means
Evaluation design	**Duration of intervention**: 16 weeks
	**Duration of follow‐up**: 2 = Short‐term (≤12 months)
	**Type of outcome measure**: Risk or behavior
Study quality	Low quality

**Table:** 

**Gregersen, NP (1996)** *Road safety improvement in large companies. An experimental comparison of different measures*. Study arm: Economic incentives	
Country	SE
Aim	To compare four different measures for reducing accident involvement through changed driver behavior.
Target population	**Occupation**: Land transport and transport via pipelines
	**Industry**: H—Transporting and storage
	**Setting**: 060 Public area (public places or transport)
	**Firm size**: 250+(large)
Study design	**CBA** with comparison conditions: TAU (Treatment as usual)
	**Unit of analysis**: Group/organizational level
	**Sample size**: 900 drivers intervention + 988 control drivers = 1888
Type of intervention	2.2.2 Economic incentives
Evaluation design	**Duration of intervention**: 1 year
	**Duration of follow‐up**: 3 = Medium‐term (12–36 months)
	**Type of outcome measure**: Injury
Study quality	High quality

**Table:** 

**Gregersen, NP (1996)** *Road safety improvement in large companies. An experimental comparison of different measures*. Study arm: Safety campaign	
Country	SE
Aim	To compare four different measures for reducing accident involvement through changed driver behavior.
Target population	**Occupation**: Land transport and transport via pipelines
	**Industry**: H—Transporting and storage
	**Setting**: 060 Public area (public places or transport)
	**Firm size**: 250+(large)
Study design	**CBA** with comparison conditions: TAU (Treatment as usual)
	**Unit of analysis**: Group/organizational level
	**Sample size**: 915 drivers intervention + 88 control drivers = 1903
Type of intervention	1.1.1 Safety campaign, by use of various means
Evaluation design	**Duration of intervention**: 1 year
	**Duration of follow‐up**: 3 = Medium‐term (12–36 months)
	**Type of outcome measure**: Injury
Study quality	High quality

**Table:** 

**Gregersen, NP (1996)** *Road safety improvement in large companies. An experimental comparison of different measures*. Study arm: Safety training	
Country	SE
Aim	To compare four different measures for reducing accident involvement through changed driver behavior.
Target population	**Occupation**: Land transport and transport via pipelines
	**Industry**: H—Transporting and storage
	**Setting**: 060 Public area (public places or transport)
	**Firm size**: 250+(large)
Study design	**CBA** with comparison conditions: TAU (Treatment as usual)
	**Unit of analysis**: Group/organizational level
	**Sample size**: 936 drivers intervention + 988 control drivers = 1924. It was impossible to choose individual drivers throughout the company. The working units were used instead.
Type of intervention	1.2.2 Safety training
Evaluation design	**Duration of intervention**: 8 h
	**Duration of follow‐up**: 3 = Medium‐term (12–36 months)
	**Type of outcome measure**: Injury
Study quality	High quality

**Table:** 

**Gregersen, NP (1996)** *Road safety improvement in large companies. An experimental comparison of different measures*. Study arm: Counseling	
Country	SE
Aim	To compare four different measures for reducing accident involvement through changed driver behavior.
Target population	**Occupation**: Land transport and transport via pipelines
	**Industry**: H—Transporting and storage
	**Setting**: 060 Public area (public places or transport)
	**Firm size**: 250+(large)
Study design	**CBA** with comparison conditions: TAU (Treatment as usual)
	**Unit of analysis**: Group/organizational level
	**Sample size**: 917 drivers intervention + 988 control drivers = 1905
Type of intervention	1.1.2 Counseling approaches
Evaluation design	**Duration of intervention**: 3 h
	**Duration of follow‐up**: 3 = Medium‐term (12–36 months)
	**Type of outcome measure**: Injury
Study quality	High quality

**Table:** 

**Grimmond, T (2010)** *Sharps injury reduction using a sharps container with enhanced engineering: A 28 hospital nonrandomized intervention and cohort study*.	
Country	NZ
Aim	To test the hypothesis that the Device's engineered safety features would reduce CASI (container‐associated sharps injuries) and, hopefully, total SI (sharps injuries).
Target population	**Occupation**: Human health activities
	**Industry**: Q—Human health and social work activities
	**Setting**: 050 Health establishment
	**Firm size**: 250+(large)
Study design	**CBA** with comparison conditions: TAU (Treatment as usual)
	**Unit of analysis**: Group/organizational level
	**Sample size**: Hospitals, 14 intervention + 14 controls = 28
Type of intervention	2.2.4 Engineering controls
Evaluation design	**Duration of intervention**: 1 year
	**Duration of follow‐up**: 2 = Short‐term (≤12 months)
	**Type of outcome measure**: Injury
Study quality	High quality

**Table:** 

**Harms‐Ringdahl, L (1987)** *Safety analysis in design—evaluation of a case study*.	
Country	SE
Aim	To find measures to decrease occupational accidents.
Target population	**Occupation**: Manufacturing of paper and paper products
	**Industry**: C— Manufacturing
	**Setting**: 010 Industrial site
	**Firm size**: 50–249 (medium)
Study design	**CBA** with comparison conditions: TAU (Treatment as usual)
	**Unit of analysis**: Group/organizational level
	**Sample size**: About 30 paper mill workers. No information on the control group.
Type of intervention	2.2.4 Engineering controls
Evaluation design	**Duration of intervention**: Not reported or unclear
	**Duration of follow‐up**: 3 = Medium‐term (12–36 months)
	**Type of outcome measure**: Injury
Study quality	Low quality

**Table:** 

**Haviland, AM (2012)** *A new estimate of the impact of OSHA inspections on manufacturing injury rates, 1998–2005*. Study arm: Programmed inspections	
Country	US
Aim	(1) Measure the average effect of inspections with and without penalties 1998–2005 and the timing of these effects. (2) Identify particular types of inspections and workplaces where the effects have been larger and smaller.
Target population	**Occupation**: static
	**Industry**: C—Manufacturing
	**Setting**: 010 Industrial site
	**Firm size**: Mixed firm size
Study design	**CBA** with comparison conditions: TAU (Treatment as usual)
	**Unit of analysis**: Group/organizational level
	**Sample size**: Industrial businesses on one site with >10 employees in Pennsylvania w restrictions applied = 8645 firms with 44,821 firm‐years of observation
Type of intervention	2.2.7 Enforcement of laws and regulations
Evaluation design	**Duration of intervention**: Inspections are short‐term (generally a day).
	**Duration of follow‐up**: 3 = Medium‐term (12–36 months)
	**Type of outcome measure**: Injury
Study quality	Low quality

**Table:** 

**Haviland, AM (2012)** *A new estimate of the impact of OSHA inspections on manufacturing injury rates, 1998–2005*. Study arm: Programmed inspections with penalty	
Country	US
Aim	(1) Measure the average effect of inspections with and without penalties 1998–2005 and the timing of these effects. (2) Identify particular types of inspections and workplaces where the effects have been larger and smaller.
Target population	**Occupation**: static
	**Industry**: C—Manufacturing
	**Setting**: 010 Industrial site
	**Firm size**: Mixed firm size
Study design	**CBA** with comparison conditions: TAU (Treatment as usual)
	**Unit of analysis**: Group/organizational level
	**Sample size**: Industrial businesses on one site with >10 employees in Pennsylvania w restrictions applied = 8645 firms with 44,821 firm‐years of observation
Type of intervention	2.2.7 Enforcement of laws and regulations
Evaluation design	**Duration of intervention**: Inspections are short‐term (generally a day).
	**Duration of follow‐up**: 3 = Medium‐term (12–36 months)
	**Type of outcome measure**: Injury
Study quality	Low quality

**Table:** 

**Haviland, AM (2012)** *A new estimate of the impact of OSHA inspections on manufacturing injury rates, 1998–2005*. Study arm: Complaint inspections with penalty	
Country	US
Aim	(1) Measure the average effect of inspections with and without penalties 1998–2005 and the timing of these effects. (2) Identify particular types of inspections and workplaces where the effects have been larger and smaller.
Target population	**Occupation**: static
	**Industry**: C—Manufacturing
	**Setting**: 010 Industrial site
	**Firm size**: Mixed firm size
Study design	**CBA** with comparison conditions: TAU (Treatment as usual)
	**Unit of analysis**: Group/organizational level
	**Sample size**: Industrial businesses on one site with >10 employees in Pennsylvania w restrictions applied = 8645 firms with 44,821 firm‐years of observation
Type of intervention	2.2.7 Enforcement of laws and regulations
Evaluation design	**Duration of intervention**: Inspections are short‐term (generally a day).
	**Duration of follow‐up**: 3 = Medium‐term (12–36 months)
	**Type of outcome measure**: Injury
Study quality	Low quality

**Table:** 

**Haviland, AM (2012)** *A new estimate of the impact of OSHA inspections on manufacturing injury rates, 1998–2005*. Study arm: Complaint inspections	
Country	US
Aim	(1) Measure the average effect of inspections with and without penalties 1998–2005 and the timing of these effects. (2) Identify particular types of inspections and workplaces where the effects have been larger and smaller.
Target population	**Occupation**: static
	**Industry**: C—Manufacturing
	**Setting**: 010 Industrial site
	**Firm size**: Mixed firm size
Study design	**CBA** with comparison conditions: TAU (Treatment as usual)
	**Unit of analysis**: Group/organizational level
	**Sample size**: Industrial businesses on one site with >10 employees in Pennsylvania w restrictions applied = 8645 firms with 44,821 firm‐years of observation
Type of intervention	2.2.7 Enforcement of laws and regulations
Evaluation design	**Duration of intervention**: Inspections are short‐term (generally a day).
	**Duration of follow‐up**: 3 = Medium‐term (12–36 months)
	**Type of outcome measure**: Injury
Study quality	Low quality

**Table:** 

**Hilyer, JC (1990)** *A flexibility intervention to reduce the incidence and severity of joint injuries among municipal firefighters*.	
Country	US
Aim	469 municipal firefighters examined the effect of flexibility training on the incidence and severity of joint injuries.
Target population	**Occupation**: Public administration and defence; compulsory social security
	**Industry**: O—Public administration and defence
	**Setting**: 130 Emergency, rescuing and military sites
	**Firm size**: 250+(large)
Study design	**CBA** with comparison conditions: No other intervention
	**Unit of analysis**: Group/organizational level
	**Sample size**: Firefighters; 251 intervention + 218 comparison = 169
Type of intervention	1.3.1 Individual physical training
Evaluation design	**Duration of intervention**: 6 months
	**Duration of follow‐up**: 3 = Medium‐term (12–36 months)
	**Type of outcome measure**: Injury
Study quality	Low quality

**Table:** 

**Johnson, OE (2012)** *Effect of health education on the riding habits of commercial motorcyclists in Uyo, southern Nigeria*.	
Country	NG
Aim	Implement and evaluate the effect of safety education on riding habits of motorcyclists.
Target population	**Occupation**: Motorcycle courriers
	**Industry**: H—Transporting and storage
	**Setting**: 060 Public area
	**Firm size**: 1–9 (micro)
Study design	**CBA** with comparison conditions: No other intervention
	**Unit of analysis**: Group/organizational level
	**Sample size**: motorcyclist: 100 intervention, 100 controls (from another town)
Type of intervention	1.1.3 Teaching, education to increase knowledge and awareness
Evaluation design	**Duration of intervention**: Not stated but <1 day
	**Duration of follow‐up**: 2 = Short‐term (≤12 months)
	**Type of outcome measure**: Injury
Study quality	Moderate quality

**Table:** 

**Kim, P (2004)** *The cost‐effectiveness of a back education program for firefighters: a case study*.	
Country	CA
Aim	To assess the feasibility of implementing a multi‐faceted back injury prevention program in the community, and to assess the effectiveness of this program.
Target population	**Occupation**: Public administration and defence; compulsory social security
	**Industry**: O—Public administration and defence
	**Setting**: 130 Emergency, rescuing and military sites
	**Firm size**: 50–249 (medium)
Study design	**CBA** with comparison conditions: TAU (Treatment as usual)
	**Unit of analysis**: Group/organizational level
	**Sample size**: Firefighters; 92 intervention + 175 comparison = 267
Type of intervention	3.1 Multifaceted at the individual level
Evaluation design	**Duration of intervention**: 1 year
	**Duration of follow‐up**: 2 = Short‐term (≤12 months)
	**Type of outcome measure**: Injury
Study quality	Moderate quality

**Table:** 

**Kines, P (2010)** *Improving construction site safety through leader‐based verbal safety communication*.	
Country	DK
Aim	To tests the effect of increasing leader‐based on‐site verbal safety communication on the level of safety and safety climate at construction sites.
Target population	**Occupation**: Construction of buildings
	**Industry**: F—Construction
	**Setting**: 020 Construction site
	**Firm size**: 10–49 (small)
Study design	**CBA** with comparison conditions: TAU (Treatment as usual)
	**Unit of analysis**: Group/organizational level
	**Sample size**: 1693 foremen‐worker verbal exchanges + 22,077 safety observations = 23,770—for up to 42 weeks = 29,430 observations
Type of intervention	2.1.7 Leadership‐based safety interventions
Evaluation design	**Duration of intervention**: 16 weeks
	**Duration of follow‐up**: 2 = Short‐term (≤12 months)
	**Type of outcome measure**: Risk or behavior
Study quality	Low quality

**Table:** 

**Knapik, JJ (2003)** *Injury and fitness outcomes during implementation of physical readiness training*. Study arm: Males	
Country	US
Aim	To examine injury and physical fitness outcomes in Basic Combat Training (BCT) during implementation of Physical Readiness Training (PRT) among male army recruits.
Target population	**Occupation**: Public administration and defence; compulsory social security
	**Industry**: O—Public administration and defence
	**Setting**: 130 Emergency, rescuing and military sites
	**Firm size**: 250+(large)
Study design	**CBA** with comparison conditions: TAU (Treatment as usual)
	**Unit of analysis**: Group/organizational level
	**Sample size**: 1414 male army recruits; 769 intervention + 645 control = 1414
Type of intervention	1.3.1 Individual physical training
Evaluation design	**Duration of intervention**: 9 weeks
	**Duration of follow‐up**: 2 = Short‐term (≤12 months)
	**Type of outcome measure**: Injury
Study quality	Moderate quality

**Table:** 

**Knapik, JJ (2003)** *Injury and fitness outcomes during implementation of physical readiness training*. Study arm: Females	
Country	US
Aim	To examine injury and physical fitness outcomes in Basic Combat Training (BCT) during implementation of Physical Readiness Training (PRT) among female army recruits.
Target population	**Occupation**: Public administration and defence; compulsory social security
	**Industry**: O—Public administration and defence
	**Setting**: 130 Emergency, rescuing and military sites
	**Firm size**: 250+(large)
Study design	**CBA** with comparison conditions: TAU (Treatment as usual)
	**Unit of analysis**: Group/organizational level
	**Sample size**: 1166 female army recruits; 515 intervention + 651 control = 1166
Type of intervention	1.3.1 Individual physical training
Evaluation design	**Duration of intervention**: 9 weeks
	**Duration of follow‐up**: 2 = Short‐term (≤12 months)
	**Type of outcome measure**: Injury
Study quality	Moderate quality

**Table:** 

**Knapik, JJ (2004)** *Influence of an injury reduction program on injury and fitness outcomes among soldiers*. Study army: Males	
Country	US
Aim	Evaluate the influence of a multiple injury control intervention on injury and physical fitness outcomes among soldiers [here: males]attending United States Army Ordnance School Advanced Individual Training.
Target population	**Occupation**: Public administration and defence; compulsory social security
	**Industry**: O—Public administration and defence
	**Setting**: 130 Emergency, rescuing and military sites
	**Firm size**: 250+(large)
Study design	**CBA** with comparison conditions: TAU (Treatment as usual)
	**Unit of analysis**: Group/organizational level
	**Sample size**: 1122 men intervention + 2303 men control = 3425
Type of intervention	3.1 Multifaceted at the individual level
Evaluation design	**Duration of intervention**: 36 weeks
	**Duration of follow‐up**: 3 = Medium‐term (12–36 months)
	**Type of outcome measure**: Injury
Study quality	Moderate quality

**Table:** 

**Knapik, JJ (2004)** *Influence of an injury reduction program on injury and fitness outcomes among soldiers*. Study arm: Females	
Country	US
Aim	Evaluate the influence of a multiple injury control intervention on injury and physical fitness outcomes among soldiers [here: females] attending United States Army Ordnance School Advanced Individual Training.
Target population	**Occupation**: Public administration and defence; compulsory social security
	**Industry**: O—Public administration and defence
	**Setting**: 130 Emergency, rescuing and military sites
	**Firm size**: 250+(large)
Study design	**CBA** with comparison conditions: TAU (Treatment as usual)
	**Unit of analysis**: Group/organizational level
	**Sample size**: 161 women intervention + 256 women control = 417
Type of intervention	3.1 Multifaceted at the individual level
Evaluation design	**Duration of intervention**: 36 weeks
	**Duration of follow‐up**: 3 = Medium‐term (12–36 months)
	**Type of outcome measure**: Injury
Study quality	Moderate quality

**Table:** 

**Lanoie, P (1992)** *Safety regulation and the risk of workplace accidents in Quebec*.	
Country	CA
Aim	To examine the effectiveness of policies adopted by Quebec's occupational safety and health authority, the Commission de la Santé et Sécurité du Travail, in reducing the incidence of workplace accidents after its creation in 1980.
Target population	**Occupation**: All occupations
	**Industry**: All or mixed industries
	**Setting**: 888 All or mixed setting
	**Firm size**: Unclear/not reported
Study design	**CBA** with comparison conditions: Not applicable
	**Unit of analysis**: Group/organizational level
	**Sample size**: not reported
Type of intervention	2.2.1 Legislative changes
Evaluation design	**Duration of intervention**: Not reported or unclear
	**Duration of follow‐up**: 0 = Not reported or unclear
	**Type of outcome measure**:
Study quality	Low quality

**Table:** 

**Levine, DI (2012)** *Randomized government safety inspections reduce worker injuries with no detectable job loss*.	
Country	US
Aim	Analyze a natural field experiment to examine how workplace safety inspections affected injury rates and other outcomes.
Target population	**Occupation**: Mixed
	**Industry**: All or mixed industries
	**Setting**: 888 All or mixed setting
	**Firm size**: Unclear/not reported
Study design	**CBA** with comparison conditions:
	**Unit of analysis**: Group/organizational level
	**Sample size**: 409 matched pairs; only sites with at least 10 employees, single business site, no prior inspection in last 2 years. Matched on industry, size, geographic region of the state.
Type of intervention	2.2.7 Enforcement of laws and regulations
Evaluation design	**Duration of intervention**: One short‐term inspection visit (estimated to be 1 day, which is the expected time needed for an inspection visit and follow‐up in case of orders or penalties)
	**Duration of follow‐up**: 2 = Short‐term (≤12 months)
	**Type of outcome measure**: Injury
Study quality	High quality

**Table:** 

**López‐Ruiz, M (2013)** *Evaluation of the effectiveness of occupational injury prevention programs at the company level*.	
Country	ES
Aim	To evaluate the effectiveness of occupational injury prevention programs (PAP) in a sample of companies in a Spanish region (Valencia), by comparing those companies that have adopted a PAP with other companies that have not adopted these plans.
Target population	**Occupation**: Mixed (3 different sectors)
	**Industry**: All or mixed industries
	**Setting**: 888 All or mixed setting
	**Firm size**: 10–49 (small)
Study design	**CBA** with comparison conditions: TAU (Treatment as usual)
	**Unit of analysis**: Group/organizational level
	**Sample size**: 556 intervention companies, 633 control companies = 1189
Type of intervention	3.2 Multifaceted at the group or organizational level
Evaluation design	**Duration of intervention**: Not stated but probably months
	**Duration of follow‐up**: 4 = Long‐term (>36 months)
	**Type of outcome measure**: Injury
Study quality	Low quality

**Table:** 

**Mattila, M (1988)** *Promoting job safety in building: an experiment on the behavior analysis approach*.	
Country	FI
Aim	To determine whether the behavior analysis approach can be used effectively to improve occupational safety in building and to evaluate experimentally the effectiveness of this safety effort.
Target population	**Occupation**: Construction of buildings
	**Industry**: F—Construction
	**Setting**: 020 construction site
	**Firm size**: Mixed firm size
Study design	**CBA** with comparison conditions: TAU (Treatment as usual)
	**Unit of analysis**: Group/organizational level
	**Sample size**: 51 accidents at intervention site (office building over 22 weeks) + 41 accident at control site (apartment building over 20 weeks) = 92
Type of intervention	2.1.1 Goal setting and feedback at group or organizational level
Evaluation design	**Duration of intervention**: Apartment house site: 20 weeks; Office building site: 22 weeks
	**Duration of follow‐up**: 2 = Short‐term (≤12 months)
	**Type of outcome measure**: Injury
Study quality	Moderate quality

**Table:** 

**Mehrdad, R (2013)** *Effects of training course on occupational exposure to bloodborne pathogens: a controlled interventional study*.	
Country	IR
Aim	To evaluate the effect of a training course on the rate of needle stick injuries and its reporting.
Target population	**Occupation**: Human health activities
	**Industry**: Q—Human health and social work activities
	**Setting**: 050 Health establishment
	**Firm size**: 250+(large)
Study design	**CBA** with comparison conditions: TAU (Treatment as usual)
	**Unit of analysis**: Group/organizational level
	**Sample size**: Intervention: 431 health care workers in hospital A; Control: 1025 health care workers in hospital B. sample size = 1456 health care workers. Hospital A, one out of 140 hospitals accepted to participate, B rejected and became control.
Type of intervention	1.1.3 Teaching, education to increase knowledge and awareness
Evaluation design	**Duration of intervention**: 3 month
	**Duration of follow‐up**: 2 = Short‐term (≤12 months)
	**Type of outcome measure**: Injury
Study quality	Low quality

**Table:** 

**Parker, DL (2009)** *A randomized, controlled intervention of machine guarding and related safety programs in small metal‐fabrication businesses*.	
Country	US
Aim	To improve machine‐related safety in small metal‐fabrication businesses.
Target population	**Occupation**: metal workers
	**Industry**: C—Manufacturing
	**Setting**: 010 Industrial site
	**Firm size**: 50–249 (medium)
Study design	**CBA** with comparison conditions: Alternative type of intervention
	**Unit of analysis**: Group/organizational level
	**Sample size**: 40 originally randomized to one of two arms, three lost to follow‐up of which two went out of business. Final analyses based on 18 control (management only) and 19 treatment shops with management‐employee involvement = 37
Type of intervention	3.3 Multifaceted across individual and organizational levels
Evaluation design	**Duration of intervention**: 4 months
	**Duration of follow‐up**: 2 = Short‐term (≤12 months)
	**Type of outcome measure**: Risk or behavior
Study quality	Low quality

**Table:** 

**Peate, WF (2007)** *Core strength: a new model for injury prediction and prevention*.	
Country	US
Aim	Many work in injury prone awkward positions that require adequate flexibility and strength in trunk stabilizer muscle groups. Performance on a Functional Movement Screen (FMS) that assessed those factors was conducted and an intervention was designed.
Target population	**Occupation**: Public administration and defence; compulsory social security
	**Industry**: O—Public administration and defence
	**Setting**: 130 Emergency, rescuing and military sites
	**Firm size**: 250+(large)
Study design	**CBA** with comparison conditions: TAU (Treatment as usual)
	**Unit of analysis**: Group/organizational level
	**Sample size**: 433 firefighters (408 male, 25 female)—size of historical control group not disclosed. Intervention at individual level and group level, but analyses on group level.
Type of intervention	3.1 Multifaceted at the individual level
Evaluation design	**Duration of intervention**: 3 h
	**Duration of follow‐up**: 3 = Medium‐term (12–36 months)
	**Type of outcome measure**: Injury
Study quality	Low quality

**Table:** 

**Quintana, R (1999)** *A task‐delineated safety approach for slip, trip and fall hazards*.	
Country	US
Aim	To address the significant problems of slip, trip, and fall accidents through task‐delineated safety (TDS), a behavior‐based safety management scheme.
Target population	**Occupation**: Warehousing and support activities for transportation
	**Industry**: H—Transporting and storage
	**Setting**: 040 tertiary activity area (such as office, teaching establishment, restaurant etc.), incl retail
	**Firm size**: Unclear/not reported
Study design	**CBA** with comparison conditions: No other intervention
	**Unit of analysis**: Group/organizational level
	**Sample size**: not reported
Type of intervention	1.2.4 Individual feedback or coaching
Evaluation design	**Duration of intervention**: Not reported or unclear
	**Duration of follow‐up**: 0 = Not reported or unclear
	**Type of outcome measure**: Risk or behavior
Study quality	Low quality

**Table:** 

**Rasmussen, K (2006)** *Worker participation in change processes in a Danish industrial setting*	
Country	DK
Aim	To study the development and implementation of the intervention with both quantitative and qualitative methods. Furthermore, intervention effects with regard to both the psychosocial and the physical work environment are assessed.
Target population	**Occupation**: Manufacture of machinery and equipment.
	**Industry**: C—Manufacturing
	**Setting**: 010 Industrial site
	**Firm size**: 250+(large)
Study design	**CBA** with comparison conditions: TAU (Treatment as usual)
	**Unit of analysis**: Group/organizational level
	**Sample size**: Production workers. Intervention: 620 at baseline and increasing gradually to 940 + 40–50 supervisors, two company directors and three top management; after downsizing: 480. Comparison: 270 increasing over two years to 520. = 1510
Type of intervention	3.3 Multifaceted across individual and organizational levels
Evaluation design	**Duration of intervention**: 2,5 years
	**Duration of follow‐up**: 2 = Short‐term (≤12 months)
	**Type of outcome measure**: Injury
Study quality	Moderate quality

**Table:** 

**Ray, PS (1997)** *Efficacy of the components of a behavioral safety program*	
Country	US
Aim	To get an indication of the relative effectiveness of safety training, performance feedback, and goal‐setting components of a behavioral safety program.
Target population	**Occupation**: Manufacture of fabricated metal products, except machinery and equipment
	**Industry**: C—Manufacturing
	**Setting**: 010 Industrial site
	**Firm size**: 250+(large)
Study design	**CBA** with comparison conditions: TAU (Treatment as usual)
	**Unit of analysis**: Group/organizational level
	**Sample size**: Workers, 22 intervention + 19 control = 41 in sample.
Type of intervention	2.1.1 Goal setting and feedback at group or organizational level
Evaluation design	**Duration of intervention**: 2 months
	**Duration of follow‐up**: 2 = Short‐term (≤12 months)
	**Type of outcome measure**: Risk or behavior
Study quality	Low quality

**Table:** 

**Santaweesuk, S (2014)** *Effects of an injury and illness prevention program on occupational safety behaviors among rice farmers in Nakhon Nayok Province, Thailand*.	
Country	TH
Aim	To determine the effects of an injury and illness prevention program intervention on occupational safety behavior among rice farmers in Nakhon Nayok province, Thailand.
Target population	**Occupation**: Crop and animal production, hunting and related service activities
	**Industry**: A—Agriculture, forestry and fishing
	**Setting**: 030 Farming and forestry
	**Firm size**: 1–9 (micro)
Study design	**CBA** with comparison conditions: TAU (Treatment as usual)
	**Unit of analysis**: Group/organizational level
	**Sample size**: Rice farmers: Intervention: 62, Control: 55 = 117.
Type of intervention	3.1 Multifaceted at the individual level
Evaluation design	**Duration of intervention**: 2 weeks
	**Duration of follow‐up**: 2 = Short‐term (≤12 months)
	**Type of outcome measure**: Risk or behavior
Study quality	Low quality

**Table:** 

**Valls, V (2007)** *Use of safety devices and the prevention of percutaneous injuries among healthcare workers*.	
Country	ES
Aim	To study the effectiveness of safety devices intended to prevent percutaneous injuries.
Target population	**Occupation**: Human health activities
	**Industry**: Q—Human health and social work activities
	**Setting**: 050 Health establishment
	**Firm size**: 250+(large)
Study design	**CBA** with comparison conditions: TAU (Treatment as usual)
	**Unit of analysis**: Group/organizational level
	**Sample size**: 75 nurses participated in intervention activities BUT needles were distributed at over half the hospital (=>500 employees) = 75. No exact information on the size of control group.
Type of intervention	3.3 Multifaceted across individual and organizational levels
Evaluation design	**Duration of intervention**: 6 months
	**Duration of follow‐up**: 2 = Short‐term (≤12 months)
	**Type of outcome measure**: Injury
Study quality	High quality

**Table:** 

**Wang, H (2003)** *A training programme for prevention of occupational exposure to bloodborne pathogens: impact on knowledge, behavior and incidence of needle stick injuries among student nurses in Changsha, People's Republic of China*.	
Country	CN
Aim	To examine the impact of structured training on prevention of occupational exposure to bloodborne pathogens (BBP) on knowledge, behavior, and incidence of medical sharp injuries among student nurses in Changsha, China.
Target population	**Occupation**: Human health activities
	**Industry**: Q—Human health and social work activities
	**Setting**: 050 Health establishment
	**Firm size**: 250+(large)
Study design	**CBA** with comparison conditions: TAU (Treatment as usual)
	**Unit of analysis**: Group/organizational level
	**Sample size**: Nursing students: 56 intervention (46 responses), 50 control (45 responses) = 106
Type of intervention	1.1.3 Teaching, education to increase knowledge and awareness
Evaluation design	**Duration of intervention**: 60 min lecture and 20 min video
	**Duration of follow‐up**: 2 = Short‐term (≤12 months)
	**Type of outcome measure**: Risk or behavior
Study quality	Low quality

#### 9.1.3 Included ITS studies

*The characteristics of studies with serial measures including interrupted time series (ITS) with and without external or internal comparison groups, retrospective cohort designs, serial cross‐sectional studies and before and after studies with at least 3 measures over time*.

**Table:** 

**Alamgir, H (2008)** *Efficiency of overhead ceiling lifts in reducing musculoskeletal injury among carers working in long‐term care institutions*.	
Country	CA
Aim	To evaluate the effectiveness and cost benefit of overhead lifts in three long‐term care facilities in reducing the risk of musculoskeletal injury among healthcare workers.
Target population	**Occupation**: Residential long term care activities/dynamic
	**Industry**: Q—Human health and social work activities
	**Setting**: 050 Health establishment
	**Firm size**: 50–249 (medium)
Study design	**ITS** with comparison conditions: 1. Simple serial measures (No comparison)
	**Unit of analysis**: Group/organizational level
	**Sample size**: 3 long‐term care facilities, 586 claims (422 before + 164 after installation of overhead lifts)total bed years=4396
Type of intervention	2.2.4 Engineering controls
Evaluation design	**Duration of intervention:** 4 years; change was permanent over observation FU
	**Duration of follow‐up**: 4 = Long‐term (>36 months)
	**Type of outcome measure**: Injury
Study quality	Moderate quality

**Table:** 

**Bell, J (2006)** *Evaluating the effectiveness of a logger safety training program*.	
Country	US
Aim	Evaluating the Effectiveness of a Logger Safety Training (LST) Program
Target population	**Occupation**: logging/dynamic
	**Industry**: A—Agriculture, forestry and fishing
	**Setting**: 030 Farming and forestry
	**Firm size**: Mixed firm size
Study design	**ITS** with comparison conditions: 3. Serial measures with external control
	**Unit of analysis**: Group/organizational level
	**Sample size**: 255 companies; most small; ~1300 workers = 1304
Type of intervention	3.2 Multifaceted at the group or organizational level
Evaluation design	**Duration of intervention:** 4 years
	**Duration of follow‐up**: 4 = Long‐term (>36 months)
	**Type of outcome measure**: Injury
Study quality	Moderate quality

**Table:** 

**Bena, A (2009)** *Effectiveness of the training program for workers at construction sites of the high‐speed railway line between Torino and Novara: impact on injury rates*.	
Country	IT
Aim	Assess impact of safety and health training on injury outcomes in large rail const project
Target population	**Occupation**: Civil engineering: excavation, drivers, operators, carpenters, iron workers, crane operators etc./dynamic
	**Industry**: F—Construction
	**Setting**:
	**Firm size**: Mixed firm size
Study design	**ITS** with comparison conditions: 1. Simple serial measures (No comparison)
	**Unit of analysis**: Group/organizational level
	**Sample size**: 2795 workers who agreed to participate out of 10,289 worker‐jobs
Type of intervention	1.1.3 Teaching, education to increase knowledge and awareness
Evaluation design	**Duration of intervention:** Days of training varied some. Basic module for all and then specific trade specific modules up to 4. Short‐term training exposures basically [Days]
	**Duration of follow‐up**: 3 = Medium‐term (12–36 months)
	**Type of outcome measure**: Injury
Study quality	High quality

**Table:** 

**Bell, J (2008)** *Evaluation of a comprehensive slip, trip, and fall prevention programme for hospital employees*.	
Country	US
Aim	To evaluate effectiveness of multi‐faceted program to prevent STF in hospital environment. implementation of broad‐scale prevention program can significantly reduce STF injury claims.
Target population	**Occupation**: hospital workers/dynamic
	**Industry**: Q—Human health and social work activities
	**Setting**: 050 Health establishment
	**Firm size**: 250+(large)
Study design	**ITS** with comparison conditions: 2. Serial measures with internal control
	**Unit of analysis**: Group/organizational level
	**Sample size**: dynamic cohort of 16,900 employees at 3 health systems contributing over 80 million work hours or 40K person years
Type of intervention	3.3 Multifaceted across individual and organizational levels
Evaluation design	**Duration of intervention:** 3 years
	**Duration of follow‐up**: 3 = Medium‐term (12–36 months)
	**Type of outcome measure**: Injury
Study quality	Low quality

**Table:** 

**Benavides, F (2009)** *Effectiveness of occupational injury prevention policies in Spain*.	
Country	ES
Aim	Examination of effectiveness of preferential actions plans (PAPS) developed by Spanish Regional Govt in 2000 to prevent non‐fatal traumatic work injuries. (discrepancy in text though, as Aragon implemented in 1999).
Target population	**Occupation**: Mixed; Manufacturing and private service (and population at risk was salaried workers only)
	**Industry**: All or mixed industries
	**Setting**: 888 All or mixed setting
	**Firm size**: Mixed firm size
Study design	**ITS** with comparison conditions: 2. Serial measures with internal control
	**Unit of analysis**: Group/organizational level
	**Sample size**: 3,252,028 injuries; used Labor Force estimates of salaried workers for denominators over time.
Type of intervention	2.2.7 Enforcement of laws and regulations
Evaluation design	**Duration of intervention:** permanent policy change
	**Duration of follow‐up**: 4 = Long‐term (>36 months)
	**Type of outcome measure**: Injury
Study quality	High quality

**Table:** 

**Birnbaum, D (1993)** *Needlestick injuries among critical care nurses before and after adoption of universal precautions or body substance isolation*.	
Country	CA
Aim	Conducted to see if adoption of Universal Precautions (UP) or Body Substance Isolation (BSI) has resulted in decreased needle recapping or injury rates.
Target population	**Occupation**: Human health activities, critical care nurses
	**Industry**: Q—Human health and social work activities
	**Setting**: 050 Health establishment
	**Firm size**: 250+(large)
Study design	**ITS** with comparison conditions: 1. Simple serial measures (No comparison)
	**Unit of analysis**: Group/organizational level
	**Sample size**: UNCLEAR: 929 hospital staff from hospitals providing some data (33), hereof 312 hospital staffs from hospitals providing complete data (11)
Type of intervention	2.2.5 Administrative controls
Evaluation design	**Duration of intervention**: 30–60–90 days before and after so total of 6 months
	**Duration of follow‐up**: 2 = Short‐term (≤12 months)
	**Type of outcome measure**: Injury
Study quality	Moderate quality

**Table:** 

**Briggs, SC (2003)** *The effect of supermaximum security prisons on aggregate levels of institutional violence*.	
Country	US
Aim	Examine the effect of supermaxes on aggregate levels of violence in three prison systems using a multiple interrupted time series design.
Target population	**Occupation**: prison guards/dynamic
	**Industry**: O—Public administration and defence
	**Setting**: 040 tertiary activity area (such as office, teaching establishment, restaurant etc.), incl retail
	**Firm size**: 250+(large)
Study design	**ITS** with comparison conditions: 3. Serial measures with external control
	**Unit of analysis**: Group/organizational level
	**Sample size**: Cannot determine clearly sample size from paper (across all 4 sites but a lot of data over time:: I tabel 1 angiver de “monthly rates of staff assaults” = UNKNOWN)
Type of intervention	2.2.4 Engineering controls
Evaluation design	**Duration of intervention**: Varied by site, 8–20 years across 4 sites
	**Duration of follow‐up**: 4 = Long‐term (>36 months)
	**Type of outcome measure**: Injury
Study quality	Moderate quality

**Table:** 

**Bull, N (2007)** *Mandatory use of eye protection prevents eye injuries in the metal industry*.	
Country	NO
Aim	To show the preventive effect of mandatory use of eye protection to challenge authorities to make use of eye protection mandatory in the metal industry in Norway.
Target population	**Occupation**: Metal workers (large hulls etc.)
	**Industry**: C—Manufacturing
	**Setting**: 010 Industrial site
	**Firm size**: 250+(large)
Study design	**ITS** with comparison conditions: 1. Simple serial measures (No comparison)
	**Unit of analysis**: Group/organizational level
	**Sample size**: 1140 metal yard workers average per year
Type of intervention	3.3 Multifaceted across individual and organizational levels
Evaluation design	**Duration of intervention:** Immediate policy change requiring use of eye protection
	**Duration of follow‐up**: 4 = Long‐term (>36 months)
	**Type of outcome measure**: Injury
Study quality	High quality

**Table:** 

**Bulzacchelli, MT (2007)** *Effects of the Occupational Safety and Health Administration's control of hazardous energy (lockout/tagout) standard on rates of machinery‐related fatal occupational injury*.	
Country	US
Aim	To evaluate the impact of the United States' federal Occupational Safety and Health Administration's control of hazardous energy (lockout/tagout) standard on rates of machinery‐related fatal occupational injury.
Target population	**Occupation**: Mixed: industrial and construction/mixed
	**Industry**: All or mixed industries
	**Setting**: 888 All or mixed setting
	**Firm size**: Mixed firm size
Study design	**ITS** with comparison conditions: 4. Serial measures with external and internal control
	**Unit of analysis**: Group/organizational level
	**Sample size**: 3323 fatal machine‐related injuries in manufacturing, used estimates of nationwide US industry workers as denominator
Type of intervention	2.2.1 Legislative changes
Evaluation design	**Duration of intervention**: Standard went into effect and was permanent
	**Duration of follow‐up**: 4 = Long‐term (>36 months)
	**Type of outcome measure**: Injury
Study quality	High quality

**Table:** 

**Casteel, C (2004)** *Effectiveness of crime prevention through environmental design in reducing criminal activity in liquor stores: A pilot study*.	
Country	US
Aim	This study examines the effectiveness of a Crime Prevention Through Environmental Design intervention in reducing criminal activity in Santa Monica, California liquor stores.
Target population	**Occupation**: sales persons
	**Industry**: G—Wholesale and retail trade
	**Setting**: 040 tertiary activity area (such as office, teaching establishment, restaurant etc.), incl retail
	**Firm size**: Mixed firm size
Study design	**ITS** with comparison conditions: 6. Other (hybrid or combinations)
	**Unit of analysis**: Group/organizational level
	**Sample size**: 22 stores, liquor stores—9 intervention + 13 controls
Type of intervention	2.2.5 Administrative controls
Evaluation design	**Duration of intervention**: Intervention is ongoing beginning in 1996 and follow‐up after 2 years.
	**Duration of follow‐up**: 3 = Medium‐term (12–36 months)
	**Type of outcome measure**: Risk or behavior
Study quality	Low quality

**Table:** 

**Casteel, C (2009)** *Hospital employee assault rates before and after enactment of the California Hospital Safety and Security Act*. Study arm: Emergency units	
Country	US
Aim	Examine changes in violent event rates to hospital employees [here: emergency department and psychiatric unit employees] before and after enactment of the California Hospital Safety and Security Act in 1995.
Target population	**Occupation**: Social and healthcare
	**Industry**: Q—Human health and social work activities
	**Setting**: 050 Health establishment
	**Firm size**: 250+(large)
Study design	**ITS** with comparison conditions: 3. Serial measures with external control
	**Unit of analysis**: Group/organizational level
	**Sample size**: Hospitals, 95 intervention departments in California (62 and 93 emergency departments pre‐ and post‐enactment, respectively); 46 control departments in New Jersey (14 and 45 emergency departments pre‐ and post‐enactment, respectively) California post: 93
Type of intervention	2.2.1 Legislative changes
Evaluation design	**Duration of intervention:** Intervention was policy, permanent. California Hospital Safety and Security Act passed in 1993.
	**Duration of follow‐up**: 4 = Long‐term (>36 months)
	**Type of outcome measure**: Other
Study quality	Moderate quality

**Table:** 

**Casteel, C (2009)** *Hospital employee assault rates before and after enactment of the California Hospital Safety and Security Act*. Study arm: psychiatric units	
Country	US
Aim	Examine changes in violent event rates to hospital employees [here: psychiatric department employees] before and after enactment of the California Hospital Safety and Security Act in 1995.
Target population	**Occupation**: Social and healthcare
	**Industry**: Q—Human health and social work activities
	**Setting**: 050 Health establishment
	**Firm size**: 250+(large)
Study design	**ITS** with comparison conditions: 3. Serial measures with external control
	**Unit of analysis**: Group/organizational level
	**Sample size**: Hospitals, 95 intervention (62 and 93 emergency departments pre‐ and post‐enactment, respectively); 46 control (14 and 45 emergency departments pre‐ and post‐enactment, respectively):: California post: 31 dept + New Jersey (control) 26 dept = 57
Type of intervention	2.2.1 Legislative changes
Evaluation design	**Duration of intervention:** Intervention was policy, permanent. California Hospital Safety and Security Act passed in 1993.
	**Duration of follow‐up**: 4 = Long‐term (>36 months)
	**Type of outcome measure**: Other
Study quality	Moderate quality

**Table:** 

**Chapman, L (2011)** *A 7‐year intervention to increase adoption of safer dairy farming work practices*.	
Country	US
Aim	Assess social marketing techniques to increase awareness and adoption of safer farm practices (Wisconsin intervention and Maryland control group)
Target population	**Occupation**: Dairy farm managers; USA
	**Industry**: A—Agriculture, forestry and fishing
	**Setting**: 31 Farming and forestry
	**Firm size**: Unclear/not reported
Study design	**ITS** with comparison conditions: 2. Serial measures with internal control
	**Unit of analysis**: Group/organizational level
	**Sample size**: 600–350 farm managers each year. Social marketing effort to improve use of 3 safer and more profitables production practices including barn lights, silage bags, and calf feed mixing sites.
Type of intervention	2.2.8 Social marketing and other approaches
Evaluation design	**Duration of intervention:** 7 years
	**Duration of follow‐up**: 4 = Long‐term (>36 months)
	**Type of outcome measure**: Risk or behavior
Study quality	Low quality

**Table:** 

**Chhokar, R (2005)** *The 3‐year economic benefits of a ceiling lift intervention aimed to reduce healthcare worker injuries*.	
Country	CA
Aim	To determine whether the initial positive results reported by Ronald et al. (2002) and Spiegel et al. (2002) were representative of the longer‐term effectiveness of overhead lifts in reducing the risk of injury to nursing staff.
Target population	**Occupation**: human health activities (long‐term care facility)/dynamic
	**Industry**: Q—Human health and social work activities
	**Setting**: 050 Health establishment
	**Firm size**: Mixed firm size
Study design	**ITS** with comparison conditions: 2. Serial measures with internal control
	**Unit of analysis**: Group/organizational level
	**Sample size**: Analyses based on less than 50 injuries over 6 years period that involved patient handling (65 pre‐intervention, 47 post‐intervention) = 112
Type of intervention	3.2 Multifaceted at the group or organizational level
Evaluation design	**Duration of intervention:** 3 year intervention
	**Duration of follow‐up**: 3 = Medium‐term (12–36 months)
	**Type of outcome measure**: Injury
Study quality	Moderate quality

**Table:** 

**Cooper, M (1994)** *Reducing accidents using goal setting and feedback: A field study*.	
Country	UK
Aim	Assesses effect of goal‐setting and feedback on safety performance measures and injury incidence.
Target population	**Occupation**: Manufacture of rubber and plastic products; factory workers (cellophane film)/static
	**Industry**: C—Manufacturing
	**Setting**: 11 Industrial site
	**Firm size**: 250+(large)
Study design	**ITS** with comparison conditions: 1. Simple serial measures (No comparison)
	**Unit of analysis**: Group/organizational level
	**Sample size**: Overall: 540 employees from a 3‐shift production plant. (Semi‐structured interviews: 72 employees)
Type of intervention	2.1.1 Goal setting and feedback at group or organizational level
Evaluation design	**Duration of intervention:** 16 weeks
	**Duration of follow‐up**: 2 = Short‐term (≤12 months)
	**Type of outcome measure**: Injury
Study quality	Low quality

**Table:** 

**Cunningham, TR (2007)** *Using goal setting, task clarification, and feedback to increase the use of the hands‐free technique by hospital operating room staff*.	
Country	US
Aim	Evaluate the effects of a behavioral treatment on safe passing of sharps in the operation room.
Target population	**Occupation**: Operation room staff
	**Industry**: Q—Human health and social work activities
	**Setting**: 050 Health establishment
	**Firm size**: 50–249 (medium)
Study design	**ITS** with comparison conditions: 1. Simple serial measures (No comparison)
	**Unit of analysis**: Group/organizational level
	**Sample size**: 348 bed hospital serving 9 county regions in midwest US; input and output operation room.
Type of intervention	2.1.1 Goal setting and feedback at group or organizational level
Evaluation design	**Duration of intervention:** 24 sessions over 5.5 weeks In output units; 15 sessions over 4 weeks in input units.
	**Duration of follow‐up**: 2 = Short‐term (≤12 months)
	**Type of outcome measure**: Risk or behavior
Study quality	Moderate quality

**Table:** 

**Derr, JD (2001)** *Fatal falls in the US construction industry, 1990 to 1999*.	
Country	US
Aim	To determine whether the 1995 OSHA revisions of 29 CFR (Code of Federal Regulation) Part 1926 Subpart M had a measurable impact on the rate of fatal falls in the US construction industry by examining fatal fall rates over time.
Target population	**Occupation**: mixed construction workers
	**Industry**: F—Construction
	**Setting**: 020 Construction site
	**Firm size**: Mixed firm size
Study design	**ITS** with comparison conditions: 1. Simple serial measures (No comparison)
	**Unit of analysis**: Group/organizational level
	**Sample size**: 2353 fatalities from falls from elevation in US over 10‐yr period surrounding the legislation = 235/year
Type of intervention	2.2.1 Legislative changes
Evaluation design	**Duration of intervention:** Permanent once in place; revisions made to some sections like scaffolding, steel erections etc.
	**Duration of follow‐up**: 4 = Long‐term (>36 months)
	**Type of outcome measure**: Injury
Study quality	Low quality

**Table:** 

**Farina, E (2013)** *Are regulations effective in reducing construction injuries? An analysis of the Italian context*.	
Country	IT
Aim	To evaluate the impact on injury rates of the intervention plans developed to enforce the two Italian decrees relating to safety in the construction industry.
Target population	**Occupation**: Construction workers
	**Industry**: F—Construction
	**Setting**: 020 Construction site
	**Firm size**: Mixed firm size
Study design	**ITS** with comparison conditions: 3. Serial measures with external control
	**Unit of analysis**: Group/organizational level
	**Sample size**: Average 85,378 workers between 1994 and 2005 (12 years) reporting average 4674 injuries (eight regions in Italy).
Type of intervention	2.2.7 Enforcement of laws and regulations
Evaluation design	**Duration of intervention:** these laws remained in place >10 years.
	**Duration of follow‐up**: 4 = Long‐term (>36 months)
	**Type of outcome measure**: Injury
Study quality	Low quality

**Table:** 

**Fellner, DJ (1984)** *Increasing industrial safety practices and conditions through posted feedback*.	
Country	US
Aim	Examine the effects of posted feedback (positive and specific) for improving safety in a paper mill.
Target population	**Occupation**: paper products
	**Industry**: C—Manufacturing
	**Setting**: 010 Industrial site
	**Firm size**: 250+(large)
Study design	**ITS** with comparison conditions: 1. Simple serial measures (No comparison)
	**Unit of analysis**: Group/organizational level
	**Sample size**: 158 participants among 500 workers = 158
Type of intervention	2.1.1 Goal setting and feedback at group or organizational level
Evaluation design	**Duration of intervention:** 22 weeks
	**Duration of follow‐up**: 2 = Short‐term (≤12 months)
	**Type of outcome measure**: Injury
Study quality	Low quality

**Table:** 

**Fujishiro, K (2005)** *The effect of ergonomic interventions in healthcare facilities on musculoskeletal disorders*.	
Country	US
Aim	To evaluate effectiveness of a multi‐faceted program to prevent musculo‐skeletal disorders in long term care facilities primarily.
Target population	**Occupation**: Primarily long term care staff
	**Industry**: Q—Human health and social work activities
	**Setting**: 050 Health establishment
	**Firm size**: Mixed firm size
Study design	**ITS** with comparison conditions: 1. Simple serial measures (No comparison)
	**Unit of analysis**: Group/organizational level
	**Sample size**: 100 work units from 85 nursing homes, 11 MR/DD (Mental Retardation and Other Developmental Disability) facilities, and 3 hospitals
Type of intervention	3.2 Multifaceted at the group or organizational level
Evaluation design	**Duration of intervention:** Changes could be permanent or not based on what facility does… made assistance available and funds to purchase equipment
	**Duration of follow‐up**: 3 = Medium‐term (12–36 months)
	**Type of outcome measure**: Injury
Study quality	Low quality

**Table:** 

**Garg, A (1999)** *Long‐term effectiveness of “Zero‐Lift Program” in seven nursing homes and one hospital*.	
Country	US
Aim	To reduce injuries to health care workers resulting from annual lifting and transferring of patients. Zero‐lift programs were established in 7 nursing homes and one hospital through employee‐management advisory teams.
Target population	**Occupation**: health care—7 nursing homes and 1 hospital
	**Industry**: Q—Human health and social work activities
	**Setting**: 050 Health establishment
	**Firm size**: 50–249 (medium)
Study design	**ITS** with comparison conditions: 1. Simple serial measures (No comparison)
	**Unit of analysis**: Group/organizational level
	**Sample size**: 8 facilities ‐‐‐ mean beds=145 and mean personnel=94 per facility
Type of intervention	3.3 Multifaceted across individual and organizational levels
Evaluation design	**Duration of intervention:** Ongoing once initiated; equipment remained
	**Duration of follow‐up**: 4 = Long‐term (>36 months)
	**Type of outcome measure**: Injury
Study quality	Low quality

**Table:** 

**Gershon, R (1999)** *The impact of multifocused interventions on sharps injury rates at an acute‐care hospital*.	
Country	US
Aim	To determine the impact of a multifocused interventional program on sharps injury rates.
Target population	**Occupation**: Human health activities/dynamic
	**Industry**: Q—Human health and social work activities
	**Setting**: 050 Health establishment
	**Firm size**: 250+(large)
Study design	**ITS** with comparison conditions: 1. Simple serial measures (No comparison)
	**Unit of analysis**: Group/organizational level
	**Sample size**: 2300 hospital employees average per year (average 1500 full‐time equivalents/year); total of 693 sharps injuries reported in 9 year period
Type of intervention	3.2 Multifaceted at the group or organizational level
Evaluation design	**Duration of intervention:** 6 years (1st year considered implementation); engineering changes permanent after 2 month phase in
	**Duration of follow‐up**: 4 = Long‐term (>36 months)
	**Type of outcome measure**: Injury
Study quality	Moderate quality

**Table:** 

**Kuehl, KS (2013)** *Economic benefit of the PHLAME wellness programme on firefighter injury*.		
Country	US	
Aim	To evaluate the impact of a workplace health promotion program on workers' compensation claims and medical costs among Oregon fire departments participating in the PHLAME health promotion programme compared to the Oregon fire departments.	
Target population	**Occupation**: Firefighters/dynamic	
	**Industry**: O—Public administration and defence	
	**Setting**: 130 Emergency, rescuing and military sites	
	**Firm size**: 50–249 (medium)	
Study design	**ITS** with comparison conditions: 3. Serial measures with external control	
	**Unit of analysis**: Group/organizational level	
	**Sample size**: Two large urban fire departments with program compared to two large without, matched for similar characteristics. Including pre intervention injury rates and size of force. N=1369	
Type of intervention	1.3.1 Individual physical training	
Evaluation design	**Duration of intervention:** Team effort was 12 peer‐led sessions for 45 min	
	**Duration of follow‐up**: 4 = Long‐term (>36 months)	
	**Type of outcome measure**: Injury	
Study quality	Low quality

**Table:** 

**Lawrence, L (1997)** *The effectiveness of a needleless intravenous connection system: An assessment by injury rate and user satisfaction*.	
Country	US
Aim	To assess impact of needleless intravenous connection system on rate of reported percutaneous injuries from intravenous connections.
Target population	**Occupation**: hospital workers clinical—nurses
	**Industry**: Q—Human health and social work activities
	**Setting**: 050 Health establishment
	**Firm size**: 250+(large)
Study design	**ITS** with comparison conditions: 2. Serial measures with internal control
	**Unit of analysis**: Group/organizational level
	**Sample size**: Two tertiary care teaching hospitals, one general and one pediatric hospital: 1989–1991:7296 full‐time equivalents (FTE)—1993: 3370 FTE + Pediatric Hospital: 1989–1991: 4320 FTE—1993: 2476 FTE = 17,462/4y = 4365/y
Type of intervention	2.2.4 Engineering controls
Evaluation design	**Duration of intervention:** Not reported or unclear
	**Duration of follow‐up**: 2 = Short‐term (≤12 months)
	**Type of outcome measure**: Injury
Study quality	Low quality

**Table:** 

**Lipscomb, H (2003)** *Work‐related falls among union carpenters in Washington State before and after the Vertical Fall Arrest Standard*.	
Country	US
Aim	Evaluated changes in the rate of falls from elevations and measures of severity among a large cohort of union carpenters after the vertical fall standard change in Washington State, taking into account the temporal trends in their overall injury rates.
Target population	**Occupation**: Construction; carpenters. Specialized construction activities/dynamic.
	**Industry**: F—Construction
	**Setting**: 020 construction site
	**Firm size**: Mixed firm size
Study design	**ITS** with comparison conditions: 6. Other (hybrid or combinations)
	**Unit of analysis**: Group/organizational level
	**Sample size**: 16,215 carpenters;102 million work hours
Type of intervention	2.2.1 Legislative changes
Evaluation design	**Duration of intervention:** Policy went into effect and remained from February 1991.
	**Duration of follow‐up**: 4 = Long‐term (>36 months)
	**Type of outcome measure**: Injury
Study quality	High quality

**Table:** 

**Lipscomb, HJ (2010)** *Continued progress in the prevention of nail gun injuries among apprentice carpenters: what will it take to see wider spread injury reductions?*	
Country	US
Aim	To add an additional follow‐up year to data previously reported on the effect of safer sequential triggers and training on nail gun injuries in apprentice carpenters.
Target population	**Occupation**: Specialized construction activities; carpenters
	**Industry**: F—Construction
	**Setting**: 020 Construction site
	**Firm size**: Mixed firm size
Study design	**ITS** with comparison conditions: 6. Other (hybrid or combinations)
	**Unit of analysis**: Group/organizational level
	**Sample size**: Additional 464 carpenters added to those above [Lipscomb et al., 2008]; included 259 with 276,294 work hours. 654 + 818 + 490 + 464/4 = 606,5
Type of intervention	3.3 Multifaceted across individual and organizational levels
Evaluation design	**Duration of intervention:** Training in nail gun use started in apprenticeship school program, sequential trigger use phased in with gradual adoption. Both exposures monitored over time.
	**Duration of follow‐up**: 4 = Long‐term (>36 months)
	**Type of outcome measure**: Injury
Study quality	Moderate quality

**Table:** 

**Lopez‐Ruiz, M (2014)** *Impact of road safety interventions on traffic‐related occupational injuries in Spain*. Study arm: Revised penalty code	
Country	ES
Aim	To evaluate the impact of road safety interventions, including the penalty point system (PPS) and the reformed Spanish penal code (RPC), on traffic‐related occupational injuries.
Target population	**Occupation**: ALL
	**Industry**: H—Transporting and storage
	**Setting**: 060 Public area (postal courrier)
	**Firm size**: Mixed firm size
Study design	**ITS** with comparison conditions: 3. Serial measures with external control
	**Unit of analysis**: Group/organizational level
	**Sample size**: all salaried workers in Spain covered by social security occupational injury benefits; 110,834,882 salaried workers with 468,000+ traffic related injuries
Type of intervention	2.2.7 Enforcement of laws and regulations
Evaluation design	**Duration of intervention:** RPC introduced in Dec 2007. (PPS introduced in July 2006)
	**Duration of follow‐up**: 4 = Long‐term (>36 months)
	**Type of outcome measure**: Injury
Study quality	Low quality

**Table:** 

**Lopez‐Ruiz, M (2014)** *Impact of road safety interventions on traffic‐related occupational injuries in Spain*. Study arm: Penalty point system	
Country	ES
Aim	To evaluate the impact of road safety interventions, including the penalty point system (PPS) and the reformed Spanish penal code (RPC), on traffic‐related occupational injuries.
Target population	**Occupation**: ALL
	**Industry**: H—Transporting and storage
	**Setting**: 060 Public area (postal courrier)
	**Firm size**: Mixed firm size
Study design	**ITS** with comparison conditions: 3. Serial measures with external control
	**Unit of analysis**: Group/organizational level
	**Sample size**: All salaried workers in Spain covered by social security occupational injury benefits; 110,834,882 salaried workers with 468,000+ traffic related injuries
Type of intervention	2.2.7 Enforcement of laws and regulations
Evaluation design	**Duration of intervention:** PPS introduced in July 2006 (RPC introduced in December 2007)
	**Duration of follow‐up**: 4 = Long‐term (>36 months)
	**Type of outcome measure**: Injury
Study quality	Low quality

**Table:** 

**Mancini, G (2005)** *Prevention of work‐related eye injuries: long term assessment of the effectiveness of a multicomponent intervention among metal workers*.	
Country	IT
Aim	To investigate long‐term effectiveness of a multi‐component intervention to prevent work related eye injuries among metal workers.
Target population	**Occupation**: Metal workers factory/static
	**Industry**: C—Manufacturing
	**Setting**: 010 Industrial site
	**Firm size**: 10–49 (small)
Study design	**ITS** with comparison conditions: 4. Serial measures with external and internal control
	**Unit of analysis**: Group/organizational level
	**Sample size**: All shops and workers in region used for long term evaluation from surveillance data, including 237 metalware factories
Type of intervention	3.3 Multifaceted across individual and organizational levels
Evaluation design	**Duration of intervention:** Intervention triggered by surveillance system noting eye injury prevalence in 1988.
	**Duration of follow‐up**: 4 = Long‐term (>36 months)
	**Type of outcome measure**: Injury
Study quality	High quality

**Table:** 

**Marlenga, B (2006)** *Evaluation of a policy to reduce youth tractor crashes on public roads*.	
Country	USA
Aim	Evaluate the effectiveness of a US state law in Wisconsin in reducing highway tractor crashes involving youth operators.
Target population	**Occupation**: Agriculture—youth tractor driving on public roads
	**Industry**: A—Agriculture, forestry and fishing
	**Setting**: 030 Farming and forestry
	**Firm size**: Mixed firm size
Study design	**ITS** with comparison conditions: 1. Simple serial measures (No comparison)
	**Unit of analysis**: Group/organizational level
	**Sample size**: 146 tractor crashes, although most of report focuses on 134 involving 12–15 year olds
Type of intervention	2.2.1 Legislative changes
Evaluation design	**Duration of intervention:** Enacted state law in 1994 that went into effect in July 1997. Permanent after that
	**Duration of follow‐up**: 4 = Long‐term (>36 months)
	**Type of outcome measure**: Injury
Study quality	Low quality

**Table:** 

**Martin, P (2009)** *Effect of a nurse back injury prevention intervention on the rate of injury compensation claims*.	
Country	AU
Aim	To evaluate the effect of introducing a No Lift Policy on back injuries to nurses across an entire health system
Target population	**Occupation**: Human health and activities/dynamic
	**Industry**: Q—Human health and social work activities
	**Setting**: 050 Health establishment
	**Firm size**: 250+(large)
Study design	**ITS** with comparison conditions: 2. Serial measures with internal control
	**Unit of analysis**: Group/organizational level
	**Sample size**: Approximately 15,000 nurse full‐time equivalents each year
Type of intervention	3.3 Multifaceted across individual and organizational levels
Evaluation design	**Duration of intervention:** Initiated in 1998 and phased in; they considered implementation period from September 98 to December 2000. Effects should be sustained after phase in period; nothing removed.
	**Duration of follow‐up**: 3 = Medium‐term (12–36 months)
	**Type of outcome measure**: Injury
Study quality	High quality

**Table:** 

**Menendez, CC (2012)** *Evaluation of a nationally funded state‐based programme to reduce fatal occupational injuries*.	
Country	US
Aim	To investigate the impact of the state‐based FACE programme on two focus areas of falls from height and electrocutions.
Target population	**Occupation**: ALL
	**Industry**: All or mixed industries
	**Setting**: 888 All or mixed setting
	**Firm size**: Mixed firm size
Study design	**ITS** with comparison conditions: 3. Serial measures with external control
	**Unit of analysis**: Group/organizational level
	**Sample size**: Falls: 20 states had participation at some point. Electrocutions: 14 states had participation. 12,781 fall‐related deaths and 7709 electrocution‐related deaths over 22 years = 931/year.
Type of intervention	3.9 Multifaceted safety interventions not listed above
Evaluation design	**Duration of intervention:** Variable by state, duration allowed to vary in analyses. Main effect was state participation or not for each year.
	**Duration of follow‐up**: 4 = Long‐term (>36 months)
	**Type of outcome measure**: Injury
Study quality	Low quality

**Table:** 

**Miller, TR (2007)** *Effectiveness and benefit‐cost of peer‐based workplace substance abuse prevention coupled with random testing*.	
Country	US
Aim	Estimate the effectiveness and benefit‐cost ratio of a peer‐based substance abuse prevention program at a U.S. transportation company, implemented in phases from 1988 to 1990.
Target population	**Occupation**: Transportation and storage/mixed
	**Industry**: H—Transporting and storage
	**Setting**: 060 Public area
	**Firm size**: 250+(large)
Study design	**ITS** with comparison conditions: 3. Serial measures with external control
	**Unit of analysis**: Group/organizational level
	**Sample size**: 26,000 employees in company ‐‐ not provided overtime
Type of intervention	3.3 Multifaceted across individual and organizational levels
Evaluation design	**Duration of intervention:** implemented in phases from 1988 to 1990 and then drug testing federal mandate in 1994
	**Duration of follow‐up**: 4 = Long‐term (>36 months)
	**Type of outcome measure**: Injury
Study quality	Moderate quality

**Table:** 

**Mode, NA (2012)** *A multifaceted public health approach to statewide aviation safety*.	
Country	US
Aim	Assess effectiveness of a multifaceted public health initiative focused on Alaskan air taxi/commuter operations, including risk factor identification, improved weather information access, formation of industry‐led safety organization.
Target population	**Occupation**: Dynamic (highly dynamic)
	**Industry**: H—Transporting and storage
	**Setting**: 090 In the air, elevated except construction
	**Firm size**: Mixed firm size
Study design	**ITS** with comparison conditions: 3. Serial measures with external control
	**Unit of analysis**: Group/organizational level
	**Sample size**: State of Alaska aircraft crashes over 20 years: 2807 = 140/year
Type of intervention	3.3 Multifaceted across individual and organizational levels
Evaluation design	**Duration of intervention:** Initially launched in 2000 and continued but the various components of the intervention occurred over a number of years
	**Duration of follow‐up**: 4 = Long‐term (>36 months)
	**Type of outcome measure**: Injury
Study quality	Low quality

**Table:** 

**Monforton, C (2010)** *An impact evaluation of a federal mine safety training regulation on injury rates among US stone, sand, and gravel mine workers: an interrupted time‐series analysis*.	
Country	US
Aim	Assess impact of the mandatory worker safety policy and training regulations issued Sept 1999 by US MSHA (Mine Safety and Health Administration) on injury rates
Target population	**Occupation**: Mine workers‐ stone, sand, gravel/dynamic
	**Industry**: B—Mining and quarrying
	**Setting**: 010 Industrial site
	**Firm size**: Mixed firm size
Study design	**ITS** with comparison conditions: 5. Serial measures with stratified analyses (allowing comparisons)
	**Unit of analysis**: Group/organizational level
	**Sample size**: 7998 mines reporting person‐hours of work to allow quarterly rate calculations.
Type of intervention	2.2.1 Legislative changes
Evaluation design	**Duration of intervention:** Permanent requirement changed for training with one year phase in period (Government regulation)
	**Duration of follow‐up**: 4 = Long‐term (>36 months)
	**Type of outcome measure**: Injury
Study quality	High quality

**Table:** 

**Mujuru, P (2009)** *Evaluating the impact of an intervention to reduce injuries among loggers in West Virginia, 1999–2007*.	
Country	US
Aim	To evaluate effectiveness of a video‐based safety raining in reducing logging injuries over an 8‐yr period.
Target population	**Occupation**: loggers/dynamic
	**Industry**: A—Agriculture, forestry and fishing
	**Setting**: 030 Farming and forestry
	**Firm size**: Mixed firm size
Study design	**ITS** with comparison conditions: 1. Simple serial measures (No comparison)
	**Unit of analysis**: Group/organizational level
	**Sample size**: 1535 loggers (mean)employed each year—analysis on industry level
Type of intervention	1.1.3 Teaching, education to increase knowledge and awareness
Evaluation design	**Duration of intervention:** the video training lasts 14 min
	**Duration of follow‐up**: 3 = Medium‐term (12–36 months)
	**Type of outcome measure**: Injury
Study quality	Low quality

**Table:** 

**Park, R (2009)** *Impact of publicly sponsored interventions on musculoskeletal injury claims in nursing homes*.	
Country	US
Aim	Evaluate the impact of Ohio Bureau of Workers' Compensation interventions on back injury claim rates 1995–2004 for all Ohio nursing homes.
Target population	**Occupation**: nursing home staff/dynamic
	**Industry**: Q—Human health and social work activities
	**Setting**: 050 Health establishment
	**Firm size**: Mixed firm size
Study design	**ITS** with comparison conditions: 6. Other (hybrid or combinations)
	**Unit of analysis**: Group/organizational level
	**Sample size**: Retrospective cohort analyses using administrative data of all nursing homes in the state of Ohio, USA 1995–2004, subset of analyses looked at effects of staffing and patient acuity on injury rates. Over 1000 employers included with over 652 million work
Type of intervention	3.2 Multifaceted at the group or organizational level
Evaluation design	**Duration of intervention:** Promotion of policy was for two years 2000–2001, effects would be potentially longer standing.
	**Duration of follow‐up**: 3 = Medium‐term (12–36 months)
	**Type of outcome measure**: Injury
Study quality	High quality

**Table:** 

**Passfield, J (2003)** *“No lift” patient handling policy implementation and staff injury rates in a public hospital*.	
Country	AU
Aim	To assess the effect of a "No lift" patient handling policy implemented in a hospital.
Target population	**Occupation**: Human health activities; nurses/dynamic
	**Industry**: Q—Human health and social work activities
	**Setting**: 050 Health establishment
	**Firm size**: 250+(large)
Study design	**ITS** with comparison conditions: 2. Serial measures with internal control
	**Unit of analysis**: Group/organizational level
	**Sample size**: 92 injury claims (44 claims pre‐training + 26 claims post‐training = 70 claims?)
Type of intervention	3.3 Multifaceted across individual and organizational levels
Evaluation design	**Duration of intervention:** 23 months
	**Duration of follow‐up**: 3 = Medium‐term (12–36 months)
	**Type of outcome measure**: Injury
Study quality	High quality

**Table:** 

**Phillips, EK (2012)** *Percutaneous injuries before and after the Needlestick Safety and Prevention Act*.	
Country	US
Aim	To determine whether the Needlestick Safety and Prevention Act (NSPA) had an effect on the rate of percutaneous injuries among hospital employees.
Target population	**Occupation**: health care workers in hospitals
	**Industry**: Q—Human health and social work activities
	**Setting**: 050 Health establishment
	**Firm size**: 250+(large)
Study design	**ITS** with comparison conditions: 1. Simple serial measures (No comparison)
	**Unit of analysis**: Group/organizational level
	**Sample size**: 85 hospitals with 23,908 injuries from 1995 to 2005 = 2173/year
Type of intervention	2.2.7 Enforcement of laws and regulations
Evaluation design	**Duration of intervention:** Ongoing once Prevention Act in place.
	**Duration of follow‐up**: 4 = Long‐term (>36 months)
	**Type of outcome measure**: Injury
Study quality	Low quality

**Table:** 

**Porru, S (2011)** *An effectiveness evaluation of a multifaceted preventive intervention on occupational injuries in foundries: a 13‐Year follow‐up study with interrupted times series analysis*.	
Country	IT
Aim	Evaluation of a multifaceted intervention to prevent occupational injuries was carried out in two foundries.
Target population	**Occupation**: Foundries; one cast‐iron and one non‐ferrous
	**Industry**: C—Manufacturing
	**Setting**: 010 Industrial site
	**Firm size**: 50–249 (medium)
Study design	**ITS** with comparison conditions: 1. Simple serial measures (No comparison)
	**Unit of analysis**: Group/organizational level
	**Sample size**: Two businesses, 230 and 50 employees respectively. Total=280
Type of intervention	3.3 Multifaceted across individual and organizational levels
Evaluation design	**Duration of intervention:** Ongoing in 2000–2006 (last follow‐up), investigators considered 2000–2002 pre‐intervention period.
	**Duration of follow‐up**: 4 = Long‐term (>36 months)
	**Type of outcome measure**: Injury
Study quality	Low quality

**Table:** 

**Prezant, D (1999)** *Impact of a modern firefighting protective uniform on the incidence and severity of burn injuries in New York City firefighters*.	
Country	USA
Aim	To determine the impact of the modern uniform on the incidence and severity of firefighter (FDNY) burn injuries.
Target population	**Occupation**: Public administration and defense; compulsory social security… fire fighters
	**Industry**: O—Public administration and defence
	**Setting**: 130 Emergency, rescuing and military sites
	**Firm size**: 250+(large)
Study design	**ITS** with comparison conditions: 1. Simple serial measures (No comparison)
	**Unit of analysis**: Group/organizational level
	**Sample size**: 11,000 firefighters
Type of intervention	2.2.4 Engineering controls
Evaluation design	**Duration of intervention:** Two years, protective uniform changes.
	**Duration of follow‐up**: 3 = Medium‐term (12–36 months)
	**Type of outcome measure**: Injury
Study quality	Moderate quality

**Table:** 

**Rautiainen, R (2005)** *Effects of premium discount on workers' compensation claims in agriculture in Finland*.	
Country	FI
Aim	Evaluate changes in injury claim rates after a premium discount program was implemented in the Finnish farmers' workers' compensation insurance.
Target population	**Occupation**: Crop and animal production, hunting and related service activities/dynamic
	**Industry**: A—Agriculture, forestry and fishing
	**Setting**: 030 Farming and forestry
	**Firm size**: Mixed firm size
Study design	**ITS** with comparison conditions: 5. Serial measures with stratified analyses (allowing comparisons)
	**Unit of analysis**: Group/organizational level
	**Sample size**: 132,134 injury claims from 1990 to 2003, population ranged from 220,000 to 110,000 over time = 9438/year
Type of intervention	2.2.2 Economic incentives
Evaluation design	**Duration of intervention:** 6.5 years, premium discounts implemented and kept throughout
	**Duration of follow‐up**: 4 = Long‐term (>36 months)
	**Type of outcome measure**: Injury
Study quality	High quality

**Table:** 

**Reddy, S (2001)** *Assessing the effect of long‐term availability of engineering controls on needlestick injuries among health care workers: A 3‐year preimplementation and postimplementation comparison*.	
Country	US
Aim	Assess whether engineering controls reduced needlestick injuries.
Target population	**Occupation**: healthcare workers; nurses and ancillary
	**Industry**: Q—Human health and social work activities
	**Setting**: 51 Health establishment
	**Firm size**: 250+(large)
Study design	**ITS** with comparison conditions: 1. Simple serial measures (No comparison)
	**Unit of analysis**: Group/organizational level
	**Sample size**: 7003 full‐time equivalents with 513 injuries
Type of intervention	2.2.4 Engineering controls
Evaluation design	**Duration of intervention:** Engineering controls, in place all three follow‐up years, BUT workers also had access to older devices (to be used in event newer ones could not be used).
	**Duration of follow‐up**: 3 = Medium‐term (12–36 months)
	**Type of outcome measure**: Injury
Study quality	Low quality

**Table:** 

**Rogues, A (2004)** *Impact of safety devices for preventing percutaneous injuries related to phlebotomy procedures in health care workers*.	
Country	FR
Aim	To determine the effectiveness of 2 protective devices in preventing needlestick injuries to health care workers.
Target population	**Occupation**: Hospital workers/dynamic
	**Industry**: Q—Human health and social work activities
	**Setting**: 050 Health establishment
	**Firm size**: 250+(large)
Study design	**ITS** with comparison conditions: 1. Simple serial measures (No comparison)
	**Unit of analysis**: Group/organizational level
	**Sample size**: 2900 nurses/8500 full‐time equivalent employees
Type of intervention	2.2.4 Engineering controls
Evaluation design	**Duration of intervention:** Permanent device changes. Old devices removed when new introduced.
	**Duration of follow‐up**: 3 = Medium‐term (12–36 months)
	**Type of outcome measure**: Injury
Study quality	High quality

**Table:** 

**Saari, J (1989)** *The effect of positive feedback on industrial housekeeping and accidents; A long‐term study at a shipyard*.	
Country	FI
Aim	To determine effects of performance feedback on housekeeping behavior and on injuries over a longer period.
Target population	**Occupation**: Manufacture of other transport equipment; shipyard workers/both
	**Industry**: C—Manufacturing
	**Setting**: 11 Industrial site
	**Firm size**: 10–49 (small)
Study design	**ITS** with comparison conditions: 2. Serial measures with internal control
	**Unit of analysis**: Group/organizational level
	**Sample size**: 64 workers, four foremen, two production engineers; multiple housekeeping indicators collected as an index.
Type of intervention	3.3 Multifaceted across individual and organizational levels
Evaluation design	**Duration of intervention:** Feedback for 8 weeks
	**Duration of follow‐up**: 3 = Medium‐term (12–36 months)
	**Type of outcome measure**: Injury
Study quality	Moderate quality

**Table:** 

**Schoenfisch, AL (2013)** *Musculoskeletal injuries among hospital patient care staff before and after implementation of patient lift and transfer equipment*. Study arm: Medical Center	
Country	US
Aim	Evaluate rates of MS injuries among patient care staff at a medical center and community hospital in the United States over 13 years, during which time a “minimal manual lift" policy and mechanical lift equipment were implemented.
Target population	**Occupation**: Healthcare, largely nurses—hospital employees/dynamic
	**Industry**: Q—Human health and social work activities
	**Setting**: 050 Health establishment
	**Firm size**: 250+(large)
Study design	**ITS** with comparison conditions: 2. Serial measures with internal control
	**Unit of analysis**: Group/organizational level
	**Sample size**: 11,545 patient care staff contributing 28,446 full‐time equivalents over 13 years. 1543 patient handling injuries and 613 non‐patient handling injuries.
Type of intervention	2.2.4 Engineering controls
Evaluation design	**Duration of intervention:** Total observation = 13 years; post IV = 4+ years post initiation of the IV
	**Duration of follow‐up**: 4 = Long‐term (>36 months)
	**Type of outcome measure**: Injury
Study quality	Low quality

**Table:** 

**Schoenfisch, AL (2013)** *Musculoskeletal injuries among hospital patient care staff before and after implementation of patient lift and transfer equipment*. Study arm: Community Hospital	
Country	US
Aim	Evaluate rates of musculoskeletal (MS) injuries among patient care staff at a medical center and community hospital in the United States over 13 years, during which time a “minimal manual lift” policy and mechanical lift equipment were implemented.
Target population	**Occupation**: Healthcare, largely nurses—hospital employees/dynamic
	**Industry**: Q—Human health and social work activities
	**Setting**: 050 Health establishment
	**Firm size**: 250+(large)
Study design	**ITS** with comparison conditions: 2. Serial measures with internal control
	**Unit of analysis**: Group/organizational level
	**Sample size**: 11,545 patient care staff contributing 28,446 full‐time equivalents over 13 years. 1543 patient handling injuries and 613 non‐patient handling injuries.
Type of intervention	2.2.4 Engineering controls
Evaluation design	**Duration of intervention:** Total observation = 13 years
	**Duration of follow‐up**: 4 = Long‐term (>36 months)
	**Type of outcome measure**: Injury
Study quality	Low quality

**Table:** 

**Smollen, P (2004)** *Evaluation of a programme designed to reduce occupational exposures from steel‐winged butterfly needles in the clinical setting*.	
Country	Australia
Aim	Evaluation of a programme devised to reduce the occupational exposures from steel‐winged butterfly needles for all hospital staff in the clinical setting.
Target population	**Occupation**: hospital/dynamic
	**Industry**: Q—Human health and social work activities
	**Setting**: 51 Health establishment
	**Firm size**: 250+(large)
Study design	**ITS** with comparison conditions: 2. Serial measures with internal control
	**Unit of analysis**: Group/organizational level
	**Sample size**: 400‐bed hospital: rates expressed per full‐time equivalents (but never told number of full‐time equivalents. needlestick injuries—Total/Butterfly needlestick injuries: Before: 92/26—Intervention programme: 71/4: total 163/butterfly 30 = 163
Type of intervention	2.2.4 Engineering controls
Evaluation design	**Duration of intervention:** 2+ years
	**Duration of follow‐up**: 3 = Medium‐term (12–36 months)
	**Type of outcome measure**: Injury
Study quality	Moderate quality

**Table:** 

**Sossai, D (2010)** *Using an intravenous catheter system to prevent needlestick injury*.	
Country	IT
Aim	To identify the effects of sharps awareness campaign and safety catheter use on the annual incidence rate of needlestick injuries 2003–2007.
Target population	**Occupation**: health care workers—hospital/dynamic
	**Industry**: Q—Human health and social work activities
	**Setting**: 51 Health establishment
	**Firm size**: 250+(large)
Study design	**ITS** with comparison conditions: 2. Serial measures with internal control
	**Unit of analysis**: Group/organizational level
	**Sample size**: 4200–4500 employees each year.
Type of intervention	2.2.4 Engineering controls
Evaluation design	**Duration of intervention:** Introduced catheters and they remained in place through two years of follow‐up; phased in at higher risk areas first and all by end of study period
	**Duration of follow‐up**: 4 = Long‐term (>36 months)
	**Type of outcome measure**: Injury
Study quality	Low quality

**Table:** 

**Spangenberg, S (2002)** *The construction of the Øresund Link between Denmark and Sweden: The effect of a multi‐faceted safety campaign*.	
Country	DK
Aim	Evaluate the effect of a safety campaign implemented midway in the construction of a railway/road link between Denmark and Sweden, using data from the Danish land works side.
Target population	**Occupation**: Construction work
	**Industry**: F—Construction
	**Setting**: 020 Construction site
	**Firm size**: Unclear/not reported
Study design	**ITS** with comparison conditions: 1. Simple serial measures (No comparison)
	**Unit of analysis**: Group/organizational level
	**Sample size**: 4250 person‐years. Intervention at firm level (construction site), and data aggregated to firm level.
Type of intervention	3.3 Multifaceted across individual and organizational levels
Evaluation design	**Duration of intervention:** 3 years
	**Duration of follow‐up**: 3 = Medium‐term (12–36 months)
	**Type of outcome measure**: Injury
Study quality	High quality

**Table:** 

**Sulzer‐Azaroff, B (1990)** *Improving occupational safety in a large industrial plant*.	
Country	US
Aim	Replication of the injury prevention model previously developed (by this team) was conducted in a large industrial plant to assess generality and to measure effect of targeting safety behaviors on accidents and lost time injuries.
Target population	**Occupation**: Electrical manufacturing workers
	**Industry**: C—Manufacturing
	**Setting**: 11 Industrial site
	**Firm size**: 250+(large)
Study design	**ITS** with comparison conditions: 1. Simple serial measures (No comparison)
	**Unit of analysis**: Group/organizational level
	**Sample size**: 200–250 employees out of 3300
Type of intervention	2.1.1 Goal setting and feedback at group or organizational level
Evaluation design	**Duration of intervention:** 24 weeks
	**Duration of follow‐up**: 2 = Short‐term (≤12 months)
	**Type of outcome measure**: Risk or behavior
Study quality	Low quality

**Table:** 

**Sulzer‐Azaroff, B (1980)** *Manufacturing safety hazard reduction through performance feedback*	
Country	UK
Aim	To analyze the reliability and generality of the feedback in reducing safety hazards during the experiment and follow‐up, and to determine if the intervention and any correlated improvement would persist following formal termination of the study.
Target population	**Occupation**: Manufacturing—not classified further
	**Industry**: C—Manufacturing
	**Setting**: 010 Industrial site
	**Firm size**: Mixed firm size
Study design	**ITS** with comparison conditions: 1. Simple serial measures (No comparison)
	**Unit of analysis**: Group/organizational level
	**Sample size**: Six production supervisors (four female + two male), 128 workers (115 according to Table 1)
Type of intervention	2.1.1 Goal setting and feedback at group or organizational level
Evaluation design	**Duration of intervention:** Variable by department, up to 4 months.
	**Duration of follow‐up**: 2 = Short‐term (≤12 months)
	**Type of outcome measure**: Risk or behavior
Study quality	Low quality

**Table:** 

**Suruda, A (2002)** *Impact of the OSHA trench and excavation standard on fatal injury in the construction industry*.	
Country	US
Aim	We examined fatal injuries from trench cave‐in in the construction industry for 5 year periods before and after the revision in the 47 US states for which data were available for both periods.
Target population	**Occupation**: Construction Industry wide
	**Industry**: F—Construction
	**Setting**: 020 Construction site
	**Firm size**: Mixed firm size
Study design	**ITS** with comparison conditions: 1. Simple serial measures (No comparison)
	**Unit of analysis**: Group/organizational level
	**Sample size**: 522 fatalities over 11 years pre and post (47 pr. year)
Type of intervention	2.2.1 Legislative changes
Evaluation design	**Duration of intervention:** Permanent trench standard, evaluated 5.75 years pre (4/1984–12/1989) and 6 years post (1/1990–12/1995).
	**Duration of follow‐up**: 4 = Long‐term (>36 months)
	**Type of outcome measure**: Injury
Study quality	Low quality

**Table:** 

**Whitby, M (2008)** *Needlestick injuries in a major teaching hospital: the worthwhile effect of hospital‐wide replacement of conventional hollow‐bore needles*.	
Country	AU
Aim	Evaluate the impact on needlestick injury rates after substantial replacement of conventional hollow‐bore needles with safety‐engineered devices including retractable syringes, needle‐free intravenous systems, and safety winged butterfly needles.
Target population	**Occupation**: Human health activities/dynamic
	**Industry**: Q—Human health and social work activities
	**Setting**: 050 Health establishment
	**Firm size**: 250+(large)
Study design	**ITS** with comparison conditions: 2. Serial measures with internal control
	**Unit of analysis**: Group/organizational level
	**Sample size**: 20,650 full‐time equivalents (FTEs) included 6500 post intervention
Type of intervention	2.2.4 Engineering controls
Evaluation design	**Duration of intervention:** permanent use of safety‐engineered devices after introduction
	**Duration of follow‐up**: 3 = Medium‐term (12–36 months)
	**Type of outcome measure**: Injury
Study quality	High quality

**Table:** 

**Wickizer, T (2004)** *Do drug‐free workplace programs prevent occuptional injuries? Evidence from Washington State*.	
Country	US
Aim	To evaluate the effect of publicly sponsored drug‐free workplace program on reducing the risk of occupational injuries.
Target population	**Occupation**: Multiple/mixed
	**Industry**: All or mixed industries
	**Setting**: 888 All or mixed setting
	**Firm size**: Mixed firm size
Study design	**ITS** with comparison conditions: 3. Serial measures with external control
	**Unit of analysis**: Group/organizational level
	**Sample size**: 261 intervention companies; 20,500 nonequivalent control companies
Type of intervention	3.3 Multifaceted across individual and organizational levels
Evaluation design	**Duration of intervention:** Ongoing program in company once initiated
	**Duration of follow‐up**: 3 = Medium‐term (12–36 months)
	**Type of outcome measure**: Injury
Study quality	High quality

**Table:** 

**Zafar, AB (1997)** *Effect of a comprehensive program to reduce needlestick injuries*.	
Country	US
Aim	To reduce needlestick injuries (NSIs) and to assess the effectiveness of the interventions.
Target population	**Occupation**: Human health activities
	**Industry**: Q—Human health and social work activities
	**Setting**: 050 Health establishment
	**Firm size**: 250+(large)
Study design	**ITS** with comparison conditions: 1. Simple serial measures (No comparison)
	**Unit of analysis**: Group/organizational level
	**Sample size**: 499 injuries over a 7 year period, 1500 health care workers.
Type of intervention	3.2 Multifaceted at the group or organizational level
Evaluation design	**Duration of intervention:** 4 years
	**Duration of follow‐up**: 4 = Long‐term (>36 months)
	**Type of outcome measure**: Injury
Study quality	Moderate quality

**Table:** 

**Zohar, D (2003)** *The use of supervisory practices as leverage to improve safety behavior: a cross‐level intervention model*.	
Country	IL
Aim	Present three intervention studies designed to modify supervisory monitoring and rewarding of subordinates' safety behaviors.
Target population	**Occupation**: Manufacturing, several: oil refinery station, canning, and distribution section, processing baked goods and milk goods
	**Industry**: C—Manufacturing
	**Setting**: 010 Industrial site
	**Firm size**: 250+(large)
Study design	**ITS** with comparison conditions: 1. Simple serial measures (No comparison)
	**Unit of analysis**: Group/organizational level
	**Sample size**: Company A 121 line workers and 13 shop‐floor supervisors, Company B 248 line workers and 23 shop‐floor supervisors; Company C 187 line workers and 13 shop‐floor supervisors
Type of intervention	2.1.7 Leadership‐based safety interventions
Evaluation design	**Duration of intervention:** 3 months
	**Duration of follow‐up**: 2 = Short‐term (≤12 months)
	**Type of outcome measure**: Risk or behavior
Study quality	Moderate quality

#### 9.1.4 Included BA studies

95 before and after studies (BA studies) were identified. However, as the types of interventions covered by this study design did not add new types of safety interventions, they were not included in the analysis of the results. In chapter 11 there is an overview of the BA studies. An overview of the before and after studies is provided in Table 16.

### 9.2 Characteristics of excluded studies

**Table:** 

Reference	Reason for exclusion
Azar‐Cavanagh M, 2007	Outcome data is lacking.
Childs JD, 2010	Comparisons of effects of different training methods, not accidents at work.
Daltroy LH, 1993	Only design article. Results reported elsewhere.
Donaldson CS, 1993	About chronic low back pain, not accidental injuries.
Hall N, 2013	Only have abstract which is not adequate to extract useful data.
Helitzer DL, 2014	Outcome is not relevant, as it is only knowledge about whether safety equipment is available, not the use of equipment.
Lavender S, 2007	About repetitive strain injuries that causes chronic diseases, and thus not acute accidental injuries.
Miller A, 2006	Only using post measures and only using cost, and no denominator for MSI.
Mullen JE, 2009	Design is unclear and more than 80% loss to follow‐up.
Nielsen KJ, 2008	Not considered an intervention study.
Reddell CR 1992	Lack of relevant outcome data for the three intervention groups and control.
Shaw W, 2006	It is about return to work, not safety interventions.
van der Molen HF, 2012	Overlap in data with other included study.
Warburton AL, 2000	The safety intervention was not as described, and implemented wrongly. (used non‐toughened glassware instead of toughened)

### 9.3 Characteristics of studies awaiting classification

**Table:** 

Reference	Reason for not being evaluated
Bena A, 2009	Article in Italian
Benavides FG, 2007	Article in Spanish
Fekieta R, 2007	Dissertation, was not able to get a copy
Hernández Navarrete MJ, 2010	We have not been able to get the article within time frame, and the article is in Spanish
Lanoie P, 1996	Article in French
Lim, 2011	Conference abstract, not enough data for analysis
López‐Rojas P, 2010	Article in Spanish
Markovic‐Denic LN, 2011	Poster presentation with insufficient data for analysis
Porru S, 2009	Article in Italian
Urban A, 2012	Article in Italian

## 10 ADDITIONAL TABLES

**Overview of additional tables:**

Tables 10, 11, 12, 13, 14, 15 Nature of included safety interventions using RCT, CBA or serial measures (ITS), by main type of safety intervention and by key components. Table 16 Nature of identified before and after studies (not included in analyses).

**Table 10:** NATURE OF INCLUDED Attitude modification INTERVENTIONS by TYPE OF SAFETY INTERVENTION

1.1.0 Attitude modification					
	Title	Participants	Intervention characteristics		Outcome
**Forst, L** (2004) (US) **CBA**	Effectiveness of community health workers for promoting use of safety eyewear by Latino farm workers Study arm: Introduction of safety information	**Industrial activity (NACE):** A—Agriculture, forestry and fishing Occupational activity: Crop and animal production, hunting and related service activities Work setting: 030 Farming and forestry	**KEY COMPONENT(S): 1.1.1 Safety campaign, by use of various means** Rationale of intervention: Campaign—if people get the right information they change attitudes and behavior.		**Outcome measure: Risk or behavior** Type of accidents: 10 = Other specific types of accident Type of injuries: 018 = Eye Injuries
			Theoretical or conceptual framework:	Low risk	
			Interaction between context and intervention:	Unclear	
			Intervention fidelity:	High risk	
**Gregersen, NP** (1996) (SE) **CBA**	Road safety improvement in large companies. An experimental comparison of different measures Study arm: Safety campaign	**Industrial activity (NACE):** H—Transporting and storage Occupational activity: Land transport and transport via pipelines Work setting: 060 Public area (public places or transport)	**KEY COMPONENT(S): 1.1.1 Safety campaign, by use of various means** Rationale of intervention: Campaigns have rarely proved effective. By focusing on specific and well known problems (such as low friction roads and darkness or loading goods), and by concentrating on small, local groups of drivers, the probability of success may be increased.		**Outcome measure: Injury** Type of accidents: 5 = Collision and other horizontal impact on body Type of injuries: 888 = All types of injuries
			Theoretical or conceptual framework:	Low risk	
			Interaction between context and intervention:	High risk	
			Intervention fidelity:	Low risk	
**Adams, JSK** (2013) (IN) **RCT**	Increasing compliance with protective eyewear to reduce ocular injuries in stone‐quarry workers in Tamil Nadu, India:	**Industrial activity (NACE):** Mining Occupational activity: Stone quarry workers Work setting: 100 Underground	**KEY COMPONENT(S): 1.1.2 Counseling approaches** Rationale of intervention: This intervention should work because it is an enhanced educational package to increase the regular use of protective eyewear and thereby reducing the incidence of eye injuries amongst quarry workers.		**Outcome measure: Injury** Type of accidents: 10 = Other specific types of accident Type of injuries: 018 = Eye Injuries
			Theoretical or conceptual framework:	Low risk	
			Interaction between context and intervention:	Unclear	
			Intervention fidelity:	Low risk	
**Gadomski, A** (2006) (US) **RCT**	Efficacy of the North American guidelines for children's agricultural tasks in reducing childhood agricultural injuries	**Industrial activity (NACE):** A—Agriculture, forestry and fishing Occupational activity: Crop and animal production, hunting and related service activities Work setting: 030 Farming and forestry	**KEY COMPONENT(S): 1.1.2 Counseling approaches** Rationale of intervention: In increasing participants awareness of child safety on farms.		**Outcome measure: Injury** Type of accidents: 1 = All types of accident Type of injuries: 888 = All types of injuries
			Theoretical or conceptual framework:	Unclear	
			Interaction between context and intervention:	High risk	
			Intervention fidelity:	High risk	
**Gregersen, NP** (1996) (SE) **CBA**	Road safety improvement in large companies. An experimental comparison of different measures Study arm: Counseling	**Industrial activity (NACE):** H—Transporting and storage Occupational activity: Land transport and transport via pipelines Work setting: 060 Public area (public places or transport)	**KEY COMPONENT(S): 1.1.2 Counseling approaches** Rationale of intervention: Group discussions have proved effective in transport before.		**Outcome measure: Injury** Type of accidents: 5 = Collision and other horizontal impact on body Type of injuries: 888 = All types of injuries
			Theoretical or conceptual framework:	Low risk	
			Interaction between context and intervention:	High risk	
			Intervention fidelity:	Low risk	
**Van der Molen, HF** (2011) (NL) **RCT**	Better effect of the use of a needle safety device in combination with an interactive workshop to prevent needle stick injuries Study arm: 1 h work shop	**Industrial activity (NACE):** Q—Human health and social work activities Occupational activity: Nurses/static Work setting: 050 Health establishment	**KEY COMPONENT(S): 1.1.2 Counseling approaches** Rationale of intervention: Not clearly specified by authors.		**Outcome measure: Injury** Type of accidents: 7 = contact with sharp or pointed materials or tools Type of injuries: 015 = Needlestick Injuries
			Theoretical or conceptual framework:	High risk	
			Interaction between context and intervention:	Unclear	
			Intervention fidelity:	High risk	
**Bena, A** (2009) (IT) **ITS**	Effectiveness of the training program for workers at construction sites of the high‐speed railway line between Torino and Novara: impact on injury rates.	**Industrial activity (NACE):** F—Construction Occupational activity: Civil engineering: excavation, drivers, operators, carpenters, iron workers, crane operators etc./dynamic Work setting:	**KEY COMPONENT(S): 1.1.3 Teaching, education to increase knowledge and awareness** Rationale of intervention: Training was designed to address: risk awareness; understanding of relationships among action, danger, and risks; improve understanding of risk management; improve understanding of safety requirements.		**Outcome measure: Injury** Type of accidents: 1 = All types of accident Type of injuries: 888 = All types of injuries
			Theoretical or conceptual framework:	Low risk	
			Interaction between context and intervention:	Low risk	
			Intervention fidelity:	Low risk	
**Johnson, OE** (2012) (NG) **CBA**	Effect of health education on the riding habits of commercial motorcyclists in Uyo, southern Nigeria	**Industrial activity (NACE):** H—Transporting and storage Occupational activity: Motorcycle courriers Work setting: 060 Public area	**KEY COMPONENT(S): 1.1.3 Teaching, education to increase knowledge and awareness** Rationale of intervention: Poor riding habits are the cause of many road traffic accidents. Improved education will reduce accidents. The intervention group received education in correct riding habits and knowledge of causes of motor‐cycle accidents.		**Outcome measure: Injury** Type of accidents: 1 = All types of accident Type of injuries: 888 = All types of injuries
			Theoretical or conceptual framework:	High risk	
			Interaction between context and intervention:	Unclear	
			Intervention fidelity:	Low risk	
**Mehrdad, R** (2013) (IR) **CBA**	Effects of training course on occupational exposure to bloodborne pathogens: a controlled interventional study	**Industrial activity (NACE):** Q—Human health and social work activities Occupational activity: Human health activities Work setting: 050 Health establishment	**KEY COMPONENT(S): 1.1.3 Teaching, education to increase knowledge and awareness** Rationale of intervention: Previous studies have shown that personal protection can play an important role in prevention of bloodborne pathogens (BBPs), and studies have shown that education can effectively reduce the incidence of needle stick injuries among Health Care Workers (HCWs).		**Outcome measure: Injury** Type of accidents: 7 = contact with sharp or pointed materials or tools Type of injuries: 015 = Needlestick Injuries
			Theoretical or conceptual framework:	High risk	
			Interaction between context and intervention:	Unclear	
			Intervention fidelity:	Unclear	
**Mujuru, P.** (2009) (US) **ITS**	Evaluating the impact of an intervention to reduce Injuries among loggers in West Virginia, 1999–2007	**Industrial activity (NACE):** A—Agriculture, forestry and fishing Occupational activity: loggers/dynamic Work setting: 030 Farming and forestry	**KEY COMPONENT(S): 1.1.3 Teaching, education to increase knowledge and awareness** Rationale of intervention: Video‐based safety training intervention with particular focus on struck by and caught in, under or between accidents (fatality data).		**Outcome measure: Injury** Type of accidents: 1 = All types of accident Type of injuries: 888 = All types of injuries
			Theoretical or conceptual framework:	Unclear	
			Interaction between context and intervention:	Unclear	
			Intervention fidelity:	High risk	
**Wang, H** (2003) (CN) **CBA**	A training programme for prevention of occupational exposure to bloodborne pathogens: impact on knowledge, behavior and incidence of needle stick injuries among student nurses in Changsha, People's Republic of China	**Industrial activity (NACE):** Q—Human health and social work activities Occupational activity: Human health activities Work setting: 050 Health establishment	**KEY COMPONENT(S): 1.1.3 Teaching, education to increase knowledge and awareness** Rationale of intervention: Students have limited knowledge of Blood Borne Pathogens, and therefore a BBP prevention program was designed, including teaching and videos. Increased knowledge should prevent injury. This included knowledge of handwashing, protective barriers and care in the use of and disposal of needles.		**Outcome measure: Risk or behavior** Type of accidents: 7 = contact with sharp or pointed materials or tools Type of injuries: 015 = Needlestick Injuries
			Theoretical or conceptual framework:	Low risk	
			Interaction between context and intervention:	Low risk	
			Intervention fidelity:	High risk	

**Table 11:** Nature of included behavior modification interventions by type of safety intervention

1.2.0 Behavior modification					
	Title	Participants	Intervention characteristics		Outcome
**Banco, L** (1997) (US) **CBA**	The Safe Teen Work Project: a study to reduce cutting injuries among young and inexperienced workers Study arm: Safety training	**Industrial activity (NACE):** G—Wholesale and retail trade Occupational activity: Retail trade, except of motor vehicles and motorcycles Work setting: 040 tertiary activity area (such as office, teaching establishment, restaurant etc.), incl retail	**KEY COMPONENT(S): 1.2.2 Safety training** Rationale of intervention: Occupational safety and health professionals have developed a “hierarchy of controls” model to analyze and intervene in work‐related injuries and illnesses. The “passive” safety benefits of the safety cutter is more likely to be continued by management given its resultant cost savings.		**Outcome measure: Injury** Type of accidents: 7 = contact with sharp or pointed materials or tools Type of injuries: 010 = Wounds and superficial injuries
			Theoretical or conceptual framework:	High risk	
			Interaction between context and intervention:	High risk	
			Intervention fidelity:	Low risk	
**Cheng, AS** (2009) (HK) **RCT**	The effect of individual job coaching and use of health threat in a job‐specific occupational health education program on prevention of work‐related musculo‐skeletal back injury	**Industrial activity (NACE):** F—Construction Occupational activity: Construction work (71: Extraction and building trades workers) Work setting: 020 Construction site	**KEY COMPONENT(S): 1.2.4 Individual feedback or coaching** Rationale of intervention: Built on evidence that feedback and health messages should make the participant understand their potential risk and vulnerability, and that psychological coping strategies, such as response efficacy and self‐efficacy at work should, and all in all change their behavior.		**Outcome measure: Injury** Type of accidents: 8 = overexertion of the musculoskeletal system Type of injuries: 030 = overexertion injuries (dislocations, sprains and strains)
			Theoretical or conceptual framework:	Low risk	
			Interaction between context and intervention:	High risk	
			Intervention fidelity:	High risk	
**Daltroy, LH** (1997) (US) **RCT**	A controlled trial of an educational program to prevent low back injuries	**Industrial activity (NACE):** H—Transporting and storage Occupational activity: Postal and courier activities Work setting: 060 Public area	**KEY COMPONENT(S): 1.2.4 Individual feedback or coaching** Rationale of intervention: Built on previous research that back schools including information about back anatomy, mechanisms of pain, safe lifting technique etc., prevent injuries.		**Outcome measure: Injury** Type of accidents: 8 = overexertion of the musculoskeletal system Type of injuries: 030 = overexertion injuries (dislocations, sprains and strains)
			Theoretical or conceptual framework:	Low risk	
			Interaction between context and intervention:	Unclear	
			Intervention fidelity:	Low risk	
**Jinnah, HA** (2014) (US) **RCT**	Involving fathers in teaching youth about farm tractor seatbelt safety—a randomized control study Study arm: Staff lead intervention	**Industrial activity (NACE):** A—Agriculture, forestry and fishing Occupational activity: Farm work Work setting: 030 Farming and forestry	**KEY COMPONENT(S): 1.2.4 Individual feedback or coaching** Rationale of intervention: Intervention treats farm safety as a family issue, capitalizes on role of parent, and appeals to fathers' strong motivation to practice tractor safety for the sake of their youth.		**Outcome measure: Risk or behavior** Type of accidents: 5 = Collision and other horizontal impact on body Type of injuries: 888 = All types of injuries
			Theoretical or conceptual framework:	Low risk	
			Interaction between context and intervention:	Low risk	
			Intervention fidelity:	Low risk	
**Jinnah, HA** (2014) (US) **RCT**	Involving fathers in teaching youth about farm tractor seatbelt safety—a randomized control study. Study arm: Parent lead intervention	**Industrial activity (NACE):** A—Agriculture, forestry and fishing Occupational activity: Farm work Work setting: 030 Farming and forestry	**KEY COMPONENT(S): 1.2.4 Individual feedback or coaching** Rationale of intervention: Intervention treats farm safety as family issue, capitalizes on the role of parent, and appeals to fathers' strong motivation to practice tractor safety for the sake of their youth.		**Outcome measure: Risk or behavior** Type of accidents: 5 = Collision and other horizontal impact on body Type of injuries: 888 = All types of injuries
			Theoretical or conceptual framework:	Low risk	
			Interaction between context and intervention:	Low risk	
			Intervention fidelity:	Low risk	
**Quintana, R** (1999) (US) **CBA**	A task‐delineated safety approach for slip, trip and fall hazards	**Industrial activity (NACE):** H—Transporting and storage Occupational activity: Warehousing and support activities for transportation Work setting: 040 tertiary activity area (such as office, teaching establishment, restaurant etc.), incl retail	**KEY COMPONENT(S): 1.2.4 Individual feedback or coaching** Rationale of intervention: Task‐delineated safety (TDS), a behavior‐based safety management scheme, was developed to address the significant problems of slip, trip and fall accidents. The hypothesis of this approach is that hazards can be minimized if personnel are held directly accountable with clear task delineation for keeping an area safe. Role ambiguity would be minimized and a less hazardous environment would result. The key, therefore, to reducing the largest proportion of undesirable consequences is to change behaviors so they are safe, rather than policing specific unsafe acts by employees.		**Outcome measure: Risk or behavior** Type of accidents: 4 = Tripping, stumbling and falling Type of injuries: 999 = other specified injuries not incl in other headings
			Theoretical or conceptual framework:	Low risk	
			Interaction between context and intervention:	Unclear	
			Intervention fidelity:	Low risk	

**Table 12:** Nature of included physical strength and resistance modification interventions, by type of safety intervention

1.3.0 Modification of physical strength and resistance					
	Title	Participants	Intervention characteristics		Outcome
**Hilyer, JC** (1990) (US) **CBA**	A flexibility intervention to reduce the incidence and severity of joint injuries among municipal firefighters	**Industrial activity (NACE):** O—Public administration and defence Occupational activity: Public administration and defence; compulsory social security Work setting: 130 Emergency, rescuing and military sites	**KEY COMPONENT(S): 1.3.1 Individual physical training** Rationale of intervention: Better fitness should protect against injuries in firefighters work. Built on previous studies on exercises and injuries.		**Outcome measure: Injury** Type of accidents: 8 = overexertion of the musculoskeletal system Type of injuries: 030 = overexertion injuries (dislocations, sprains and strains)
			Theoretical or conceptual framework:	Low risk	
			Interaction between context and intervention:	High risk	
			Intervention fidelity:	Unclear	
**Knapik, JJ** (2003) (US) **CBA**	Injury and fitness outcomes during implementation of physical readiness training Study arm: Males	**Industrial activity (NACE):** O—Public administration and defence Occupational activity: Public administration and defence; compulsory social security Work setting: 130 Emergency, rescuing and military sites	**KEY COMPONENT(S): 1.3.1 Individual physical training** Rationale of intervention: Physical Readiness Training (PRT) differs from current training practices in that it de‐emphasizes running, and incorporates procedures and principles designed to reduce injuries and increase functional fitness.		**Outcome measure: Injury** Type of accidents: 1 = All types of accident Type of injuries: 888 = All types of injuries
			Theoretical or conceptual framework:	Low risk	
			Interaction between context and intervention:	Low risk	
			Intervention fidelity:	Low risk	
**Knapik, JJ** (2003) (US) **CBA**	Injury and fitness outcomes during implementation of physical readiness training Study arm: Females	**Industrial activity (NACE):** O—Public administration and defence Occupational activity: Public administration and defence; compulsory social security Work setting: 130 Emergency, rescuing and military sites	**KEY COMPONENT(S): 1.3.1 Individual physical training** Rationale of intervention: Physical Readiness Training (PRT) differs from current training practices in that it de‐emphasizes running, and incorporates procedures and principles designed to reduce injuries and increase functional fitness.		**Outcome measure: Injury** Type of accidents: 1 = All types of accident Type of injuries: 888 = All types of injuries
			Theoretical or conceptual framework:	Low risk	
			Interaction between context and intervention:	Low risk	
			Intervention fidelity:	Low risk	
**Kuehl, KS** (2013) (US) **ITS**	Economic benefit of the PHLAME wellness programme on firefighter injury	**Industrial activity (NACE):** O—Public administration and defence Occupational activity: Firefighters/dynamic Work setting: 130 Emergency, rescuing and military sites	**KEY COMPONENT(S): 1.3.1 Individual physical training** Rationale of intervention: Fitness improvement is the basis for reduced injuries.		**Outcome measure: Injury** Type of accidents: 1 = All types of accident Type of injuries: 888 = All types of injuries
			Theoretical or conceptual framework:	Unclear	
			Interaction between context and intervention:	High risk	
			Intervention fidelity:	Unclear	
**Morgan, PJ** (2012) (AU) **RCT**	The impact of a workplace‐based weight loss program on work‐related outcomes in overweight male shift workers	**Industrial activity (NACE):** C—Manufacturing Occupational activity: Shift workers at metal factory Work setting: 010 Industrial site	**KEY COMPONENT(S): 1.3.1 Individual physical training** Rationale of intervention: Improvement in ability to perform physical demanding tasks can be related to weight loss.		**Outcome measure: Other** Type of accidents: 1 = All types of accident Type of injuries: 888 = All types of injuries
			Theoretical or conceptual framework:	Low risk	
			Interaction between context and intervention:	Low risk	
			Intervention fidelity:	High risk	

**Table 13:** Nature of included safety interventions using climate modification, by type of safety intervention

2.1.0 Climate modifications					
	Title	Participants	Intervention characteristics		Outcome
**Cooper, M** (1994) (UK) **ITS**	Reducing accidents using goal setting and feedback: A field study	**Industrial activity (NACE):** C—Manufacturing Occupational activity: Manufacture of rubber and plastic products; factory workers (cellophane film)/static Work setting: 11 Industrial site	**KEY COMPONENT(S): 2.1.1 Goal setting and feedback at group or organizational level** Rationale of intervention: Group goal setting creates a normative pressure and may thus provide an influencing component on safe behavior.		**Outcome measure: Injury** Type of accidents: 1 = All types of accident Type of injuries: 888 = All types of injuries
			Theoretical or conceptual framework:	Low risk	
			Interaction between context and intervention:	High risk	
			Intervention fidelity:	High risk	
**Cunningham, TR** (2007) (US) **ITS**	Using goal setting, task clarification, and feedback to increase the use of the hands‐free technique by hospital operating room staff	**Industrial activity (NACE)**: Q—Human health and social work activities Occupational activity: Operation room staff Work setting: 050 Health establishment	**KEY COMPONENT(S): 2.1.1 Goal setting and feedback at group or organizational level** Rationale of intervention: To avoid contamination between nurse and physician a “hands‐free technique” was used combined with goal setting and feedback mechanism.		**Outcome measure: Risk or behavior** Type of accidents: 7 = contact with sharp or pointed materials or tools Type of injuries: 999 = other specified injuries not incl in other headings
			Theoretical or conceptual framework:	Low risk	
			Interaction between context and intervention:	High risk	
			Intervention fidelity:	Low risk	
**Fellner, DJ** (1984) (US) **ITS**	Increasing industrial safety practices and conditions through posted feedback	**Industrial activity (NACE)**: C—Manufacturing Occupational activity: paper products Work setting: 010 Industrial site	**KEY COMPONENT(S): 2.1.1 Goal setting and feedback at group or organizational level** Rationale of intervention: The intervention works because workers are trained to discriminate between safe and unsafe practices, setting goals for safe practices and are provided with weekly posted and specific feedback.		**Outcome measure: Injury** Type of accidents: 1 = All types of accident Type of injuries: 888 = All types of injuries
			Theoretical or conceptual framework:	Low risk	
			Interaction between context and intervention:	High risk	
			Intervention fidelity:	High risk	
**Mattila, M** (1988) (FI) **CBA**	Promoting job safety in building: an experiment on the behavior analysis approach	**Industrial activity (NACE)**: F—Construction Occupational activity: Construction of buildings Work setting: 020 construction site	**KEY COMPONENT(S): 2.1.1 Goal setting and feedback at group or organizational level** Rationale of intervention: The intervention will work because reinforcing the positive consequences of the wanted (safety) behavior while reducing the positive consequences and/or strengthening the negative consequences of the unwanted (safety) behavior, will improve safety at construction sites.		**Outcome measure: Injury** Type of accidents: 1 = All types of accident Type of injuries: 888 = All types of injuries
			Theoretical or conceptual framework:	Low risk	
			Interaction between context and intervention:	Unclear	
			Intervention fidelity:	Low risk	
**Ray, PS** (1997) (US) **CBA**	Efficacy of the components of a behavioral safety program	**Industrial activity (NACE)**: C—Manufacturing Occupational activity: Manufacture of fabricated metal products, except machinery and equipment Work setting: 010 Industrial site	**KEY COMPONENT(S): 2.1.1 Goal setting and feedback at group or organizational level** Rationale of intervention: Due to the presence of several competing motivations not to follow safety rules, (e.g., production pressures, macho attitudes) it is essential to motivate workers to increase safety behavior in the workplace.		**Outcome measure: Risk or behavior** Type of accidents: 1 = All types of accident Type of injuries: 888 = All types of injuries
			Theoretical or conceptual framework:	Low risk	
			Interaction between context and intervention:	Low risk	
			Intervention fidelity:	Low risk	
**Sulzer‐Azaroff, B** (1990) (US) **ITS**	Improving occupational safety in a large industrial plant	**Industrial activity (NACE)**: C—Manufacturing Occupational activity: Electrical manufacturing workers Work setting: 11 Industrial site	**KEY COMPONENT(S): 2.1.1 Goal setting and feedback at group or organizational level** Rationale of intervention: Behavior that is rewarded by, e.g., positive feedback and extrinsic rewards are being reinforced, performance goals may be motivating. Extrinsic rewards offered to groups present strong group pressure. Clearly communicated and assigned priority from superior management exerts pressure on and motivates subordinate management levels to follow safe practices.		**Outcome measure: Risk or behavior** Type of accidents: 1 = All types of accident Type of injuries: 888 = All types of injuries
			Theoretical or conceptual framework:	Low risk	
			Interaction between context and intervention:	High risk	
			Intervention fidelity:	High risk	
**Sulzer‐Azaroff, B.** (1980) (UK) **ITS**	Manufacturing safety hazard reduction through performance feedback	**Industrial activity (NACE):** C—Manufacturing Occupational activity: Manufacturing‐ not classified further Work setting: 010 Industrial site	**KEY COMPONENT(S): 2.1.1 Goal setting and feedback at group or organizational level** Rationale of intervention: Feedback in manufacturing has previously shown short term results, based on behavioral literature.		**Outcome measure: Risk or behavior** Type of accidents: 0 = Not specified Type of injuries: 000 = Type of injury unknown
			Theoretical or conceptual framework:	Low risk	
			Interaction between context and intervention:	High risk	
			Intervention fidelity:	High risk	
**Kines, P** (2010) (DK) **CBA**	Improving construction site safety through leader‐based verbal safety communication	**Industrial activity (NACE):** F—Construction Occupational activity: Construction of buildings Work setting: 020 Construction site	**KEY COMPONENT(S): 2.1.7 Leadership‐based safety interventions** Rationale of intervention: Supervisory feedback and recognition were amongst the most powerful incentives influencing job performance. Daily supervisory feedback regarding safe and unsafe behavior and conditions provides an indication of the true priorities between production and safety. Priority of safety should increase because of the leader based safety intervention.		**Outcome measure: Risk or behavior** Type of accidents: 1 = All types of accident Type of injuries: 888 = All types of injuries
			Theoretical or conceptual framework:	Low risk	
			Interaction between context and intervention:	High risk	
			Intervention fidelity:	Low risk	
**Kines, P** (2013) (DK) **RCT**	Improving safety in small enterprises through an integrated safety management intervention	**Industrial activity (NACE):** C—Manufacturing Occupational activity: Metal processing/Dynamic and static Work setting: 010 Industrial site	**KEY COMPONENT(S): 2.1.7 Leadership‐based safety interventions** Rationale of intervention: Dejoy's (2005) integrated safety management theory is composed of both a problem solving process and a culture change process that work in parallel. This will improve both management support and worker involvement, and in turn improved safety.		**Outcome measure: Risk or behavior** Type of accidents: 1 = All types of accident Type of injuries: 888 = All types of injuries
			Theoretical or conceptual framework:	Low risk	
			Interaction between context and intervention:	High risk	
			Intervention fidelity:	Low risk	
**Zohar, D** (2002) (IL) **RCT**	Modifying supervisory practices to improve subunit safety: a leadership‐based intervention model	**Industrial activity (NACE)**: C—Manufacturing Occupational activity: Repair and installation of machinery and equipment Work setting: 010 Industrial site	**KEY COMPONENT(S): 2.1.7 Leadership‐based safety interventions** Rationale of intervention: A leader‐based safety intervention allows modification of all subordinate safety behaviors (including transient and uncommon ones), because antecedents and consequences are based on continual supervisory monitoring in ever‐changing situations:		**Outcome measure: Injury** Type of accidents: 1 = All types of accident Type of injuries: 888 = All types of injuries
			Theoretical or conceptual framework:	Low risk	
			Interaction between context and intervention:	High risk	
			Intervention fidelity:	Low risk	
**Zohar, D.** (2003) (IL) **ITS**	The use of supervisory practices as leverage to improve safety behavior: a cross‐level intervention model	**Industrial activity (NACE)**: C—Manufacturing Occupational activity: Manufacturing, several: oil refinery station, canning and distribution section, processing baked goods and milk goods Work setting: 010 Industrial site	**KEY COMPONENT(S): 2.1.7 Leadership‐based safety interventions** Rationale of intervention: The expected utility of unsafe behavior exceeds that of safe behavior. This intervention works because supervisory practices and higher‐level managers communicate high safety priorities, even under increased work pressure.		**Outcome measure: Risk or behavior** Type of accidents: 1 = All types of accident Type of injuries: 888 = All types of injuries
			Theoretical or conceptual framework:	Low risk	
			Interaction between context and intervention:	Low risk	
			Intervention fidelity:	Low risk	

**Table 14:** Nature of included structural safety interventions, by main type of safety intervention

2.2.0 Structural modifications					
	Title	Participants	Intervention characteristics		Outcome
**Bulzacchelli, MT** (2007) (US) **ITS**	Effects of the Occupational Safety and Health Administration's control of hazardous energy (lockout/tagout) standard on rates of machinery‐related fatal occupational injury.	**Industrial activity (NACE):** All or mixed industries Occupational activity: Mixed: industrial and construction/mixed Work setting: 888 All or mixed setting	**2.2.1 Legislative changes** Rationale of intervention: The (lockout/tagout) standard addresses the source of the fatality—hazardous transfer of energy (electrical, mechanical, hydraulic, pneumatic, chemical, thermal, or other), aiming to remove it while workers service or perform maintenance on machines/equipment. The typical barrier in place between the worker and the energy during normal operation is not feasible for the tasks of servicing and maintenance. The standard's polices/procedures include multiple components: a program, training, and inspections.		**Outcome measure: Injury** Type of accidents: 2 = contact with electrical voltage Type of injuries: 700 = Fatal Injuries
			Theoretical or conceptual framework:	Low risk	
			Interaction between context and intervention:	Low risk	
			Intervention fidelity:	High risk	
**Casteel, C** (2009) (US) **ITS**	Hospital employee assault rates before and after enactment of the California Hospital Safety and Security Act. Study arm: Emergency units	**Industrial activity (NACE):** Q—Human health and social work activities Occupational activity: Social and healthcare Work setting: 050 Health establishment	**2.2.1 Legislative changes** Rationale of intervention: Comprehensive plans required of hospitals to contain and deal with violence; to include layout, staffing, security personnel, policies, education, and training. Multifaceted safety intervention.		**Outcome measure: Other** Type of accidents: 9 = Assault or violence at work. Type of injuries: 888 = All types of injuries
			Theoretical or conceptual framework:	Low risk	
			Interaction between context and intervention:	Low risk	
			Intervention fidelity:	Unclear	
**Casteel, C** (2009) (US) **ITS**	Hospital employee assault rates before and after enactment of the California Hospital Safety and Security Act Study arm: psychiatric units	**Industrial activity (NACE):** Q—Human health and social work activities Occupational activity: Social and healthcare Work setting: 050 Health establishment	**2.2.1 Legislative changes** Rationale of intervention: Comprehensive plans required of hospitals to contain and deal with violence; to include layout, staffing, security personnel, policies, education, and training. Multifaceted safety intervention.		**Outcome measure: Other** Type of accidents: 9 = Assault or violence at work. Type of injuries: 888 = All types of injuries
			Theoretical or conceptual framework:	High risk	
			Interaction between context and intervention:	Low risk	
			Intervention fidelity:	Unclear	
**Derr, JD** (2001) (US) **ITS**	Fatal falls in the US construction industry, 1990 to 1999	**Industrial activity (NACE):** F—Construction Occupational activity: mixed construction workers Work setting: 020 Construction site	**2.2.1 Legislative changes** Rationale of intervention: Not clearly described. The authors do not state in the outset of the article what this standard revision provided regarding making workers' jobs safer with respect to fall prevention.		**Outcome measure: Injury** Type of accidents: 4 = Tripping, stumbling and falling Type of injuries: 700 = Fatal Injuries
			Theoretical or conceptual framework:	High risk	
			Interaction between context and intervention:	High risk	
			Intervention fidelity:	High risk	
**Lanoie, P** (1992) (CA) **CBA**	Safety regulation and the risk of workplace accidents in Quebec	**Industrial activity (NACE):** All or mixed industries Occupational activity: All occupations Work setting: 888 All or mixed setting	**2.2.1 Legislative changes** Rationale of intervention: Not specified, but lies implicit in the theoretical approach taken, which is based on the concept of the principal‐agent framework, in which the determination of wage is considered and both firm and worker can influence the risk.		**Outcome measure:** Type of accidents: Type of injuries: 888 = All types of injuries
			Theoretical or conceptual framework:	High risk	
			Interaction between context and intervention:	Unclear	
			Intervention fidelity:	Unclear	
**Lipscomb, H** (2003) (US) **ITS**	Work‐related falls among union carpenters in Washington State before and after the Vertical Fall Arrest Standard.	**Industrial activity (NACE):** F—Construction Occupational activity: Construction; carpenters. Specialized construction activities/dynamic. Work setting: 020 construction site	**2.2.1 Legislative changes** Rationale of intervention: Policy mandates improved protection and aspects to decrease severity. The Washington State standard outlined (1) activities that could reduce the risk of falling, (2) the use of equipment and safety planning to reduce the impact of falls, and (3) provisions for rapid evacuation of the worker in the event of injury, any of which could influence the rates at which fall from high occur and/or the severity of those falls.		**Outcome measure: Injury** Type of accidents: 4 = Tripping, stumbling and falling Type of injuries: 888 = All types of injuries
			Theoretical or conceptual framework:	Low risk	
			Interaction between context and intervention:	Unclear	
			Intervention fidelity:	Unclear	
**Marlenga, B** (2006) (USA) **ITS**	Evaluation of a policy to reduce youth tractor crashes on public roads	**Industrial activity (NACE):** A—Agriculture, forestry and fishing Occupational activity: Agriculture—youth tractor driving on public roads Work setting: 030 Farming and forestry	**2.2.1 Legislative changes** Rationale of intervention: The tractor certification course was designed to meet federal exemption requirements under the Fair Labor Standards Act—Hazardous Occupations Order for Agriculture, which includes education. But authors state that it was not designed to prevent youth tractor crashes on public roads.		**Outcome measure: Injury** Type of accidents: 5 = Collision and other horizontal impact on body Type of injuries: 888 = All types of injuries
			Theoretical or conceptual framework:	Low risk	
			Interaction between context and intervention:	Low risk	
			Intervention fidelity:	Low risk	
**Monforton, C** (2010) (US) **ITS**	An impact evaluation of a federal mine safety training regulation on injury rates among US stone, sand, and gravel mine workers: an interrupted time‐series analysis.	**Industrial activity (NACE):** B—Mining and quarrying Occupational activity: Mine workers‐ stone, sand, gravel/dynamic Work setting: 010 Industrial site	**2.2.1 Legislative changes** Rationale of intervention: Education is often assumed to be effective in preventing injuries, but with often little evidence. Authors suggest that training should be combined with an evaluation of safety.		**Outcome measure: Injury** Type of accidents: 1 = All types of accident Type of injuries: 888 = All types of injuries
			Theoretical or conceptual framework:	Low risk	
			Interaction between context and intervention:	Unclear	
			Intervention fidelity:	Unclear	
**Suruda, A** (2002) (US) **ITS**	Impact of the OSHA trench and excavation standard on fatal injury in the construction industry	**Industrial activity (NACE):** F—Construction Occupational activity: Construction Industry wide Work setting: 020 Construction site	**2.2.1 Legislative changes** Rationale of intervention: Standard for trenching activates with targeted inspections and fines. The authors are not very specific about whether this standard change was expected to influence rates of injury. They indicated that the effectiveness of OSHA regulatory changes and enforcement is unclear, but that similar efforts by the Mine, Safety and Health Administration (MSHA) have been effective. They mentioned that this standard change included the use of a targeted inspection program and a revised standard (which improved upon a previously ambiguous standard).		**Outcome measure: Injury** Type of accidents: 6 = Trapped, crushed, struck by equipment or objects Type of injuries: 700 = Fatal Injuries
			Theoretical or conceptual framework:	Low risk	
			Interaction between context and intervention:	High risk	
			Intervention fidelity:	Unclear	
**Gregersen, NP** (1996) (SE) **CBA**	Road safety improvement in large companies. An experimental comparison of different measures Study arm: Economic incentives	**Industrial activity (NACE):** H—Transporting and storage Occupational activity: Land transport and transport via pipelines Work setting: 060 Public area (public places or transport)	**2.2.2 Economic incentives** Rationale of intervention: A way of combining individual gain with the important effect of social norms is to make groups of drivers earn the bonus together. The driver should then feel a responsibility not only toward himself and the company, but also toward his fellow drivers. The fewer the accidents in the group, the larger the reward to be gained.		**Outcome measure: Injury** Type of accidents: 5 = Collision and other horizontal impact on body Type of injuries: 888 = All types of injuries
			Theoretical or conceptual framework:	Low risk	
			Interaction between context and intervention:	High risk	
			Intervention fidelity:	Low risk	
**Rautiainen, R** (2005) (FI) **ITS**	Effects of premium discount on workers' compensation claims in agriculture in Finland	**Industrial activity (NACE):** A—Agriculture, forestry and fishing Occupational activity: Crop and animal production, hunting and related service activities/dynamic Work setting: 030 Farming and forestry	**2.2.2 Economic incentives** Rationale of intervention: Finnish farmers are self‐employed, and safety regulation is rarely enforced. Insurance premium reduction could provide economic incentives for safety. Incentives are based on their own experiences rather than group.		**Outcome measure: Injury** Type of accidents: 1 = All types of accident Type of injuries: 888 = All types of injuries
			Theoretical or conceptual framework:	Low risk	
			Interaction between context and intervention:	Low risk	
			Intervention fidelity:	Low risk	
**Foley, M** (2012) (US) **CBA**	The impact of regulatory enforcement and consultation visits on workers' compensation claims incidence rates and costs, 1999–2008 Study arm: Non‐fixed sites with consultation	**Industrial activity (NACE):** All or mixed industries Occupational activity: mixed, all Work setting: 888 All or mixed setting	**2.2.3 Soft regulation** Rationale of intervention: Interventions are required to set and enforce OHS standards and regulations to reduce injury and illness rates, although several preconditions must exist for such reductions to occur.		**Outcome measure: Injury** Type of accidents: 8 = overexertion of the musculoskeletal system Type of injuries: 030 = overexertion injuries (dislocations, sprains and strains)
			Theoretical or conceptual framework:	Low risk	
			Interaction between context and intervention:	Unclear	
			Intervention fidelity:	Low risk	
**Foley, M** (2012) (US) **CBA**	The impact of regulatory enforcement and consultation visits on workers' compensation claims incidence rates and costs, 1999–2008 Study arm: Fixed sites with consultation	**Industrial activity (NACE):** All or mixed industries Occupational activity: mixed, all Work setting: 888 All or mixed setting	**2.2.3 Soft regulation** Rationale of intervention: Interventions are required to set and enforce OHS standards and regulations to reduce injury and illness rates, although several preconditions must exist for such reductions to occur.		**Outcome measure: Injury** Type of accidents: 8 = overexertion of the musculoskeletal system Type of injuries: 030 = overexertion injuries (dislocations, sprains and strains)
			Theoretical or conceptual framework:	Low risk	
			Interaction between context and intervention:	Unclear	
			Intervention fidelity:	Low risk	
**Hogg‐Johnson, S** (2012) (CA) **RCT**	A randomized controlled study to evaluate the effectiveness of targeted occupational health and safety consultation or inspection in Ontario manufacturing workplaces Study arm: Voluntary consultation services	**Industrial activity (NACE):** C—Manufacturing Occupational activity: Mixed Work setting: 010 Industrial site	**2.2.3 Soft regulation** Rationale of intervention: The implicit assumption is that consultation by Health & Safety Association (HSA) or Ontario Workplace Safety and Insurance Board (WSIB) on OHS matters should decrease occupational injuries.		**Outcome measure: Injury** Type of accidents: 1 = All types of accident Type of injuries: 888 = All types of injuries
			Theoretical or conceptual framework:	Low risk	
			Interaction between context and intervention:	Low risk	
			Intervention fidelity:	High risk	
**Alamgir, H** (2008) (CA) **ITS**	Efficiency of overhead ceiling lifts in reducing musculoskeletal injury among carers working in long‐term care institutions.	**Industrial activity (NACE):** Q—Human health and social work activities Occupational activity: Residential long term care activities/dynamic Work setting: 050 Health establishment	**2.2.4 Engineering controls** Rationale of intervention: The mechanical part is explained and why it should be better than floor based lifting equipment "Ceiling lifts involve a ceiling mounted track, an electric motor and a sling to assist with lifting, transferring and repositioning patients"		**Outcome measure: Injury** Type of accidents: 8 = overexertion of the musculoskeletal system Type of injuries: 030 = overexertion injuries (dislocations, sprains and strains)
			Theoretical or conceptual framework:	Low risk	
			Interaction between context and intervention:	High risk	
			Intervention fidelity:	Low risk	
**Banco, L** (1997) (US) **CBA**	The Safe Teen Work Project: a study to reduce cutting injuries among young and inexperienced workers Study arm: Engineering control	**Industrial activity (NACE):** G—Wholesale and retail trade Occupational activity: Retail trade, except of motor vehicles and motorcycles Work setting: 040 tertiary activity area (such as office, teaching establishment, restaurant etc.), incl retail	**2.2.4 Engineering controls** Rationale of intervention: Occupational safety and health professionals have developed a “hierarchy of controls” model to analyze and intervene in work‐related injuries and illnesses. The “passive” safety benefits of the safety cutter is more likely to be continued by management given its resultant cost savings.		**Outcome measure: Injury** Type of accidents: 7 = contact with sharp or pointed materials or tools Type of injuries: 010 = Wounds and superficial injuries
			Theoretical or conceptual framework:	Low risk	
			Interaction between context and intervention:	Unclear	
			Intervention fidelity:	Low risk	
**Bell, JL** (2002) (US) **CBA**	Changes in logging injury rates associated with use of feller‐bunchers in West Virginia	**Industrial activity (NACE):** A—Agriculture, forestry and fishing Occupational activity: Agriculture, forestry and fishing Work setting: 030 Farming and forestry	**2.2.4 Engineering controls** Rationale of intervention: A feller‐buncher is likely to have the strongest impact on injuries because it is used to cut down trees. OSHA describes a feller‐buncher as a mobile machine with an operator enclosure and an articulating extensible arm onto which a felling head (either a disc saw or chain saw) is attached.		**Outcome measure: Injury** Type of accidents: 1 = All types of accident Type of injuries: 888 = All types of injuries
			Theoretical or conceptual framework:	Low risk	
			Interaction between context and intervention:	High risk	
			Intervention fidelity:	High risk	
**Briggs, SC** (2003) (US) **ITS**	The effect of supermaximum security prisons on aggregate levels of institutional violence	**Industrial activity (NACE):** O—Public administration and defence Occupational activity: prison guards/dynamic Work setting: 040 tertiary activity area (such as office, teaching establishment, restaurant etc.), incl retail	**2.2.4 Engineering controls** Rationale of intervention: Supermaximum prisons should be harder for prisoners to attack guards, although the possibility of isolation might also motivate prisoners to attack guards under certain conditions.		**Outcome measure: Injury** Type of accidents: 9 = Assault or violence at work. Type of injuries: 888 = All types of injuries
			Theoretical or conceptual framework:	Low risk	
			Interaction between context and intervention:	Low risk	
			Intervention fidelity:	Low risk	
**Grimmond, T** (2010) (NZ) **CBA**	Sharps injury reduction using a sharps container with enhanced engineering: a 28 hospital nonrandomized intervention and cohort study	**Industrial activity (NACE):** Q—Human health and social work activities Occupational activity: Human health activities Work setting: 050 Health establishment	**2.2.4 Engineering controls** Rationale of intervention: This study assume that containers with enhanced engineering can reduce Container‐Associated Sharps Injuries (CASI).		**Outcome measure: Injury** Type of accidents: 7 = contact with sharp or pointed materials or tools Type of injuries: 015 = Needlestick Injuries
			Theoretical or conceptual framework:	Low risk	
			Interaction between context and intervention:	High risk	
			Intervention fidelity:	Low risk	
**Harms‐Ringdahl, L** (1987) (SE) **CBA**	Safety analysis in design—evaluation of a case study	**Industrial activity (NACE):** C—Manufacturing Occupational activity: Manufacturing of paper and paper products Work setting: 010 Industrial site	**2.2.4 Engineering controls** Rationale of intervention: A system approach was used to analyses safety on the paper mill plant, including job safety analysis, energy analysis, and deviation analysis, as a basis for re‐design of production system. A safety analysis procedure are followed.		**Outcome measure: Injury** Type of accidents: 1 = All types of accident Type of injuries: 888 = All types of injuries
			Theoretical or conceptual framework:	Low risk	
			Interaction between context and intervention:	High risk	
			Intervention fidelity:	High risk	
**Jensen, SL** (1997) (DK) **RCT**	Double gloving as self‐protection in abdominal surgery	**Industrial activity (NACE):** Q—Human health and social work activities Occupational activity: Human health activities Work setting: 050 Health establishment	**2.2.4 Engineering controls** Rationale of intervention: Surgical gloves constitute a barrier between the surgeon and the patient, and prevent contamination. Previous research demonstrate that double gloving has been recommended to increase protection of surgeons.		**Outcome measure: Risk or behavior** Type of accidents: 7 = contact with sharp or pointed materials or tools Type of injuries: 015 = Needlestick Injuries
			Theoretical or conceptual framework:	Low risk	
			Interaction between context and intervention:	Low risk	
			Intervention fidelity:	Low risk	
**Lawrence, L** (1997) (US) **ITS**	The effectiveness of a needleless intravenous connection system: An assessment by injury rate and user satisfaction.	**Industrial activity (NACE):** Q—Human health and social work activities Occupational activity: hospital workers clinical—nurses Work setting: 050 Health establishment	**2.2.4 Engineering controls** Rationale of intervention: Behavioral interventions alone are not sufficient to eliminate percutaneous injuries caused by needles, therefore unnecessary needles were removed by introduction of safety equipment.		**Outcome measure: Injury** Type of accidents: 7 = contact with sharp or pointed materials or tools Type of injuries: 015 = Needlestick Injuries
			Theoretical or conceptual framework:	Low risk	
			Interaction between context and intervention:	Low risk	
			Intervention fidelity:	High risk	
**Prezant, D** (1999) (USA) **ITS**	Impact of a modern firefighting protective uniform on the incidence and severity of burn injuries in New York City firefighters.	**Industrial activity (NACE):** O—Public administration and defence Occupational activity: Public administration and defense; compulsory social security… fire fighters Work setting: 130 Emergency, rescuing and military sites	**2.2.4 Engineering controls** Rationale of intervention: Engineering top of PHHHC. The intervention should work because the entire New York City firefighter department (FDNY) replaced all traditional firefighter uniforms by modern uniforms that provide better protection from heat and fire (engineering control).		**Outcome measure: Injury** Type of accidents: 10 = Other specific types of accident Type of injuries: 060 = Burns, scalds and frostbites
			Theoretical or conceptual framework:	Low risk	
			Interaction between context and intervention:	Low risk	
			Intervention fidelity:	Low risk	
**Prunet, B** (2008) (FR) **RCT**	A prospective randomized trial of two safety peripheral intravenous catheters Study arm: Passive engineering control	**Industrial activity (NACE):** Q—Human health and social work activities Occupational activity: Human health activities Work setting: 050 Health establishment	**2.2.4 Engineering controls** Rationale of intervention: Authors refers to previous experience that education and organization of cases are insufficient in prevention of Needle Stick Injuries (NSI). They state that the passive safety catheter is automatically triggered, and therefore no contact with needle.		**Outcome measure: Injury** Type of accidents: 7 = contact with sharp or pointed materials or tools Type of injuries: 015 = Needlestick Injuries
			Theoretical or conceptual framework:	Low risk	
			Interaction between context and intervention:	Unclear	
			Intervention fidelity:	Low risk	
**Prunet, B** (2008) (FR) **RCT**	A prospective randomized trial of two safety peripheral intravenous catheters Study arm. Active engineering control	**Industrial activity (NACE):** Q—Human health and social work activities Occupational activity: Human health activities Work setting: 050 Health establishment	**2.2.4 Engineering controls** Rationale of intervention: Authors refers to previous experience that education and organization of cases are insufficient in prevention of Needle Stick Injuries (NSI). They state that the passive safety catheter is automatically triggered, and therefore no contact with needle.		**Outcome measure: Injury** Type of accidents: 7 = contact with sharp or pointed materials or tools Type of injuries: 015 = Needlestick Injuries
			Theoretical or conceptual framework:	Low risk	
			Interaction between context and intervention:	Unclear	
			Intervention fidelity:	Low risk	
**Reddy, S** (2001) (US) **ITS**	Assessing the effect of long‐term availability of engineering controls on needlestick injuries among health care workers: A 3‐year preimplementation and post‐implementation comparison	**Industrial activity (NACE):** Q—Human health and social work activities Occupational activity: healthcare workers; nurses and ancillary Work setting: 51 Health establishment	**2.2.4 Engineering controls** Rationale of intervention: Prevents exposure to sharps through engineering changes in devices.		**Outcome measure: Injury** Type of accidents: 7 = contact with sharp or pointed materials or tools Type of injuries: 015 = Needlestick Injuries
			Theoretical or conceptual framework:	Low risk	
			Interaction between context and intervention:	High risk	
			Intervention fidelity:	High risk	
**Rogues, A** (2004) (FR) **ITS**	Impact of safety devices for preventing percutaneous injuries related to phlebotomy procedures in health care workers.	**Industrial activity (NACE):** Q—Human health and social work activities Occupational activity: Hospital workers/dynamic Work setting: 050 Health establishment	**2.2.4 Engineering controls** Rationale of intervention: Engineering changes designed to remove exposure risk supported by education and surveillance.		**Outcome measure: Injury** Type of accidents: 7 = contact with sharp or pointed materials or tools Type of injuries: 015 = Needlestick Injuries
			Theoretical or conceptual framework:	Low risk	
			Interaction between context and intervention:	High risk	
			Intervention fidelity:	Low risk	
**Schoenfisch, AL** (2013) (US) **ITS**	Musculoskeletal injuries among hospital patient care staff before and after implementation of patient lift and transfer equipment. Study arm: Medical Center	**Industrial activity (NACE):** Q—Human health and social work activities Occupational activity: Healthcare, largely nurses—hospital employees/dynamic Work setting: 050 Health establishment	**2.2.4 Engineering controls** Rationale of intervention: Exposure reduction; Public Health Hierarchy of Hazard Control, but adoption of the equipment by staff actively is required for it to work.		**Outcome measure: Injury** Type of accidents: 8 = overexertion of the musculoskeletal system Type of injuries: 030 = overexertion injuries (dislocations, sprains and strains)
			Theoretical or conceptual framework:	Low risk	
			Interaction between context and intervention:	Low risk	
			Intervention fidelity:	High risk	
**Schoenfisch, AL** (2013) (US) **ITS**	Musculoskeletal injuries among hospital patient care staff before and after implementation of patient lift and transfer equipment. Study arm: Community Hospital	**Industrial activity (NACE):** Q—Human health and social work activities Occupational activity: Healthcare, largely nurses—hospital employees/dynamic Work setting: 050 Health establishment	**2.2.4 Engineering controls** Rationale of intervention: Exposure reduction; but adoption of the equipment by staff actively is required for it to work.		**Outcome measure: Injury** Type of accidents: 8 = overexertion of the musculoskeletal system Type of injuries: 030 = overexertion injuries (dislocations, sprains and strains)
			Theoretical or conceptual framework:	Low risk	
			Interaction between context and intervention:	Low risk	
			Intervention fidelity:	High risk	
**Smollen, P** (2004) (Australia) **ITS**	Evaluation of a programme designed to reduce occupational exposures from steel‐winged butterfly needles in the clinical setting.	**Industrial activity (NACE):** Q—Human health and social work activities Occupational activity: hospital/dynamic Work setting: 51 Health establishment	**2.2.4 Engineering controls** Rationale of intervention: Introduced engineering protection and exposure reduction, based on prior experience with engineering control in this context.		**Outcome measure: Injury** Type of accidents: 7 = contact with sharp or pointed materials or tools Type of injuries: 015 = Needlestick Injuries
			Theoretical or conceptual framework:	Low risk	
			Interaction between context and intervention:	High risk	
			Intervention fidelity:	Low risk	
**Sossai, D** (2010) (IT) **ITS**	Using an intravenous catheter system to prevent needlestick injury.	**Industrial activity (NACE):** Q—Human health and social work activities Occupational activity: health care workers‐ hospital/dynamic Work setting: 51 Health establishment	**2.2.4 Engineering controls** Rationale of intervention: Public Health Hierarchy of Hazards Controls and engineering solutions is emphasized. The authors stress the importance of engineering approaches, and in particular that passive is most useful in preventing exposure to a biological agency.		**Outcome measure: Injury** Type of accidents: 7 = contact with sharp or pointed materials or tools Type of injuries: 015 = Needlestick Injuries
			Theoretical or conceptual framework:	Low risk	
			Interaction between context and intervention:	High risk	
			Intervention fidelity:	Low risk	
**Van der Molen, HF** (2011) (NL) **RCT**	Better effect of the use of a needle safety device in combination with an interactive workshop to prevent needle stick injuries Study arm: Introduction of needles with safety devices	**Industrial activity (NACE):** Q—Human health and social work activities Occupational activity: Nurses/static Work setting: 050 Health establishment	**2.2.4 Engineering controls** Rationale of intervention: Not clearly specified by authors.		**Outcome measure: Injury** Type of accidents: 7 = contact with sharp or pointed materials or tools Type of injuries: 015 = Needlestick Injuries
			Theoretical or conceptual framework:	High risk	
			Interaction between context and intervention:	Unclear	
			Intervention fidelity:	Low risk	
**Whitby, M** (2008) (AU) **ITS**	Needlestick injuries in a major teaching hospital: the worthwhile effect of hospital‐wide replacement of conventional hollow‐bore needles.	**Industrial activity (NACE):** Q—Human health and social work activities Occupational activity: Human health activities/dynamic Work setting: 050 Health establishment	**2.2.4 Engineering controls** Rationale of intervention: Intervention work because engineering, not education, would provide a more effective solution and would significantly reduce high‐risk hollow‐bore Needle Stick Injuries.		**Outcome measure: Injury** Type of accidents: 7 = contact with sharp or pointed materials or tools Type of injuries: 015 = Needlestick Injuries
			Theoretical or conceptual framework:	Low risk	
			Interaction between context and intervention:	Low risk	
			Intervention fidelity:	Low risk	
**Birnbaum, D** (1993) (CA) **ITS**	Needlestick injuries among critical care nurses before and after adoption of universal precautions or body substance isolation	**Industrial activity (NACE):** Q—Human health and social work activities Occupational activity: Human health activities, critical care nurses Work setting: 050 Health establishment	**2.2.5 Administrative controls** Rationale of intervention: The intervention implies that risk drives the protective activity.		**Outcome measure: Injury** Type of accidents: 7 = contact with sharp or pointed materials or tools Type of injuries: 015 = Needlestick Injuries
			Theoretical or conceptual framework:	Low risk	
			Interaction between context and intervention:	High risk	
			Intervention fidelity:	High risk	
**Casteel, C** (2004) (US) **ITS**	Effectiveness of crime prevention through environmental design in reducing criminal activity in liquor stores: A pilot study	**Industrial activity (NACE):** G—Wholesale and retail trade Occupational activity: sales persons Work setting: 040 tertiary activity area (such as office, teaching establishment, restaurant etc.), incl retail	**2.2.5 Administrative controls** Rationale of intervention: Based on previous experience this approach—CPTED concept uses a behavioral approach (teaching sales persons) and environmental approach (reduce available cash, light, and escape routes.		**Outcome measure: Risk or behavior** Type of accidents: 1 = All types of accident Type of injuries: 888 = All types of injuries
			Theoretical or conceptual framework:	Low risk	
			Interaction between context and intervention:	Low risk	
			Intervention fidelity:	Low risk	
**Benavides, F** (2009) (ES) **ITS**	Effectiveness of occupational Injury prevention policies in Spain	**Industrial activity (NACE):** All or mixed industries Occupational activity: Mixed; Manufacturing and private service (and population at risk was salaried workers only) Work setting: 888 All or mixed setting	**2.2.7 Enforcement of laws and regulations** Rationale of intervention: Focus was on high risk companies with the goal of reinforcing the labor inspectorate; Checks to see if companies are fulfilling required responsibilities, offer suggestions and deadlines to meet. Policy/action plans at the national/regional level presents opportunity for relatively broad improvement in injury prevention. Designed to be tailored approaches (at regional level) to improving safety in high risk companies through inspection, identification of injury prevention needs, offering solutions, and setting a timeline for changes to be in place. Higher quality PAPs should be more effective than lower quality PAPs (or no PAPs at all).		**Outcome measure: Injury** Type of accidents: 6 = Trapped, crushed, struck by equipment or objects Type of injuries: 888 = All types of injuries
			Theoretical or conceptual framework:	Low risk	
			Interaction between context and intervention:	High risk	
			Intervention fidelity:	Low risk	
**Farina, E** (2013) (IT) **ITS**	Are regulations effective in reducing construction injuries? An analysis of the Italian context.	**Industrial activity (NACE):** F—Construction Occupational activity: Construction workers Work setting: 020 Construction site	**'2.2.7 Enforcement of laws and regulations** Rationale of intervention: Discussion of enforced regulation. Based on belief, most injuries are due to failures before starting work and, particularly with fatalities, are caused by activities taking place simultaneously. Thus pre plans should reduce danger if enforced. These laws went beyond just describing minimum safety and health requirements by requiring the creation of safety‐specific roles and plans, which must be in place before starting work.		**Outcome measure: Injury** Type of accidents: 1 = All types of accident Type of injuries: 888 = All types of injuries
			Theoretical or conceptual framework:	Low risk	
			Interaction between context and intervention:	Low risk	
			Intervention fidelity:	Unclear	
**Foley, M** (2012) (US) **CBA**	The impact of regulatory enforcement and consultation visits on workers' compensation claims incidence rates and costs, 1999–2008 Study arm: Fixed sites with enforcement	**Industrial activity (NACE):** All or mixed industries Occupational activity: mixed, all Work setting: 888 All or mixed setting	**2.2.7 Enforcement of laws and regulations** Rationale of intervention: Interventions are required to set and enforce OHS standards and regulations to reduce injury and illness rates, although several preconditions must exist for such reductions to occur.		**Outcome measure: Injury** Type of accidents: 8 = overexertion of the musculoskeletal system Type of injuries: 030 = overexertion injuries (dislocations, sprains and strains)
			Theoretical or conceptual framework:	Low risk	
			Interaction between context and intervention:	Unclear	
			Intervention fidelity:	Low risk	
**Foley, M** (2012) (US) **CBA**	The impact of regulatory enforcement and consultation visits on workers' compensation claims incidence rates and costs, 1999–2008. Study arm: Non‐fixed sites with enforcement	**Industrial activity (NACE):** All or mixed industries Occupational activity: mixed, all Work setting: 888 All or mixed setting	**2.2.7 Enforcement of laws and regulations** Rationale of intervention: Interventions are required to set and enforce OHS standards and regulations to reduce injury and illness rates, although several preconditions must exist for such reductions to occur.		**Outcome measure: Injury** Type of accidents: 8 = overexertion of the musculoskeletal system Type of injuries: 030 = overexertion injuries (dislocations, sprains and strains)
			Theoretical or conceptual framework:	Low risk	
			Interaction between context and intervention:	Unclear	
			Intervention fidelity:	Low risk	
**Haviland, AM** (2012) (US) **CBA**	A new estimate of the impact of OSHA inspections on manufacturing injury rates, 1998–2005 Study arm: Complaint inspections	**Industrial activity (NACE):** C—Manufacturing Occupational activity: static Work setting: 010 Industrial site	**2.2.7 Enforcement of laws and regulations** Rationale of intervention: Specific and general deterrence post inspection and penalty. Inspections of worksites should motivate employers to comply with OSH standards.		**Outcome measure: Injury** Type of accidents: 1 = All types of accident Type of injuries: 888 = All types of injuries
			Theoretical or conceptual framework:	Low risk	
			Interaction between context and intervention:	Low risk	
			Intervention fidelity:	Low risk	
**Haviland, AM** (2012) (US) **CBA**	A new estimate of the impact of OSHA inspections on manufacturing injury rates, 1998–2005 Study arm: Complaint inspections with penalty	**Industrial activity (NACE):** C—Manufacturing Occupational activity: static Work setting: 010 Industrial site	**2.2.7 Enforcement of laws and regulations** Rationale of intervention: Specific and general deterrence post inspection and penalty. Inspections of worksites should motivate employers to comply with OSH standards.		**Outcome measure: Injury** Type of accidents: 1 = All types of accident Type of injuries: 888 = All types of injuries
			Theoretical or conceptual framework:	Low risk	
			Interaction between context and intervention:	Low risk	
			Intervention fidelity:	Low risk	
**Haviland, AM** (2012) (US) **CBA**	A new estimate of the impact of OSHA inspections on manufacturing injury rates, 1998–2005 Study arm: Programmed inspections	**Industrial activity (NACE):** C—Manufacturing Occupational activity: static Work setting: 010 Industrial site	**2.2.7 Enforcement of laws and regulations** Rationale of intervention: Specific and general deterrence post inspection and penalty. Inspections of worksites should motivate employers to comply with OSH standards.		**Outcome measure: Injury** Type of accidents: 1 = All types of accident Type of injuries: 888 = All types of injuries
			Theoretical or conceptual framework:	Low risk	
			Interaction between context and intervention:	Low risk	
			Intervention fidelity:	Low risk	
**Haviland, AM** (2012) (US) **CBA**	A new estimate of the impact of OSHA inspections on manufacturing injury rates, 1998–2005 Study arm: Programmed inspections with penalty	**Industrial activity (NACE):** C—Manufacturing Occupational activity: static Work setting: 010 Industrial site	**2.2.7 Enforcement of laws and regulations** Rationale of intervention: Specific and general deterrence post inspection and penalty. Inspections of worksites should motivate employers to comply with OSH standards.		**Outcome measure: Injury** Type of accidents: 1 = All types of accident Type of injuries: 888 = All types of injuries
			Theoretical or conceptual framework:	Low risk	
			Interaction between context and intervention:	Low risk	
			Intervention fidelity:	Low risk	
**Hogg‐Johnson, S** (2012) (CA) **RCT**	A randomized controlled study to evaluate the effectiveness of targeted occupational health and safety consultation or inspection in Ontario manufacturing workplaces Study arm: Legal enforcement	**Industrial activity (NACE):** C—Manufacturing Occupational activity: Mixed Work setting: 010 Industrial site	**2.2.7 Enforcement of laws and regulations** Rationale of intervention: The implicit assumption is that consultation by Health & Safety Association (HSA) or Ontario Workplace Safety and Insurance Board (WSIB) on OHS matters should decrease occupational injuries.		**Outcome measure: Injury** Type of accidents: 1 = All types of accident Type of injuries: 888 = All types of injuries
			Theoretical or conceptual framework:	Low risk	
			Interaction between context and intervention:	Low risk	
			Intervention fidelity:	High risk	
**Levine, DI** (2012) (US) **CBA**	Randomized government safety inspections reduce worker injuries with no detectable job loss.	**Industrial activity (NACE):** All or mixed industries Occupational activity: Mixed Work setting: 888 All or mixed setting	**2.2.7 Enforcement of laws and regulations** Rationale of intervention: Inspections identify hazards and guidance given to control; financial costs if cited.		**Outcome measure: Injury** Type of accidents: 1 = All types of accident Type of injuries: 888 = All types of injuries
			Theoretical or conceptual framework:	Low risk	
			Interaction between context and intervention:	Low risk	
			Intervention fidelity:	Low risk	
**Lopez‐Ruiz, M** (2014) (ES) **ITS**	Impact of road safety interventions on traffic‐related occupational injuries in Spain. Study arm: Revised penalty code	**Industrial activity (NACE):** H—Transporting and storage Occupational activity: ALL Work setting: 060 Public area (postal courrier)	**2.2.7 Enforcement of laws and regulations** Rationale of intervention: In Spain, the decreasing trend in road traffic injuries, in general, has been attributed to the effectiveness of road safety interventions. Many occupational injuries involve traffic collisions. These road safety interventions should also reduce traffic‐related occupational injuries.		**Outcome measure: Injury** Type of accidents: 5 = Collision and other horizontal impact on body Type of injuries: 888 = All types of injuries
			Theoretical or conceptual framework:	Low risk	
			Interaction between context and intervention:	High risk	
			Intervention fidelity:	High risk	
**Lopez‐Ruiz, M** (2014) (ES) **ITS**	Impact of road safety interventions on traffic‐related occupational injuries in Spain. Study arm: Penalty point system	**Industrial activity (NACE):** H—Transporting and storage Occupational activity: ALL Work setting: 060 Public area (postal courrier)	**2.2.7 Enforcement of laws and regulations** Rationale of intervention: In Spain, the decreasing trend in road traffic injuries, in general, has been attributed to the effectiveness of road safety interventions. Many occupational injuries involve traffic collisions. These road safety interventions should also reduce traffic‐related occupational injuries.		**Outcome measure: Injury** Type of accidents: 5 = Collision and other horizontal impact on body Type of injuries: 888 = All types of injuries
			Theoretical or conceptual framework:	Low risk	
			Interaction between context and intervention:	High risk	
			Intervention fidelity:	High risk	
**Phillips, EK** (2012) (US) **ITS**	Percutaneous injuries before and after the Needlestick Safety and Prevention Act	**Industrial activity (NACE):** Q—Human health and social work activities Occupational activity: health care workers in hospitals Work setting: 050 Health establishment	**2.2.7 Enforcement of laws and regulations** Rationale of intervention: Removes exposure with safety‐engineered devices. NSPA mandated that hospitals use safety engineered devices, which were readily available to hospitals at this time. The implementation of the NSPA also coincided with an increase in the number of OSHA citations for violation of the revised standard.		**Outcome measure: Injury** Type of accidents: 7 = contact with sharp or pointed materials or tools Type of injuries: 015 = Needlestick Injuries
			Theoretical or conceptual framework:	Low risk	
			Interaction between context and intervention:	Unclear	
			Intervention fidelity:	Low risk	
**Chapman, L** (2011) (US) **ITS**	A 7‐year intervention to increase adoption of safer dairy farming work practices	**Industrial activity (NACE):** A—Agriculture, forestry and fishing Occupational activity: Dairy farm managers; USA Work setting: 31 Farming and forestry	**2.2.8 Social marketing and other approaches** Rationale of intervention: socially acceptable—and already utilized channels of dissemination were considered to increase awareness and encourage adoption, and also considered (cost) effective.		**Outcome measure: Risk or behavior** Type of accidents: 1 = All types of accident Type of injuries: 888 = All types of injuries
			Theoretical or conceptual framework:	Low risk	
			Interaction between context and intervention:	High risk	
			Intervention fidelity:	Low risk	

**Table 15:** Nature of included multifaceted safety interventions, by type of safety intervention

3.0.0 Multifaceted interventions						
	Title	Participants	Intervention characteristics			Outcome
**Forst, L** (2004) (US) **CBA**	Effectiveness of community health workers for promoting use of safety eyewear by Latino farm workers Study arm: Introduction of safety training and safety information	**Industrial activity (NACE):** A—Agriculture, forestry and fishing Occupational activity: Crop and animal production, hunting and related service activities Work setting: 030 Farming and forestry	**3.1 Multifaceted at the individual level** Rationale of intervention: The role of Community Health Worker (CHW) models is to connecting people with available services, bridging cultural gaps between communities and building capacity of communities and individuals to get their own health care needs met.			**Outcome measure: Risk or behavior** Type of accidents: 10 = Other specific types of accident Type of injuries: 018 = Eye Injuries
			Theoretical or conceptual framework:		Low risk	
			Interaction between context and intervention:		Unclear	
			Intervention fidelity:		High risk	
**Kim, P** (2004) (CA) **CBA**	The cost‐effectiveness of a back education program for firefighters: a case study	**Industrial activity (NACE):** O—Public administration and defence Occupational activity: Public administration and defence; compulsory social security Work setting: 130 Emergency, rescuing and military sites	**3.1 Multifaceted at the individual level** Rationale of intervention: Based on previous review on back education programs. The present program will be effective because it empowers the worker, change their behavior and attitudes, to change habits and emphasizing that most low back pain is controllable and the individual is key.			**Outcome measure: Injury** Type of accidents: 8 = overexertion of the musculoskeletal system Type of injuries: 030 = overexertion injuries (dislocations, sprains and strains)
			Theoretical or conceptual framework:		Low risk	
			Interaction between context and intervention:		Unclear	
			Intervention fidelity:		Low risk	
**Knapik, JJ** (2004) (US) **CBA**	Influence of an injury reduction program on injury and fitness outcomes among soldiers Study army: Males	**Industrial activity (NACE):** O—Public administration and defence Occupational activity: Public administration and defence; compulsory social security Work setting: 130 Emergency, rescuing and military sites	**3.1 Multifaceted at the individual level** Rationale of intervention: Physical Readiness Training (PRT)differs from current training practices in that it de‐emphasizes running, and incorporates procedures and principles designed to reduce injuries and increase functional fitness.			**Outcome measure: Injury** Type of accidents: 1 = All types of accident Type of injuries: 888 = All types of injuries
			Theoretical or conceptual framework:		Low risk	
			Interaction between context and intervention:		Unclear	
			Intervention fidelity:		Low risk	
**Knapik, JJ** (2004) (US) **CBA**	Influence of an injury reduction program on injury and fitness outcomes among soldiers Study arm: Females	**Industrial activity (NACE):** O—Public administration and defence Occupational activity: Public administration and defence; compulsory social security Work setting: 130 Emergency, rescuing and military sites	**3.1 Multifaceted at the individual level** Rationale of intervention: Physical Readiness Training (PRT)differs from current training practices in that it de‐emphasizes running, and incorporates procedures and principles designed to reduce injuries and increase functional fitness.			**Outcome measure: Injury** Type of accidents: 1 = All types of accident Type of injuries: 888 = All types of injuries
			Theoretical or conceptual framework:		Low risk	
			Interaction between context and intervention:		Unclear	
			Intervention fidelity:		Low risk	
**Peate, WF** (2007) (US) **CBA**	Core strength: a new model for injury prediction and prevention	**Industrial activity (NACE):** O—Public administration and defence Occupational activity: Public administration and defence; compulsory social security Work setting: 130 Emergency, rescuing and military sites	**3.1 Multifaceted at the individual level** Rationale of intervention: Current research suggests that training may increase core strength and decrease musculoskeletal damage, because improvements in core or static strength, flexibility and the three dimensions of movement: acceleration; deceleration; and dynamic stabilization is proposed as injury prevention possibilities.			**Outcome measure: Injury** Type of accidents: 1 = All types of accident Type of injuries: 030 = overexertion injuries (dislocations, sprains and strains)
			Theoretical or conceptual framework:		Low risk	
			Interaction between context and intervention:		Unclear	
			Intervention fidelity:		Unclear	
**Rasmussen, K** (2003) (DK) **RCT**	Prevention of farm injuries in Denmark	**Industrial activity (NACE):** A—Agriculture, forestry and fishing Occupational activity: Crop and animal production, hunting and related service activities Work setting: 030 Farming and forestry	**3.1 Multifaceted at the individual level** Rationale of intervention: Authors state that the dialogue with the farmers initiate a catalytic process which should increase farmers' motivation to act, by making risks emotionally salient and getting farmers to commit themselves to make changes at the farm.			**Outcome measure: Injury** Type of accidents: 1 = All types of accident Type of injuries: 888 = All types of injuries
			Theoretical or conceptual framework:		Low risk	
			Interaction between context and intervention:		High risk	
			Intervention fidelity:		Low risk	
**Rautiainen, RH** (2004) (US) **RCT**	Injuries in the Iowa Certified Safe Farm Study	**Industrial activity (NACE):** A—Agriculture, forestry and fishing Occupational activity: Crop and animal production, hunting and related service activities Work setting: 030 Farming and forestry	**3.1 Multifaceted at the individual level** Rationale of intervention: Based on previous research it is assumed that multifaceted programs are most successful. This include detect problems, remove hazards, educate farmers and share experiences on cost savings.			**Outcome measure: Injury** Type of accidents: 1 = All types of accident Type of injuries: 888 = All types of injuries
			Theoretical or conceptual framework:		Low risk	
			Interaction between context and intervention:		High risk	
			Intervention fidelity:		High risk	
**Santaweesuk, S** (2014) (TH) **CBA**	Effects of an injury and illness prevention program on occupational safety behaviors among rice farmers in Nakhon Nayok Province, Thailand	**Industrial activity (NACE):** A—Agriculture, forestry and fishing Occupational activity: Crop and animal production, hunting and related service activities Work setting: 030 Farming and forestry	**3.1 Multifaceted at the individual level** Rationale of intervention: Four elements were covered: 1) health education, 2) safety inspection, 3) safety communication, and 4) health surveillance. It was expected that this program could offer rice farmers an effective and efficient approach to improving safety behavior and their work environment.			**Outcome measure: Risk or behavior** Type of accidents: 1 = All types of accident Type of injuries: 888 = All types of injuries
			Theoretical or conceptual framework:		High risk	
			Interaction between context and intervention:		High risk	
			Intervention fidelity:		High risk	
**Bell, J** (2006) (US) **ITS**	Evaluating the effectiveness of a logger safety training program	**Industrial activity (NACE):** A—Agriculture, forestry and fishing Occupational activity: logging/dynamic Work setting: 030 Farming and forestry	**3.2 Multifaceted at the group or organizational level** Rationale of intervention: Trading and best practices should increase safety. Site visits are further incentives to comply with standards of practice. On top of that there was also an economic incentive (reduced premium rates) and penalty (expulsion from the program).			**Outcome measure: Injury** Type of accidents: 1 = All types of accident Type of injuries: 888 = All types of injuries
			Theoretical or conceptual framework:		Low risk	
			Interaction between context and intervention:		High risk	
			Intervention fidelity:		Low risk	
**Carrivick, PJW** (2002) (AU) **CBA**	Effectiveness of a workplace risk assessment team in reducing the rate, cost, and duration of occupational injury	**Industrial activity (NACE):** N—Administrative and support service activities Occupational activity: Services to buildings and landscape activities Work setting: 050 Health establishment	**3.2 Multifaceted at the group or organizational level** Rationale of intervention: Application of an iterative injury risk identification, assessment and control process. Will achieve a sustained reduction in overall injuries.			**Outcome measure: Injury** Type of accidents: 1 = All types of accident Type of injuries: 888 = All types of injuries
			Theoretical or conceptual framework:		Low risk	
			Interaction between context and intervention:		Unclear	
			Intervention fidelity:		High risk	
**Chhokar, R** (2005) (CA) **ITS**	The 3‐year economic benefits of a ceiling lift intervention aimed to reduce healthcare worker injuries.	**Industrial activity (NACE):** Q—Human health and social work activities Occupational activity: human health activities (long‐term care facility)/dynamic Work setting: 050 Health establishment	**3.2 Multifaceted at the group or organizational level** Rationale of intervention: Celling lifts is close and more convenient, combined w policy and training and thereby reduce injuries to health care workers.			**Outcome measure: Injury** Type of accidents: 8 = overexertion of the musculoskeletal system Type of injuries: 030 = overexertion injuries (dislocations, sprains and strains)
			Theoretical or conceptual framework:		Low risk	
			Interaction between context and intervention:		High risk	
			Intervention fidelity:		Unclear	
**Fujishiro, K** (2005) (US) **ITS**	The effect of ergonomic interventions in healthcare facilities on musculoskeletal disorders	**Industrial activity (NACE):** Q—Human health and social work activities Occupational activity: Primarily long term care staff Work setting: 050 Health establishment	**3.2 Multifaceted at the group or organizational level** Rationale of intervention: The Program offered financial support and ergonomic consultation to facilities for installing ergonomic devices to reduce the risk of injuries. Workplaces had self‐choice of most relevant ergonomic device.			**Outcome measure: Injury** Type of accidents: 8 = overexertion of the musculoskeletal system Type of injuries: 030 = overexertion injuries (dislocations, sprains and strains)
			Theoretical or conceptual framework:		Low risk	
			Interaction between context and intervention:		Unclear	
			Intervention fidelity:		Unclear	
**Gershon, R** (1999) (US) **ITS**	The impact of multifocused interventions on sharps injury rates at an acute‐care hospital.	**Industrial activity (NACE):** Q—Human health and social work activities Occupational activity: Human health activities/dynamic Work setting: 050 Health establishment	**3.2 Multifaceted at the group or organizational level** Rationale of intervention: Based on literature that multifaceted programs that include engineering changes have lasting effect on NSI reduction.			**Outcome measure: Injury** Type of accidents: 7 = contact with sharp or pointed materials or tools Type of injuries: 015 = Needlestick Injuries
			Theoretical or conceptual framework:		Low risk	
			Interaction between context and intervention:		Unclear	
			Intervention fidelity:		Low risk	
**López‐Ruiz, M** (2013) (ES) **CBA**	Evaluation of the effectiveness of occupational injury prevention programs at the company level	**Industrial activity (NACE):** All or mixed industries Occupational activity: Mixed (3 different sectors) Work setting: 888 All or mixed setting	**3.2 Multifaceted at the group or organizational level** Rationale of intervention: The main goal of preferential action plans (PAP) is to promoted the prevention of occupational injuries through intervention in high companies by introducing a number of programmes at organizational and workgroup level.			**Outcome measure: Injury** Type of accidents: 1 = All types of accident Type of injuries: 999 = other specified injuries not incl in other headings
			Theoretical or conceptual framework:		Unclear	
			Interaction between context and intervention:		High risk	
			Intervention fidelity:		Unclear	
**Park, R** (2009) (US) **ITS**	Impact of publicly sponsored interventions on musculoskeletal injury claims in nursing homes.	**Industrial activity (NACE):** Q—Human health and social work activities Occupational activity: nursing home staff/dynamic Work setting: 050 Health establishment	**3.2 Multifaceted at the group or organizational level** Rationale of intervention: Decreased physical exposures and provided consultation to implement, resources from policy initiative that smaller nursing homes might not have. Education and resources would increase knowledge and engineering would decreases load on the lower back when lifting.			**Outcome measure: Injury** Type of accidents: 8 = overexertion of the musculoskeletal system Type of injuries: 030 = overexertion injuries (dislocations, sprains and strains)
			Theoretical or conceptual framework:		Low risk	
			Interaction between context and intervention:		Low risk	
			Intervention fidelity:		Low risk	
**Zafar, AB** (1997) (US) **ITS**	Effect of a comprehensive program to reduce needlestick injuries	**Industrial activity (NACE):** Q—Human health and social work activities Occupational activity: Human health activities Work setting: 050 Health establishment	**3.2 Multifaceted at the group or organizational level** Rationale of intervention: Multi‐component including engineering; Organizational approach, education and awareness including engineering control, should modify HCW behavior and create awareness, and in turn reduce number of needlesticks.			**Outcome measure: Injury** Type of accidents: 7 = contact with sharp or pointed materials or tools Type of injuries: 015 = Needlestick Injuries
			Theoretical or conceptual framework:	Low risk		
			Interaction between context and intervention:	Unclear		
			Intervention fidelity:	High risk		
**Bell, J** (2008) (US) **ITS**	Evaluation of a comprehensive slip, trip, and fall prevention programme for hospital employees	**Industrial activity (NACE):** Q—Human health and social work activities Occupational activity: hospital workers/dynamic Work setting: 050 Health establishment	**3.3 Multifaceted across individual and organizational levels** Rationale of intervention: The programme was aiming the mitigation of extrinsic hazards inside and outside the hospitals. The intervention works by better control, or elimination of hazards. Well described in the paper (p. 1909). Multi‐causal problems with multi‐faceted intervention based on hazard assessments.			**Outcome measure: Injury** Type of accidents: 4 = Tripping, stumbling and falling Type of injuries: 999 = other specified injuries not incl in other headings
			Theoretical or conceptual framework:	Low risk		
			Interaction between context and intervention:	Low risk		
			Intervention fidelity:	Unclear		
**Black, TR** (2011) (CA) **CBA**	Effect of transfer, lifting, and repositioning (TLR) injury prevention program on musculoskeletal injury among direct care workers	**Industrial activity (NACE):** Q—Human health and social work activities Occupational activity: Residential care activities Work setting: 050 Health establishment	**3.3 Multifaceted across individual and organizational levels** Rationale of intervention: Based on previous literature that a Transfer, Lifting, and Repositioning (TLR) program should work by defining, assessing, and standardizing patient handling requirements and procedures for each individual patient to ensure both patient and worker safety, including reinforcement of good practice.			**Outcome measure: Injury** Type of accidents: 8 = overexertion of the musculoskeletal system Type of injuries: 030 = overexertion injuries (dislocations, sprains and strains)
			Theoretical or conceptual framework:	Low risk		
			Interaction between context and intervention:	High risk		
			Intervention fidelity:	High risk		
**Bull, N** (2007) (NO) **ITS**	Mandatory use of eye protection prevents eye injuries in the metal industry	**Industrial activity (NACE):** C—Manufacturing Occupational activity: Metal workers (large hulls etc.) Work setting: 010 Industrial site	**3.3 Multifaceted across individual and organizational levels** Rationale of intervention: Safe glasses and goggles were made available (PPE) and management enforced its use.			**Outcome measure: Injury** Type of accidents: 10 = Other specific types of accident Type of injuries: 018 = Eye Injuries
			Theoretical or conceptual framework:	Low risk		
			Interaction between context and intervention:	Low risk		
			Intervention fidelity:	Low risk		
**Evanoff, BA** (1999) (US) **CBA**	Effects of a participatory ergonomics team among hospital orderlies	**Industrial activity (NACE):** Q—Human health and social work activities Occupational activity: Human health activities Work setting: 050 Health establishment	**3.3 Multifaceted across individual and organizational levels** Rationale of intervention: PE programs brings together workers and management to work cooperatively to identify safety and health problems, and to implement appropriate changes in work practices or job design. In contrast to the more common “top‐down” safety programs, participatory ergonomics teams may more effectively take advantage of worker knowledge and problemsolving skills, reduce resistance to change, and improve workplace communication and worker motivation.			**Outcome measure: Injury** Type of accidents: 8 = overexertion of the musculoskeletal system Type of injuries: 030 = overexertion injuries (dislocations, sprains and strains)
			Theoretical or conceptual framework:	Low risk		
			Interaction between context and intervention:	High risk		
			Intervention fidelity:	Low risk		
**Garg, A** (1999) (US) **ITS**	Long‐term effectiveness of 'Zero‐Lift Program' in seven nursing homes and one hospital	**Industrial activity (NACE):** Q—Human health and social work activities Occupational activity: health care—7 nursing homes and 1 hospital Work setting: 050 Health establishment	**3.3 Multifaceted across individual and organizational levels** Rationale of intervention: The "zero‐lift program" work as it replaced manual lifting and transferring of patients, with modern, battery operated, portable hoists and other patient transfer assistive devices.			**Outcome measure: Injury** Type of accidents: 8 = overexertion of the musculoskeletal system Type of injuries: 030 = overexertion injuries (dislocations, sprains and strains)
			Theoretical or conceptual framework:	Low risk		
			Interaction between context and intervention:	Low risk		
			Intervention fidelity:	Unclear		
**Lipscomb, HJ** (2010) (US) **ITS**	Continued progress in the prevention of nail gun injuries among apprentice carpenters: what will it take to see wider spread injury reductions?	**Industrial activity (NACE):** F—Construction Occupational activity: Specialized construction activities; carpenters Work setting: 020 Construction site	**3.3 Multifaceted across individual and organizational levels** Rationale of intervention: Defined risk factors are being addressed with training and safer tool (nail gun trigger mechanism).			**Outcome measure: Injury** Type of accidents: Type of injuries: 888 = All types of injuries
			Theoretical or conceptual framework:	Low risk		
			Interaction between context and intervention:	Unclear		
			Intervention fidelity:	Low risk		
**Mancini, G** (2005) (IT) **ITS**	Prevention of work‐related eye injuries: long term assessment of the effectiveness of a multicomponent intervention among metal workers	**Industrial activity (NACE):** C—Manufacturing Occupational activity: Metal workers factory/static Work setting: 010 Industrial site	**3.3 Multifaceted across individual and organizational levels** Rationale of intervention: Tailored educational brochure combined with inspection with legal powers should raise the use of protective eye‐wear, which in turn should lower the number of eye injuries.			**Outcome measure: Injury** Type of accidents: 10 = Other specific types of accident Type of injuries: 018 = Eye Injuries
			Theoretical or conceptual framework:	Low risk		
			Interaction between context and intervention:	Low risk		
			Intervention fidelity:	Low risk		
**Martin, P** (2009) (AU) **ITS**	Effect of a nurse back injury prevention intervention on the rate of injury compensation claims	**Industrial activity (NACE):** Q—Human health and social work activities Occupational activity: Human health and activities/dynamic Work setting: 050 Health establishment	**3.3 Multifaceted across individual and organizational levels** Rationale of intervention: No lift policies' bring about changes in the physical workload, by replacing manual lifting and transferring of patients with modern hoists and other patient transfer devices.			**Outcome measure: Injury** Type of accidents: 8 = overexertion of the musculoskeletal system Type of injuries: 030 = overexertion injuries (dislocations, sprains and strains)
			Theoretical or conceptual framework:	Low risk		
			Interaction between context and intervention:	Low risk		
			Intervention fidelity:	Unclear		
**Miller, TR** (2007) (US) **ITS**	Effectiveness and benefit‐cost of peer‐based workplace substance abuse prevention coupled with random testing.	**Industrial activity (NACE):** H—Transporting and storage Occupational activity: Transportation and storage/mixed Work setting: 060 Public area	**3.3 Multifaceted across individual and organizational levels** Rationale of intervention: The intervention should work by changing occupational norms that condone this behavior. This union‐management partnership uses the occupational peer group to achieve a cultural shift from enabling working under the influence of drugs or alcohol to maintaining a substance‐free workplace. In exchange for employee efforts, management moves from a punitive approach to supportive and restorative aid for substance abusers.			**Outcome measure: Injury** Type of accidents: 1 = All types of accident Type of injuries: 888 = All types of injuries
			Theoretical or conceptual framework:	Low risk		
			Interaction between context and intervention:	Low risk		
			Intervention fidelity:	Low risk		
**Mode, NA** (2012) (US) **ITS**	A multifaceted public health approach to statewide aviation safety.	**Industrial activity (NACE):** H—Transporting and storage Occupational activity: Dynamic (highly dynamic) Work setting: 090 In the air, elevated except construction	**3.3 Multifaceted across individual and organizational levels** Rationale of intervention: Basic public health approach. The multifaceted approach to improve aviation safety using the public health approach to injury prevention. This included employing technology, providing education to pilots and consumers, and by encouraging voluntary changes to improve safety within the aviation industry.			**Outcome measure: Injury** Type of accidents: 5 = Collision and other horizontal impact on body Type of injuries: 999 = other specified injuries not incl in other headings
			Theoretical or conceptual framework:	Low risk		
			Interaction between context and intervention:	High risk		
			Intervention fidelity:	Low risk		
**Parker, DL** (2009) (US) **CBA**	A randomized, controlled intervention of machine guarding and related safety programs in small metal‐fabrication businesses	**Industrial activity (NACE):** C—Manufacturing Occupational activity: metal workers Work setting: 010 Industrial site	**3.3 Multifaceted across individual and organizational levels** Rationale of intervention: Proper use of machine guarding should protect worker from injuries as previous research indicate that lack of machine guarding increase risk of accidents for workers. The intervention also included hazard identification and control, training, and persuasive materials and improved involvement of workers in decision making.			**Outcome measure: Risk or behavior** Type of accidents: 6 = Trapped, crushed, struck by equipment or objects Type of injuries: 888 = All types of injuries
			Theoretical or conceptual framework:	Low risk		
			Interaction between context and intervention:	Unclear		
			Intervention fidelity:	Low risk		
**Passfield, J** (2003) (AU) **ITS**	"No lift" patient handling policy implementation and staff injury rates in a public hospital.	**Industrial activity (NACE):** Q—Human health and social work activities Occupational activity: Human health activities; nurses/dynamic Work setting: 050 Health establishment	**3.3 Multifaceted across individual and organizational levels** Rationale of intervention: Multi components with “no lift” policy backup and lift equipment, training support etc.			**Outcome measure: Injury** Type of accidents: 8 = overexertion of the musculoskeletal system Type of injuries: 030 = overexertion injuries (dislocations, sprains and strains)
			Theoretical or conceptual framework:	Low risk		
			Interaction between context and intervention:	High risk		
			Intervention fidelity:	Low risk		
**Peek‐Asa, C** (2004) (US) **RCT**	Compliance to a workplace violence prevention program in small businesses	**Industrial activity (NACE):** G—Wholesale and retail trade Occupational activity: Sales persons Work setting: 040 tertiary activity area (such as office, teaching establishment, restaurant etc.), incl retail	**3.3 Multifaceted across individual and organizational levels** Rationale of intervention: Using Crime Prevention Through Environmental Design (CPTED), which is based on the principle that crime can be reduced by controlling the business environment.			**Outcome measure: Injury** Type of accidents: 9 = Assault or violence at work. Type of injuries: Not reported
			Theoretical or conceptual framework:	Low risk		
			Interaction between context and intervention:	High risk		
			Intervention fidelity:	Low risk		
**Porru, S** (2011) (IT) **ITS**	An effectiveness evaluation of a multifaceted preventive intervention on occupational injuries in foundries: a 13‐Year follow‐up study with interrupted times series analysis.	**Industrial activity (NACE):** C—Manufacturing Occupational activity: Foundries; one cast‐iron and one non‐ferrous Work setting: 010 Industrial site	**3.3 Multifaceted across individual and organizational levels** Rationale of intervention: Multifaceted approach with many components.			**Outcome measure: Injury** Type of accidents: 1 = All types of accident Type of injuries: 888 = All types of injuries
			Theoretical or conceptual framework:	High risk		
			Interaction between context and intervention:	Unclear		
			Intervention fidelity:	High risk		
**Rasmussen, K** (2006) (DK) **CBA**	Worker participation in change processes in a Danish industrial setting	**Industrial activity (NACE):** C—Manufacturing Occupational activity: Manufacture of machinery and equipment. Work setting: 010 Industrial site	**3.3 Multifaceted across individual and organizational levels** Rationale of intervention: A participatory action research (PAR) approach was adopted. interventions need to involve both management and employees in the conceptualization, design, and implementation processes.			**Outcome measure: Injury** Type of accidents: 1 = All types of accident Type of injuries: 888 = All types of injuries
			Theoretical or conceptual framework:	Low risk		
			Interaction between context and intervention:	Low risk		
			Intervention fidelity:	Low risk		
**Saari, J** (1989) (FI) **ITS**	The effect of positive feedback on industrial housekeeping and accidents; A long‐term study at a shipyard	**Industrial activity (NACE):** C—Manufacturing Occupational activity: Manufacture of other transport equipment; shipyard workers/both Work setting: 11 Industrial site	**3.3 Multifaceted across individual and organizational levels** Rationale of intervention: Behavior modification built on the provision on positive consequences for desired safety behavior. Based on evidence that reinforcement modifies many behaviors. But has lesser influence on injuries.			**Outcome measure: Injury** Type of accidents: 1 = All types of accident Type of injuries: 888 = All types of injuries
			Theoretical or conceptual framework:	Low risk		
			Interaction between context and intervention:	High risk		
			Intervention fidelity:	Low risk		
**Spangenberg, S** (2002) (DK) **ITS**	The construction of the Øresund Link between Denmark and Sweden: the effect of a multi‐faceted safety campaign.	**Industrial activity (NACE):** F—Construction Occupational activity: Construction work Work setting: 020 Construction site	**3.3 Multifaceted across individual and organizational levels** Rationale of intervention: Based on past literature on behavioral change. The intervention does not appear to have been designed by the researchers (although this is not explicit). Information on on‐site safety was provided, and feedback with results coupled to economic incentives and in competition with other groups was expected to have an immediate, but not sustained post‐intervention effect.			**Outcome measure: Injury** Type of accidents: 1 = All types of accident Type of injuries: 888 = All types of injuries
			Theoretical or conceptual framework:	Low risk		
			Interaction between context and intervention:	Low risk		
			Intervention fidelity:	High risk		
**Srikrajang, J** (2005) (TH) **RCT**	Effectiveness of education and problem solving work group on nursing practices to prevent needlestick and sharp injury	**Industrial activity (NACE):** Q—Human health and social work activities Occupational activity: Nursing work Work setting: 050 Health establishment	**3.3 Multifaceted across individual and organizational levels** Rationale of intervention: Educational programs, including problem solving working group can be an effective intervention strategy to prevent needlestick and sharp injury in developing countries.			**Outcome measure: Risk or behavior** Type of accidents: 7 = contact with sharp or pointed materials or tools Type of injuries: 015 = Needlestick Injuries
			Theoretical or conceptual framework:	Low risk		
			Interaction between context and intervention:	Unclear		
			Intervention fidelity:	Low risk Low risk		
**Valls, V** (2007) (ES) **CBA**	Use of safety devices and the prevention of percutaneous injuries among healthcare workers	**Industrial activity (NACE):** Q—Human health and social work activities Occupational activity: Human health activities Work setting: 050 Health establishment	**3.3 Multifaceted across individual and organizational levels** Rationale of intervention: Safety devices use engineering control and reduce the human factors.			**Outcome measure: Injury** Type of accidents: 7 = contact with sharp or pointed materials or tools Type of injuries: 015 = Needlestick Injuries
			Theoretical or conceptual framework:	Low risk		
			Interaction between context and intervention:	Unclear		
			Intervention fidelity:	Low risk		
**Wickizer, T** (2004) (US) **ITS**	Do drug‐free workplace programs prevent occuptional injuries? Evidence from Washington State.	**Industrial activity (NACE):** All or mixed industries Occupational activity: Multiple/mixed Work setting: 888 All or mixed setting	**3.3 Multifaceted across individual and organizational levels** Rationale of intervention: The Federal Drug‐Free Workplace Program is a multifaceted approach, non‐punitive, keeps workers from being fired, establishes operating procedures.			**Outcome measure: Injury** Type of accidents: 1 = All types of accident Type of injuries: 888 = All types of injuries
			Theoretical or conceptual framework:	Low risk		
			Interaction between context and intervention:	High risk		
			Intervention fidelity:	Unclear		

**Table 16:** Nature of identified before and after studies, by main type of safety intervention (*n* = 94)

Author (year)	Study title	Type of safety intervention	Participants (Country)	Outcome
**1.1.0 Attitude modification**				
De Souza, RA (2012)	Novel approaches to development, delivery and evaluation of a peer‐led occupational safety training for Latino day laborers	1.1.0 Attitude modification	F—Construction WORK SETTING: 020 Construction site (US)	1 = All types of accident 888 = All types of injuries Outcome measure: Risk or behavior
El Beltagy, K (2012)	Impact of infection control educational activities on rates and frequencies of percutaneous injuries (Pis) at a tertiary care hospital in Saudi Arabia	1.1.0 Attitude modification	Q—Human health and social work activities WORK SETTING: 050 Health establishment (SA)	7 = contact with sharp or pointed materials or tools 015 = Needlestick Injuries Outcome measure: Injury
Arphorn, S (2010)	A program for Thai rubber tappers to improve the cost of occupational health and safety	1.1.2 Counseling approaches	A—Agriculture, forestry and fishing WORK SETTING: 030 Farming and forestry (TH)	1 = All types of accident 888 = All types of injuries Outcome measure: Injury
Chenoweth, C (2013)	Reducing nursing needlestick injuries in hemodialysis clinics: a quality improvement program	1.1.3 Teaching, education to increase knowledge and awareness	Q—Human health and social work activities WORK SETTING: 050 Health establishment (AU + NZ)	7 = contact with sharp or pointed materials or tools 015 = Needlestick Injuries Outcome measure: Injury
Fisher, TF (2015)	Radiologic and sonography professionals' ergonomics: an occupational therapy intervention for preventing work injuries	1.1.9 Other types of attitude modifications	Q—Human health and social work activities WORK SETTING: 050 Health establishment (US)	8 = overexertion of the musculoskeletal system 030 = overexertion injuries (dislocations, sprains and strains) Outcome measure: Risk or behavior
**1.2.0 Behavior modification**				
Evanoff, B (2012)	Outcomes of a revised apprentice carpenter fall prevention training curriculum	1.2.2 Safety training	F—Construction WORK SETTING: 020 Construction site (US)	7 = contact with sharp or pointed materials or tools 888 = All types of injuries Outcome measure: Risk or behavior
Charney, W (1991)	The lifting team. A design method to reduce lost time back injury in nursing	1.2.2 Safety training	Q—Human health and social work activities WORK SETTING: 050 Health establishment (US)	8 = overexertion of the musculoskeletal system 030 = overexertion injuries (dislocations, sprains and strains) Outcome measure: Injury
Lingard, H (2002)	The effect of first aid training on Australian construction workers' occupational health and safety motivation and risk control behavior	1.2.2 Safety training	F—Construction WORK SETTING: 020 Construction site (AU)	1 = All types of accident 888 = All types of injuries Outcome measure: Risk or behavior
Kowalski‐Trakofler, KM (2003)	The concept of degraded images applied to hazard recognition training in mining for reduction of lost‐time injuries	1.2.2 Safety training	B—Mining and quarrying WORK SETTING: 100 Underground (US)	1 = All types of accident 888 = All types of injuries Outcome measure: Injury
McCallum, DM (2005)	Safety‐related knowledge and behavior changes in participants of farm safety day camps	1.2.2 Safety training	A—Agriculture, forestry and fishing WORK SETTING: 030 Farming and forestry (US)	1 = All types of accident 888 = All types of injuries Outcome measure: Risk or behavior
McAdam, TK (2004)	Non‐touch suturing technique fails to reduce glove puncture rates in an accident and emergency department	1.2.2 Safety training	Q—Human health and social work activities WORK SETTING: 050 Health establishment (IE)	7 = contact with sharp or pointed materials or tools 015 = Needlestick Injuries Outcome measure: Other
**1.3.0 Modification of physical strength and resistance**				
Leffer, M (2010)	Implementation of a physician‐organized wellness regime (POWR) enforcing the 2007 NFPA standard 1582: injury rate reduction and associated cost savings	1.3.1 Individual physical training	O—Public administration and defence WORK SETTING: 130 Emergency, rescuing and military sites (US)	1 = All types of accident 888 = All types of injuries Outcome measure: Injury
Wassell, JT (2000)	A prospective study of back belts for prevention of back pain and injury	1.3.2 PPE, back belts and other devices	G—Wholesale and retail trade WORK SETTING: 040 tertiary activity area (such as office, teaching establishment, restaurant etc.), incl retail (US)	1 = All types of accident 888 = All types of injuries Outcome measure: Injury
Roberts, D (2013)	Injury prevention for ski‐area employees: a physiological assessment of lift operators, instructors, and patrollers	1.3.1 Individual physical training	R—Arts, entertainment and recreation WORK SETTING: 080 Sports area (CA)	1 = All types of accident 888 = All types of injuries Outcome measure: Injury
Monaghan, PF (2011)	Preventing eye injuries among citrus harvesters: the Community Health Worker model	1.3.0 Modification of physical strength and resistance	A—Agriculture, forestry and fishing WORK SETTING: 030 Farming and forestry (US)	10 = Other specific types of accident 018 = Eye Injuries Outcome measure: Risk or behavior
Monaghan, PF (2012)	Adoption of safety eyewear among citrus harvesters in rural Florida	1.3.2 PPE, back belts and other devices	A—Agriculture, forestry and fishing WORK SETTING: 030 Farming and forestry (US)	10 = Other specific types of accident 018 = Eye Injuries Outcome measure: Risk or behavior
**2.1.0 Climate modifications**				
Moore‐Ede, M (2004)	Circadian alertness simulator for fatigue risk assessment in transportation: application to reduce frequency and severity of truck accidents	2.1.5 Safety feedback to work groups and leaders	H—Transporting and storage WORK SETTING: 060 Public area (NORTH AMERICA)	1 = All types of accident 888 = All types of injuries Outcome measure: Injury
**2.2.0 Structural modifications**				
Carrivick, PJ (2005)	Evaluating the effectiveness of a participatory ergonomics approach in reducing the risk and severity of injuries from manual handling	2.2.0 Structural modifications	Q—Human health and social work activities WORK SETTING: 050 Health establishment (AU)	8 = overexertion of the musculoskeletal system 030 = overexertion injuries (dislocations, sprains and strains) Outcome measure: Injury
Hooper, J (2005)	Creation of a safety culture: reducing workplace injuries in a rural hospital setting	2.2.0 Structural modifications	Q—Human health and social work activities WORK SETTING: 050 Health establishment (US)	1 = All types of accident 888 = All types of injuries Outcome measure: Injury
Arocena, P (2009)	The effect of occupational safety legislation in preventing accidents at work: traditional versus advanced manufacturing industries	2.2.1 Legislative changes	C—Manufacturing WORK SETTING: 010 Industrial site (ES)	1 = All types of accident 888 = All types of injuries Outcome measure: Injury
Jagger, J (2010)	Increase in sharps injuries in surgical settings versus nonsurgical settings after passage of national needlestick legislation	2.2.1 Legislative changes	Q—Human health and social work activities WORK SETTING: 050 Health establishment (US)	7 = contact with sharp or pointed materials or tools 015 = Needlestick Injuries Outcome measure: Injury
Perry, J (2012)	Disposal of sharps medical waste in the United States: impact of recommendations and regulations, 1987–2007	2.2.1 Legislative changes	Q—Human health and social work activities WORK SETTING: 050 Health establishment (US)	7 = contact with sharp or pointed materials or tools 015 = Needlestick Injuries Outcome measure: Injury
Alvarado‐Ramy, F (2003)	A comprehensive approach to percutaneous injury prevention during phlebotomy: results of a multicenter study, 1993–1995	2.2.4 Engineering controls	Q—Human health and social work activities WORK SETTING: 050 Health establishment (US)	7 = contact with sharp or pointed materials or tools 015 = Needlestick Injuries Outcome measure: Injury
Anyan, W (2013)	Overhead lift systems reduce back injuries among burn care providers	2.2.4 Engineering controls	Q—Human health and social work activities WORK SETTING: 050 Health establishment (US)	8 = overexertion of the musculoskeletal system 030 = overexertion injuries (dislocations, sprains and strains) Outcome measure: Injury
Catalán Gómez, MT (2010)	Implementation of safety devices: biological accident prevention	2.2.4 Engineering controls	Q—Human health and social work activities WORK SETTING: 050 Health establishment (ES)	7 = contact with sharp or pointed materials or tools 015 = Needlestick Injuries Outcome measure: Injury
Gartner, K (1993)	Impact of a needleless intravenous system in a university hospital	2.2.4 Engineering controls	Q—Human health and social work activities WORK SETTING: 050 Health establishment (US)	7 = contact with sharp or pointed materials or tools 015 = Needlestick Injuries Outcome measure: Injury
Grimmond, T (2014)	Sharps injury reduction: a 6‐year, three‐phase study comparing use of a small patient‐room sharps disposal container with a larger engineered container	2.2.4 Engineering controls	Q—Human health and social work activities WORK SETTING: 050 Health establishment (AUSTRALIA)	7 = contact with sharp or pointed materials or tools 015 = Needlestick Injuries Outcome measure: Injury
Grimmond, T (2003)	Sharps injury reduction using Sharpsmart‐‐a reusable sharps management system	2.2.4 Engineering controls	Q—Human health and social work activities WORK SETTING: 050 Health establishment (AU + NZ + GB)	7 = contact with sharp or pointed materials or tools 015 = Needlestick Injuries Outcome measure: Injury
Hatcher, IB (2002)	Reducing sharps injuries among health care workers: a sharps container quality improvement project	2.2.4 Engineering controls	Q—Human health and social work activities WORK SETTING: 050 Health establishment (US)	1 = All types of accident 015 = Needlestick Injuries Outcome measure: Injury
Krasinski, K (1987)	Effect of changing needle disposal systems on needle puncture injuries	2.2.4 Engineering controls	Q—Human health and social work activities WORK SETTING: 050 Health establishment (US)	7 = contact with sharp or pointed materials or tools 015 = Needlestick Injuries Outcome measure: Injury
Li, J (2004)	Use of mechanical patient lifts decreased musculoskeletal symptoms and injuries among health care workers	2.2.4 Engineering controls	Q—Human health and social work activities WORK SETTING: 050 Health establishment (US)	8 = overexertion of the musculoskeletal system 030 = overexertion injuries (dislocations, sprains and strains) Outcome measure: Injury
Makofsky, D (1993)	Installing needle disposal boxes closer to the bedside reduces needle‐recapping rates in hospital units	2.2.4 Engineering controls	Q—Human health and social work activities WORK SETTING: 050 Health establishment (US)	7 = contact with sharp or pointed materials or tools 015 = Needlestick Injuries Outcome measure: Risk or behavior
Menezes, JA (2014)	Impact of a single safety‐engineered device on the occurrence of percutaneous injuries in a general hospital in Brazil	2.2.4 Engineering controls	Q—Human health and social work activities WORK SETTING: 050 Health establishment (BR)	7 = contact with sharp or pointed materials or tools 015 = Needlestick Injuries Outcome measure: Injury
Orenstein, R (1995)	Do protective devices prevent needlestick injuries among health care workers?	2.2.4 Engineering controls	Q—Human health and social work activities WORK SETTING: 050 Health establishment (US)	7 = contact with sharp or pointed materials or tools 015 = Needlestick Injuries Outcome measure: Injury
Peate, WF (2001)	Preventing needlesticks in emergency medical system workers	2.2.4 Engineering controls	O—Public administration and defence WORK SETTING: 130 Emergency, rescuing and military sites (US)	7 = contact with sharp or pointed materials or tools 015 = Needlestick Injuries Outcome measure: Injury
Ribner, BS (1987)	Impact of a rigid, puncture resistant container system upon needlestick injuries	2.2.4 Engineering controls	Q—Human health and social work activities WORK SETTING: 050 Health establishment (US)	7 = contact with sharp or pointed materials or tools 015 = Needlestick Injuries Outcome measure: Injury
Sedlak, CA (2009)	The clinical nurse specialist as change agent: reducing employee injury and related costs	2.2.4 Engineering controls	Q—Human health and social work activities WORK SETTING: 050 Health establishment (US)	8 = overexertion of the musculoskeletal system 030 = overexertion injuries (dislocations, sprains and strains) Outcome measure: Injury
Sherwood, CS (2007)	Needleguard systems: an evaluation	2.2.4 Engineering controls	Q—Human health and social work activities WORK SETTING: 050 Health establishment (GB)	7 = contact with sharp or pointed materials or tools 015 = Needlestick Injuries Outcome measure: Risk or behavior
Silverwood, S (2006)	Reduction of musculoskeletal injuries in intensive care nurses using ceiling‐mounted patient lifts	2.2.4 Engineering controls	Q—Human health and social work activities WORK SETTING: 050 Health establishment (CA)	8 = overexertion of the musculoskeletal system 030 = overexertion injuries (dislocations, sprains and strains) Outcome measure: Injury
Smith, DA (1992)	Constant incidence rates of needle‐stick injury paradoxically suggest modest preventive effect of sharps disposal system	2.2.4 Engineering controls	Q—Human health and social work activities WORK SETTING: 050 Health establishment (US)	7 = contact with sharp or pointed materials or tools 015 = Needlestick Injuries Outcome measure: Injury
Sohn, S (2004)	Safety‐engineered device implementation: does it introduce bias in percutaneous injury reporting?	2.2.4 Engineering controls	Q—Human health and social work activities WORK SETTING: 050 Health establishment (US)	7 = contact with sharp or pointed materials or tools 015 = Needlestick Injuries Outcome measure: Injury
Stevens, L (2013)	Creating a culture of safety for safe patient handling	2.2.4 Engineering controls	Q—Human health and social work activities WORK SETTING: 050 Health establishment (US)	8 = overexertion of the musculoskeletal system 030 = overexertion injuries (dislocations, sprains and strains) Outcome measure: Injury
Wolfrum, J (1994)	A follow‐up evaluation to a needle‐free i.v. system	2.2.4 Engineering controls	Q—Human health and social work activities WORK SETTING: 050 Health establishment (US)	7 = contact with sharp or pointed materials or tools 015 = Needlestick Injuries Outcome measure: Injury
Wright, GD (1993)	Needle covers reduce needlestick injury	2.2.4 Engineering controls	Q—Human health and social work activities WORK SETTING: 050 Health establishment (AU)	7 = contact with sharp or pointed materials or tools 015 = Needlestick Injuries Outcome measure: Injury
Yassi, A (1995)	Efficacy and cost‐effectiveness of a needleless intravenous access system	2.2.4 Engineering controls	Q—Human health and social work activities WORK SETTING: 050 Health establishment (CA)	7 = contact with sharp or pointed materials or tools 015 = Needlestick Injuries Outcome measure: Injury
McGrail, MP (1995)	A comprehensive initiative to manage the incidence and cost of occupational injury and illness. Report of an outcomes analysis	2.2.5 Administrative controls	Q—Human health and social work activities WORK SETTING: 050 Health establishment (US)	1 = All types of accident 888 = All types of injuries Outcome measure: Injury
Reimer, DS (1994)	A novel approach to preemployment worker fitness evaluations in a material‐handling industry	2.2.5 Administrative controls	H—Transporting and storage WORK SETTING: 010 Industrial site (US)	1 = All types of accident 888 = All types of injuries Outcome measure: Injury
Thorson, J (1999)	Lyckad profylax i Sverige mot traktorolyckor	2.2.5 Administrative controls	A—Agriculture, forestry and fishing WORK SETTING: 030 Farming and forestry (SE)	1 = All types of accident 888 = All types of injuries Outcome measure: Injury
Altayeb, S (1992)	Efficacy of drug testing programs implemented by contractors	2.2.7 Enforcement of laws and regulations	F—Construction WORK SETTING: 020 Construction site (US)	1 = All types of accident 888 = All types of injuries Outcome measure: Injury
**3.0.0 Multifaceted interventions**				
Williams, Q Jr (2010)	The impact of a peer‐led participatory health and safety training program for Latino day laborers in construction	3.1 Multifaceted at the individual level	A—Agriculture, forestry and fishing WORK SETTING: 030 Farming and forestry (US)	1 = All types of accident 015 = Needlestick Injuries Outcome measure: Risk or behavior
Bejan, A (2015)	2‐year follow‐up of the Collision Auto Repair Safety Study (CARSS)	3.2 Multifaceted at the group or organizational level	G—Wholesale and retail trade WORK SETTING: 010 Industrial site (US)	1 = All types of accident 888 = All types of injuries Outcome measure: Risk or behavior
Dawson, EB (2010)	Effect of the Workforce Initiatives Safe Handling Minimal Lift Program on patient care provider injuries, attributable costs and satisfaction	3.2 Multifaceted at the group or organizational level	Q—Human health and social work activities WORK SETTING: 050 Health establishment (US)	8 = overexertion of the musculoskeletal system 030 = overexertion injuries (dislocations, sprains and strains) Outcome measure: Injury
Parker, DL (2015)	The Collision Auto Repair Safety Study (CARSS): a health and safety intervention	3.2 Multifaceted at the group or organizational level	G—Wholesale and retail trade WORK SETTING: 010 Industrial site (US)	1 = All types of accident 888 = All types of injuries Outcome measure: Risk or behavior
Roudot‐Thoraval, F (1999)	Costs and benefits of measures to prevent needlestick injuries in a university hospital	3.2 Multifaceted at the group or organizational level	Q—Human health and social work activities WORK SETTING: 050 Health establishment (FR)	7 = contact with sharp or pointed materials or tools 030 = overexertion injuries (dislocations, sprains and strains) Outcome measure: Injury
Shouman, AE (2002)	Accident prevention program in a glass factory in Shoubra El Khema district	3.2 Multifaceted at the group or organizational level	C—Manufacturing WORK SETTING: 010 Industrial site (EG)	1 = All types of accident 888 = All types of injuries Outcome measure: Injury
Springer, PJ (2009)	Preventing employee injury. Implementation of a lift team	3.2 Multifaceted at the group or organizational level	Q—Human health and social work activities WORK SETTING: 050 Health establishment (US)	8 = overexertion of the musculoskeletal system 030 = overexertion injuries (dislocations, sprains and strains) Outcome measure: Injury
Adams, D (2006)	Impact of safety needle devices on occupationally acquired needlestick injuries: a 4‐year prospective study	3.3 Multifaceted across individual and organizational levels	Q—Human health and social work activities WORK SETTING: 050 Health establishment (GB)	7 = contact with sharp or pointed materials or tools 888 = All types of injuries Outcome measure: Injury
Allen, D (2013)	Staying safe: Re‐examining workplace violence in acute psychiatric settings	3.3 Multifaceted across individual and organizational levels	Q—Human health and social work activities WORK SETTING: 050 Health establishment (US)	1 = All types of accident 888 = All types of injuries Outcome measure: Injury
Bell, JL (2006)	Evaluating the effectiveness of a logger safety training program	3.3 Multifaceted across individual and organizational levels	A—Agriculture, forestry and fishing WORK SETTING: 030 Farming and forestry (NORTH AMERICA)	1 = All types of accident 888 = All types of injuries Outcome measure: Injury
Bhimani, R (2014)	Prevention of work‐related musculoskeletal injuries in rehabilitation nursing	3.3 Multifaceted across individual and organizational levels	Q—Human health and social work activities WORK SETTING: 050 Health establishment (US)	8 = overexertion of the musculoskeletal system 030 = overexertion injuries (dislocations, sprains and strains) Outcome measure: Injury
Boynton, T (2008)	Participatory ergonomics intervention in a sterile processing center: a case study	3.3 Multifaceted across individual and organizational levels	Q—Human health and social work activities WORK SETTING: 050 Health establishment (US)	8 = overexertion of the musculoskeletal system 888 = All types of injuries Outcome measure: Injury
Brophy, MO (2001)	Reducing incidence of low‐back injuries reduces cost	3.3 Multifaceted across individual and organizational levels	Q—Human health and social work activities WORK SETTING: 050 Health establishment (US)	8 = overexertion of the musculoskeletal system 030 = overexertion injuries (dislocations, sprains and strains) Outcome measure: Injury
Charney, W (2006)	Zero lift programs in small rural hospitals in Washington state: reducing back injuries among health care workers	3.3 Multifaceted across individual and organizational levels	Q—Human health and social work activities WORK SETTING: 050 Health establishment (US)	8 = overexertion of the musculoskeletal system 030 = overexertion injuries (dislocations, sprains and strains) Outcome measure: Injury
Collins, JW (2004)	An evaluation of a "best practices" musculoskeletal injury prevention program in nursing homes	3.3 Multifaceted across individual and organizational levels	Q—Human health and social work activities WORK SETTING: 050 Health establishment (US)	8 = overexertion of the musculoskeletal system 030 = overexertion injuries (dislocations, sprains and strains) Outcome measure: Injury
Darragh, AR (2004)	Effectiveness of the Home Safe Pilot Program in reducing injury rates among residential construction workers, 1994–1998	3.3 Multifaceted across individual and organizational levels	F—Construction WORK SETTING: 020 Construction site (US)	1 = All types of accident 888 = All types of injuries Outcome measure: Injury
Evanoff, B (2003)	Reduction in injury rates in nursing personnel through introduction of mechanical lifts in the workplace	3.3 Multifaceted across individual and organizational levels	Q—Human health and social work activities WORK SETTING: 050 Health establishment (US)	8 = overexertion of the musculoskeletal system 030 = overexertion injuries (dislocations, sprains and strains) Outcome measure: Injury
Garb, JR (1995)	Reducing employee back injuries in the perioperative setting	3.3 Multifaceted across individual and organizational levels	Q—Human health and social work activities WORK SETTING: 050 Health establishment (US)	8 = overexertion of the musculoskeletal system 030 = overexertion injuries (dislocations, sprains and strains) Outcome measure: Injury
Haiduven, DJ (1995)	Percutaneous injury analysis: consistent categorization, effective reduction methods, and future strategies	3.3 Multifaceted across individual and organizational levels	Q—Human health and social work activities WORK SETTING: 050 Health establishment (US)	7 = contact with sharp or pointed materials or tools 015 = Needlestick Injuries Outcome measure: Injury
Haiduven, DJ (1992)	A 5‐year study of needlestick injuries: significant reduction associated with communication, education, and convenient placement of sharps containers	3.3 Multifaceted across individual and organizational levels	Q—Human health and social work activities WORK SETTING: 050 Health establishment (US)	7 = contact with sharp or pointed materials or tools 015 = Needlestick Injuries Outcome measure: Injury
Kutash, M (2009)	The lift team's importance to a successful safe patient handling program	3.3 Multifaceted across individual and organizational levels	Q—Human health and social work activities WORK SETTING: 050 Health establishment (US)	8 = overexertion of the musculoskeletal system 030 = overexertion injuries (dislocations, sprains and strains) Outcome measure: Injury
Landers, M (2004)	Effects of a work injury prevention program for housekeeping in the hotel industry	3.3 Multifaceted across individual and organizational levels	I—Accommodation and food service activities WORK SETTING: 040 tertiary activity area (such as office, teaching establishment, restaurant etc.), incl retail (US)	1 = All types of accident 888 = All types of injuries Outcome measure: Injury
Linnemann, CC Jr (1991)	Effect of educational programs, rigid sharps containers, and universal precautions on reported needlestick injuries in healthcare workers	3.3 Multifaceted across individual and organizational levels	Q—Human health and social work activities WORK SETTING: 050 Health establishment (US)	7 = contact with sharp or pointed materials or tools 015 = Needlestick Injuries Outcome measure: Injury
Lynch, RM (2000)	Short‐term efficacy of back injury intervention project for patient care providers at one hospital	3.3 Multifaceted across individual and organizational levels	Q—Human health and social work activities WORK SETTING: 050 Health establishment (US)	8 = overexertion of the musculoskeletal system 030 = overexertion injuries (dislocations, sprains and strains) Outcome measure: Injury
Mendelson, MH (2003)	Evaluation of a safety resheathable winged steel needle for prevention of percutaneous injuries associated with intravascular‐access procedures among healthcare workers	3.3 Multifaceted across individual and organizational levels	Q—Human health and social work activities WORK SETTING: 050 Health establishment (US)	7 = contact with sharp or pointed materials or tools 015 = Needlestick Injuries Outcome measure: Injury
Nelson, A (2006)	Development and evaluation of a multifaceted ergonomics program to prevent injuries associated with patient handling tasks	3.3 Multifaceted across individual and organizational levels	Q—Human health and social work activities WORK SETTING: 050 Health establishment (US)	8 = overexertion of the musculoskeletal system 030 = overexertion injuries (dislocations, sprains and strains) Outcome measure: Injury
Nielsen, KJ (2014)	Improving safety culture through the health and safety organization: A case study	3.3 Multifaceted across individual and organizational levels	C—Manufacturing WORK SETTING: 010 Industrial site (DK)	1 = All types of accident 888 = All types of injuries Outcome measure: Injury
Sellick, JA (1991)	Influence of an educational program and mechanical opening needle disposal boxes on occupational needlestick injuries	3.3 Multifaceted across individual and organizational levels	Q—Human health and social work activities WORK SETTING: 050 Health establishment (US)	10 = Other specific types of accident 015 = Needlestick Injuries Outcome measure: Injury
Sohn, S (2004)	Effect of implementing safety‐engineered devices on percutaneous injury epidemiology	3.3 Multifaceted across individual and organizational levels	Q—Human health and social work activities WORK SETTING: 050 Health establishment (US)	6 = Trapped, crushed, struck by equipment or objects 015 = Needlestick Injuries Outcome measure: Injury
Theis, JL (2013)	Long‐term effects of safe patient handling program on staff injuries	3.3 Multifaceted across individual and organizational levels	Q—Human health and social work activities WORK SETTING: 050 Health establishment (US)	8 = overexertion of the musculoskeletal system 030 = overexertion injuries (dislocations, sprains and strains) Outcome measure: Injury
Wardell, H (2007)	Reduction of injuries associated with patient handling	3.3 Multifaceted across individual and organizational levels	Q—Human health and social work activities WORK SETTING: 050 Health establishment (US)	8 = overexertion of the musculoskeletal system 030 = overexertion injuries (dislocations, sprains and strains) Outcome measure: Risk or behavior
Whitby, M (1991)	Needlestick injury: impact of a recapping device and an associated education program	3.3 Multifaceted across individual and organizational levels	Q—Human health and social work activities WORK SETTING: 050 Health establishment (AU)	7 = contact with sharp or pointed materials or tools 015 = Needlestick Injuries Outcome measure: Injury
Yao, W‐X (2013)	Occupational safety training and education for needlestick injuries among nursing students in China: intervention study	3.3 Multifaceted across individual and organizational levels	Q—Human health and social work activities WORK SETTING: 050 Health establishment (ASIA)	7 = contact with sharp or pointed materials or tools 015 = Needlestick Injuries Outcome measure: Injury
Zafar, A (2009)	Impact of infection control activities on the rate of needle stick injuries at a tertiary care hospital of Pakistan over a period of 6 years: an observational study	3.3 Multifaceted across individual and organizational levels	Q—Human health and social work activities WORK SETTING: 050 Health establishment (ASIA)	7 = contact with sharp or pointed materials or tools 015 = Needlestick Injuries Outcome measure: Injury
Zawilla, NH (2013)	Sharps injuries among health care workers in Cairo University Hospitals	3.3 Multifaceted across individual and organizational levels	Q—Human health and social work activities WORK SETTING: 050 Health establishment (AFRICA)	7 = contact with sharp or pointed materials or tools 015 = Needlestick Injuries Outcome measure: Injury
Hale, AR (2010)	Evaluating safety management and culture interventions to improve safety: Effective intervention strategies	3.9 Multifaceted safety interventions not listed above	??? WORK SETTING:??? (EUROPE)	1 = All types of accident 888 = All types of injuries Outcome measure: Risk or behavior
**Type of intervention not reported or unclear**				
Alamgir, H (2011)	Peer coaching and mentoring: a new model of educational intervention for safe patient handling in health care	0.0.0 Type of intervention not reported or unclear	Q—Human health and social work activities WORK SETTING: 050 Health establishment (CA)	8 = overexertion of the musculoskeletal system 030 = overexertion injuries (dislocations, sprains and strains) Outcome measure: Risk or behavior
Guimarães, LB (2012)	Cost‐benefit analysis of a socio‐technical intervention in a Brazilian footwear company	0.0.0 Type of intervention not reported or unclear	C—Manufacturing WORK SETTING: 010 Industrial site (BR)	1 = All types of accident 888 = All types of injuries Outcome measure: Injury
Mobasherizadeh, S (2005)	Intervention study of needle stick injury in Iran	0.0.0 Type of intervention not reported or unclear	Q—Human health and social work activities WORK SETTING: 050 Health establishment (IR)	7 = contact with sharp or pointed materials or tools 888 = All types of injuries Outcome measure: Injury
Moens, G (2004)	Analyzing and interpreting routinely collected data on sharps injuries in assessing preventative actions	0.0.0 Type of intervention not reported or unclear	Q—Human health and social work activities WORK SETTING: 050 Health establishment (BE)	7 = contact with sharp or pointed materials or tools 015 = Needlestick Injuries Outcome measure: Injury
Pekkarinen, A (1992)	Prevention of accidents in reindeer herding work	0.0.0 Type of intervention not reported or unclear	A—Agriculture, forestry and fishing WORK SETTING: 030 Farming and forestry (FI)	1 = All types of accident 888 = All types of injuries Outcome measure: Injury
Powell‐Cope, G (2014)	Effects of a national safe patient handling program on nursing injury incidence rates	0.0.0 Type of intervention not reported or unclear	Q—Human health and social work activities WORK SETTING: 050 Health establishment (US)	8 = overexertion of the musculoskeletal system 030 = overexertion injuries (dislocations, sprains and strains) Outcome measure: Injury
Viljoen, D (2010)	Improved injury management at an Australian aluminium smelter	0.0.0 Type of intervention not reported or unclear	C—Manufacturing WORK SETTING: 010 Industrial site (AU)	1 = All types of accident 888 = All types of injuries Outcome measure: Injury
Yassin, AS (2004)	The effectiveness of the revised scaffold safety standard in the construction industry	0.0.0 Type of intervention not reported or unclear	F—Construction WORK SETTING: 020 Construction site (US)	Outcome measure:
Calé, MH (2012)	Improving safety behavior and accident rates of professional drivers: the Dead Sea project	Type of intervention not reported or unclear	H—Transporting and storage WORK SETTING: 060 Public area (IL)	1 = All types of accident 888 = All types of injuries Outcome measure: Risk or behavior
Kaskutas, V (2013)	Fall prevention and safety communication training for foremen: report of a pilot project designed to improve residential construction safety	Type of intervention not reported or unclear	F—Construction WORK SETTING: 020 Construction site (NORTH AMERICA)	1 = All types of accident 888 = All types of injuries Outcome measure: Risk or behavior

Table 16 Nature of included before and after studies (BA) by main type of safety intervention and specific type of safety intervention.

## 11 DATA AND ANALYSIS

### 11.1 Forest plots by type of safety intervention

#### 11.1.1 Forest plots for RCT and CBA study designs, by type of safety intervention (comparison)

We have included meta‐analyses where applicable (see methods Section 3). We have evaluated each study for risk of bias (4.3) and additional quality criteria, and included this in the Forest plot tables. In the Table 17, is shown the list of risk of bias dimensions included and the three quality criteria used for the assessment of the RoB/quality of the studies.

**Table 17:** Risk of bias dimensions (A–K) referring to legends in comparison A1–A30

Risk of bias	Legend
A	Random sequence generation (selection bias)
B	Allocation concealment (selection bias)
C	Equivalent groups
D	Blinding of participants and personnel (performance bias)
E	Blinding of outcome assessment (detection bias)
F	Statistical analysis (Detection Bias)
G	Incomplete outcome data (attrition bias)
H	Selective reporting (reporting bias)
I	Intervention fidelity
J	Intervention rationale (why the intervention should work)
K	Other bias

We have used a random effects analysis when synthesizing average effect sizes (see Section 3.4).

Comparison A1: 1.1.1 Safety Campaign versus usual control conditions, at short‐term follow‐up (FU), for outcome: 1.1 Risk and Behavior.


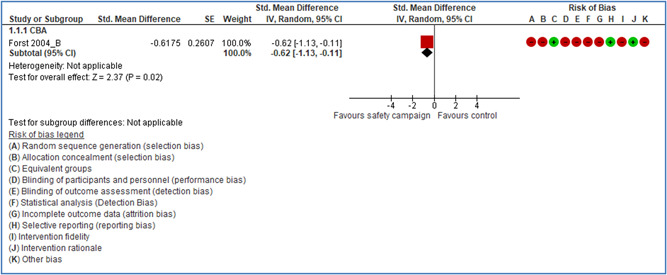


Comparison A2: 1.1.1 Safety Campaign versus usual control conditions, at medium‐term FU, for outcome: 2.1 Injuries.


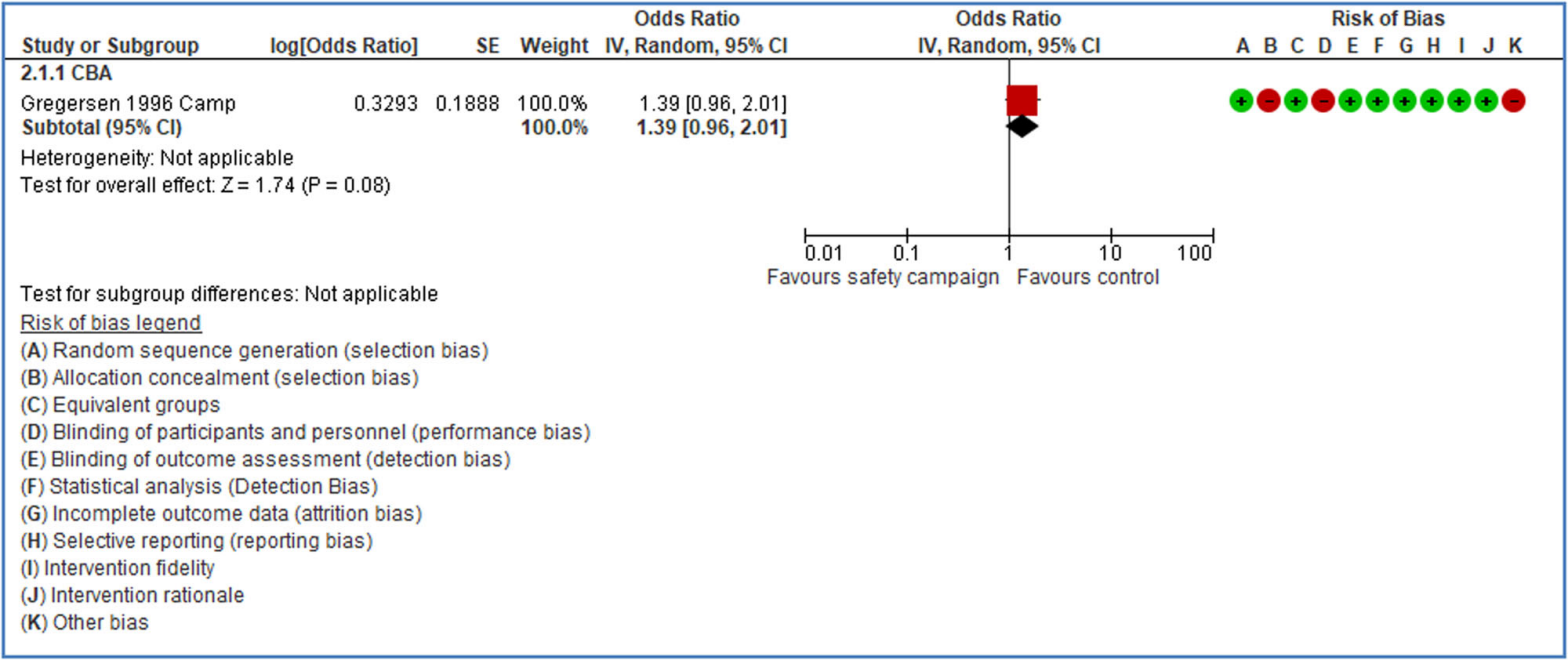


Comparison A3: 1.1.2 Counseling approaches versus usual control conditions, at short‐term FU, for outcome: 3.1 injuries


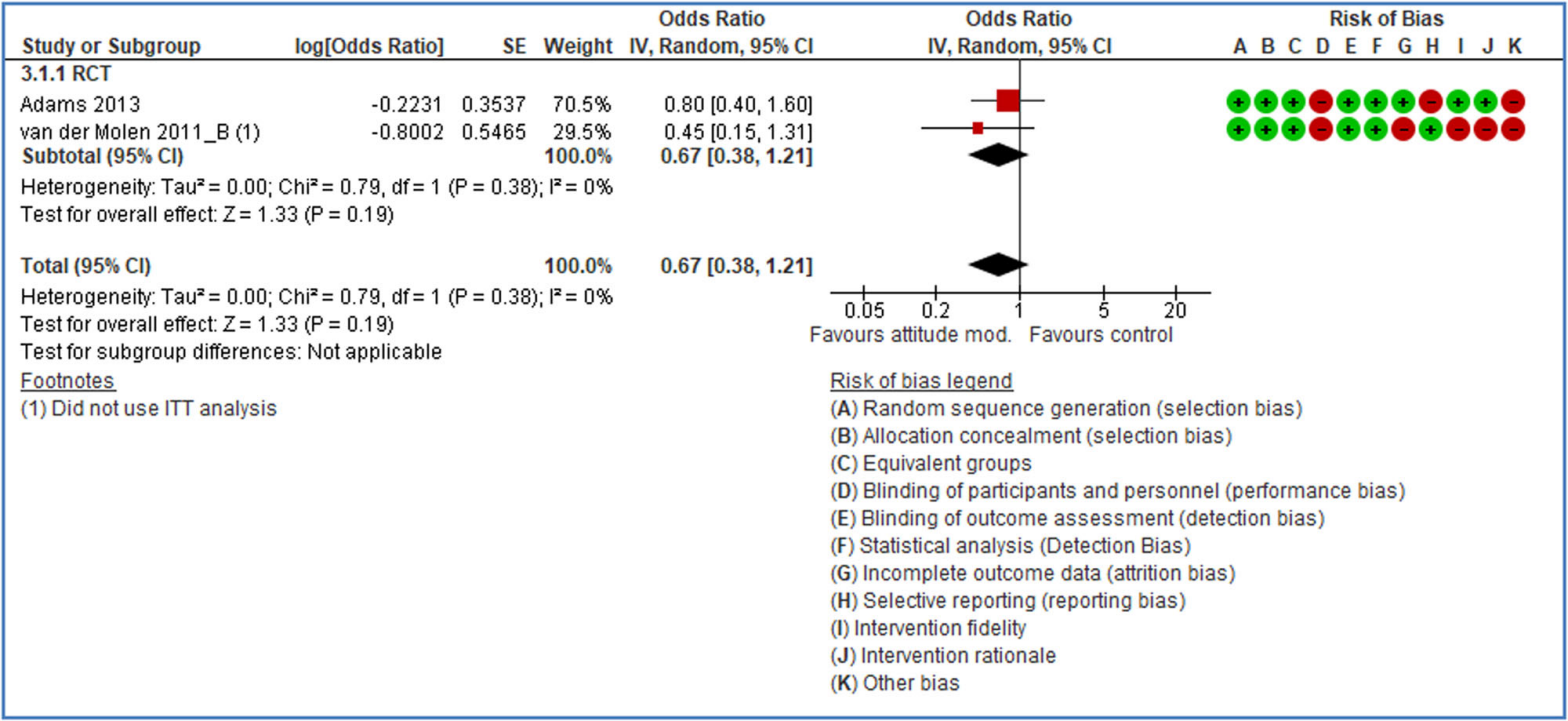


Comparison A4: 1.1.2 Counseling approaches versus usual control conditions, at medium‐term FU, for outcome: 4.1 injuries


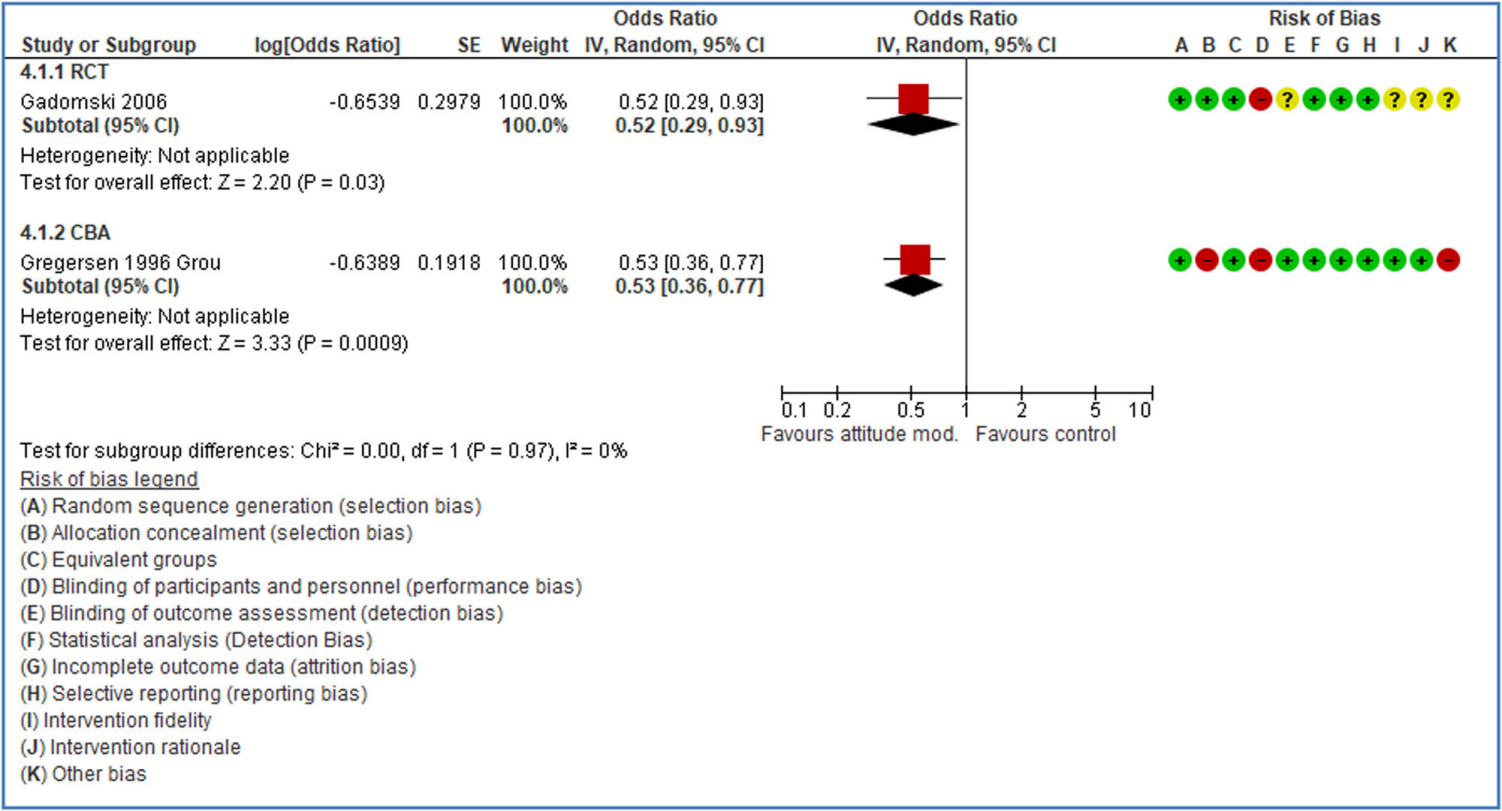


Comparison A5: 1.1.3 Teaching and education versus usual control conditions, at short‐term FU, for outcome: 5.1 injuries


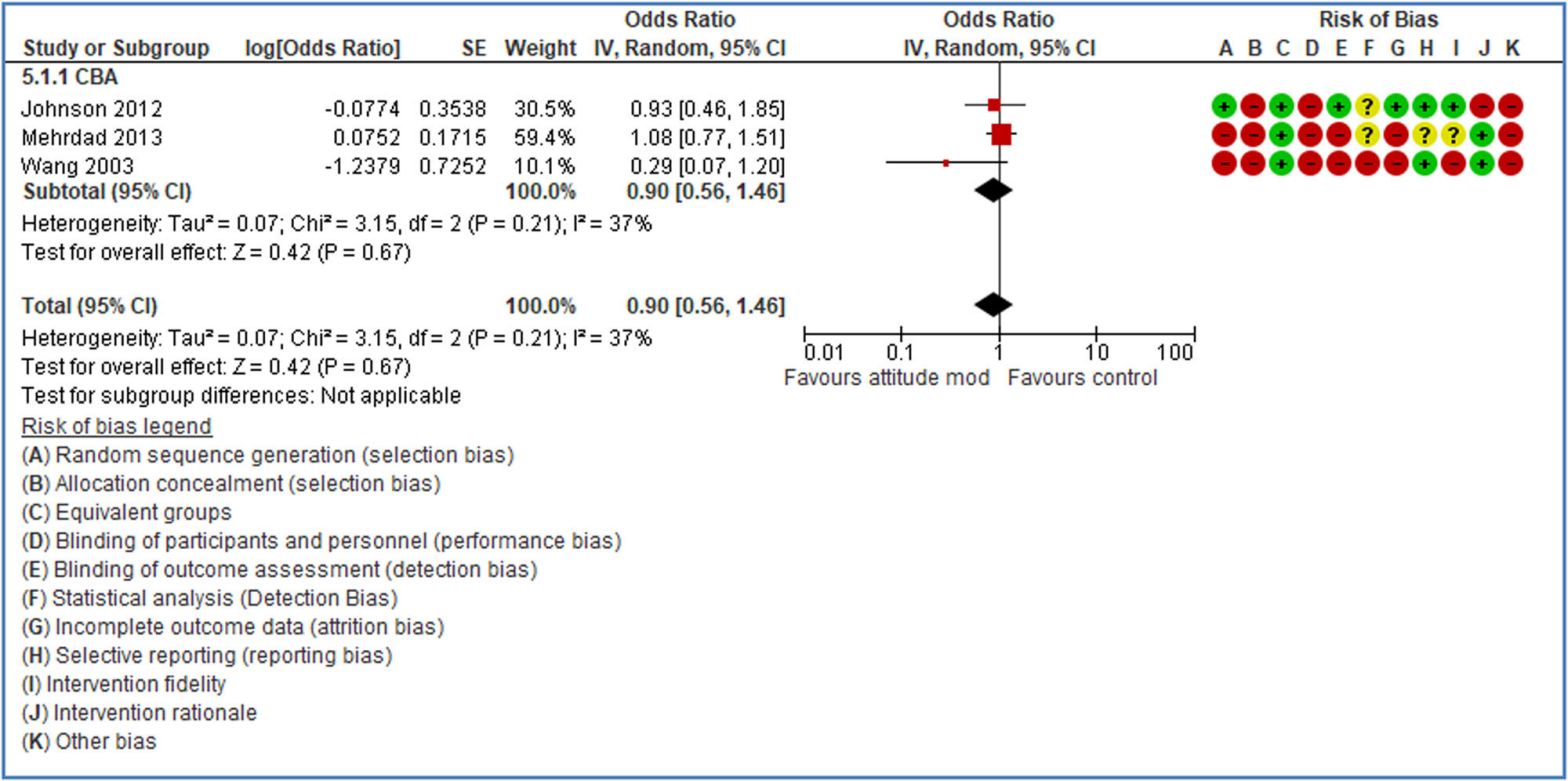


Comparison: A6 1.2.2 Safety Training versus usual control conditions, at short‐term FU, for outcome: 6.1 Injuries.


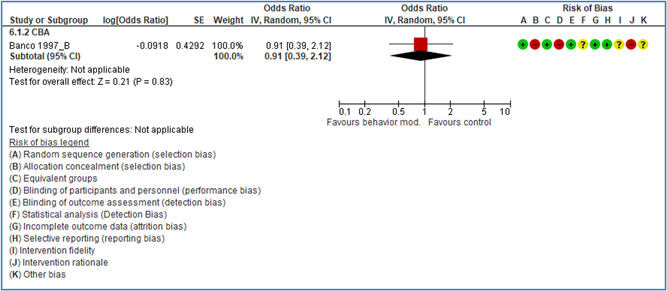


Comparison A7: 1.2.2 Safety Training versus usual control conditions, at medium‐term FU, for outcome: 7.1 Injuries.


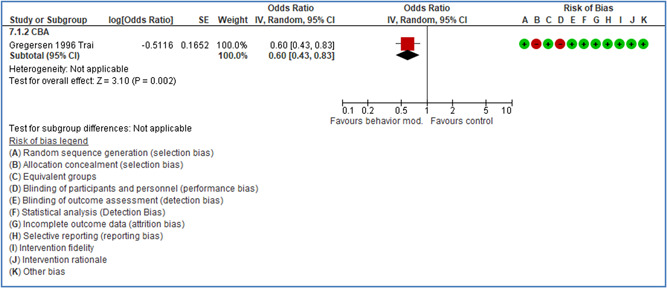


Comparison A8: 1.2.4 Individual feedback versus usual control conditions, at short‐term FU, for outcome: 8.1 Injuries.


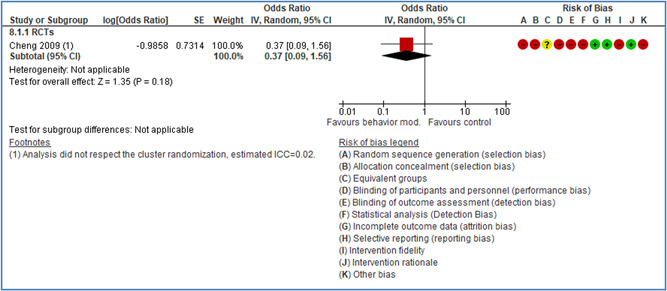


Comparison A8: 1.2.4 Individual feedback versus usual control conditions, at short‐term FU, for outcome: 8.2 Risk and Behavior.


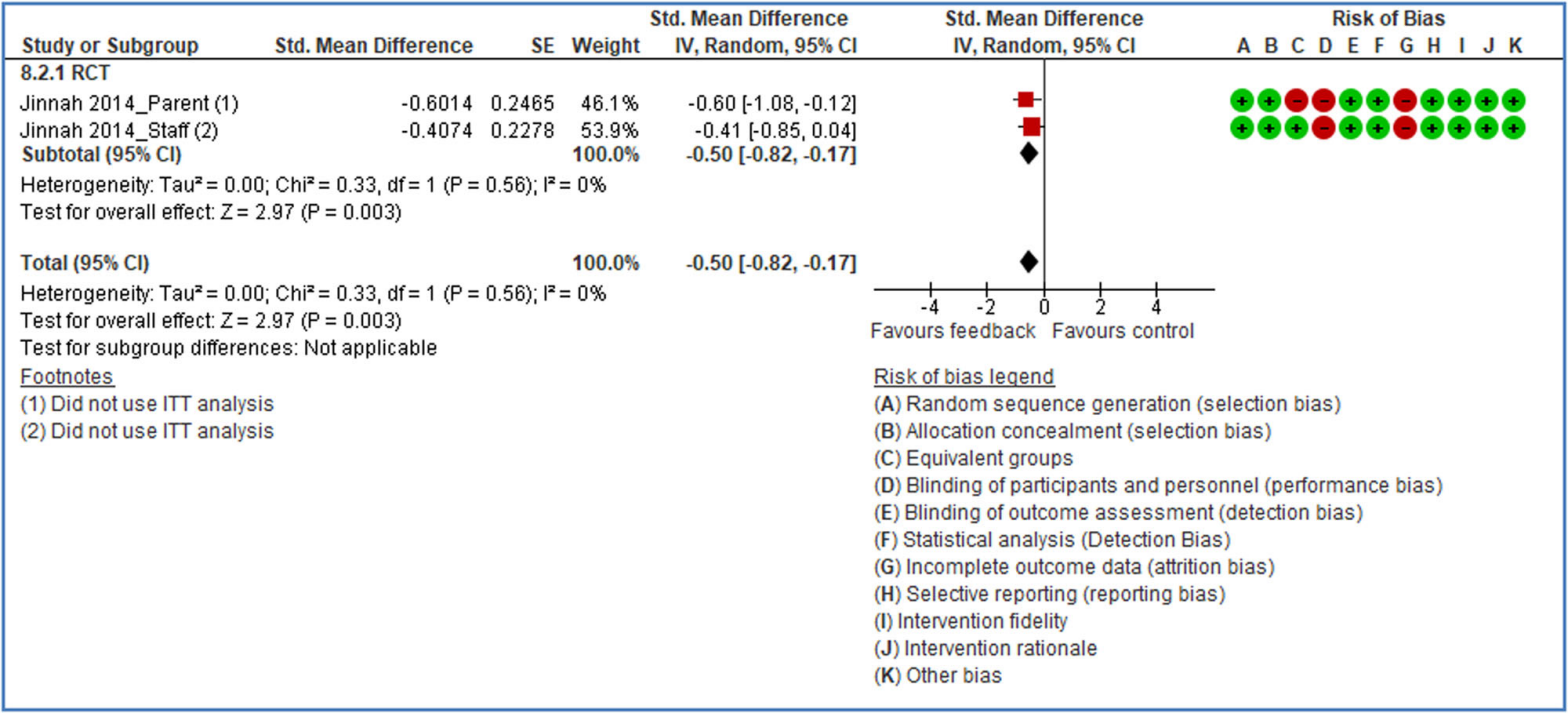


Comparison A9: 1.2.4 Individual feedback versus usual control conditions, at long‐term FU, for outcome: 9.1 Injuries.


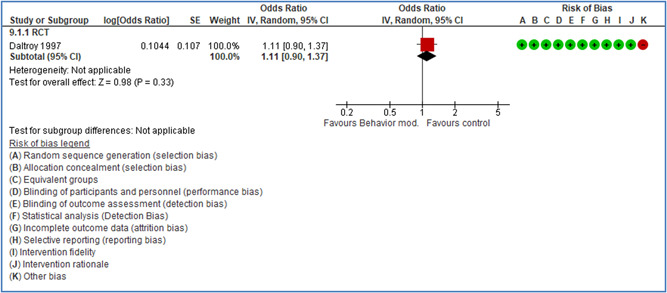


Comparison A10: 1.2.4 Individual feedback versus usual control conditions, at unknown FU, for outcome: 10.1 Injuries.


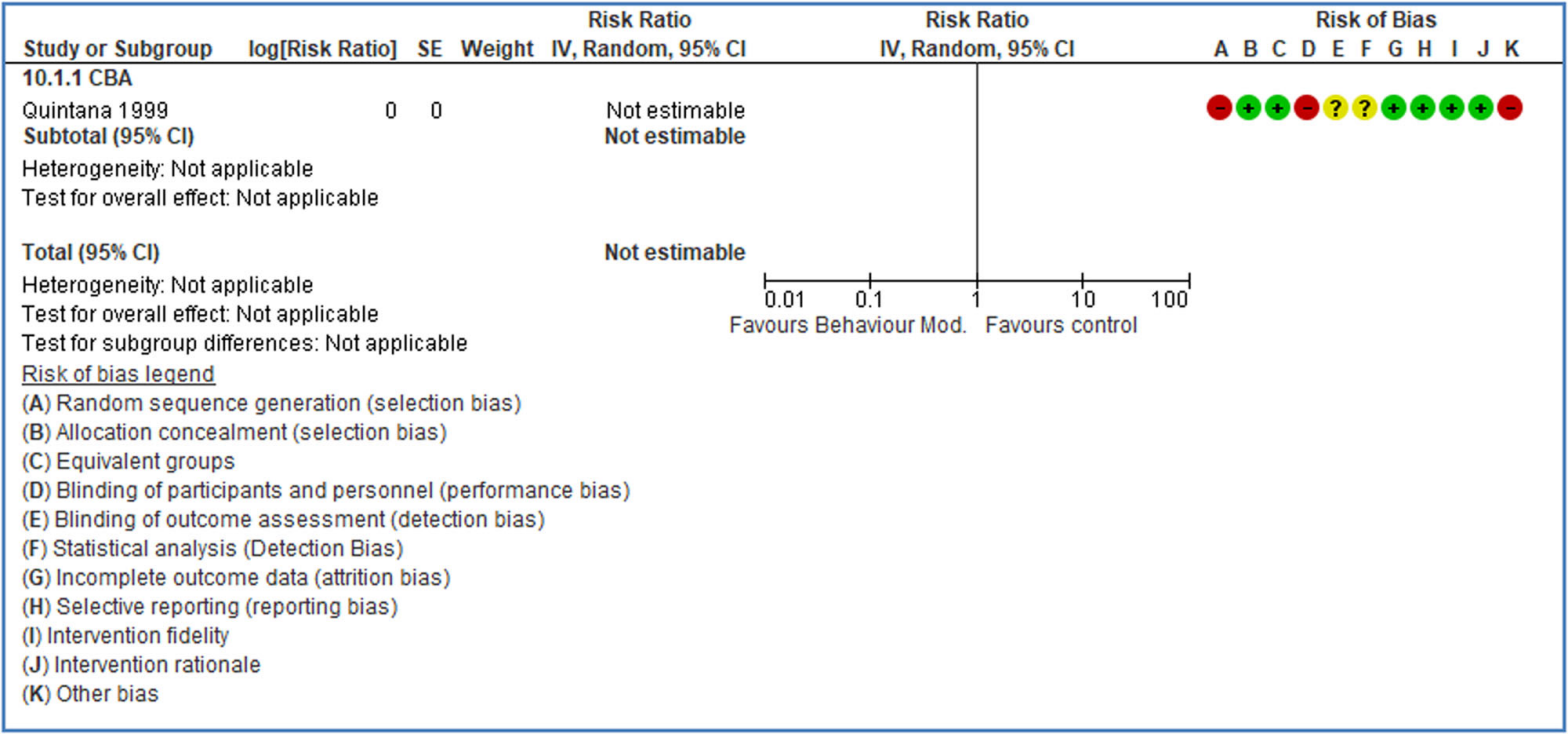


Comparison A11: Individual physical training versus usual control conditions, at short‐term FU, for outcome: 11.1 Injuries.


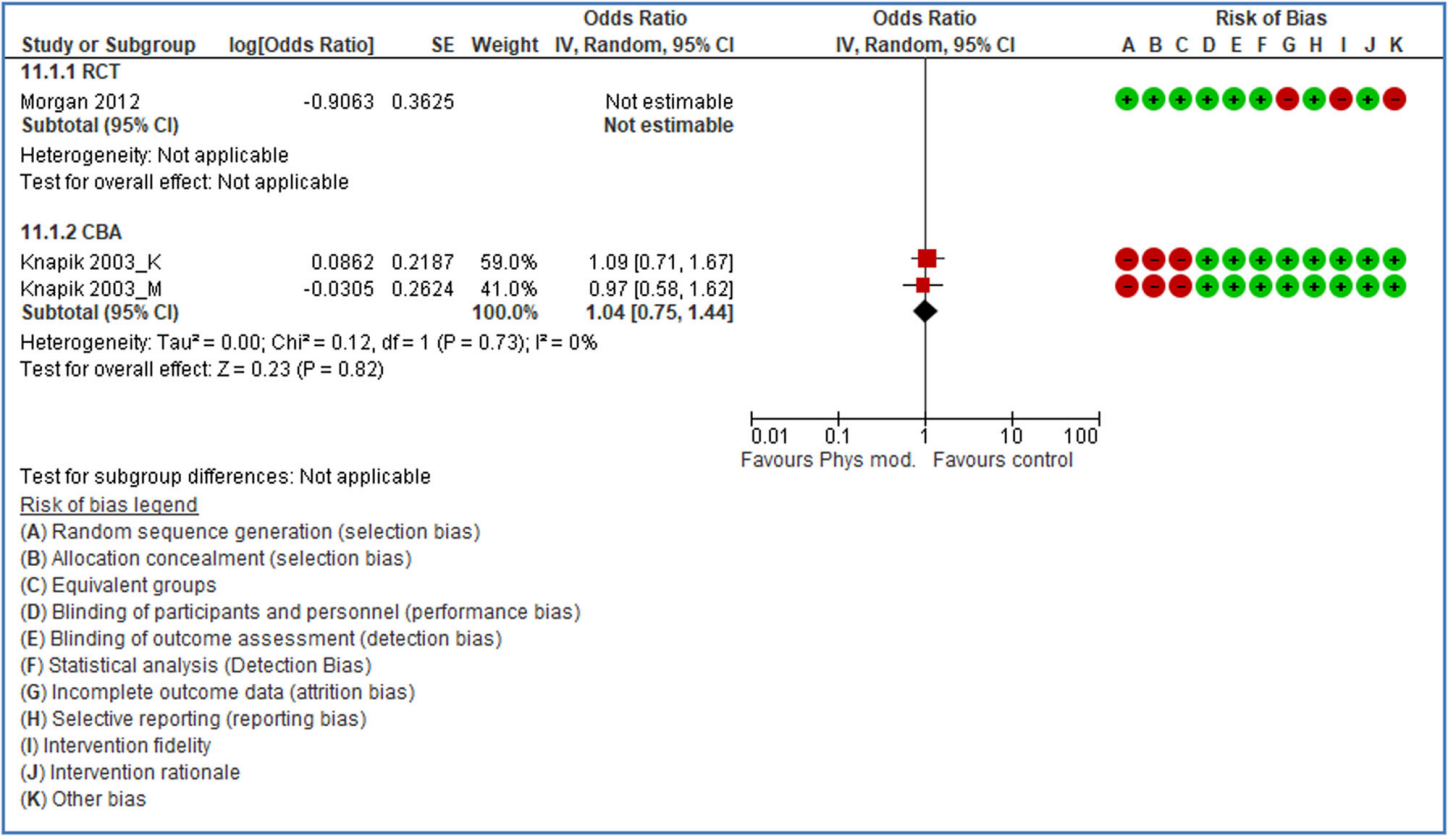


Comparison A12: Individual physical training versus usual control conditions, at medium‐term FU, for outcome: 12.1 Injuries.


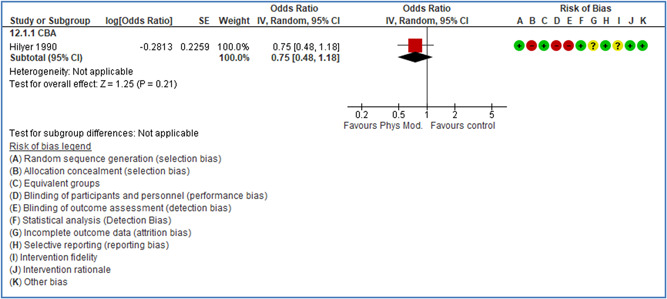


Comparison A13: 2.1.5 safety feedback (Organizational) versus usual control conditions, at short‐term FU, for outcome: 13.1 Injuries.


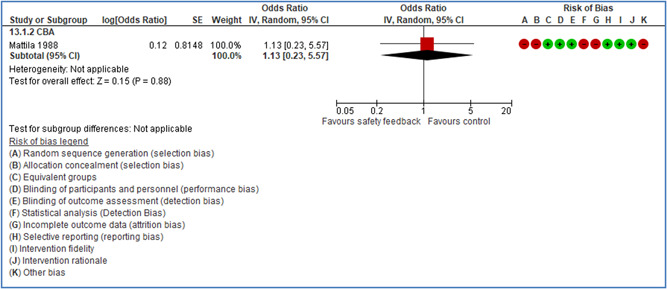


Comparison A13: 2.1.5 safety feedback (Organizational) versus usual control conditions, at short‐term FU, for outcome: 13.2 Risk and Behavior.


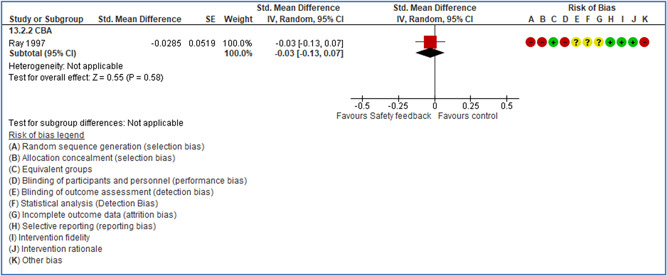


Comparison A14: 2.1.7 Leadership based safety interventions versus usual control conditions, at short‐term FU, for outcome: 14.1 Injuries.


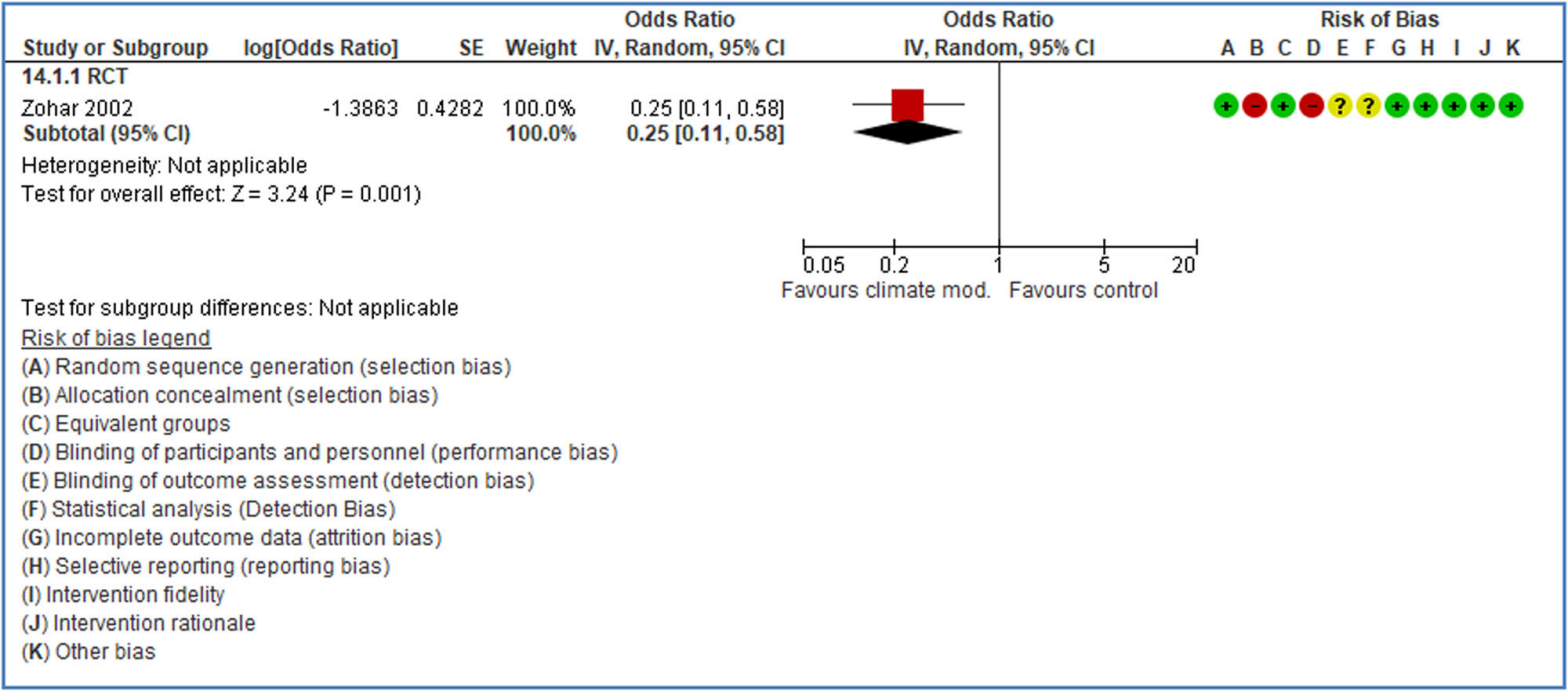


Comparison A14: 2.1.7 Leadership based safety interventions, versus usual control conditions, at short‐term FU, for outcome: 14.2 Risk and Behavior.


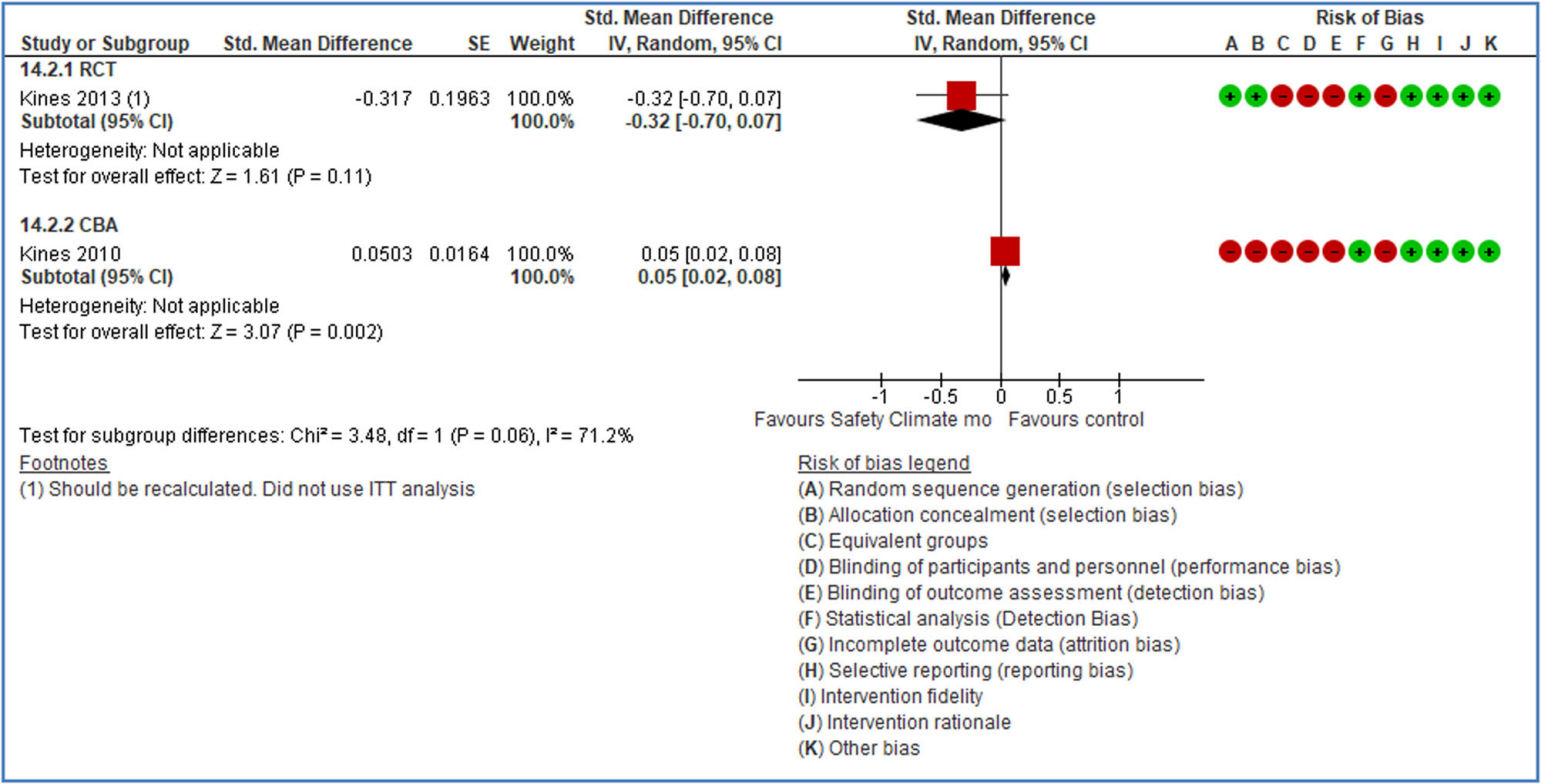


Comparison A15: Enforcement/compliance versus no intervention, at short‐term FU, for outcome: 15.1 Injuries.


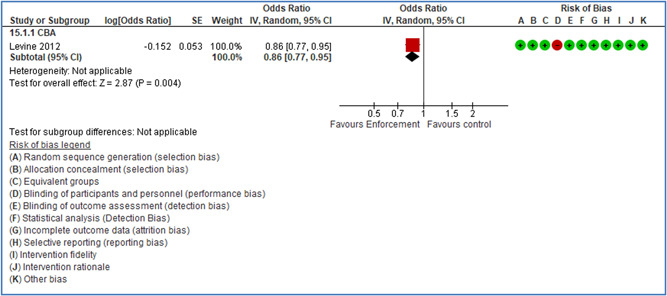


Comparison: A 16 Enforcement/compliance versus no intervention, at medium‐term FU, for outcome: 16.1 Injuries.


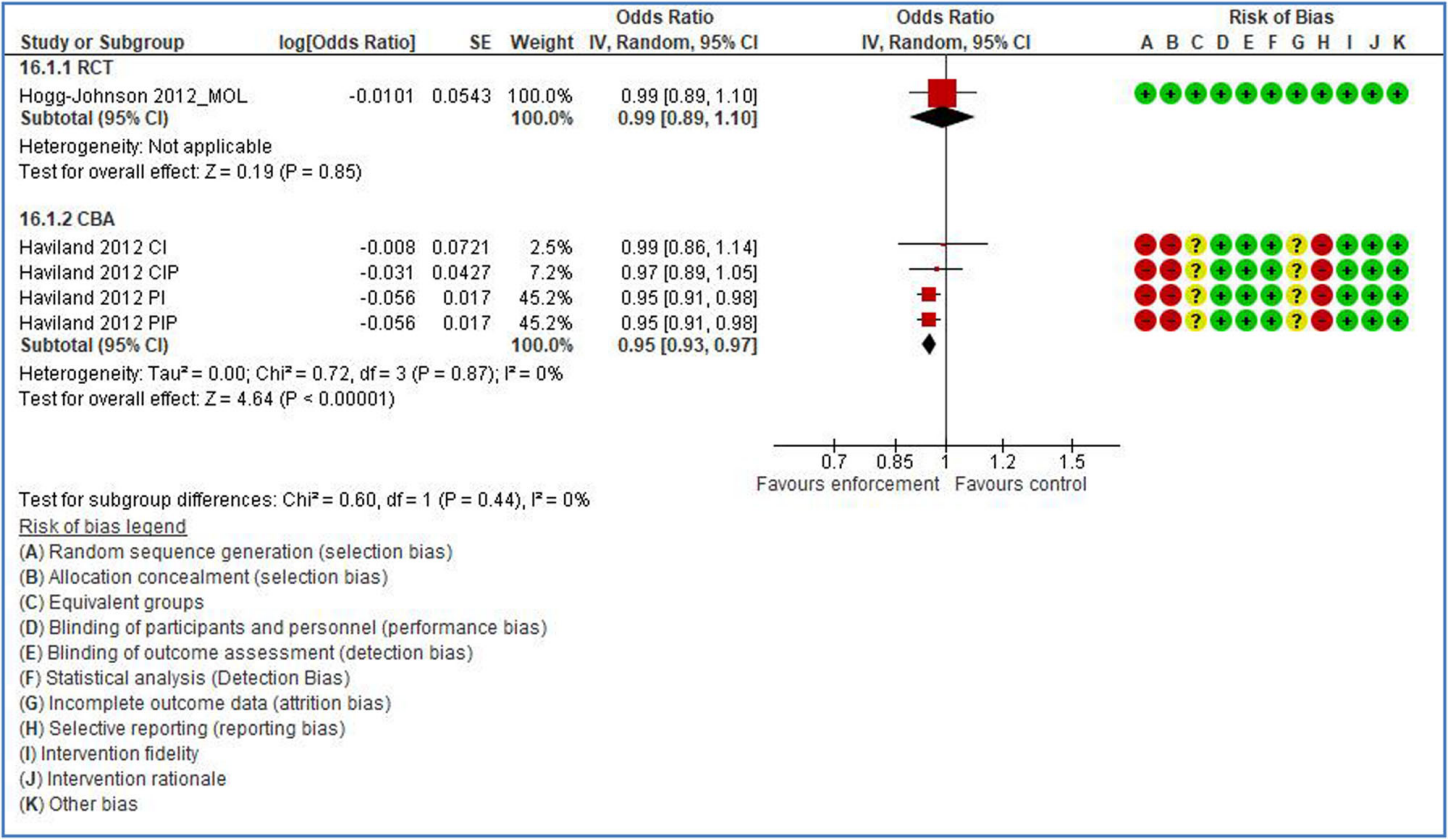


Comparison A17: Enforcement and compliance versus no interventions, at long‐term FU, for outcome: 17.1 Injuries.


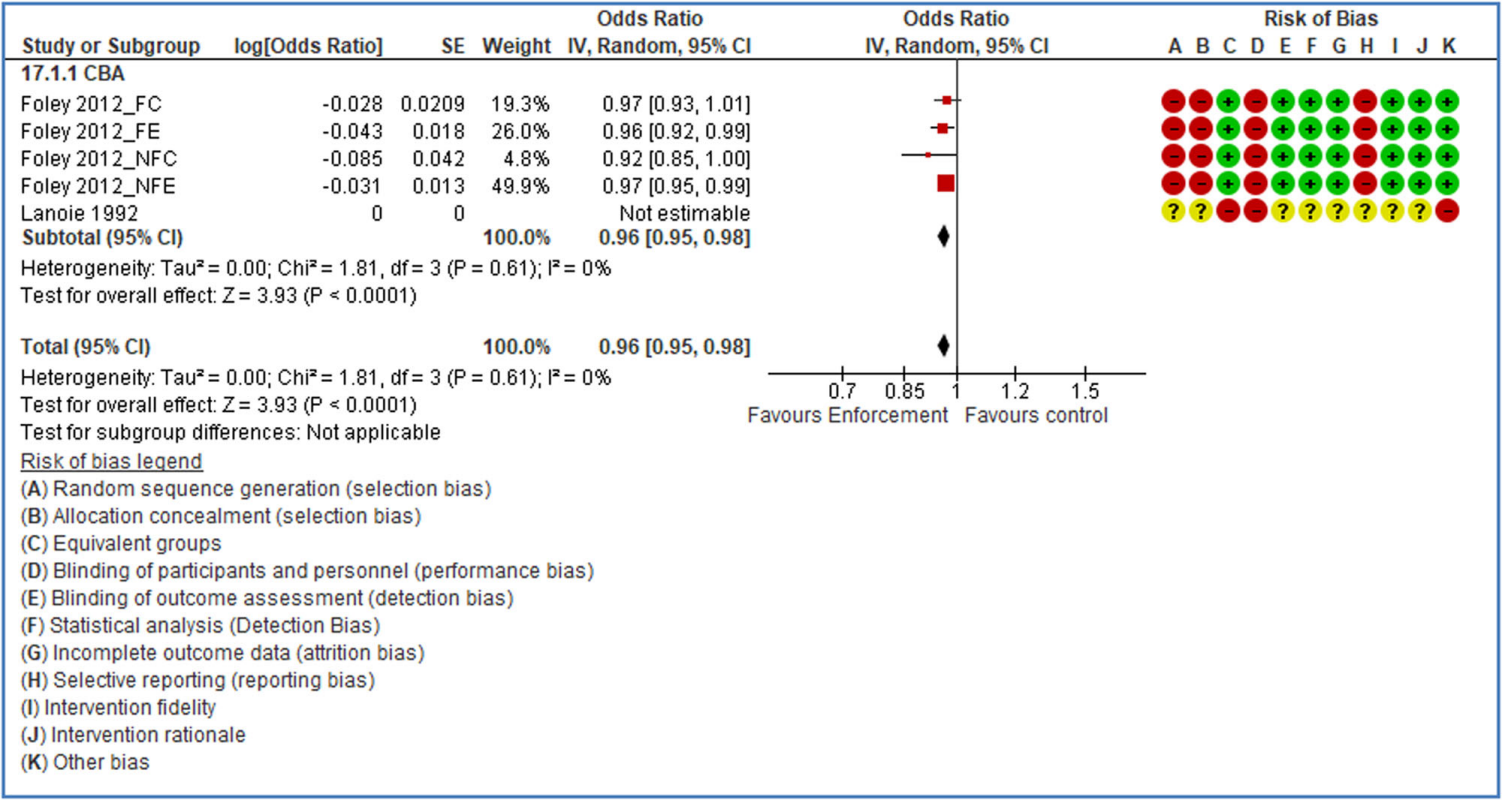


Comparison A18: Enforcement with penalty versus no intervention, at medium‐term FU, for outcome: 18.1 Injuries.


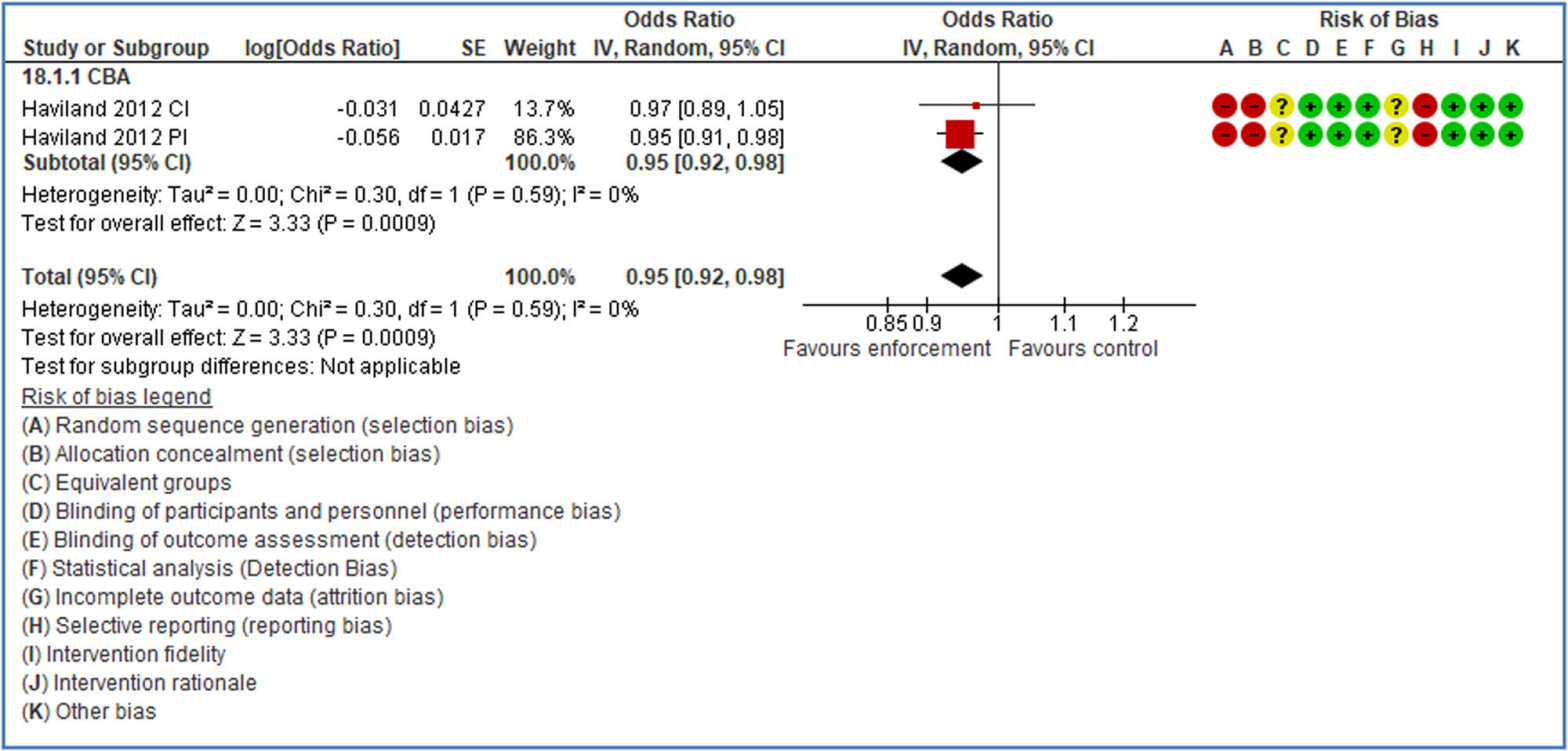


Comparison A19: Economic incentives versus usual control conditions, at medium‐term FU, for outcome: 19.1 Injuries.


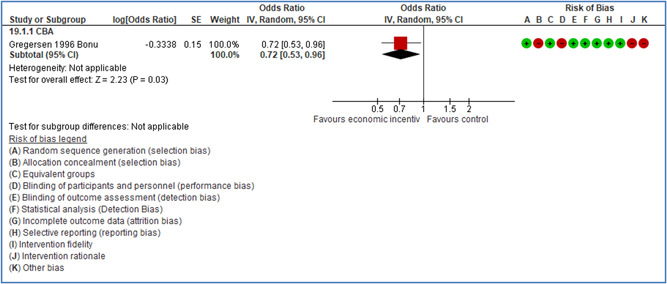


Comparison A20: Soft regulation versus usual control conditions, at medium‐term FU, for outcome: 20.1 Injuries.


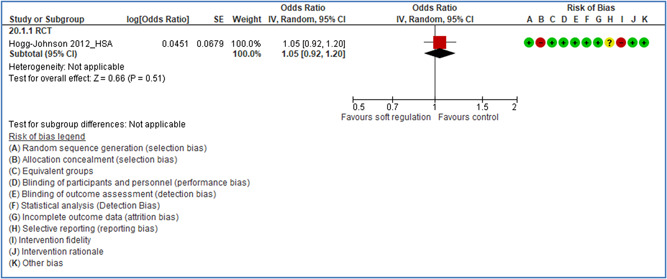


Comparison A21: Engineering control versus usual control conditions, at Posttest FU, for outcome: 21.1 Injuries


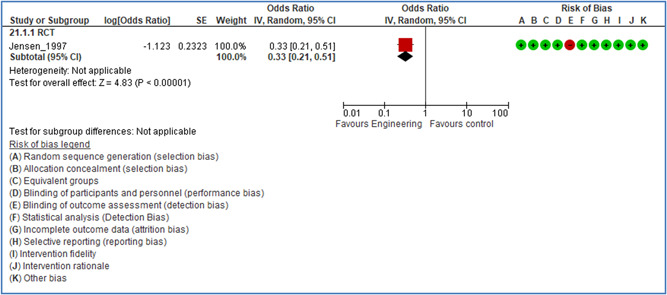


Comparison A22: Engineering control versus usual control conditions, at short‐term FU, for outcome: 22.1 Injuries.


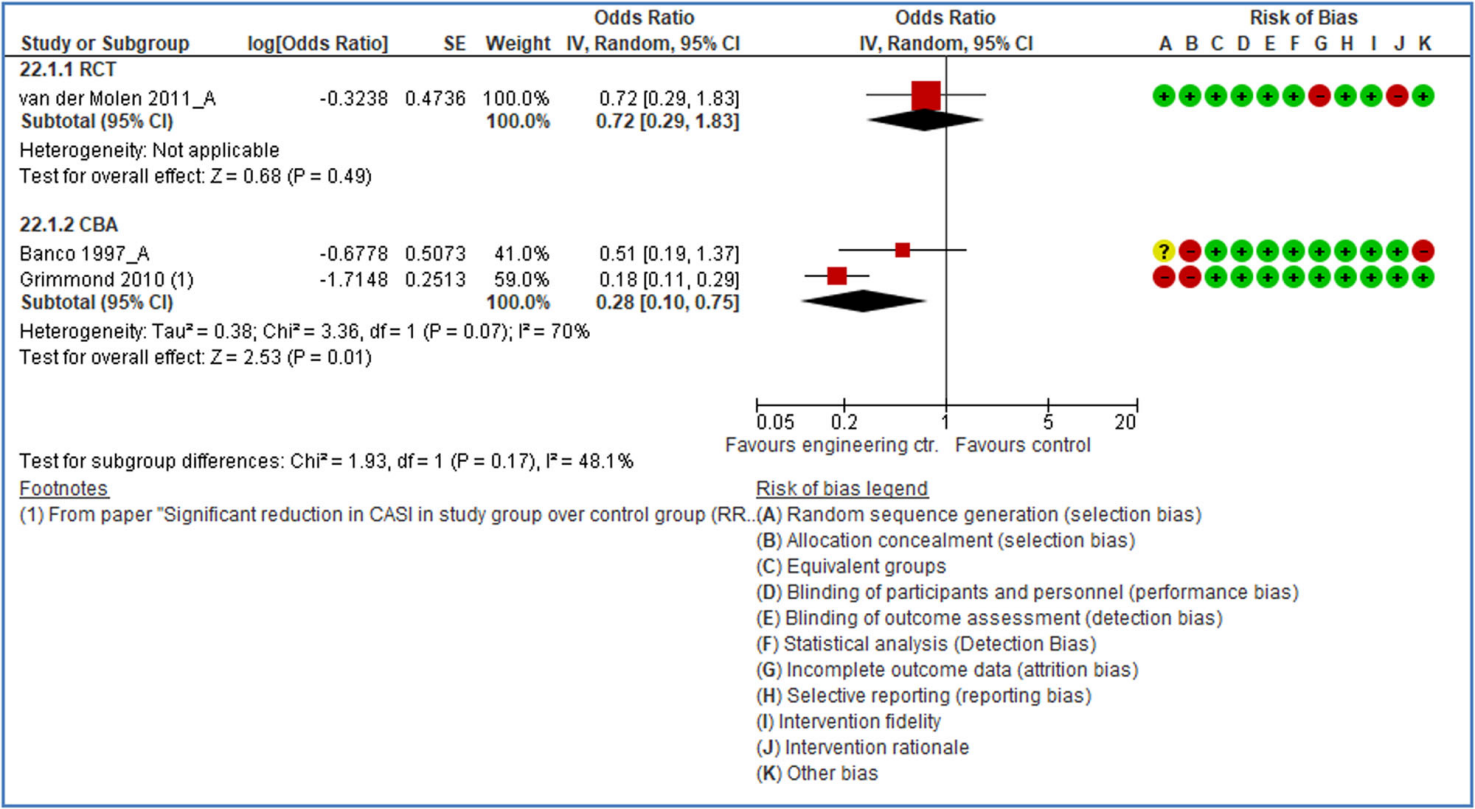


Comparison A22: Engineering control versus usual control conditions, at short term FU, for outcome: 22.2 Risk and Behavior.


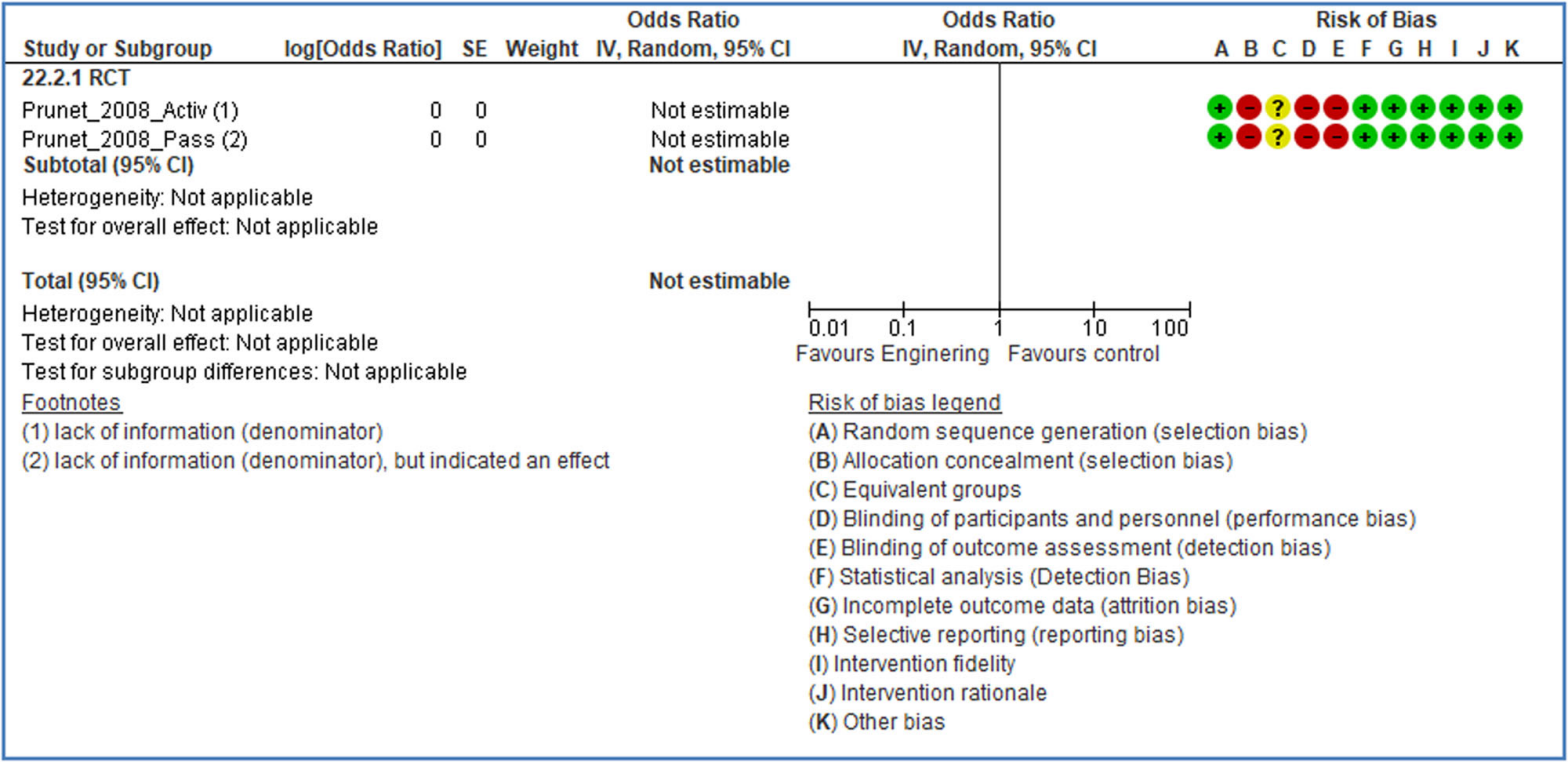


Comparison A23: Engineering control versus usual control conditions, at medium term FU, for outcome: 23.1 Injuries.


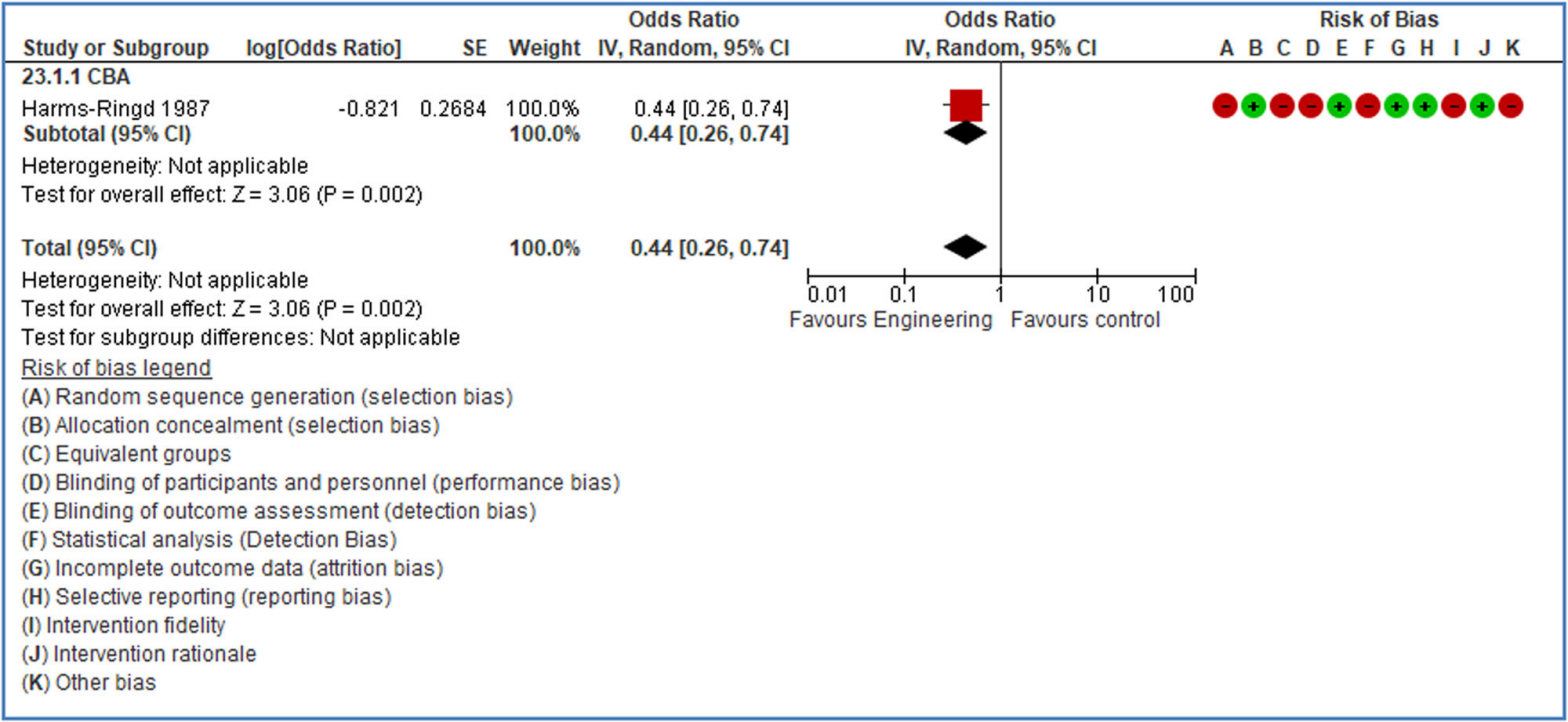


Comparison: A24 Engineering control versus usual control conditions, at long term FU, for outcome: 24.1 Injuries.


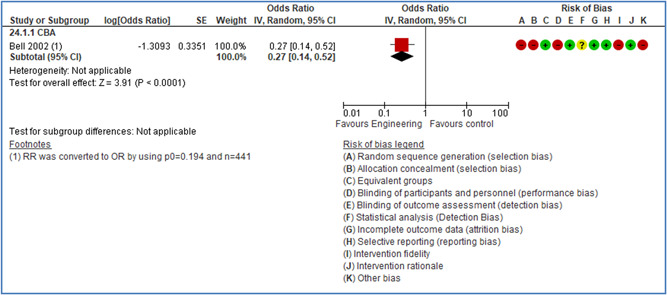


Comparison A25: Multifaceted safety interventions at the individual level versus usual control conditions, at short‐term FU, for outcome: 25.1 Injuries.


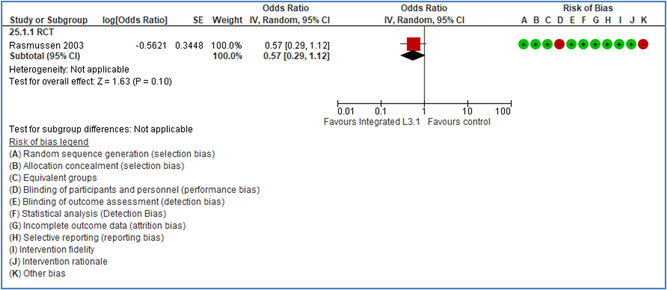


Comparison A26: Multifaceted safety interventions at the individual level versus usual control conditions, at medium‐term FU, for outcome: 26.1 Injuries.


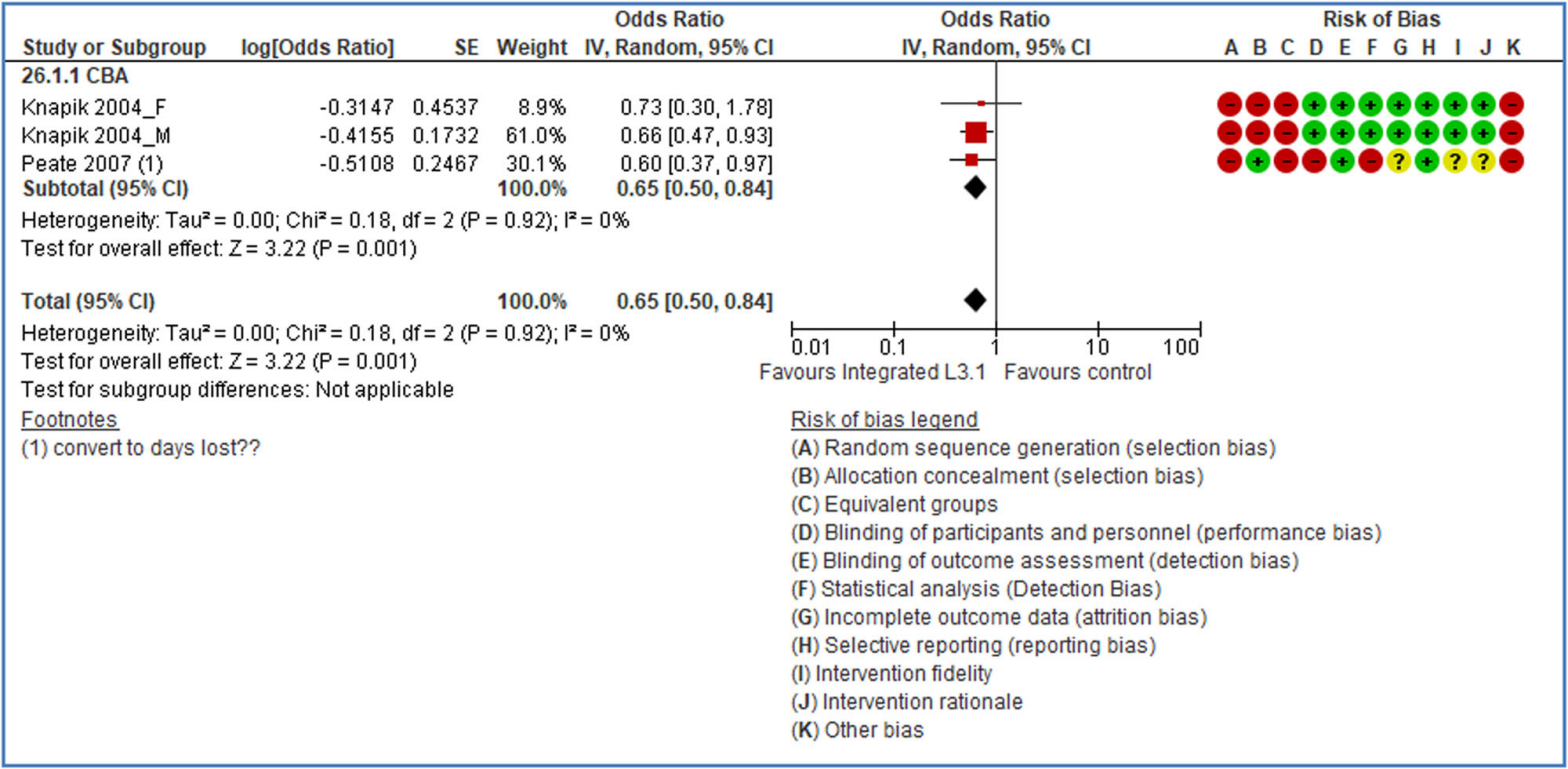


Comparison A27: Multifacet safety interventions at the individual level versus usual control conditions, long‐term FU, for outcome: 27.1 Injuries


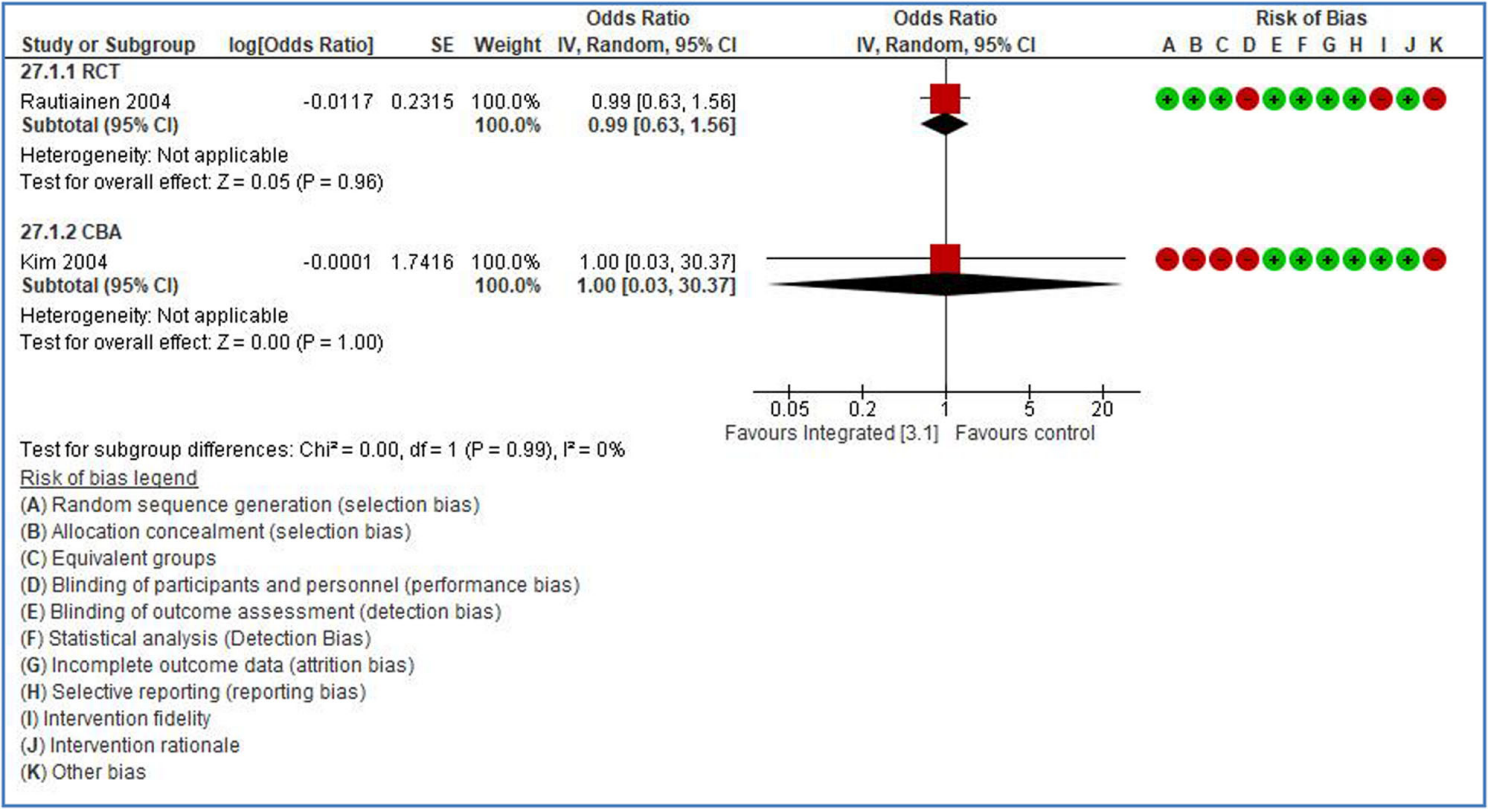


Comparison A27: Multifacet safety interventions at the individual level versus usual control conditions, at long‐term FU, for outcome: 27.2 Risk behavior.


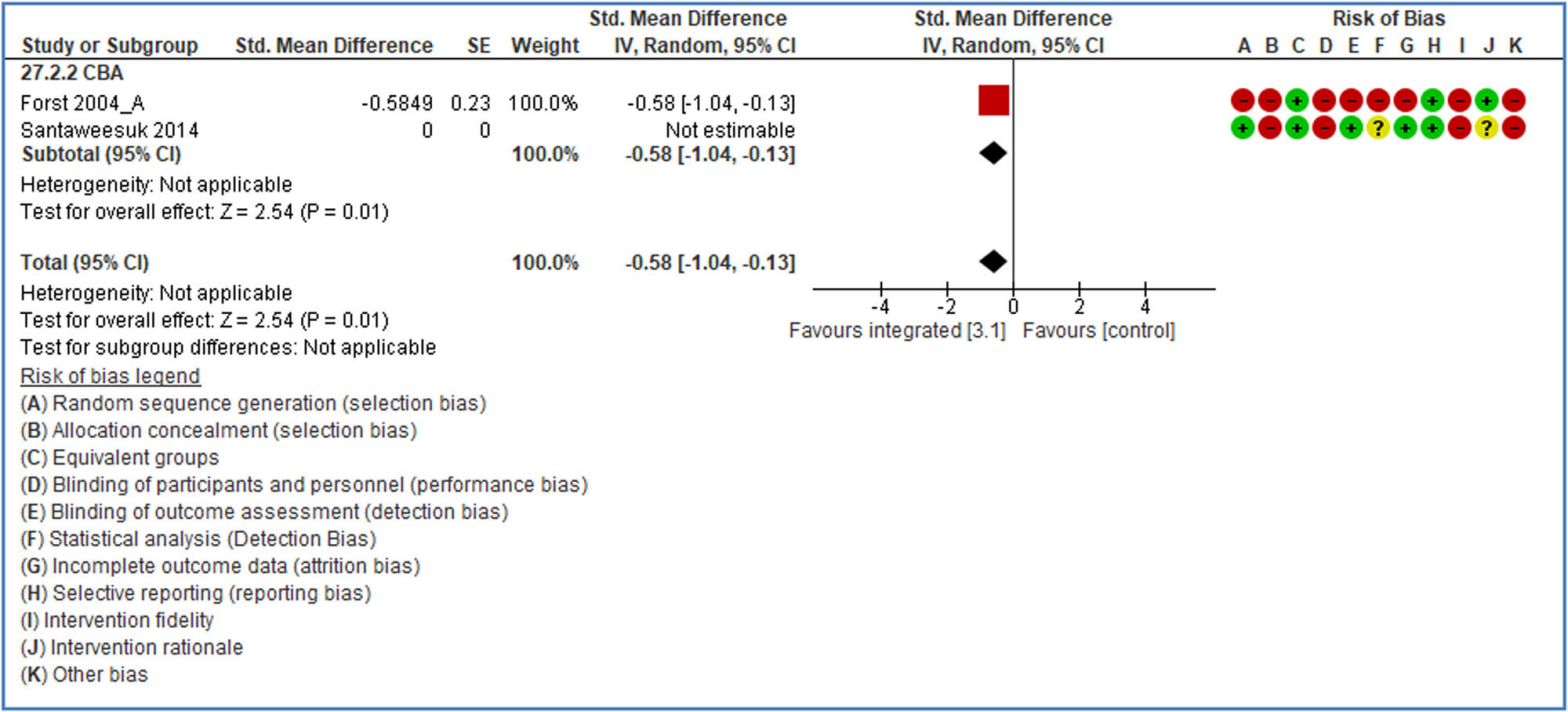


Comparison: A28 Multifaceted safety interventions at the organizational level versus usual control conditions, at medium‐term FU, for outcome: 28.1 Injuries


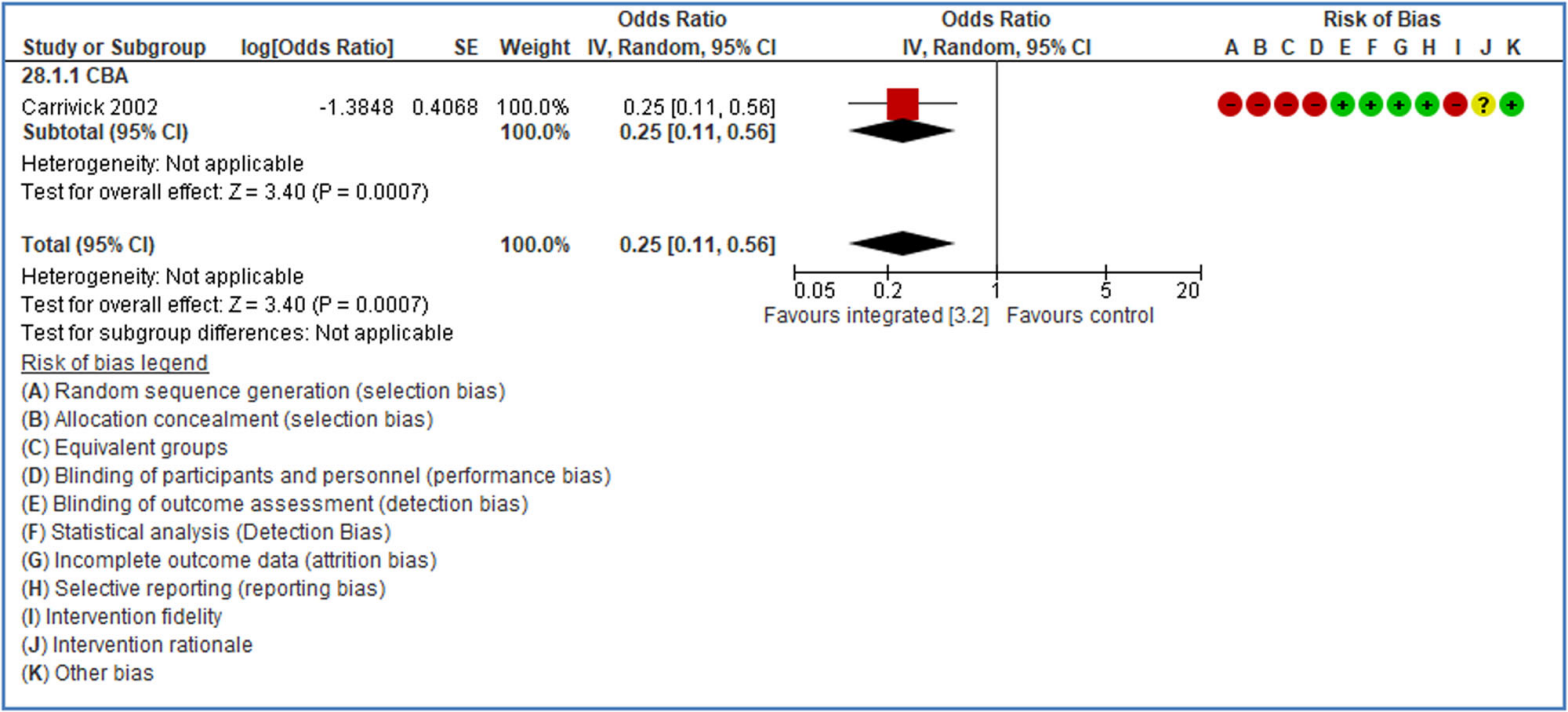


Comparison A29: Multifaceted safety interventions at the organizational level versus usual control conditions, at long‐term FU, for outcome: 29.1 Injuries.


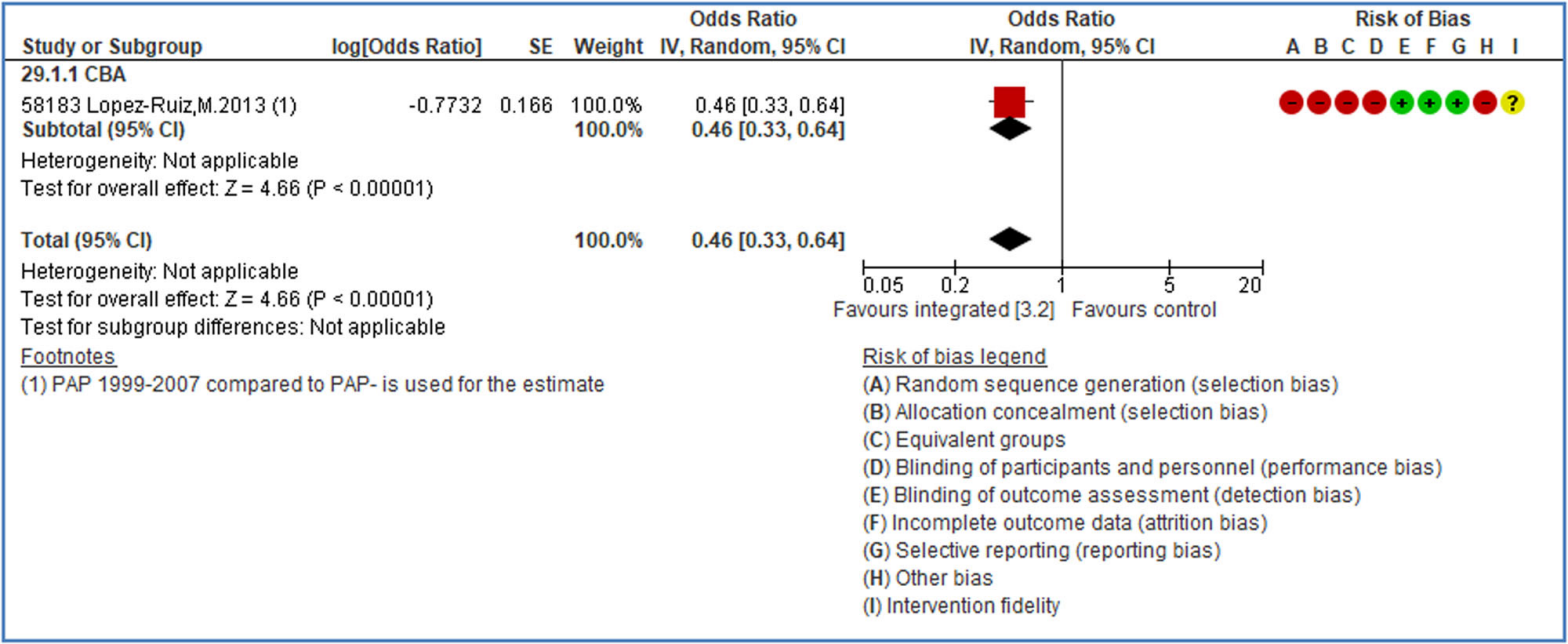


Comparison A30: Multifaceted safety intervention across levels versus usual control conditions, at short‐term FU, for outcome: 30.1 injuries.


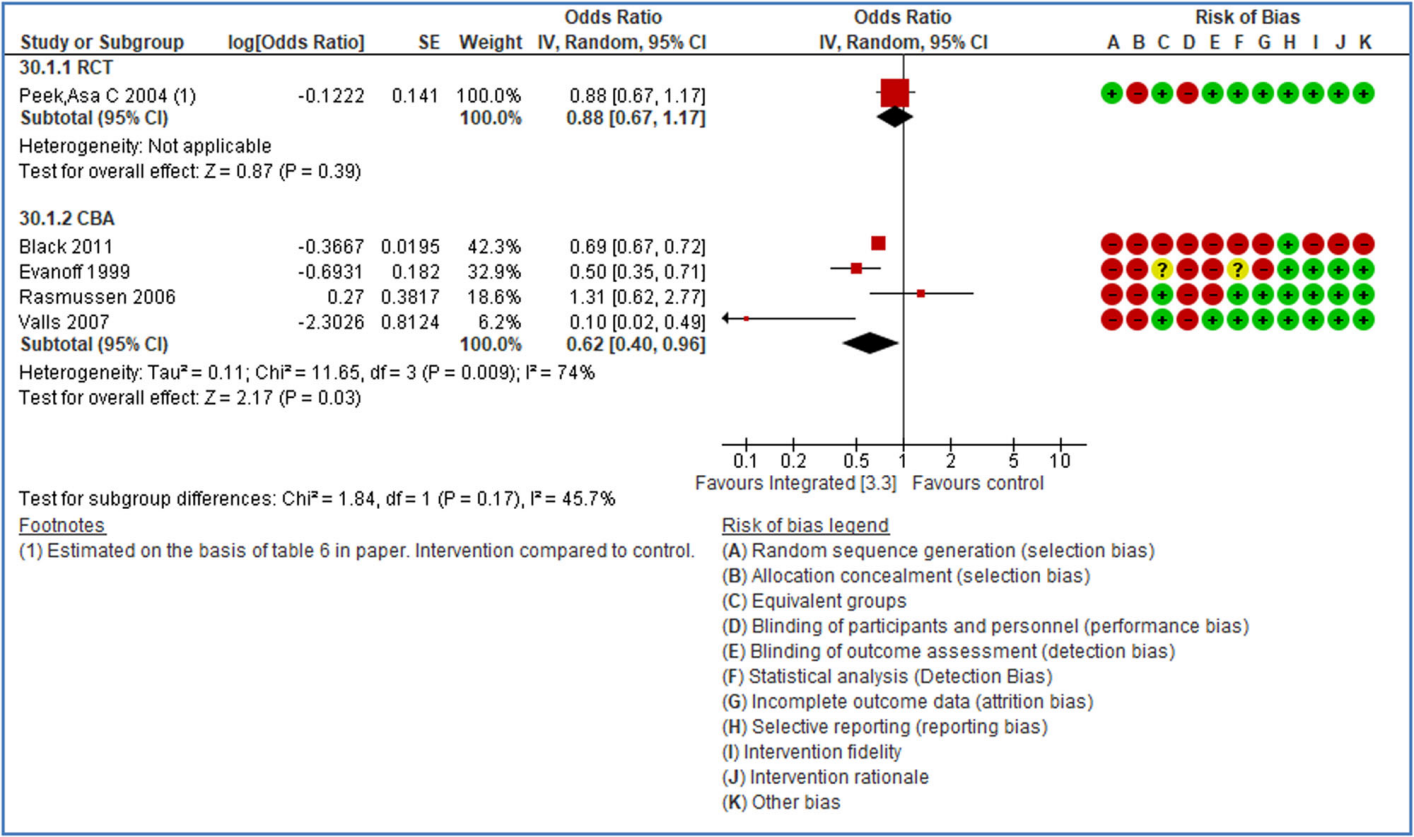


Comparison A30: Multifaceted safety intervention across levels versus usual control conditions, at short‐term FU, for outcome: 30.2 Risk and behavior.


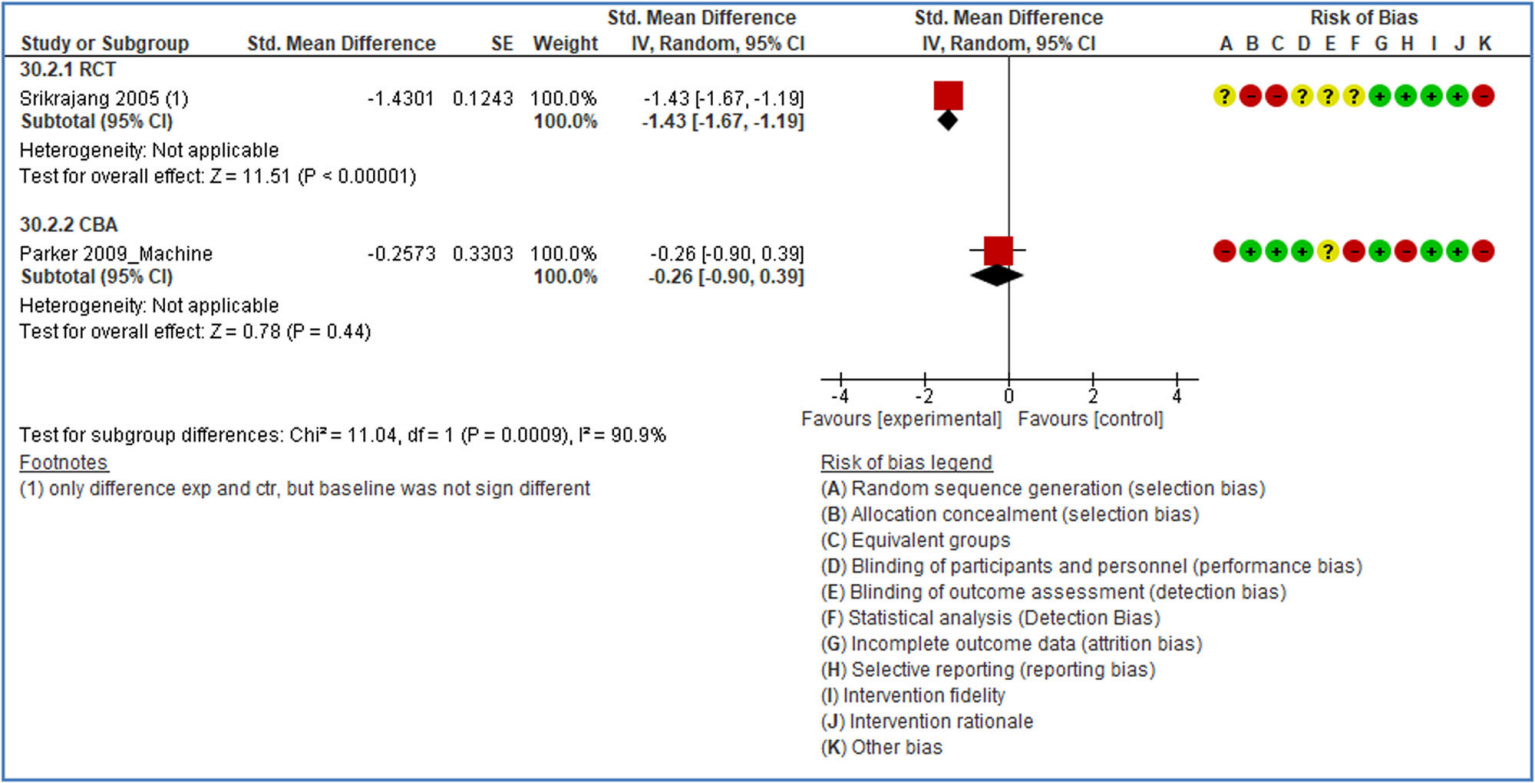


#### 11.1.2 Forest plots for study designs using serial measures, by type of safety intervention (comparison)

For the following forest plots of studies using serial measures no meta‐analyses has been included, as decided at the protocol stage. Only the point estimates for each study for each type of safety interventions (comparison) has been presented.

In the Table 18, is shown the list of risk of bias dimensions included and the two quality criteria used for the assessment of the quality of the studies.

**Table 18:** Risk of bias dimensions (A–J) referring to legends in comparison B1–B17.

Risk of bias	Legend
A	Maturation
B	Shape of intervention pre‐spec
C	Intervention likely to affect data coll.
D	Blinding of outcome assessment (detection bias)
E	Statistical analysis (Detection Bias)
F	Incomplete outcome data (attrition bias)
G	Selective reporting (reporting bias)
H	Intervention fidelity
I	Intervention rationale (why the intervention should work)
J	Other bias

Comparison B1: Teaching and education, at long‐term follow‐up (FU), for outcome: 1.1 Injuries.


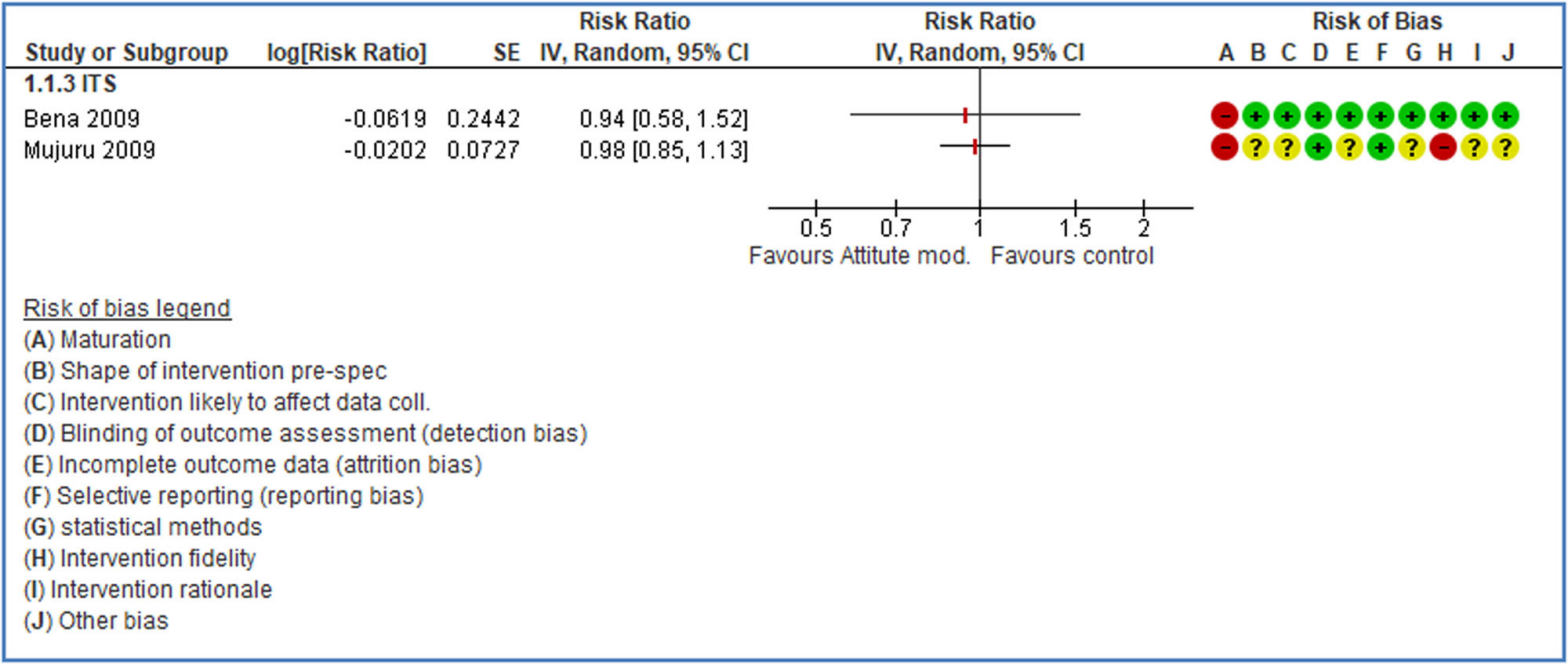


Comparison B2: Individual physical training, at long‐term FU, for outcome: 2.1 Injuries


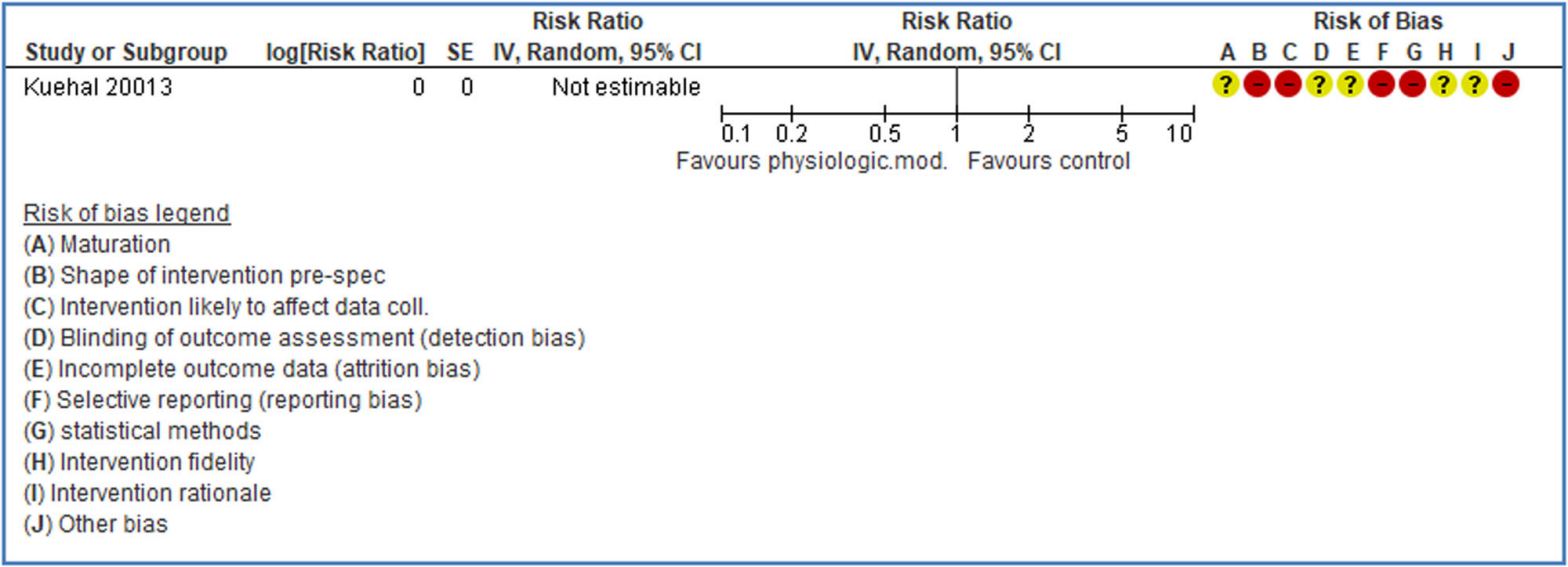


Comparison B3: Leader‐based safety interventions, at short‐term FU, outcome: 3.1 Risk behavior.


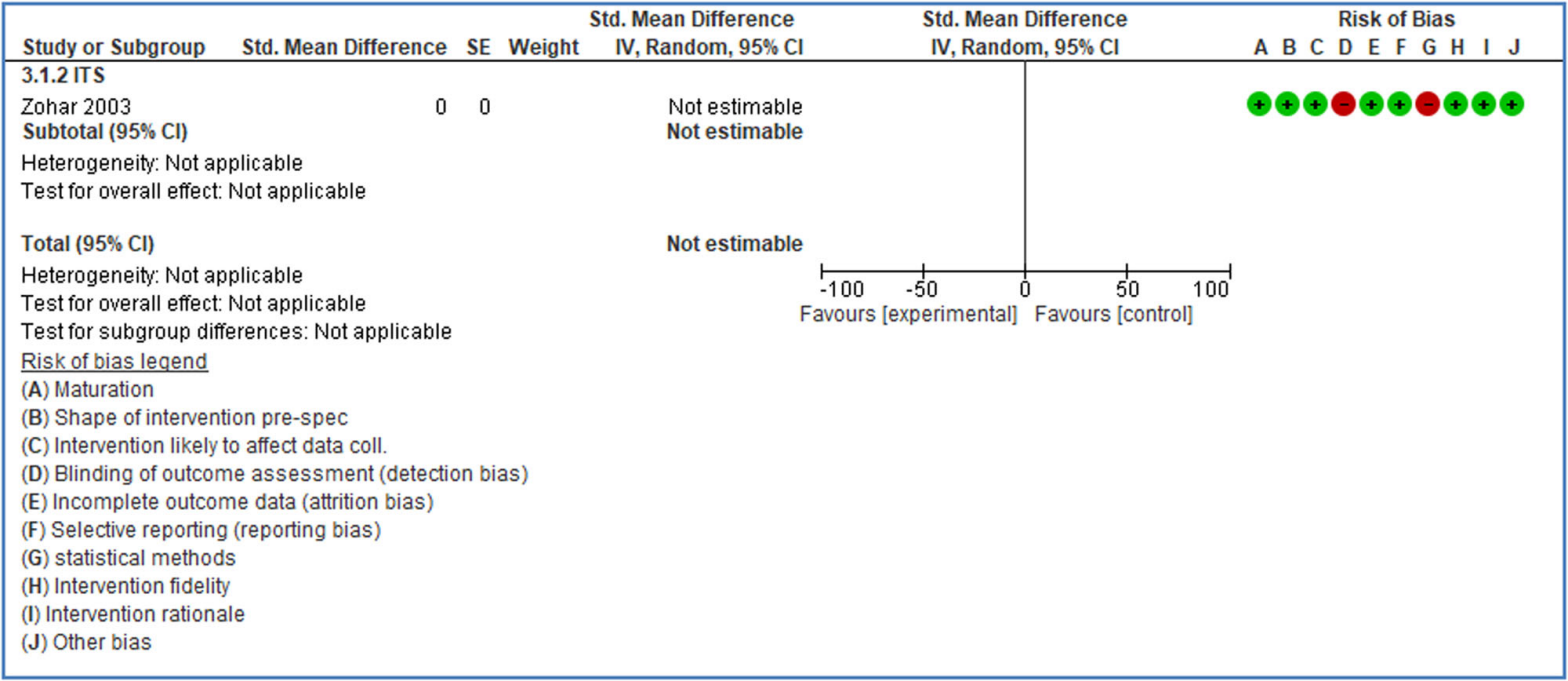


Comparison B4: Safety feedback at Group level, at short‐term FU, for outcome: 4.1 Injuries


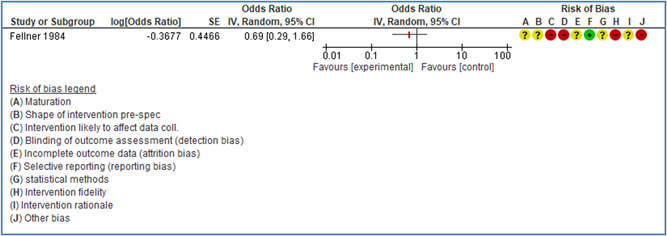


Comparison B4: Safety feedback at Group level, at short‐term FU, for outcome: 4.2 Risk and behavior.


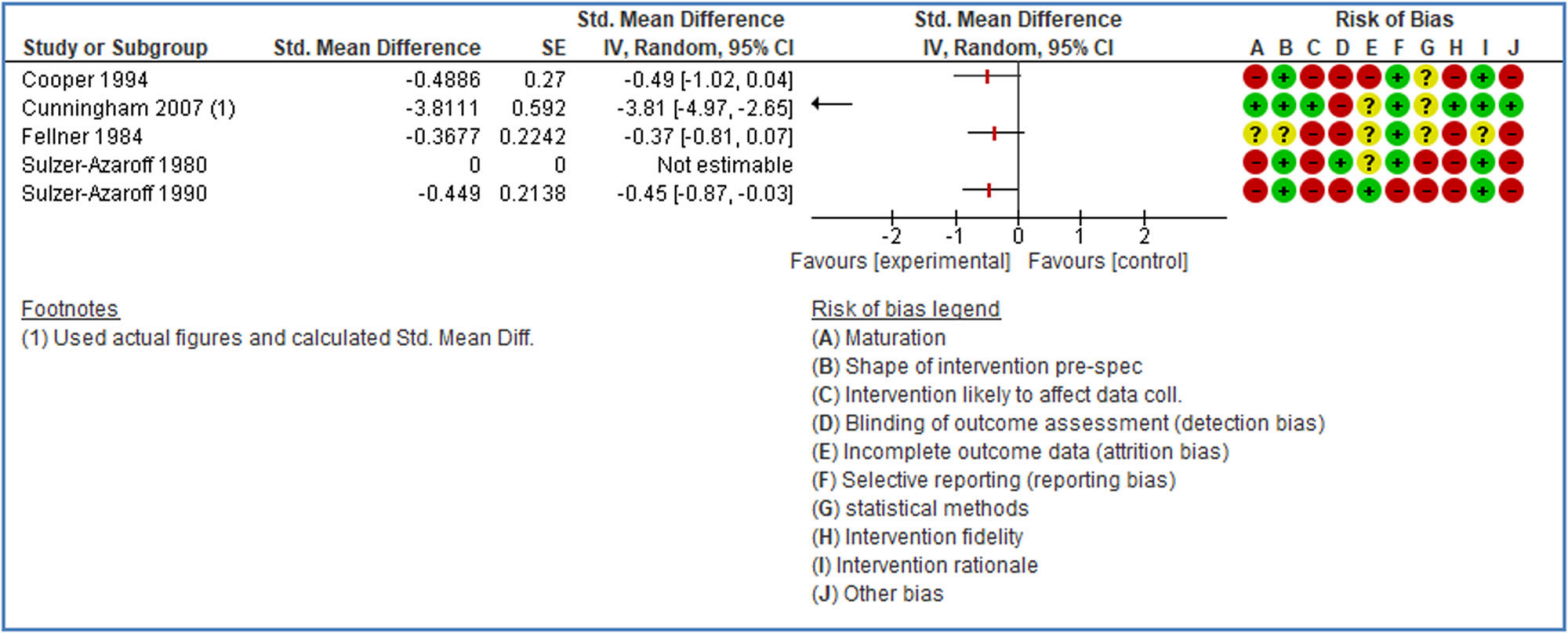


Comparison B5: Legislative changes, at long‐term FU, for outcome: 5.1 Injuries.


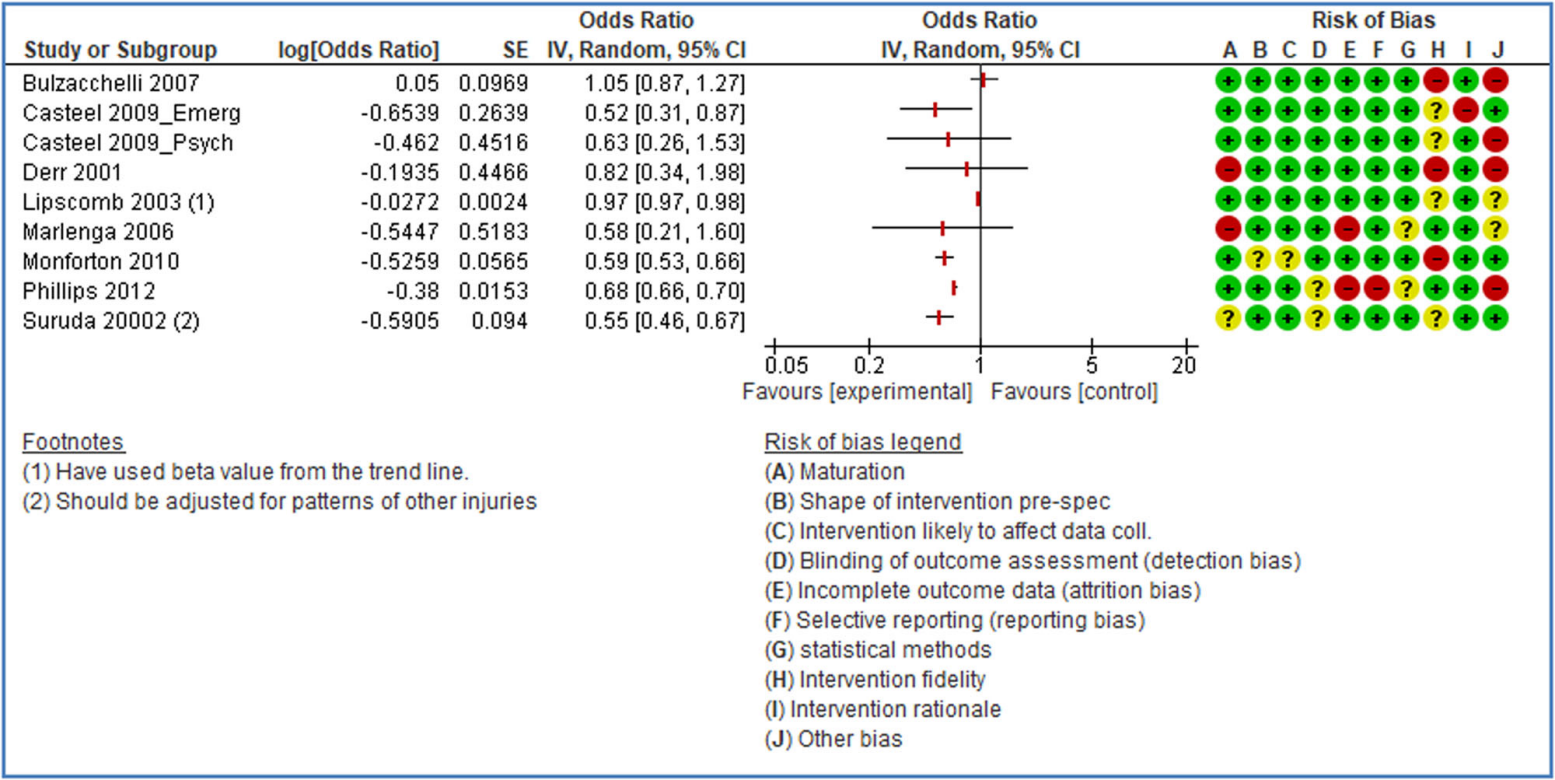


Comparison B6: Enforcement and compliance, at long‐term, for outcome: 6.1 Injuries.


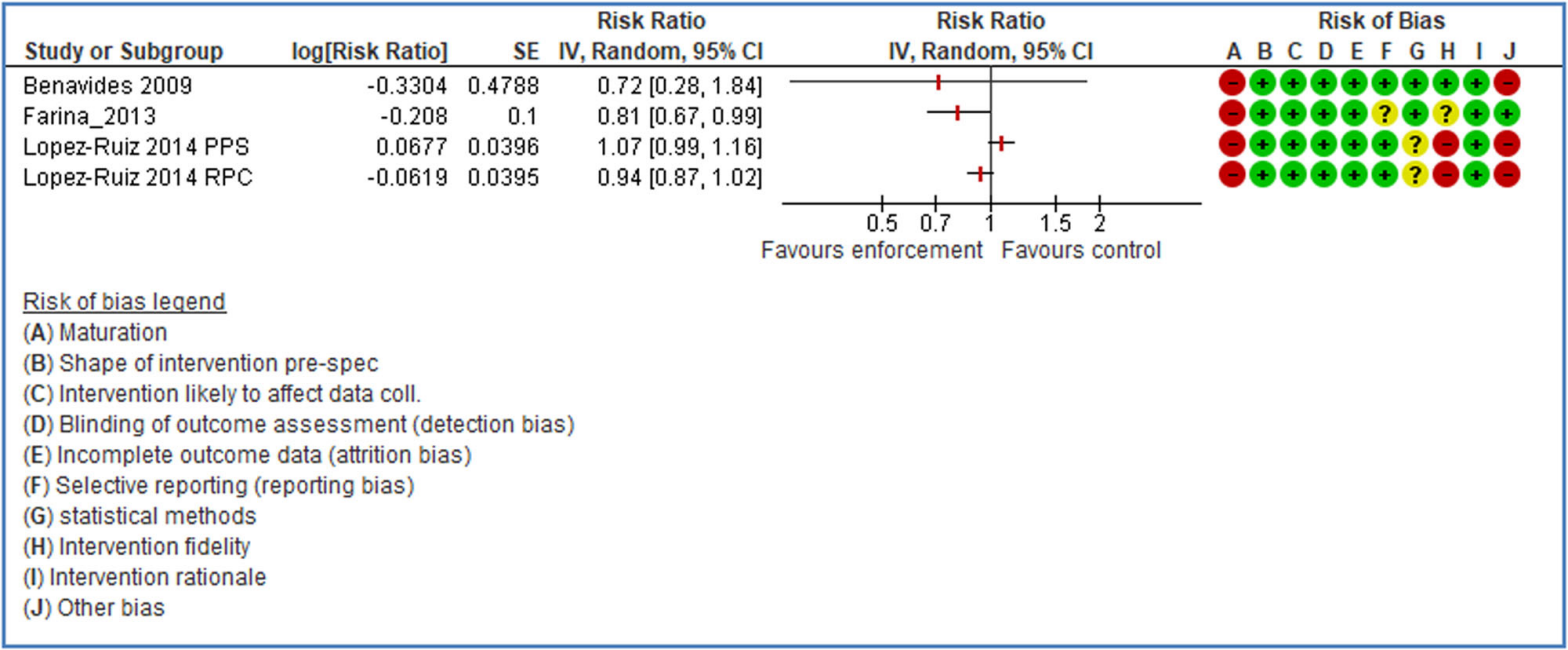


Comparison B7: Economic incentives, at long‐term FU, for outcome: 7.1 Injuries.


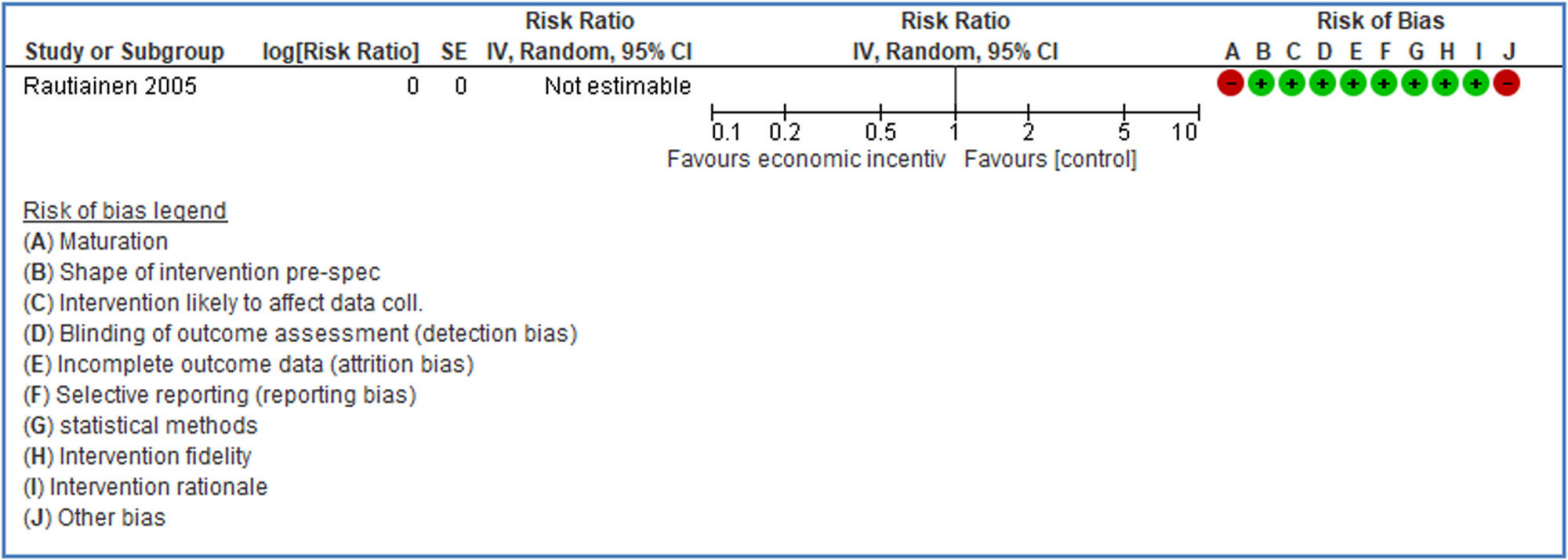


Comparison B8: Engineering control, at short‐term FU, for outcome: 8.1 Injuries.


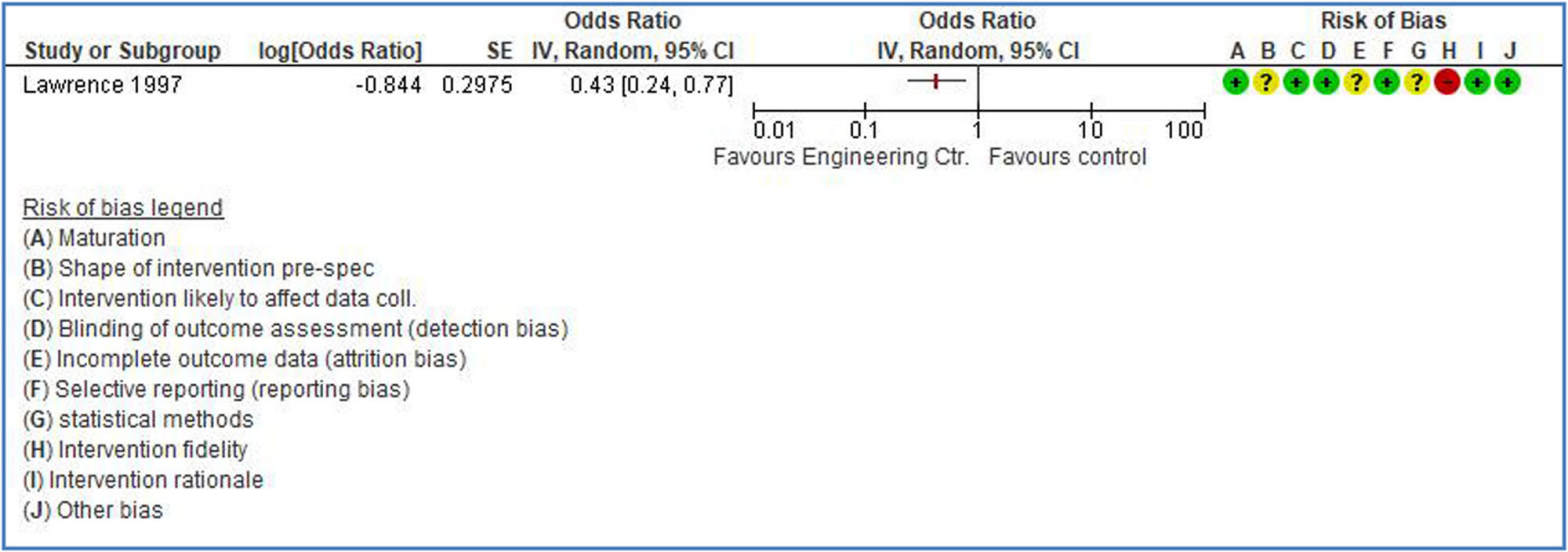


Comparison B9: Engineering control, at medium‐term FU, for outcome: 9.1 Injuries.


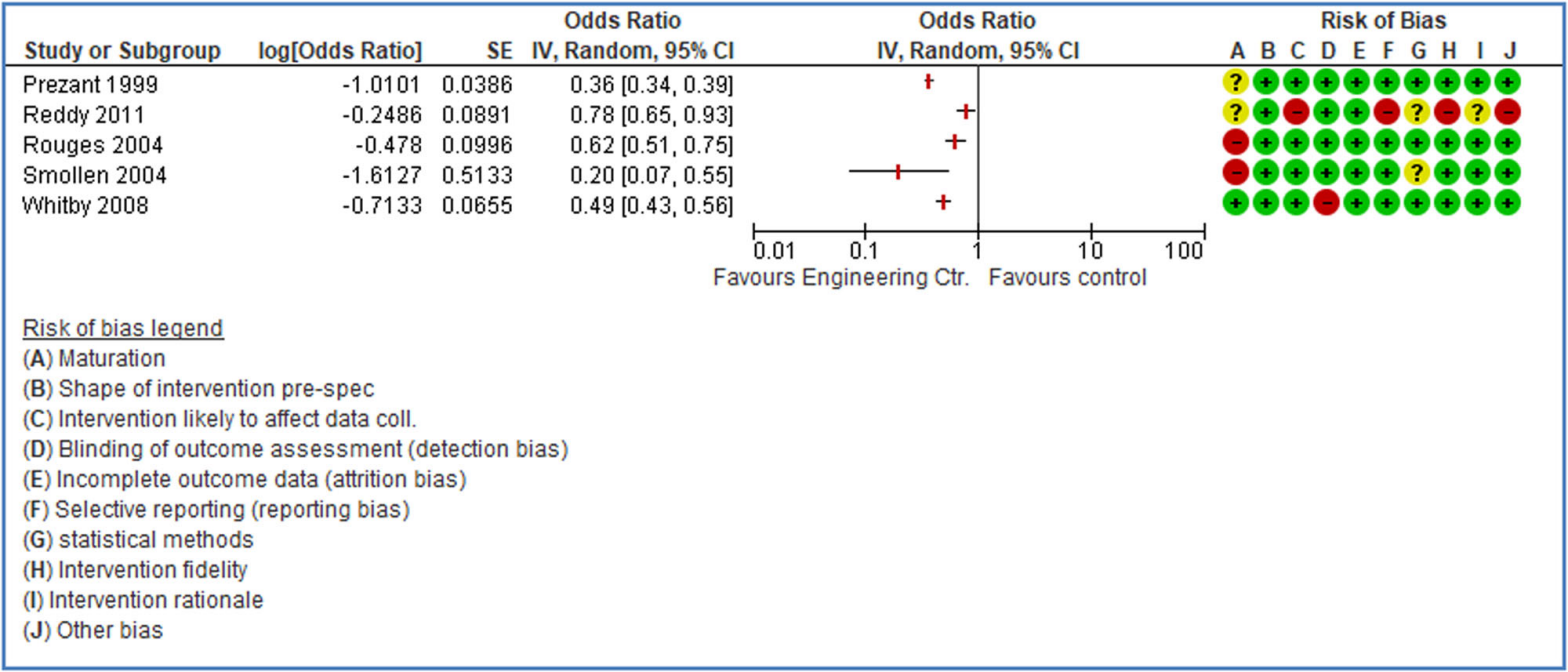


Comparison B10: Engineering control, at long‐term FU, for outcome: 10.1 Injuries.


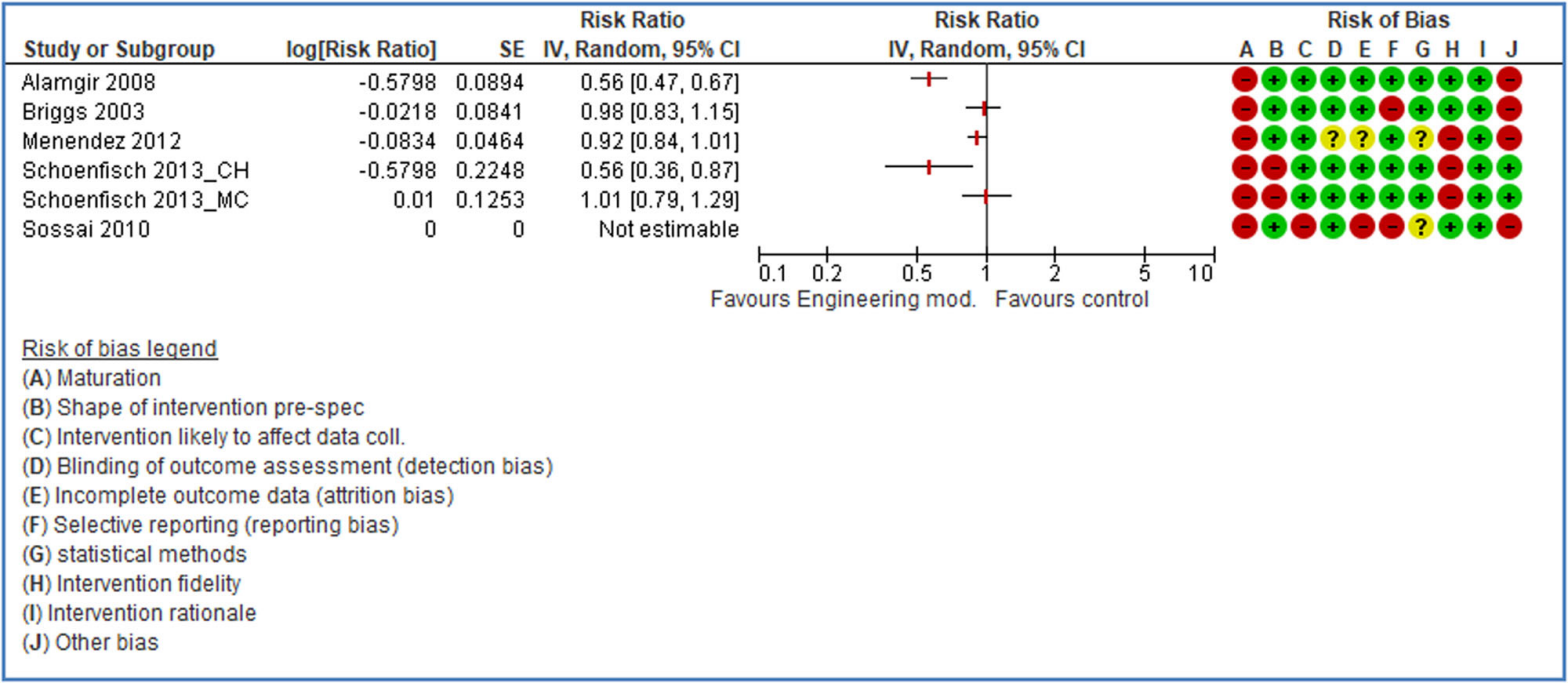


Comparison B11: Administrative controls, at short‐term FU, for outcome: 11.1 Injuries.


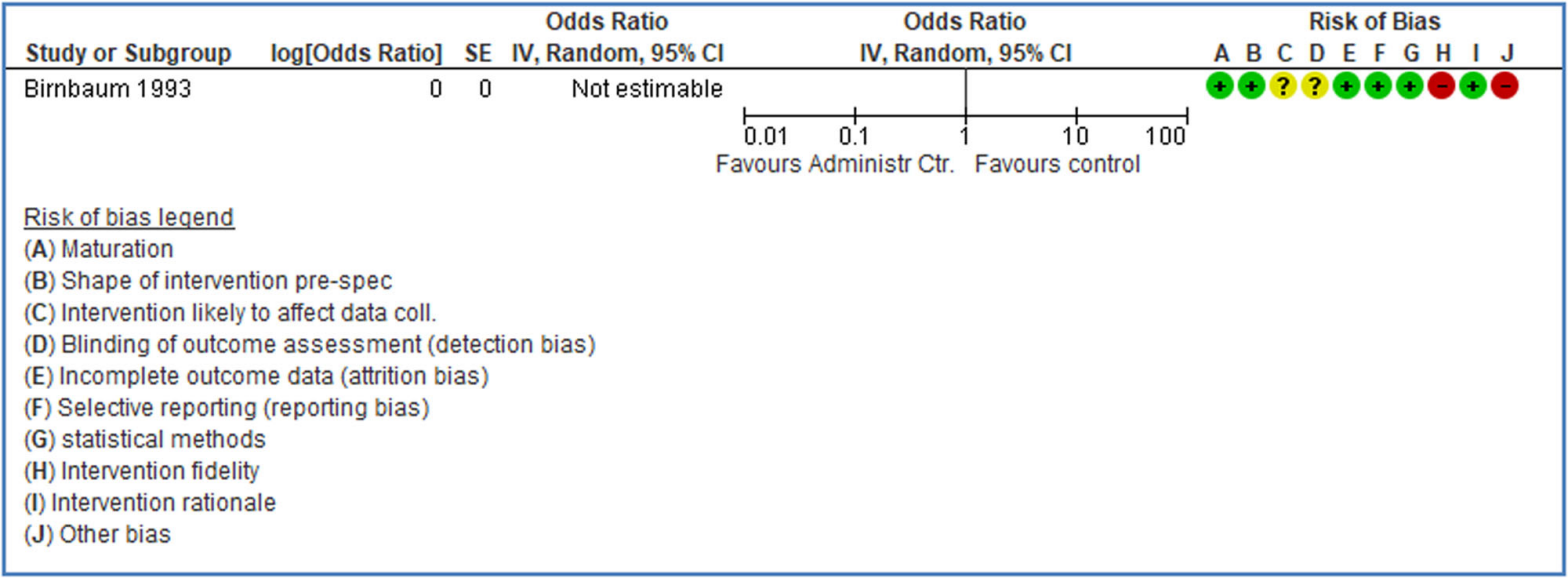


Comparison B12: Administrative controls, at medium‐term FU, outcome: 12.1 Injuries.


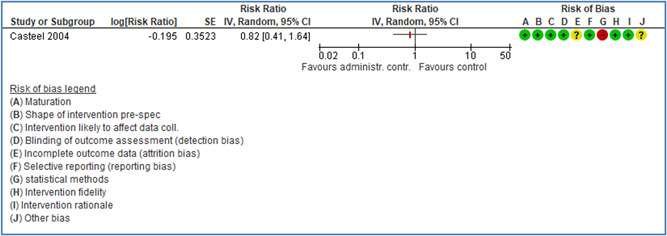


Comparison B13: Multifaceted safety interventions at the organizational level, at medium‐term FU, for outcome: 13.1 Injuries.


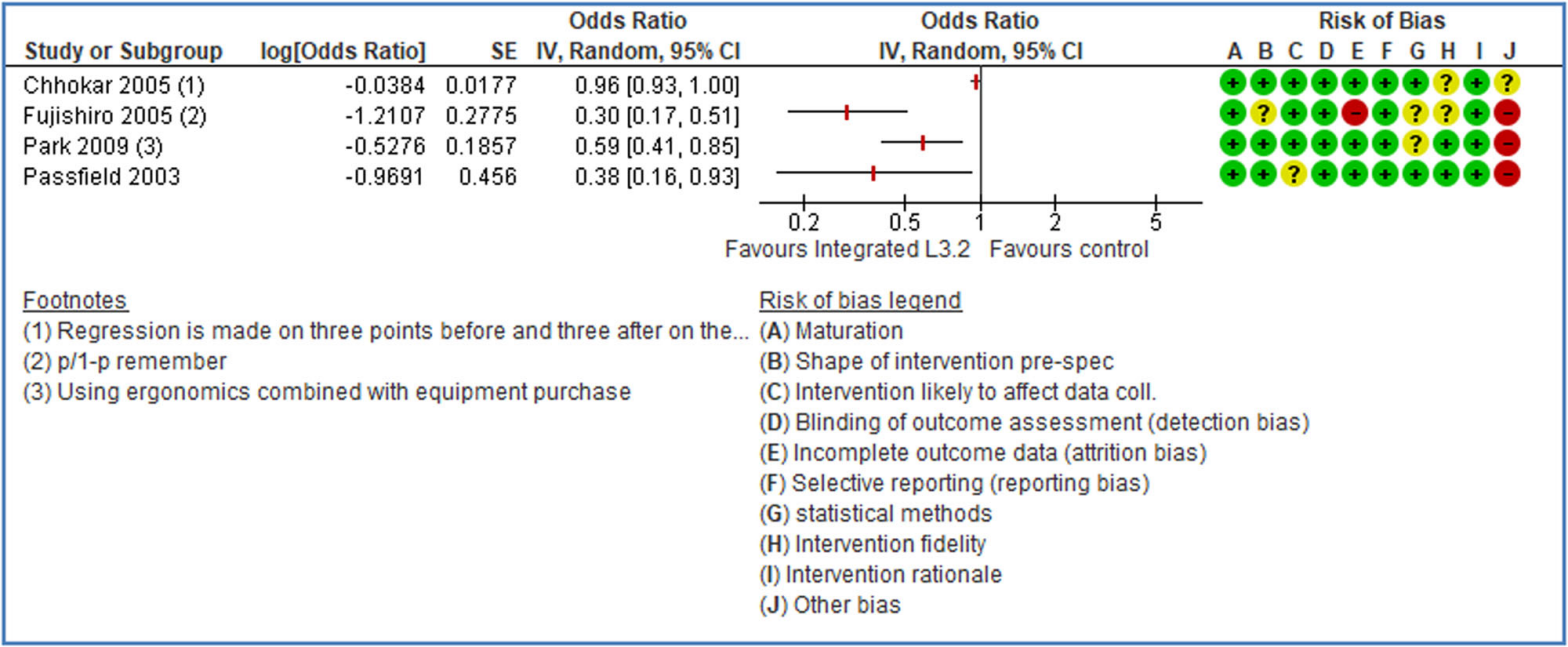


Comparison B14: Multifaceted safety interventions at the organizational level, at long‐term FU, for outcome: 14.1 Injuries.


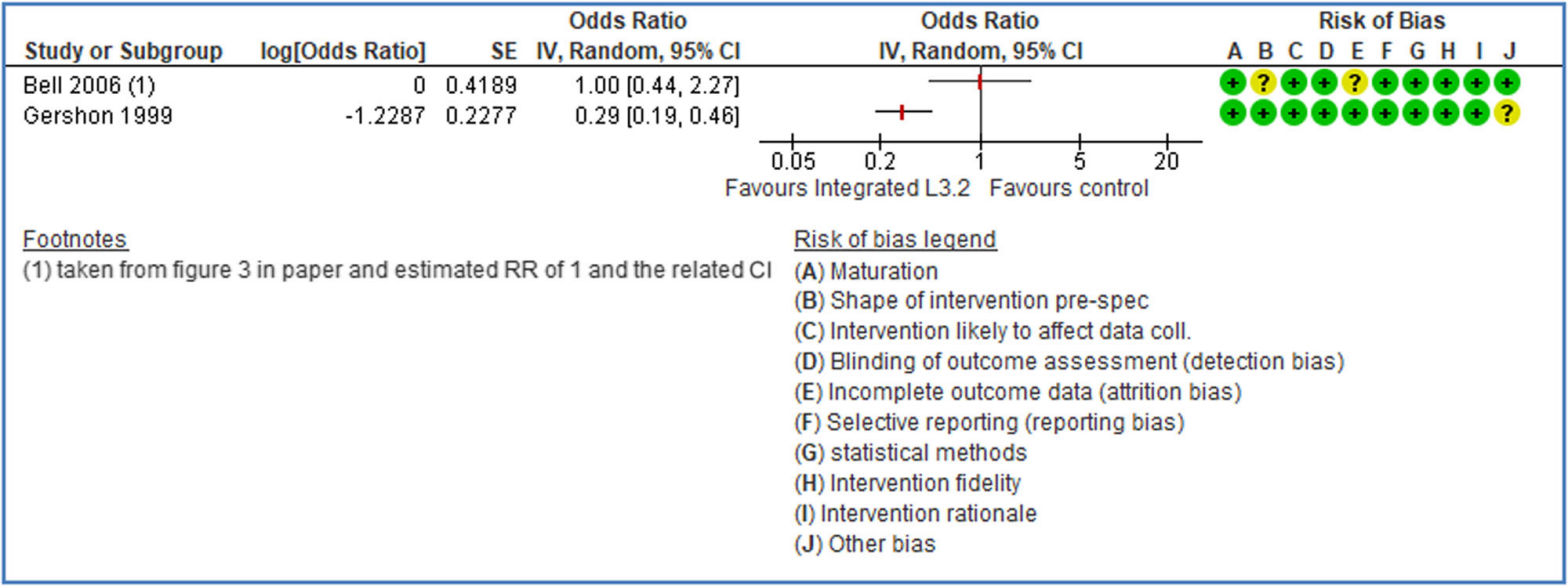


Comparison B15: Multifaceted safety interventions across levels, for medium‐term FU, for outcome: 15.1 Injuries.


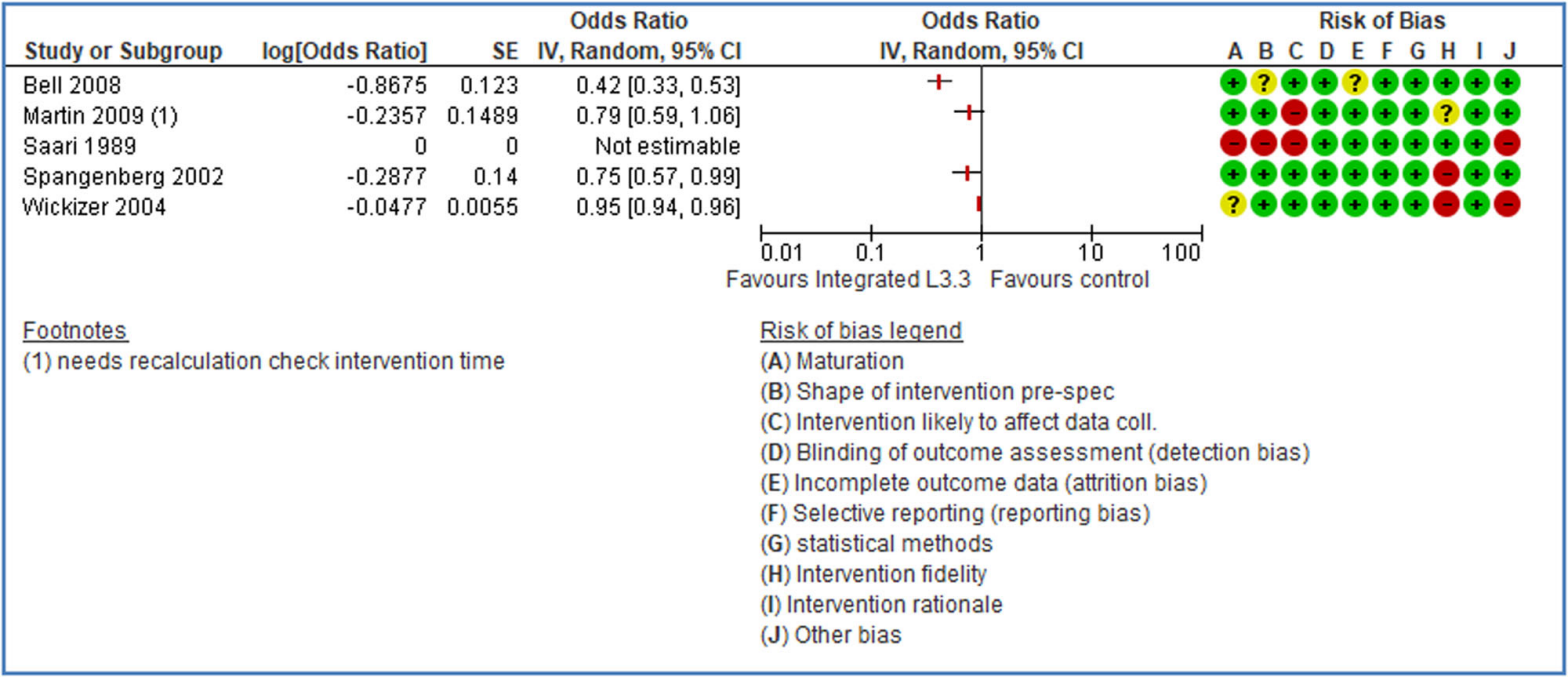


Comparison B16: Multifaceted safety interventions across levels, at long‐term FU, for outcome: 16.1 Injuries.


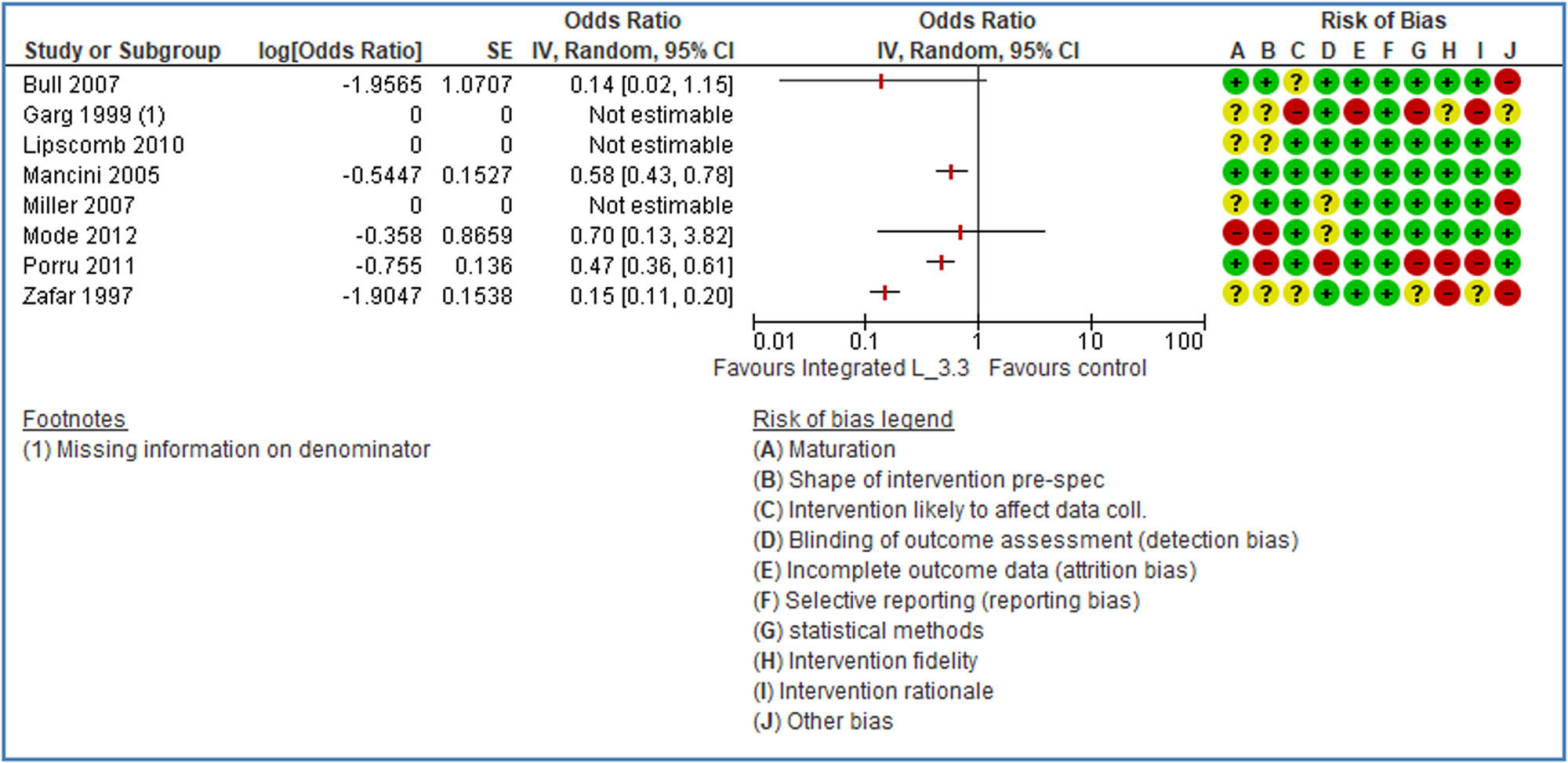


Comparison B17: Social marketing, at unknown FU, for outcome: 17.1 Risk and Behavior


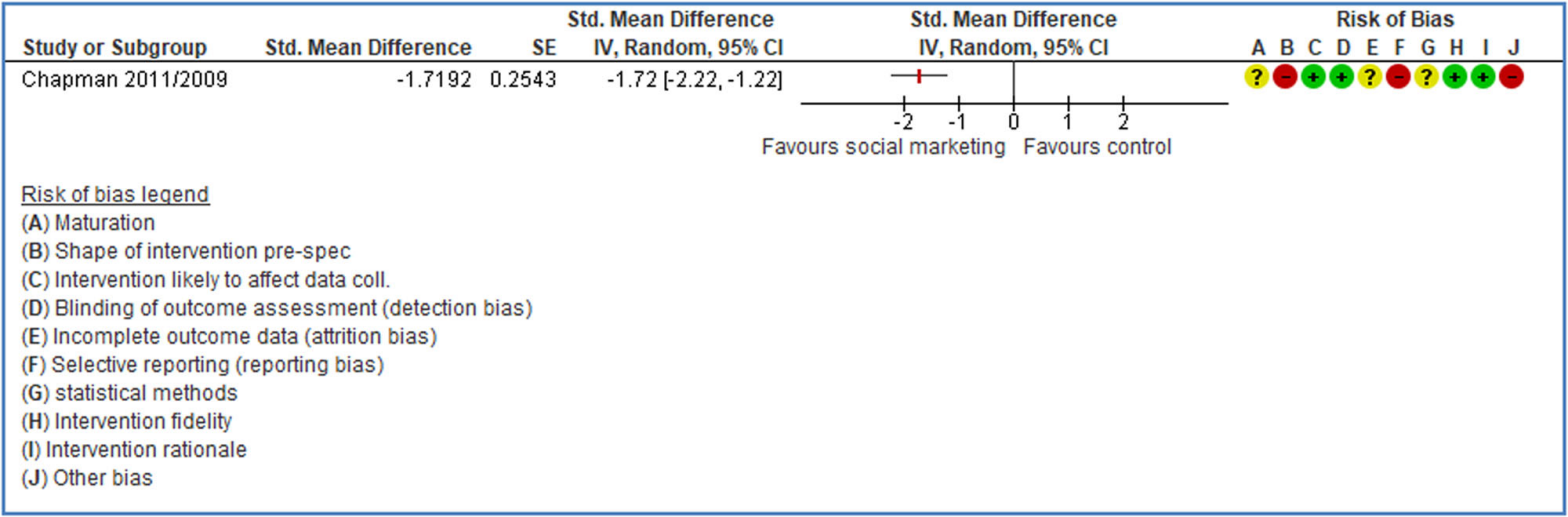


### 11.2 Sensitivity analysis

#### 11.2.1 Sensitivity analysis for the type of outcome

Risk and behavior can be seen as an intermediate variable that to some extend can be seen as a proxy for injury outcomes. However, they are also less objective measures of outcome. We conducted a sensitivity analysis to see whether excluding risk and behavior as outcome measures would change the (a) level of evidence (b) effect evaluation of each type of safety interventions.

In total seven comparisons are made on the basis of risk and behavior outcomes. Two of them we could not establish an effect measure, leaving five comparisons which could be affected by excluding studies using risk and behavior as outcome measure.

For individual feedback (Comparison 9.2) excluding risk and behavior outcomes would result in non‐significant effects at medium‐term follow‐up, and level of evidence would go from moderate to limited evidence of effects. This will not change the overall direction of effect, but in that case individual feedback would no longer have a significant effect on reducing injuries (Comparison A10.1).

For the safety intervention goal setting and feedback, the level of evidence would go from limited evidence to insufficient level of evidence if risk and behavior outcomes were excluded, and there would no longer a significant effect of goal setting and feedback (comparison 14.2)

The level of evidence will not be affected for leadership based safety interventions, but the effect measure will go from moderate to strong (analysis 15.2)

For engineering control (analysis 23.2) we included one study evaluation effect at pretest, which used risk and behavior as outcome measure. For the other follow‐up points we did not include risk and behavior in the evaluation of the effect of engineering controls.

The other types of safety interventions were not affected by excluding studies using risk and behavior as outcome variable.

To assess the robustness of the combined effect measures in meta‐analysis we did sensitivity analysis. For the comparison A32, multifaceted safety interventions across levels, we found that the combined effect estimate were not robust, as results would not be significant if we just excluded a single study. However, the overall direction in the effect measure did not change.

### 11.3 Other analyses

No other analyses were undertaken.

## 12 TABLES

In Table 19 we have presented the ranges of effect we have used in the review, and the terms we have used for the various levels of strength of effect (Tables 20 and 21).

**Table 19:** Strength of an epidemiological effect in safety interventions for ratio measures and correlations (Cohen's *d*)

Range of effect			
Strength	Rate ratio (increased risk)^a^	Rate ratio (decreased risk)^a^	Cohen's d correlation^b^
None	1.0–1.1	0.95–1.0	<*r* = .10
Little	1.1–1.3	0.75–0.95	*r* = .10 – *r* = .30
Moderate	1.3–1.7	0.55–0.75	*r* = .30 – *r* = .50
Strong	1.7 < 2.0	0.35–0.55	*r* = .50 – *r* = .70
Very strong	2.0<	<0.35	*r* = .70<

^a^ Adjusted from Monson (1990).

^b^ Adjusted from Cohen 1988.

**Table 20:** Summary of meta‐analysis for a subset of safety interventions, by type of safety intervention, quality assessment, level of evidence, strength of effect, and heterogeneity

Number of safety interventions	Quality assessment				Level of evidence	Strength of effect	Meta‐analysis (injury outcomes)		Hetero‐geneity
Types of safety interventions and follow‐up periods	High quality	Moderate quality	Low quality	Total	RCT and CBA	RCT and CBA	RCT	CBA	I^2 RCT/CBA^
**1.1.0.: Attitude and belief modification:**									
**1.1.1** Safety campaign, by use of various means	**1**		**1**	**2**					
2 = Short‐term (−12 months)			1	1	insufficient	*Not estimable*			
3 = Medium‐term (12–36 months)	1			1	insufficient	*Not estimable*			
**1.1.2** Counseling approaches	**1**	**3**		**4**					
2 = Short‐term (−12 months)	1	1		2	Limited	None	OR: 0.67 [0.38, 1.21]		0%
3 = Medium‐term (12–36 months)		2		2	Limited	Strong	**OR: 0.52 [0.29, 0.93]**	**OR: 0.53 [0.36, 0.77]**	NA/NA
**1.1.3** Teaching, education to increase awareness	**1**	**1**	**1**	**3**					
2 = Short‐term (−12 months)	1	1	1	3	Limited	None		OR: 0.90 [0.56, 1.46]	37%
**1.3.0.: Physiological modification:**									
**1.3.1** Individual physical training	**1**	**2**	**1**	**4**					
2 = Short‐term (−12 months)	1	2		3	Limited	None		OR: 1.04 [0.75, 1.44]	0%
3 = Medium‐term (12–36 months)			1	1	insufficient	*Not estimable*			/
**2.2.0.: Structural safety interventions:**									
**2.2.4** Engineering controls	**4**	**2**		**6**					
1 = Posttest	1			1	Limited	**Strong to very strong**	**OR: 0.33 [0.21, 0.51]**		NA
2 = Short‐term (−12 months)	3			3	Strong	Moderate	**OR: 0.72 [0.29, 1.83]**	**OR: 0.28 [0.10, 0.75]**	NA/70%
3 = Medium‐term (12–36 months)		1		1	insufficient	Strong		**OR: 0.44 [0.26, 0.74]**	NA
4 = Long‐term (36‐month)		1		1	insufficient	**Very strong**		**OR: 0.27 [0.14, 0.52]**	NA
**2.2.7** Enforcement of laws and regulations	**7**		**4**	**11**					
2 = Short‐term (−12 months)	1			1	Limited	Little		**OR: 0.86 [0.77, 0.95]**	NA
3Medium‐term (12–36 months)	2		4	6	Moderate	**None to little**	**OR: 0.99 [0.89, 1.10]**	**OR: 0.95 [0.93, 0.97]**	NA/0%
4 = Long‐term (36‐month)	4			4	Strong	Little		**OR: 0.96 [0.93, 0.98]**	0%
**2.2.7** Enforcement of laws w/penalties	**2**			**2**					
3 = Medium‐term (12–36 months)	2			2	Moderate	**None to little**		**OR: 0.95 [0.92, 0.98]**	0%
**3.0. Multifaceted safety interventions:**									
**3.3 Multifaceted across levels**	**2**	**1**	**2**	**5**					
2 = Short‐term (−12 months)	2	1	2	5	Moderate	None to moderate	**OR: 0.88 [0.67, 1.17]**	**OR: 0.62 [0.40, 0.96]**	NA/74%
	14	4	6	24					

Abbreviations: *I* ^2^, Heterogeneity; NA, Not applicable.

**Table 21:** Assessment of safety interventions not included in meta‐analysis, by type of safety intervention, quality assessment, level of evidence, and estimated strength of effect

Number of safety interventions	Quality assessment				Level of evidence	Strength of effect
Type of safety intervention and follow‐up periods	High quality	Moderate quality	Low quality	Total	RCT, CBA, and serial measures (ITS)	RCT, CBA, and serial measures (ITS)
**1.1.0.: Attitude and belief modification, not specified:**			**1**	**1**		
**1.1.3 Teaching, education to increase awareness**	**1**		**1**	**2**		
3 = Medium‐term (12–36 months)	1		1	2	Limited	None
**1.2.0.: Behavior modification**						
**1.2.2 Safety training**	**2**			**2**		
2 = Short‐term (−12 months)	1			1	Limited	None
3 = Medium‐term (12–36 months)	1			1	Limited	Moderate
**1.2.4 Individual feedback or coaching**	**3**		**2**	**5**		
0 = Not reported or unclear			1	1	insufficient	*Not estimable*
2 = Short‐term (−12 months)	2		1	3	Moderate	**None to little**
4 = Long‐term (36‐month)	1			1	Limited	None
**1.3.0.: Physiological modification:**						
**1.3.1 Individual physical training**	**0**	**0**	**1**	**1**		
4 = Long‐term (36‐month)			1	1	insufficient	*Not estimable*
**2.1.0. Climate, norms or culture modifications:**						
**2.1.1 Goal setting and FB at group or org. level**	**0**	**2**	**5**	**7**		
2 = Short‐term (−12 months)		2	5	7	Limited	None
**2.1.7 Leadership‐based safety interventions**	**1**	**2**	**1**	**4**		
2 = Short‐term (−12 months)	1	2	1	4	Limited	**Little to moderate**
**2.2.0. Structural modifications:**						
**2.2.1 Legislative changes**	**1**	**0**	**8**	**9**		
4 = Long‐term (36‐month)	1	0	8	9	Limited	**Little to moderate**
**2.2.2 Economic incentives**	**2**	**0**	**0**	**2**		
3 = Medium‐term (12–36 months)	1			1	Limited	**Little to moderate**
4 = Long‐term (36‐month)	1			1	Limited	*Not estimable*
**2.2.3 Soft regulation**	**1**	**2**	**0**	**3**		
3 = Medium‐term (12–36 months)	1			1	Limited	None
4 = Long‐term (36‐month)	0	2	0	2	Limited	None
**2.2.4 Engineering controls**	**3**	**3**	**5**	**11**		
2 = Short‐term (−12 months)	0	0	1	1	insufficient	*Not estimable*
3 = Medium‐term (12–36 months)	3	1	1	5	Strong	Moderate
4 = Long‐term (36‐month)	0	2	3	5	Limited	Little
**2.2.5 Administrative controls**		**1**	**1**	**2**		
2 = Short‐term (−12 months)	0	1		1	Insufficient	*Not estimable*
3 = Medium‐term (12–36 months)	0	0	1	1	Insufficient	*Not estimable*
**2.2.7 Enforcement of laws and regulations**	**1**	**2**	**3**	**6**		
4 = Long‐term (36‐month)	1	2	3	6	Moderate	**None to little**
**2.2.8 Social marketing and other approaches**			**1**	**1**		
4 = Long‐term (36‐month)	0		1	1	Insufficient	**Very strong**
**3.0. Multifaceted safety interventions:**						
**3.1 Multifaceted at the individual level**	**1**	**5**	**2**	**8**		
2 = Short‐term (−12 months)	1	2	1	4	Moderate	None
3 = Medium‐term (12–36 months)	0	3	1	4	Limited	**Little to moderate**
**3.2 Multifaceted at the group or org. level**	**2**	**4**	**3**	**9**		
3 = Medium‐term (12–36 months)	2	1	2	5	Moderate	Strong
4 = Long‐term (36‐month)	0	3	1	4	Limited	Moderate
**3.3 Multifaceted across individ. and org. levels**	**5**	**4**	**4**	**13**		
3 = Medium‐term (12–36 months)	3	2	1	6	Strong	None
4 = Long‐term (36‐month)	2	2	3	7	Moderate	Strong
**3.9 Multifaceted safety interventions, other**			1	1		
4 = Long‐term (36‐month)	0	0	1	1	Insufficient	*Not estimable*
**Total**	**26**	**27**	**35**	**88**		

Abbreviations: CBA, controlled before‐and‐after; ITS, interrupted time series; RCT, randomized controlled trial.

## CONTRIBUTION OF AUTHORS

- Content: JDY, HJL, KNI, MTÖ, KBF, PKI, KRA, FWG, and JLU, DZO (protocol stage)
- Systematic review methods: OOL (protocol stage), JDY, JLU, HJL, KRA, UGE
- Statistical analysis: HBA, JDY, OOL (in the first phase)
- Information retrieval: ELB, JDY, and PKI

## DECLARATIONS OF INTEREST

None known.

## SOURCES OF SUPPORT

### INTERNAL SOURCES

The National Research Centre for the Working Environment in Denmark has co‐financed the study.

### EXTERNAL SOURCES

A grant of one million Danish crowns (134.000 €) has been received from the Working Environment Research Fund, Denmark (Grant project number: 48‐2010‐09).

## Supporting information

Supporting information.

## References

[cl21234-bib-0001] INCLUDED STUDIES

[cl21234-bib-0002] INCLUDED RCT STUDIES (*n* = 16)

[cl21234-bib-0003] Adams, J. S. , Raju, R. , Solomon, V. , Samuel, P. , Dutta, A. K. , Rose, J. S. , & Tharyan, P. (2013). Increasing compliance with protective eyewear to reduce ocular injuries in stone‐quarry workers in Tamil Nadu, India: A pragmatic, cluster randomised trial of a single education session versus an enhanced education package delivered over six months. Injury, 44, 118–125. 22075447 10.1016/j.injury.2011.10.001

[cl21234-bib-0004] Cheng, A. S. , & Chan, E. P. (2009). The effect of individual job coaching and use of health threat in a job‐specific occupational health education program on prevention of work‐related musculoskeletal back injury. Journal of Occupational and Environmental Medicine, 51(12), 1413–1421. 19952790 10.1097/JOM.0b013e3181bfb2a8

[cl21234-bib-0005] Daltroy, L. H. , Iversen, M. D. , Larson, M. G. , Lew, R. , Wright, E. , Ryan, J. , Zwerling, C. , Fossel, A. H. , & Liang, M. H. (1997). A controlled trial of an educational program to prevent low back injuries. New England Journal of Medicine, 337(5), 322–328. 9233870 10.1056/NEJM199707313370507

[cl21234-bib-0006] Gadomski, A. , Ackerman, S. , Burdick, P. , & Jenkins, P. (2006). Efficacy of the North American guidelines for children's agricultural tasks in reducing childhood agricultural injuries. American Journal of Public Health, 96(4), 722–727. 16507741 10.2105/AJPH.2003.035428 PMC1470549

[cl21234-bib-0007] Hogg‐Johnson, S. , Robson, L. , Cole, D. C. , Amick, B. C., III , Tompa, E. , Smith, P. M. , van, E. D. , & Mustard, C. (2012). A randomised controlled study to evaluate the effectiveness of targeted occupational health and safety consultation or inspection in Ontario manufacturing workplaces. Occupational and Environmental Medicine, 69(12), 890–900. 22918898 10.1136/oemed-2011-100333

[cl21234-bib-0008] Jensen, S. L. , Kristensen, B. , & Fabrin, K. (1997). Double gloving as self protection in abdominal surgery. European Journal of Surgery, 163(3), 163–167. 9085056

[cl21234-bib-0009] Jinnah, H. A. , Stoneman, Z. , & Rains, G. (2014). Involving fathers in teaching youth about farm tractor seatbelt safety—A randomized control study. Journal of Adolescent Health, 54(3), 255–261. 10.1016/j.jadohealth.2013.10.010 24360924

[cl21234-bib-0010] Kines, P. , Andersen, D. , Andersen, L. P. , Nielsen, K. , & Pedersen, L. (2013). Improving safety in small enterprises through an integrated safety management intervention. Journal of Safety Research, 44, 87–95. 23398709 10.1016/j.jsr.2012.08.022

[cl21234-bib-0011] Morgan, P. J. , Collins, C. E. , Plotnikoff, R. C. , Cook, A. T. , Berthon, B. , Mitchell, S. , & Callister, R. (2012). The impact of a workplace‐based weight loss program on work‐related outcomes in overweight male shift workers. Journal of Occupational and Environmental Medicine, 54(2), 122–127. 22269987 10.1097/JOM.0b013e31824329ab

[cl21234-bib-0012] Peek, A. C. , Casteel, C. , Mineschian, L. , Erickson, R. J. , & Kraus, J. F. (2004). Compliance to a Workplace Violence Prevention Program in small businesses. American Journal of Preventive Medicine, 26, 276–283. 15110053 10.1016/j.amepre.2004.01.004

[cl21234-bib-0013] Prunet, B. , Meaudre, E. , Montcriol, A. , Asencio, Y. , Bordes, J. , Lacroix, G. , & Kaiser, E. (2008). A prospective randomized trial of two safety peripheral intravenous catheters. Anesthesia and Analgesia, 107, 155–158. 18635482 10.1213/ane.0b013e318174df5f

[cl21234-bib-0014] Rasmussen, K. , Carstensen, O. , Lauritsen, J. M. , Glasscock, D. J. , Hansen, O. N. , & Jensen, U. F. (2003). Prevention of farm injuries in Denmark. Scandinavian Journal of Work, Environment and Health, 29(4), 288–296. 10.5271/sjweh.733 12934722

[cl21234-bib-0015] Rautiainen, R. H. , Lange, J. L. , Hodne, C. J. , Schneiders, S. , & Donham, K. J. (2004). Injuries in the Iowa Certified Safe Farm Study. Journal of Agricultural Safety and Health, 10(1), 51–63. 15017805 10.13031/2013.15674

[cl21234-bib-0016] Srikrajang, J. , Pochamarn, C. , Chittreecheur, J. , Apisarnthanarak, A. , & Danchaivijitr, S. (2005). Effectiveness of education and problem solving work group on nursing practices to prevent needlestick and sharp injury. Journal of the Medical Association of Thailand, 88(Suppl. 10), S115–S119. 16850654

[cl21234-bib-0017] van der Molen, H. F. , Zwinderman, K. A. H. , Sluiter, J. K. , & Frings‐Dresen, M. H. W. (2011). Better effect of the use of a needle safety device in combination with an interactive workshop to prevent needle stick injuries. Safety Science, 49(8–9), 1180–1186.

[cl21234-bib-0018] Zohar, D. (2002). Modifying supervisory practices to improve subunit safety: A leadership‐based intervention model. Journal of Applied Psychology, 87(1), 156–163. 11916209 10.1037/0021-9010.87.1.156

[cl21234-bib-0019] INCLUDED CBA STUDIES (*n* = 30)

[cl21234-bib-0020] Banco, L. , Lapidus, G. , Monopoli, J. , & Zavoski, R. (1997). The Safe Teen Work Project: A study to reduce cutting injuries among young and inexperienced workers. American Journal of Industrial Medicine, 31(5), 619–622. 9099365 10.1002/(sici)1097-0274(199705)31:5<619::aid-ajim17>3.0.co;2-1

[cl21234-bib-0021] Bell, J. L. (2002). Changes in logging injury rates associated with use of feller‐bunchers in West Virginia. Journal of Safety Research, 33(4), 463–471. 12429103 10.1016/s0022-4375(02)00048-8

[cl21234-bib-0022] Black, T. R. , Shah, S. M. , Busch, A. J. , Metcalfe, J. , & Lim, H. J. (2011). Effect of transfer, lifting, and repositioning (TLR) injury prevention program on musculoskeletal injury among direct care workers. Journal of Occupational and Environmental Hygiene, 8(4), 226–235. 21400388 10.1080/15459624.2011.564110

[cl21234-bib-0023] Carrivick, P. J. , Lee, A. H. , & Yau, K. K. (2002). Effectiveness of a workplace risk assessment team in reducing the rate, cost, and duration of occupational injury. Journal of Occupational and Environmental Medicine, 44(2), 155–159. 11851216 10.1097/00043764-200202000-00010

[cl21234-bib-0024] Evanoff, B. A. , Bohr, P. C. , & Wolf, L. D. (1999). Effects of a participatory ergonomics team among hospital orderlies. American Journal of Industrial Medicine, 35(4), 358–365. 10086212 10.1002/(sici)1097-0274(199904)35:4<358::aid-ajim6>3.0.co;2-r

[cl21234-bib-0025] Foley, M. , Fan, Z. J. , Rauser, E. , & Silverstein, B. (2012). The impact of regulatory enforcement and consultation visits on workers' compensation claims incidence rates and costs, 1999–2008. American Journal of Industrial Medicine, 55(11), 976–990. 22715086 10.1002/ajim.22084

[cl21234-bib-0026] Forst, L. , Lacey, S. , Chen, H. Y. , Jimenez, R. , Bauer, S. , Skinner, S. , Alvarado, R. , Nickels, L. , Zanoni, J. , Petrea, R. , & Conroy, L. (2004). Effectiveness of community health workers for promoting use of safety eyewear by Latino farm workers. American Journal of Industrial Medicine, 46(6), 607–613. 15551366 10.1002/ajim.20103

[cl21234-bib-0027] Gregersen, N. P. , Brehmer, B. , & Moren, B. (1996). Road safety improvement in large companies. An experimental comparison of different measures. Accident Analysis and Prevention, 28(3), 297–306. 8799433 10.1016/0001-4575(95)00060-7

[cl21234-bib-0028] Grimmond, T. , Bylund, S. , Anglea, C. , Beeke, L. , Callahan, A. , Christiansen, E. , Flewelling, K. , McIntosh, K. , Richter, K. , & Vitale, M. (2010). Sharps injury reduction using a sharps container with enhanced engineering: A 28 hospital nonrandomized intervention and cohort study. American Journal of Infection Control, 38(10), 799–805. 21093697 10.1016/j.ajic.2010.06.010

[cl21234-bib-0029] Harms‐Ringdahl, L. (1987). Safety analysis in design—Evaluation of a case study. Accident Analysis and Prevention, 19(4), 305–317. 3651203 10.1016/0001-4575(87)90064-9

[cl21234-bib-0030] Haviland, A. M. , Burns, R. M. , Gray, W. B. , Ruder, T. , & Mendeloff, J. (2012). A new estimate of the impact of OSHA inspections on manufacturing injury rates, 1998–2005. American Journal of Industrial Medicine, 55(11), 964–975. 22566135 10.1002/ajim.22062

[cl21234-bib-0031] Hilyer, J. C. , Brown, K. C. , Sirles, A. T. , & Peoples, L. (1990). A flexibility intervention to reduce the incidence and severity of joint injuries among municipal firefighters. Journal of Occupational Medicine, 32(7), 631–637. 2391578 10.1097/00043764-199007000-00015

[cl21234-bib-0291] Johnson, A. (2011). Examining the foundation‐Were Herbert William Heinrich's theories valid, and do they still matter? SH‐Safety and Health‐National Safety Council, 184(4), 62.

[cl21234-bib-0032] Johnson, O. E. , & Owoaje, E. T. (2012). Effect of health education on the riding habits of commercial motorcyclists in Uyo, southern Nigeria. West African Journal of Medicine, 31(1), 39–46. 23115095

[cl21234-bib-0033] Kim, P. , Hayden, J. A. , & Mior, S. A. (2004). The cost‐effectiveness of a back education program for firefighters: A case study. Journal of the Canadian Chiropractic Association, 48(1), 13–19. 17549215 PMC1840037

[cl21234-bib-0034] Kines, P. , Andersen, L. P. , Spangenberg, S. , Mikkelsen, K. L. , Dyreborg, J. , & Zohar, D. (2010). Improving construction site safety through leader‐based verbal safety communication. Journal of safety research, 41(5), 399–406. 21059457 10.1016/j.jsr.2010.06.005

[cl21234-bib-0035] Knapik, J. J. , Bullock, S. H. , Canada, S. , Toney, E. , Wells, J. D. , Hoedebecke, E. , & Jones, B. H. (2004). Influence of an injury reduction program on injury and fitness outcomes among soldiers. Injury Prevention, 10(1), 37–42. 14760025 10.1136/ip.2003.002808 PMC1756537

[cl21234-bib-0036] Knapik, J. J. , Hauret, K. G. , Arnold, S. , Canham‐Chervak, M. , Mansfield, A. J. , Hoedebecke, E. L. , & McMillian, D. (2003). Injury and fitness outcomes during implementation of physical readiness training. International Journal of Sports Medicine, 24(5), 372–381. 12868050 10.1055/s-2003-40710

[cl21234-bib-0037] Lanoie, P. (1992). The impact of occupational safety and health regulation on the risk of workplace accidents: Quebec, 1983–87. Journal of Human Resources, 27(4), 643–660.

[cl21234-bib-0038] Levine, D. I. , Toffel, M. W. , & Johnson, M. S. (2012). Randomized government safety inspections reduce worker injuries with no detectable job loss. Science, 336(6083), 907–911. 22605775 10.1126/science.1215191

[cl21234-bib-0039] Lopez‐Ruiz, M. , Martinez, J. M. , Gil, J. M. , Boix, P. , Garcia, A. M. , Rodrigo, F. , Moreno, A. , & Benavides, F. G. (2013). Evaluation of the effectiveness of occupational injury prevention programs at the company level. Safety Science, 51(1), 250–256.

[cl21234-bib-0040] Mattila, M. , & Hyoedynamaa, M. (1988). Promoting job safety in building: An experiment on the behavious analysis approach. Journal of Occupational Accidents, 9(4), 255–267.

[cl21234-bib-0041] Mehrdad, R. , Meshki, M. , & Pouryagub, G. (2013). Effects of training course on occupational exposure to bloodborne pathogens: A controlled interventional study. International Journal of Preventive Medicine, 4(11), 1136–1142. PMC3883246 24404356

[cl21234-bib-0042] Parker, D. L. , Brosseau, L. M. , Samant, Y. , Xi, M. , Pan, W. , & Haugan, D. (2009). A randomized, controlled intervention of machine guarding and related safety programs in small metal‐fabrication businesses. Public Health Reports, 124(Suppl. 1), 90–100. 10.1177/00333549091244S111 PMC2708660 19618811

[cl21234-bib-0043] Peate, W. F. , Bates, G. , Lunda, K. , Francis, S. , & Bellamy, K. (2007). Core strength: A new model for injury prediction and prevention. Journal of Occupational Medicine and Toxicology, 2, 3. 17428333 10.1186/1745-6673-2-3 PMC1865378

[cl21234-bib-0044] Quintana, R. (1999). A task‐delineated safety approach for slip, trip and fall hazards. Safety Science, 33(1‐2), 31–45.

[cl21234-bib-0045] Rasmussen, K. , Glasscock, D. J. , Hansen, O. N. , Carstensen, O. , Jepsen, J. F. , & Nielsen, K. J. (2006). Worker participation in change processes in a Danish industrial setting. American Journal of Industrial Medicine, 49(9), 767–779. 16804911 10.1002/ajim.20350

[cl21234-bib-0046] Ray, P. S. , Bishop, P. A. , & Wang, M. Q. (1997). Efficacy of the components of a behavioral safety program. International Journal of Industrial Ergonomics, 19(1), 19–29.

[cl21234-bib-0047] Santaweesuk, S. , Chapman, R. S. , & Siriwong, W. (2014). Effects of an injury and illness prevention program on occupational safety behaviors among rice farmers in Nakhon Nayok Province, Thailand. Risk Management and Healthcare Policy, 7, 51–60. 24634590 10.2147/RMHP.S55810 PMC3953033

[cl21234-bib-0048] Valls, V. , Lozano, M. S. , Yanez, R. , Martinez, M. J. , Pascual, F. , Lloret, J. , & Ruiz, J. A. (2007). Use of safety devices and the prevention of percutaneous injuries among healthcare workers [corrected] [published erratum appears in INFECT CONTROL HOSP EPIDEMIOL 2008 Mar;29(3):288]. Infection Control & Hospital Epidemiology, 28(12), 1352–1360. 17994515 10.1086/523275

[cl21234-bib-0049] Wang, H. H. , Fennie, K. , He, G. P. , Burgess, J. , & Williams, A. B. (2003). A training programme for prevention of occupational exposure to bloodborne pathogens: Impact on knowledge, behaviour and incidence of needle stick injuries among student nurses in Changsha, People's Republic of China. Journal of Advanced Nursing, 41(2), 187–194. 12519278 10.1046/j.1365-2648.2003.02519.x

[cl21234-bib-0050] INCLUDED STUDIES WITH SERIAL MEASURES (ITS) (*n* = 54)

[cl21234-bib-0051] Alamgir, H. , Yu, S. , Fast, C. , Hennessy, S. , Kidd, C. , & Yassi, A. (2008). Efficiency of overhead ceiling lifts in reducing musculoskeletal injury among carers working in long‐term care institutions. Injury(), 39(5), 570–577. 18377908 10.1016/j.injury.2007.11.420

[cl21234-bib-0052] Bell, J. L. , & Grushecky, S. T. (2006). Evaluating the effectiveness of a logger safety training program. Journal of Safety Research, 37(1), 53–61. 16516237 10.1016/j.jsr.2005.10.019

[cl21234-bib-0053] Bell, J. L. , Collins, J. W. , Wolf, L. , Gronqvist, R. , Chiou, S. , Chang, W. R. , Sorock, G. S. , Courtney, T. K. , Lombardi, D. A. , & Evanoff, B. (2008). Evaluation of a comprehensive slip, trip and fall prevention programme for hospital employees. Ergonomics, 51(12), 1906–1925. 18932056 10.1080/00140130802248092

[cl21234-bib-0054] Bena, A. , Berchialla, P. , Coffano, M. E. , Debernardi, M. L. , & Icardi, L. G. (2009). Effectiveness of the training program for workers at construction sites of the high‐speed railway line between Torino and Novara: Impact on injury rates. American Journal of Industrial Medicine, 52(12), 965–972. 19877110 10.1002/ajim.20770

[cl21234-bib-0055] Benavides, F. G. , Garcia, A. M. , Lopez‐Ruiz, M. , Gil, J. , Boix, P. , Martinez, J. M. , & Rodrigo, F. (2009). Effectiveness of occupational injury prevention policies in Spain. Public Health Reports, 124(Suppl. 1), 180–187. 19618820 10.1177/00333549091244S120 PMC2708669

[cl21234-bib-0056] Birnbaum, D. (1993). Needlestick injuries among critical care nurses before and after adoption of universal precautions or body substance isolation. Journal of Healthcare Materiel Management, 11(8), 38–42. 10128145

[cl21234-bib-0057] Briggs, C. S. , Sundt, J. L. , & Castellano, T. C. (2003). The effect of supermaximum security prisons on aggregate levels of institutional violence. Criminology, 41(4), 1341–1376.

[cl21234-bib-0058] Bull, N. (2007). Mandatory use of eye protection prevents eye injuries in the metal industry. Occupational Medicine, 57(8), 605–606. 17675660 10.1093/occmed/kqm083

[cl21234-bib-0059] Bulzacchelli, M. T. , Vernick, J. S. , Webster, D. W. , & Lees, P. S. (2007). Effects of the Occupational Safety and Health Administration's control of hazardous energy (lockout/tagout) standard on rates of machinery‐related fatal occupational injury. Injury Prevention, 13(5), 334–338. 17916891 10.1136/ip.2007.015677 PMC2610620

[cl21234-bib-0060] Casteel, C. , Peek‐Asa, C. , Howard, J. , & Kraus, J. F. (2004). Effectiveness of crime prevention through environmental design in reducing criminal activity in liquor stores: A pilot study. Journal of Occupational and Environmental Medicine, 46(5), 450–458. 15167393 10.1097/01.jom.0000126025.14847.b1

[cl21234-bib-0061] Casteel, C. , Peek‐Asa, C. , Nocera, M. , Smith, J. B. , Blando, J. , Goldmacher, S. , O'Hagan, E. , Valiante, D. , & Harrison, R. (2009). Hospital employee assault rates before and after enactment of the California Hospital Safety and Security Act. Annals of Epidemiology, 19(2), 125–133. 19185807 10.1016/j.annepidem.2008.10.009

[cl21234-bib-0292] Chapman, L. J. , Brunette, C. M. , Karsh, B. T. , Taveira, A. D. , & Josefsson, K. G. (2011). A 4‐year intervention to increase adoption of safer dairy farming work practices. American Journal of Industrial Medicine, 54(3), 232–243. 21298698 10.1002/ajim.20920

[cl21234-bib-0062] Chapman, L. J. , Brunette, C. M. , & Taveira, A. D. (2013). A seven‐year intervention to diffuse economic innovations with safety benefits to Wisconsin dairy farmers. Journal of Agricultural Safety and Health, 19(3), 147–162. 24400420 10.13031/jash.19.9944

[cl21234-bib-0063] Chhokar, R. , Engst, C. , Miller, A. , Robinson, D. , Tate, R. B. , & Yassi, A. (2005). The three‐year economic benefits of a ceiling lift intervention aimed to reduce healthcare worker injuries. Applied Ergonomics, 36(2), 223–229. 15694077 10.1016/j.apergo.2004.10.008

[cl21234-bib-0064] Cooper, M. D. , Phillips, R. A. , Sutherland, V. J. , & Makin, P. J. (1994). Reducing accidents using goal setting and feedback: A field study. Journal of Occupational and Organizational Psychology, 67(3), 219–240.

[cl21234-bib-0065] Cunningham, T. R. , & Austin, J. (2007). Using goal setting, task clarification, and feedback to increase the use of the hands‐free technique by hospital operating room staff. Journal of Applied Behavior Analysis, 40(4), 673–677. 18189098 10.1901/jaba.2007.673-677 PMC2078574

[cl21234-bib-0066] Derr, J. , Forst, L. , Chen, H. Y. , & Conroy, L. (2001). Fatal falls in the US construction industry, 1990 to 1999. Journal of Occupational and Environmental Medicine, 43(10), 853–860. 11665454 10.1097/00043764-200110000-00004

[cl21234-bib-0067] Farina, E. , Bena, A. , Pasqualini, O. , & Costa, G. (2013). Are regulations effective in reducing construction injuries? An analysis of the Italian context. Occupational and Environmental Medicine, 70(9), 611–616. 23503417 10.1136/oemed-2012-101087

[cl21234-bib-0068] Fellner, D. J. , & Sulzer‐Azaroff, B. (1984). Increasing industrial safety practices and conditions through posted feedback. Journal of Safety Research, 15(1), 7–21.

[cl21234-bib-0069] Fujishiro, K. , Weaver, J. L. , Heaney, C. A. , Hamrick, C. A. , & Marras, W. S. (2005). The effect of ergonomic interventions in healthcare facilities on musculoskeletal disorders. American Journal of Industrial Medicine, 48(5), 338–347. 16254947 10.1002/ajim.20225

[cl21234-bib-0070] Garg, A. (1999). Long‐term effectiveness of “zero‐lift program” in seven nursing homes and one hospital. NIOSH.

[cl21234-bib-0071] Gershon, R. R. , Pearse, L. , Grimes, M. , Flanagan, P. A. , & Vlahov, D. (1999). The impact of multifocused interventions on sharps injury rates at an acute‐care hospital. Infection Control and Hospital Epidemiology, 20(12), 806–811. 10614603 10.1086/501588

[cl21234-bib-0072] Kuehl, K. S. , Elliot, D. L. , Goldberg, L. , Moe, E. L. , Perrier, E. , & Smith, J. (2013). Economic benefit of the PHLAME wellness programme on firefighter injury. Occupational Medicine, 63(3), 203–209. 23416849 10.1093/occmed/kqs232 PMC3617369

[cl21234-bib-0073] Lawrence, L. W. , Delclos, G. L. , Felknor, S. A. , Johnson, P. C. , Frankowski, R. F. , Cooper, S. P. , & Davidson, A. (1997). The effectiveness of a needleless intravenous connection system: An assessment by injury rate and user satisfaction. Infection Control & Hospital Epidemiology, 18(3), 175–182. 9090545 10.1086/647583

[cl21234-bib-0074] Lipscomb, H. J. , Li, L. , & Dement, J. (2003). Work‐related falls among union carpenters in Washington State before and after the Vertical Fall Arrest Standard. American Journal of Industrial Medicine, 44(2), 157–165. 12874848 10.1002/ajim.10254

[cl21234-bib-0075] Lipscomb, H. J. , Nolan, J. , Patterson, D. , & Dement, J. M. (2010). Continued progress in the prevention of nail gun injuries among apprentice carpenters: What will it take to see wider spread injury reductions? Journal of safety research, 41(3), 241–245. 20630275 10.1016/j.jsr.2010.01.005

[cl21234-bib-0076] Lopez‐Ruiz, M. , Martinez, J. M. , Perez, K. , Novoa, A. M. , Tobias, A. , & Benavides, F. G. (2014). Impact of road safety interventions on traffic‐related occupational injuries in Spain, 2004–2010. Accident Analysis and Prevention, 66, 114–119. 24531113 10.1016/j.aap.2014.01.012

[cl21234-bib-0077] Mancini, G. , Baldasseroni, A. , Laffi, G. , Curti, S. , Mattioli, S. , & Violante, F. S. (2005). Prevention of work related eye injuries: Long term assessment of the effectiveness of a multicomponent intervention among metal workers. Occupational and Environmental Medicine, 62(12), 830–835. 16299090 10.1136/oem.2004.019570 PMC1740928

[cl21234-bib-0078] Marlenga, B. , Doty, B. C. , Berg, R. L. , & Linneman, J. G. (2006). Evaluation of a policy to reduce youth tractor crashes on public roads. Injury Prevention, 12(1), 46–51. 16502564 10.1136/ip.2005.008771 PMC2563500

[cl21234-bib-0079] Martin, P. J. , Harvey, J. T. , Culvenor, J. F. , & Payne, W. R. (2009). Effect of a nurse back injury prevention intervention on the rate of injury compensation claims. Journal of safety research, 40(1), 13–19. 19285581 10.1016/j.jsr.2008.10.013

[cl21234-bib-0080] Menendez, C. C. , Castillo, D. , Rosenman, K. , Harrison, R. , & Hendricks, S. (2012). Evaluation of a nationally funded state‐based programme to reduce fatal occupational injuries. Occupational and Environmental Medicine, 69(11), 810–814. 22864251 10.1136/oemed-2011-100213 PMC4681488

[cl21234-bib-0081] Miller, T. R. , Zaloshnja, E. , & Spicer, R. S. (2007). Effectiveness and benefit‐cost of peer‐based workplace substance abuse prevention coupled with random testing. Accident Analysis and Prevention, 39(3), 565–573. 17125723 10.1016/j.aap.2006.10.001

[cl21234-bib-0082] Mode, N. A. , O'Connor, M. B. , Conway, G. A. , & Hill, R. D. (2012). A multifaceted public health approach to statewide aviation safety. American Journal of Industrial Medicine, 55(2), 176–186. 22170605 10.1002/ajim.21993

[cl21234-bib-0083] Monforton, C. , & Windsor, R. (2010). An impact evaluation of a federal mine safety training regulation on injury rates among US stone, sand, and gravel mine workers: An interrupted time‐series analysis. American Journal of Public Health, 100(7), 1334–1340. 20466960 10.2105/AJPH.2009.178301 PMC2882415

[cl21234-bib-0084] Mujuru, P. , Helmkamp, J. C. , Mutambudzi, M. , Hu, W. , & Bell, J. L. (2009). Evaluating the impact of an intervention to reduce injuries among loggers in West Virginia, 1999–2007. Journal of Agricultural Safety and Health, 15(1), 75–88. 19266885 10.13031/2013.25416

[cl21234-bib-0085] Park, R. M. , Bushnell, P. T. , Bailer, A. J. , Collins, J. W. , & Stayner, L. T. (2009). Impact of publicly sponsored interventions on musculoskeletal injury claims in nursing homes. American Journal of Industrial Medicine, 52(9), 683–697. 19670260 10.1002/ajim.20731

[cl21234-bib-0086] Passfield, J. , Marshall, E. , & Adams, R. (2003). “No lift” patient handling policy implementation and staff injury rates in a public hospital. Journal of Occupational Health and Safety—Australia and New Zealand, 19(1), 73–85.

[cl21234-bib-0087] Phillips, E. K. , Conaway, M. R. , & Jagger, J. C. (2012). Percutaneous injuries before and after the needlestick safety and prevention act. New England Journal of Medicine, 366(7), 670–671. 22335760 10.1056/NEJMc1110979

[cl21234-bib-0088] Porru, S. , Calza, S. , & Arici, C. (2011). An effectiveness evaluation of a multifaceted preventive intervention on occupational injuries in foundries: A 13‐year follow‐up study with interrupted time series analysis. International Archives of Occupational and Environmental Health, 84(8), 867–876. 21499873 10.1007/s00420-011-0638-3

[cl21234-bib-0089] Prezant, D. J. , Kelly, K. J. , Malley, K. S. , Karwa, M. L. , McLaughlin, M. T. , Hirschorn, R. , & Brown, A. (1999). Impact of a modern firefighting protective uniform on the incidence and severity of burn injuries in New York City firefighters. Journal of Occupational and Environmental Medicine, 41(6), 469–479. 10390698 10.1097/00043764-199906000-00013

[cl21234-bib-0090] Rautiainen, R. H. , Ledolter, J. , Sprince, N. L. , Donham, K. J. , Burmeister, L. F. , Ohsfeldt, R. , Reynolds, S. J. , Phillips, K. , & Zwerling, C. (2005). Effects of premium discount on workers' compensation claims in agriculture in Finland. American Journal of Industrial Medicine, 48(2), 100–109. 16032738 10.1002/ajim.20192

[cl21234-bib-0091] Reddy, S. G. , & Emery, R. J. (2001). Assessing the effect of long‐term availability of engineering controls on needlestick injuries among health care workers: A 3‐year preimplementation and postimplementation comparison. American Journal of Infection Control, 29(6), 425–427. 11743491 10.1067/mic.2001.118404

[cl21234-bib-0092] Rogues, A. , Verdun‐Esquer, C. , Buisson‐Valles, I. , Laville, M. , Lasheras, A. , Sarrat, A. , Beaudelle, H. , Brochard, P. , & Gachie, J. (2004). Impact of safety devices for preventing percutaneous injuries related to phlebotomy procedures in health care workers. American Journal of Infection Control, 32(8), 441–444. 15573049 10.1016/j.ajic.2004.07.006

[cl21234-bib-0093] Saari, J. , & Näsänen, M. (1989). The effect of positive feedback on industrial housekeeping and accidents; A long‐term study at a shipyard. International Journal of Industrial Ergonomics, 4(3), 201–211.

[cl21234-bib-0094] Schoenfisch, A. L. , Lipscomb, H. J. , Pompeii, L. A. , Myers, D. J. , & Dement, J. M. (2013). Musculoskeletal injuries among hospital patient care staff before and after implementation of patient lift and transfer equipment. Scandinavian Journal of Work, Environment and Health, 39(1), 27–36. 10.5271/sjweh.3288 22396049

[cl21234-bib-0095] Smollen, P. (2004). Evaluation of a programme designed to reduce occupational exposures from steel‐winged butterfly needles in the clinical setting. Australian Infection Control, 9(2), 47–55.

[cl21234-bib-0096] Sossai, D. , Puro, V. , Chiappatoli, L. , Dagnino, G. , Odone, B. , Polimeri, A. , Ruzza, L. , Palombo, P. , Fuscoe, M. S. , & Scognamiglio, P. (2010). Using an intravenous catheter system to prevent needlestick injury. Nursing Standard, 24(29), 42–46. 10.7748/ns2010.03.24.29.42.c7628 20426370

[cl21234-bib-0097] Spangenberg, S. , Mikkelsen, K. L. , Kines, P. , Dyreborg, J. , & Baarts, C. (2002). The construction of the Øresund Link between Denmark and Sweden: The effect of a multifaceted safety campaign. Safety Science, 40(5), 457–465.

[cl21234-bib-0098] Sulzer‐Azaroff, B. , & de Santamaria, M. C. (1980). Industrial safety hazard reduction through performance feedback. Journal of Applied Behavior Analysis, 13(2), 287–295. 7380753 10.1901/jaba.1980.13-287 PMC1308132

[cl21234-bib-0099] Sulzer‐Azaroff, B. , Loafman, B. , Merante, R. J. , & Hlavacek, A. C. (1990). Improving occupational safety in a large industrial plant. Journal of Organizational Behavior Management, 11(1), 99–120.

[cl21234-bib-0100] Suruda, A. , Whitaker, B. , Bloswick, D. , Philips, P. , & Sesek, R. (2002). Impact of the OSHA trench and excavation standard on fatal injury in the construction industry. Journal of Occupational and Environmental Medicine, 44(10), 902–905. 12391768 10.1097/00043764-200210000-00007

[cl21234-bib-0101] Whitby, M. , McLaws, M. L. , & Slater, K. (2008). Needlestick injuries in a major teaching hospital: The worthwhile effect of hospital‐wide replacement of conventional hollow‐bore needles. American Journal of Infection Control, 36(3), 180–186. 18371513 10.1016/j.ajic.2007.07.009

[cl21234-bib-0102] Wickizer, T. M. , Kopjar, B. , Franklin, G. , & Joesch, J. (2004). Do drug‐free workplace programs prevent occupational injuries? Evidence from Washington State. Health Services Research, 39(1), 91–110. 14965079 10.1111/j.1475-6773.2004.00217.x PMC1360996

[cl21234-bib-0103] Zafar, A. B. , Butler, R. C. , Podgorny, J. M. , Mennonna, P. A. , Gaydos, L. A. , & Sandiford, J. A. (1997). Effect of a comprehensive program to reduce needlestick injuries. Infection Control and Hospital Epidemiology, 18(10), 712–715. 9350465 10.1086/647518

[cl21234-bib-0104] Zohar, D. , & Luria, G. (2003). The use of supervisory practices as leverage to improve safety behavior: A cross‐level intervention model. Journal of safety research, 34(5), 567–577. 14733991 10.1016/j.jsr.2003.05.006

[cl21234-bib-0105] EXCLUDED STUDIES

[cl21234-bib-0106] SEE REASONS FOR EXCLUSION IN TABLE 9.2

[cl21234-bib-0107] Azar‐Cavanagh, M. , Burdt, P. , & Green‐McKenzie, J. (2007). Effect of the introduction of an engineered sharps injury prevention device on the percutaneous injury rate in healthcare workers. Infection Control and Hospital Epidemiology, 28, 165–170. 17265397 10.1086/511699

[cl21234-bib-0108] Childs, J. D. , Teyhen, D. S. , Casey, P. R. , McCoy‐Singh, K. A. , Feldtmann, A. W. , Wright, A. C. , Dugan, J. L. , Wu, S. S. , & George, S. Z. (2010). Effects of traditional sit‐up training versus core stabilization exercises on short‐term musculoskeletal injuries in US Army soldiers: A cluster randomized trial. Physical Therapy, 90, 1404–1412. 20651013 10.2522/ptj.20090389

[cl21234-bib-0109] Daltroy, L. H. , Iversen, M. D. , Larson, M. G. , Ryan, J. , Zwerling, C. , Fossel, A. H. , & Liang, M. H. (1993). Teaching and social support—Effects on knowledge, attitudes, and behaviors to prevent low‐back injuries in industry. Health Education Quarterly, 20, 43–62. 8444625 10.1177/109019819302000106

[cl21234-bib-0111] Donaldson, C. S. , Stanger, L. M. , Donaldson, M. W. , & Cram, J. (1993). A randomized crossover investigation of a back pain and disability prevention program: Possible mechanisms of change. Journal of Occupational Rehabilitation, 3, 83–94. 24243228 10.1007/BF01078161

[cl21234-bib-0112] Hall, N. , Brown, M. , & Constantinou, M. (2013). Does a structured neuromuscular training program reduce the incidence of lower limb injuries in NZDF army recruits? (p. e17). Elsevier Ltd.

[cl21234-bib-0113] Helitzer, D. L. , Hathorn, G. , Benally, J. , & Ortega, C. (2014). Culturally relevant model program to prevent and reduce agricultural injuries. Journal of Agricultural Safety and Health, 20, 175–198. 25174150 10.13031/jash.20.10333

[cl21234-bib-0293] Jagger, J. , & Bentley, M. B. (1997). Injuries from vascular access devices: high risk and preventable. Collaborative EPINet Surveillance Group. Journal of Intravenous Nursing, 20(6 Suppl), S33–S39. 9423399

[cl21234-bib-0114] Lavender, S. A. , Lorenz, E. P. , & Andersson, G. B. (2007). Can a new behaviorally oriented training process to improve lifting technique prevent occupationally related back injuries due to lifting? Spine, 32, 487–494. 17304142 10.1097/01.brs.0000255203.96898.f2

[cl21234-bib-0115] Miller, A. , Engst, C. , Tate, R. B. , & Yassi, A. (2006). Evaluation of the effectiveness of portable ceiling lifts in a new long‐term care facility. Applied Ergonomics, 37, 377–385. 16380072 10.1016/j.apergo.2005.05.012

[cl21234-bib-0116] Mullen, J. , & Kelloway, E. K. (2009). Safety leadership: A longitudinal study of the effects of transformational leadership on safety outcomes. Journal of Occupational and Organizational Psychology, 82(2), 253–272.

[cl21234-bib-0117] Nielsen, K. J. (2014). Improving safety culture through the health and safety organization: A case study. Journal of Safety Research, 48, 7–17. 24529086 10.1016/j.jsr.2013.10.003

[cl21234-bib-0118] Reddell, C. R. , Congleton, J. J. , Dale, H. R. , & Montgomery, J. F. (1992). An evaluation of a weightlifting belt and back injury prevention training class for airline baggage handlers. Applied Ergonomics, 23, 319–329. 15676878 10.1016/0003-6870(92)90293-5

[cl21234-bib-0119] Shaw, W. S. , Robertson, M. M. , McLellan, R. K. , Verma, S. , & Pransky, G. (2006). A controlled case study of supervisor training to optimize response to injury in the food processing industry. Work, 26, 107–114. 16477102

[cl21234-bib-0120] van der Molen, H. F. , Zwinderman, K. A. , Sluiter, J. K. , & Frings‐Dresen, M. H. (2012). Interventions to prevent needle stick injuries among health care workers. Work, 41(Suppl. 1), 1969–1971. 22317004 10.3233/WOR-2012-0416-1969

[cl21234-bib-0121] Warburton, A. L. , & Shepherd, J. P. (2000). Effectiveness of toughened glassware in terms of reducing injury in bars: A randomised controlled trial. Injury Prevention, 6, 36–40. 10728540 10.1136/ip.6.1.36 PMC1730594

[cl21234-bib-0122] STUDIES AWAITING CLASSIFICATION

[cl21234-bib-0123] SEE REASONS FOR AWAITING CLASSIFICATION IN TABLE 9.3

[cl21234-bib-0124] Bena, A. , Berchialla, P. , Coffano, E. , Debernardi, M. , Icardi, L. , & Dettoni, L. (2009). [Effectiveness of a training programme in reducing occupational injuries: The Turin‐Novara high‐speed railway line experience]. Medicina del Lavoro, 100, 295–298. 19764188

[cl21234-bib-0125] Benavides, F. G. , Rodrigo, F. , García, A. M. , Lopez‐Ruiz, M. , Gil, J. , Boix, P. , & Martínez, J. M. (2007). [Evaluation of the effectiveness of preventive activities (Strategic Action Plans) on the incidence of non‐fatal traumatic occupational injuries leading to disabilities in Spain (1994–2004)]. Revista Espanola de Salud Publica, 81, 615–624. 18347745 10.1590/s1135-57272007000600005

[cl21234-bib-0126] Fekieta, R. (2008). Pre and post evaluation of a participatory ergonomics approach to promote usage of patient lifting equipment (p. 5151). ProQuest Information & Learning.

[cl21234-bib-0127] Hernández Navarrete, M. J. , Lapresta Moros, C. , del Villar Belzunce, A. , Gaite Villagra, A. , Ballabriga Clavería, J. , Solano Bernad, V. M. , & Franco Sorolla, J. M. (2010). [Efficiency and cost‐effectiveness for a safety device]. Revista de Enfermeria, 33, 48–53. 20201200

[cl21234-bib-0128] Lanoie, P. , & Streliski, D. (1996). The impact of workplace health and safety policies on accident risk in Quebec: New results. Relations Industrielles‐Industrial Relations, 51, 778–801.

[cl21234-bib-0129] Lim, H. , Black, T. , Sarker, S. , & Metcalfe, J. (2011). Long‐term effect of multifactor transfer, lifting, and repositioning intervention program among health care workers (p. S260). Lippincott Williams and Wilkins.

[cl21234-bib-0130] Lopez‐Rojas, P. , Salinas‐Tovar, S. , Marin‐Cotonieto, I. A. , Mendez‐Vargas, M. M. , Quezada‐Ortega, R. M. , & Martinez‐Ramirez, E. (2010). [The influence of preventive programs on laboral injuries]. Revista medica del Instituto Mexicano del Seguro Social, 48, 353–360. 21194503

[cl21234-bib-0131] Porru, S. , Arici, C. , Calza, S. , & Campagna, M. (2009). [Prevention of occupational injuries in foundries: Multidisciplinary intervention and evaluation of effectiveness]. Medicina del Lavoro, 100, 290–294. 19764187

[cl21234-bib-0132] Urban, A. , Murru, C. , & Marraccini, G. (2012). [Workplace injury prevention in airport: Intervention model]. Giornale Italiano di Medicina del Lavoro ed Ergonomia, 34, 759–761. 23405772

[cl21234-bib-0133] Markovic‐Denic, L. , Mihajlovic, B. , Cemerlic‐Adjic, N. , Pavlovic, K. , & Nicin, S. (2011). The effect of training program to reduce needlestick injuries. BMC proceedings, 5(Suppl. 6), P217.

[cl21234-bib-0134] ONGOING STUDIES

[cl21234-bib-0135] NO ONGOING STUDIES IDENTIFIED

[cl21234-bib-0136] ADDITIONAL REFERENCES

[cl21234-bib-0137] Ajzen, I. (2012). The theory of planned behavior, Handbook of theories of social psychology (pp. 438–459). Sage.

[cl21234-bib-0138] Ajzen, I. , & Fishbein, M. (1980). Understanding attitudes and predicting social behavior. Prentice‐Hall.

[cl21234-bib-0139] Andrée Löfholm, C. , Brännström, L. , Olsson, M. , & Hansson, K. (2013). Treatment‐as‐usual in effectiveness studies: What is it and does it matter? International Journal of Social Welfare, 2013 22, 25–34.

[cl21234-bib-0140] Armitage, C. J. , & Conner, M. (2001). Efficacy of the theory of planned behaviour: A meta‐analytic review. British Journal of Social Psychology, 40(4), 471–499. 11795063 10.1348/014466601164939

[cl21234-bib-0141] Bandura, A. (1971). Social learning theory. General Learning Press.

[cl21234-bib-0142] Beus, J. M. , Payne, S. C. , Bergman, M. E. , & Arthur, W., Jr. (2010). Safety climate and injuries: An examination of theoretical and empirical relationships. Journal of Applied Psychology, 95, 713–727. 20604591 10.1037/a0019164

[cl21234-bib-0143] Borenstein, M. , Hedges, L. V. , Higgins, J. P. T. , & Rothstein, H. R. (2009). Introduction to meta‐analysis. John Wiley & Sons.

[cl21234-bib-0144] Bullock, S. H. , Jones, B. H. , Gilchrist, J. , & Marshall, S. W. (2010). Prevention of physical training–related injuries: Recommendations for the military and other active populations based on expedited systematic reviews. American Journal of Preventive Medicine, 38(1), S156–S181. 20117590 10.1016/j.amepre.2009.10.023

[cl21234-bib-0145] Burke, M. J. , Sarpy, S. A. , Smith‐Crowe, K. , Chan‐Serafin, S. , Salvador, R. O. , & Islam, G. (2006). Relative effectiveness of worker safety and health training methods. American Journal of Public Health, 96(2), 315–324. 16380566 10.2105/AJPH.2004.059840 PMC1470479

[cl21234-bib-0146] Cameron, I. , & Duff, R. (2007). A critical review of safety initiatives using goal setting and feedback. Construction Management and Economics, 25, 495–508.

[cl21234-bib-0294] Cashman, C. M. , Ruotsalainen, J. H. , Greiner, B. A. , Beirne, P. V. , & Verbeek, J. H. (2009). Alcohol and drug screening of occupational drivers for preventing injury. *Cochrane Database of Systematic Reviews*, (2). 10.1002/14651858.CD006566.pub2 PMC7387791 19370641

[cl21234-bib-0147] Castillo, D. N. , Pizatella, T. J. , & Stout, N. A. (2006). In Levy, B. S. , Wegman, D. H. , Baron, S. L. & Sokas, R. K. , Chapter 22: Injuries in occupational and environmental health. Recognizing and preventing disease and injury. Lippincott, Williams, and Wilkins.

[cl21234-bib-0148] Chambers, A. , Mustard, C. A. , Breslin, C. , Holness, L. , & Nichol, K. (2013). Evaluating the implementation of health and safety innovations under a regulatory context: A collective case study of Ontario's safer needle regulation. Implementation Science, 8(1), 9. 23339295 10.1186/1748-5908-8-9 PMC3556097

[cl21234-bib-0149] Choudhry, R. M. , Fang, D. , & Mohamed, S. (2007). The nature of safety culture: A survey of the state‐of‐the‐art. Safety Science, 45(10), 993–1012.

[cl21234-bib-0150] Christian, M. S. , Bradley, J. C. , Wallace, J. C. , & Burke, M. J. (2009). Workplace safety: A meta‐analysis of the roles of person and situation factors. Journal of Applied Psychology, 94, 1103–1127. 19702360 10.1037/a0016172

[cl21234-bib-0151] Christopher, J. , & Conner, M. (2001). Efficacy of the Theory of Planned Behaviour: A meta‐analytic review. British Journal of Social Psychology, 40(4), 471–499. 11795063 10.1348/014466601164939

[cl21234-bib-0152] Concha‐Barrientos, M. , Nelson, D. I. , Fingerhut, M. , Driscoll, T. , & Leigh, J. (2005). The global burden due to occupational injury. American Journal of Industrial Medicine, 48, 470–481. 16299709 10.1002/ajim.20226

[cl21234-bib-0153] Cooper, D. (2007). Behavioral safety approaches: Which are the most effective. White Paper—Behavioural Safety Approaches—August 2007, 1‐13. BSMS Inc.

[cl21234-bib-0154] Cooper, D. (2009). Behavioral Safety Interventions: A review of process design factors. Professional Safety, 54(2), 36–45.

[cl21234-bib-0155] Cox, S. J. , & Jones, B. M. (2006). Behavioural safety and accident prevention: Short‐term “fad” or sustainable “fix”? Trans IChemE, Part B. Process Safety and Environmental Protection, 84(B3), 164–170. 10.1205/psep.05186

[cl21234-bib-0156] da Costa, B. R. , & Vieira, E. R. (2008). Stretching to reduce work‐related musculoskeletal disorders: A systematic review. Journal of Rehabilitation Medicine, 40(5), 321–328. 18461255 10.2340/16501977-0204

[cl21234-bib-0157] Davis, R. M. , & Pless, B. (2001). BMJ bans "accidents". BMJ, 322(7298), 1320–1321. 11387166 10.1136/bmj.322.7298.1320 PMC1120417

[cl21234-bib-0158] Dedobbeleer, N. , & Béland, F. (1991). A safety climate measure for construction sites. Journal of Safety Research, 22, 97–103.

[cl21234-bib-0159] DeJoy, D. M. (2005). Behavior change versus culture change: Divergent approaches to managing workplace safety. Safety Science, 43, 105–129.

[cl21234-bib-0160] DeRoo, L. A. , & Rautiainen, R. H. (2000). A systematic review of farm safety interventions. American Journal of Preventive Medicine, 18(4), 51–62. 10793281 10.1016/s0749-3797(00)00141-0

[cl21234-bib-0161] DerSimonian, R. , & Laird, N. (1986). Meta‐analysis in clinical trials. Controlled Clinical Trials, 7, 177–188. 3802833 10.1016/0197-2456(86)90046-2

[cl21234-bib-0162] Dyreborg, J. (2011). ‘Safety matters have become too important for management to leave it up to the workers’—The Nordic OSH model between implicit and explicit frameworks. Nordic Journal of Working Life Studies, 1(1), 135–160.

[cl21234-bib-0163] Dyreborg, J. , Lipscomb, H. , Olsen, O. , Törner, M. , Nielsen, K. , Lund, J. , Kines, P. , Guldenmund, F. , Bengtsen, E. , & Gensby, U. (2015). Safety interventions for the prevention of accidents at work: A protocol. Campbell Systematic Reviews, 11, 1–70. 10.1002/cl2.1234 PMC9159701 36911341

[cl21234-bib-0164] Effective Practice and Organisation of Care (EPOC) . (2016). Suggested risk of bias criteria for EPOC reviews. EPOC Resources for review authors (2016). Norwegian Knowledge Centre for the Health Services. http://epoc.cochrane.org/epoc-specific-resources-review-authors

[cl21234-bib-0165] EU‐OSHA . (2017). *Estimating the costs of work‐related accidents and ill‐health: An analysis of European data sources*. Publications Office of the European Union. https://osha.europa.eu/en/tools-and-publications/publications/estimating-cost-work-relatedaccidents-and-ill-health-analysis/view

[cl21234-bib-0166] European Commission . (1999). *European statistics on accidents at work: Methodology* (1998 ed.). Eurostat.Office for Official Publications of the European Communities. Theme 3: Population and Social Conditions. Methods and Nomenclature.

[cl21234-bib-0167] Eurostat . (2004a). *Statistical analysis of Socio‐economic costs of accidents at work in the European Union*. Working papers and studies. Luxembourg, Office for Official Publications of the European Communities. Theme 3: Population and social conditions.

[cl21234-bib-0168] Eurostat . (2004b). *Work and health in the EU. A statistical portrait. Panaroma of the European Union*. Office for Official Publications of the European Communities. Theme 3: Population and social conditions.

[cl21234-bib-0169] Eurostat . (2009). *8.6% of workers in the EU experienced work‐related health problems Results from the Labour Force Survey 2007 ad hoc module on accidents at work and work‐related health problems*. Eurostat, Statistics in focus, 63/2009.

[cl21234-bib-0170] Eurostat . (2012). *Number of non‐fatal and fatal accidents at work, 2012 in the EU‐28*. European Union. Office for Official Publications of the European Communities.

[cl21234-bib-0171] Eurostat . (2015). *Labour force survey: Accidents at work*. http://ec.europa.eu/eurostat/web/lfs/data/database

[cl21234-bib-0172] Eurostat . (2017). *Accidents at work statistics*. http://ec.europa.eu/eurostat/statistics-explained/index.php/Accidents_at_work_statistics

[cl21234-bib-0173] Farrington‐Darby, T. , Pickup, L. , & Wilson, J. R. (2005). Safety culture in railway maintenance. Safety Science, 43, 39–60.

[cl21234-bib-0174] Fishbein, M. , & Ajzen, I. (1975). Belief, attitude, intention, and behavior: An introduction to theory and research. Addison‐Wesley.

[cl21234-bib-0175] Flin, R. , Mearns, K. , O'Connor, P. , & Bryden, R. (2000). Measuring safety climate: Identifying the common features. Safety Science, 34, 177–192.

[cl21234-bib-0176] Fraser, C. , & Thomson‐O'Brien, M. A. (2000). Identifying non‐randomised studies in Medline. 8th International Congress on Medical Librarianship, July 2–5.

[cl21234-bib-0177] Gadd, S. , & Collins, A. M. (2002). Safety culture: A review of the literature. Sheffield, Health & Safety Laboratory.

[cl21234-bib-0178] Glasziou, P. P. , Irwig, L. , Bain, C. , & Colditz, G. (2001). Systematic reviews in health care. Cambridge University Press.

[cl21234-bib-0179] Glendon, A. I. , & Litherland, D. K. (2001). Safety climate factors, group differences and safety behaviour in road construction. Safety Science, 39, 157–188.

[cl21234-bib-0295] Gray, W. B. , & Mendeloff, J. (2002). The declining effects of OSHA inspections on manufacturing injuries: 1979 to 1998 . NBER Working paper No, 9119 JEL No. J28.

[cl21234-bib-0296] Gray, W. B. , & Scholz, J. T. (1991). *Do OSHA inspections reduce injuries? A panel analysis*. National Bureau of Economic Research, 1991. Working paper 3774, p. 38.

[cl21234-bib-0180] Grote, G. (2007). Understanding and assessing safety culture through the lens of organizational management of uncertainty. Safety Science, 45, 637–652.

[cl21234-bib-0181] Guastello, S. J. (1993). Do we really know how well our occupational accident prevention programs work? Safety Science, 16, 445–463.

[cl21234-bib-0182] Guldenmund, F. W. (2000). The nature of safety culture: A review of theory and research. Safety Science, 34, 215–257.

[cl21234-bib-0183] Guldenmund, F. W. (2010a). (Mis)understanding safety culture and its relationship to safety management. *Risk analysis*, Early view (published online in advance of print). 10.1111/j.1539-6924.2010.01452.x 20626685

[cl21234-bib-0184] Guldenmund, F. W. (2010b). Understanding and exploring safety culture. Uitgeverij Boxpress.

[cl21234-bib-0185] Haddon, W. (1968). The changing approach to the epidemiology, prevention, and amelioration of trauma: The transition to approaches etiologically rather than descriptively based. Phenomena of Trauma, 1968, 1431–1438. 10.2105/ajph.58.8.1431 PMC1228774 5691377

[cl21234-bib-0186] Hale, A. R. (2006). Method in your madness: System in your safety. Teknische Universiteit Delft.

[cl21234-bib-0187] Hale, A. R. , Guldenmund, F. W. , van Loenhout, P. L. C. H. , & Oh, J. I. H. (2010). Evaluating safety management and culture interventions to improve safety: Effective intervention strategies. Safety Science, 48, 1026–1035.

[cl21234-bib-0188] Hale, A. R. , & Hovden, J. (1998). Management and culture: The third age of safety. A review of approaches to organizational aspects of safety, health and environment. In A.‐M. Feyer & A. Williamson (Eds.), Occupational injury: Risk, prevention and intervention (pp. 129–165). Taylor & Francis Ltd.

[cl21234-bib-0189] Haslam, R. A. , Hide, S. A. , Gibb, A. G. , Gyi, D. E. , Pavitt, T. , Atkinson, S. , & Duff, A. R. (2005). Contributing factors in construction accidents. Applied Ergonomics, 36, 401–415. 15892935 10.1016/j.apergo.2004.12.002

[cl21234-bib-0190] Hayashino, Y. , Noguchi, Y. , & Fukui, T. (2005). Systematic evaluation and comparison of statistical tests for publication bias. Journal of Epidemiology, 15(6), 235–243. 16276033 10.2188/jea.15.235 PMC7904376

[cl21234-bib-0191] Heinrich, H. W. (1931). Industrial accident prevention. McGraw‐Hill.

[cl21234-bib-0192] Herrick, R. , & Dement, J. (1994). Chapter 10. Industrial hygiene. In L. Rosenstock & M. Cullen , Textbook of clinical occupational and environmental medicine (pp. 169–193). WB Saunders Co.

[cl21234-bib-0193] Higgins, J. P. T. , & Green, S. (2008). Cochrane handbook for systematic reviews of interventions. Wiley‐Blackwell.

[cl21234-bib-0194] Hofmann, D. A. , Jacobs, R. , & Landy, F. (1995). High reliability process industries: Individual, micro, and macro organizational influences on safety performance. Journal of Safety Research, 26, 131–149.

[cl21234-bib-0195] Hofmann, D. A. , & Tetrick, L. E. (2003). Health and safety in organizations—A multilevel perspective. Jossey‐Bass.

[cl21234-bib-0196] Hogan, D. A. , Greiner, B. A. , & O'Sullivan, L. (2014). The effect of manual handling training on achieving training transfer, employee's behaviour change and subsequent reduction of work‐related musculoskeletal disorders: A systematic review. Ergonomics, 57(1), 93–107. 24387742 10.1080/00140139.2013.862307

[cl21234-bib-0197] Hsiao, H. , & Simeonov, P. (2001). Preventing falls from roofs: A critical review. Ergonomics, 44, 537–561. 11345496 10.1080/00140130110034480

[cl21234-bib-0198] Kines, P. , Andersen, L. P. , Mikkelsen, K. L. , Dyreborg, J. , & Zohar, D. (2010). Improving construction site safety through leader‐based safety communication. Journal of Safety Research, 41, 399–406. 21059457 10.1016/j.jsr.2010.06.005

[cl21234-bib-0199] Kines, P. , Lappalainen, J. , Mikkelsen, K. L. , Pousette, A. , Tharaldsen, J. , Tómasson, K. , & Törner, M. (2011). Nordic Safety Climate Questionnaire (NOSACQ‐50): A new tool for measuring occupational safety climate. International Journal of Industrial Ergonomics, 41, 634–646.

[cl21234-bib-0200] Kjellén, U. (2000). Prevention of accidents through experience feedback. Taylor & Francis.

[cl21234-bib-0201] Krause, T. R. , Seymour, K. J. , & Sloat, K. C. M. (1999). Long‐term evaluation of a behavior‐based method for improving safety performance: A meta‐analysis of 73 interrupted time‐series replications. Safety Science, 32, 1–18.

[cl21234-bib-0202] Krause, T. R. , & Russel, L. R. (1994). The behavior‐based approach to proactive accident investigation. *Professional Safety*, 22–26.

[cl21234-bib-0203] Kristensen, T. S. (2005). Intervention studies in occupational epidemiology. Occupational and Environmental Medicine, 62, 205–210. 15723887 10.1136/oem.2004.016097 PMC1740975

[cl21234-bib-0204] Laitinen, H. , & Ruohomäki, I. (1996). The effects of feedback and goal setting on safety performance at two construction sites. Safety Science, 24(1), 61–73.

[cl21234-bib-0205] Laitinen, H. , Marjamäki, M. , & Päivärinta, K. (2003). The validity of the TR safety observation method on building construction. Accident Analysis & Prevention, 31, 463–472. 10.1016/s0001-4575(98)00084-0 10440543

[cl21234-bib-0206] Laitinen, H. , & Päivärinta, K. (2010). A new‐generation safety contest in the construction industry–A long‐term evaluation of a real‐life intervention. Safety Science, 48, 680–686.

[cl21234-bib-0207] Landeweerd, J. A. , Urlings, I. J. M. , De Jong, A. H. J. , Nijhuis, F. J. N. , & Bouter, L. M. (1990). Risk taking tendency among construction workers. Journal of occupational accidents, 11, 183–196.

[cl21234-bib-0301] Leffer, M. , & Grizzell, T. (2010). Implementation of a physician‐organized wellness regime (POWR) enforcing the 2007 NFPA standard 1582: Injury rate reduction and associated cost savings. Journal of Occupational and Environmental Medicine, 52(3), 36–339. 10.1097/JOM.0b013e3181d44d8d 20190648

[cl21234-bib-0208] Lingard, H. , & Holmes, N. (2001). Understandings of occupational health and safety risk control in small business construction firms: Barriers to implementing technological controls. Construction Management and Economics, 19(2), 217–226.

[cl21234-bib-0209] Lipscomb, H. J. (2000). Effectiveness of interventions to prevent work‐related eye injuries. American Journal of Preventive Medicine, 18(4), 27–32. 10.1016/s0749-3797(00)00138-0 10793278

[cl21234-bib-0210] Lipscomb, H. J. , Li, L. , & Dement, J. (2003). Work‐related falls among union carpenters in Washington State before and after the Vertical Fall Arrest Standard. American Journal of Industrial Medicine, 44, 157–165. 12874848 10.1002/ajim.10254

[cl21234-bib-0211] Lipscomb, H. J. , Pompeii, L. A. , Myers, D. I. , Schoenfisch, A. L. , & Dement, J. M. (2009). Systematic reviews of workplace injury interventions: What are we missing? La Medicina del Lavoro, 100, 247–257. 19764180

[cl21234-bib-0212] Lipscomb, H. J. , Nolan, J. , Patterson, D. , & Dement, J. M. (2008). Prevention of traumatic nail gun injuries in apprentice carpenters: Use of population‐based measures to monitor intervention effectiveness. American Journal of Industrial Medicine, 51(10), 719–727. 18704898 10.1002/ajim.20628 PMC2574677

[cl21234-bib-0213] Lipsey, M. W. , & Wilson, D. B. (2001). Practical meta‐analysis. Sage Publications.

[cl21234-bib-0214] Lisa, A. D. , & Risto, H. R. (2000). A systematic review of farm safety interventions. American Journal of Preventive Medicine, 18(4), 51–62. 10793281 10.1016/s0749-3797(00)00141-0

[cl21234-bib-0215] Lehtola, M. M. , van der Molen, H. F. , Lappalainen, J. , Hoonakker, P. L. T. , Hongwei, H. , Haslam, R. A. , & Hale, A. R. (2008). The effectiveness of interventions for preventing injuries in the construction industry: A systematic review. American Journal of Preventive Medicine, 35(1), 77–85. 18482821 10.1016/j.amepre.2008.03.030

[cl21234-bib-0216] Lewin, K. (1947). Frontiers in group dynamics. Concepts, method and reality in social science; social equilibria and social change. Human Relations, 1, 5–38.

[cl21234-bib-0217] Lund, J. , & Aarø, L. E. (2004). Accident prevention. Presentation of a model placing emphasis on human, structural and cultural factors. Safety Science, 42, 271–324.

[cl21234-bib-0218] Luthans, F. , & Kreitner, R. (1985). Organizational behavior modification and beyond. An operant and social learning approach. Scott, Foresman and Company.

[cl21234-bib-0302] Manuele, F. A. (2014). Incident investigation: Our methods are flawed. Professional Safety, 59(10), 34–43.

[cl21234-bib-0219] McAfee, R. B. , & Winn, A. R. (1989). The use of incentives/feedback to enhance workplace safety: A critique of the literature. Journal of Safety Research, 20(1), 7–19.

[cl21234-bib-0220] McDonald, N. , Corrigan, S. , Daly, S. , & Cromie, S. (2000). Safety management systems and safety culture in aircraft maintenance organisations. Safety Science, 34, 151–176.

[cl21234-bib-0221] Mearns, K. , Whitaker, S. M. , & Flin, R. (2003). Safety climate, safety management practice and safety performance in offshore environments. Safety Science, 41, 641–680.

[cl21234-bib-0222] Mearns, K. , Hope, L. , Ford, M. T. , & Tetrick, L. E. (2010). Investment in workforce health: Exploring the implications for workforce safety climate and commitment. Accident Analysis & Prevention, 42, 1445–1454. 20538100 10.1016/j.aap.2009.08.009

[cl21234-bib-0223] Mischke, C. , Verbeek, J. H. , Morata, T. , Alvesalo‐Kuusi, A. , Neuvonen, K. , Clarke, S. , & Pedlow, R. (2013). Occupational safety and health enforcement tools for preventing occupational diseases and injuries. *Cochrane Database of Systematic Reviews*. (8, Art. No.: CD010183). 10.1002/14651858.CD010183.pub2 PMC11707410 23996220

[cl21234-bib-0297] Misumi, J. (1978). The effects of organizational climate variables, particularly leadership variables and group decisions on accident prevention. In the 19th International Congress of Applied Psychology.

[cl21234-bib-0298] Misumi, J. (1982). Action research on group decision making and organisation development. In H. Hiebsch , H. Brandstatter , H. H. Kelley (Eds.), Berlin: VEB Deutscher Verlag der Wissenschaften. 1982.

[cl21234-bib-0299] Mobasherizadeh, S. , Ebneshahidi, A. , Rahimi, M. , Ostadrahimi, M. , & Masoumi, G. R. (2011). Needle‐stick injuries in Isfahan, Iran: Quality improvement. *BMC Proceedings*.

[cl21234-bib-0300] Moore‐Ede, M. , Heitmann, A. , Guttkuhn, R. , Trutschei, U. , Aguirre, A. , & Croke, D. (2004). Circadian alertness simulator for fatigue risk assessment in transportation: Application to reduce frequency and severity of truck accidents. Aviation, Space, and Environmental Medicine, 75(3), A107–A118. 15018271

[cl21234-bib-0224] Morgeson, F. P. , & Hofmann, D. A. (1999). The structure and function of collective constructs: Implications for multilevel research and theory development. Academy of Management Review, 24, 249–265.

[cl21234-bib-0225] Nahrgang, J. D. , Morgeson, F. P. , & Hofmann, D. A. (2007). *Predicting safety performance: A meta‐analysis of safety and organizational constructs*. Poster session presented at the 22nd Annual Conference of the Society for Industrial and Organizational Psychology. New York, NY.

[cl21234-bib-0226] Nahrgang, J. D. , Morgeson, F. P. , & Hofmann, D. A. (2011). Safety at work: A meta‐analytic investigation of the link between job demands, job resources, burnout, engagement, and safety outcomes. Journal of Applied Psychology, 96, 71–94. 21171732 10.1037/a0021484

[cl21234-bib-0227] Nielsen, K. J. (2014). Improving safety culture through the health and safety organization: A case study. Journal of Safety Research, 48(0), 7–17. 24529086 10.1016/j.jsr.2013.10.003

[cl21234-bib-0228] Parker, D. , Lawrie, M. , & Hudson, P. (2006). A framework for understanding the development of organizational safety culture. Safety Science, 44, 551–562.

[cl21234-bib-0229] Petticrew, M. , & Roberts, H. (2006). Systematic reviews in the social science: A practical guide. Padstow, Cornwall, United Kingdom: Blackwell Publishing.

[cl21234-bib-0230] Pidd, K. , & Roche, A. M. (2014). How effective is drug testing as a workplace safety strategy? A systematic review of the evidence. Accident Analysis & Prevention, 71, 154–165. 24922614 10.1016/j.aap.2014.05.012

[cl21234-bib-0231] Rasmussen, J. (1997). Risk management in a dynamic society: A modelling problem. Safety Science, 27, 183–213.

[cl21234-bib-0232] Rautiainen, R. , Lehtola, M. M. , Day, L. M. , Schonstein, E. , Suutarinen, J. , Salminen, S. , & Verbeek, J. H. (2008). Interventions for preventing injuries in the agricultural industry. *Cochrane Database of Systematic Reviews: Reviews*, 2008. 10.1002/14651858.CD006398.pub2 PMC12433932 18254102

[cl21234-bib-0233] Reason, J. (1997). Managing the risks of organizational accidents. Ashgate Publishing Limited.

[cl21234-bib-0234] Reichers, A. E. , & Schneider, B. (1990). Climate and culture: An evolution of constructs. In B. Schneider (Ed.), Organizational climate and culture (pp. 5–39). Jossey‐Bass.

[cl21234-bib-0235] RevMan . (2014). Review Manager (RevMan) [Computer program]. Version 5.3. The Nordic Cochrane Centre, The Cochrane Collaboration.

[cl21234-bib-0236] Richter, A. , & Koch, C. (2004). Integration, differentiation and ambiguity in safety cultures. Safety Science, 42, 703–722.

[cl21234-bib-0237] Rivara, F. P. , & Thompson, D. C. (2000a). Prevention of falls in the construction industry—Evidence for program effectiveness. American Journal of Preventive Medicine, 18, 23–26. 10793277 10.1016/s0749-3797(00)00137-9

[cl21234-bib-0238] Rivara, F. P. , & Thompson, D. C. (2000b). Systematic reviews of injury‐prevention strategies for occupational injuries—An overview. American Journal of Preventive Medicine, 18, 1–3. 10.1016/s0749-3797(00)00134-3 10793273

[cl21234-bib-0239] Robson, L. S. , Clarke, J. , Cullen, K. , Bielecky, A. , Severin, C. , Bigelow, P. , Irvin, E. , Culyer, A. J. , Mahood, Q. (2005). The effectiveness of occupational health and safety management systems: A systematic review. Full report. Institute for Work & Health.

[cl21234-bib-0240] Robson, L. S. , Shannon, H. S. , Goldenhar, L. M. , & Hale, A. R. (2001). *Guide to evaluating the effectiveness of strategies for preventing work injuries—How to show whether a safety intervention really works* (Publication No. 2001‐119). NIOSH.

[cl21234-bib-0241] Robson, L. S. , Clarke, J. A. , Cullen, K. , Bielecky, A. , Severin, C. , Bigelow, P. L. , Irvin, E. , Culyer, A. , & Mahood, Q. (2007). The effectiveness of occupational health and safety management system interventions: A systematic review. Safety Science, 45, 329–353.

[cl21234-bib-0242] Robson, L. S. , Stephenson, C. M. , Schulte, P. A. , Amick, B. C., III , Irvin, E. L. , Eggerth, D. E. , & Peters, R. H. (2012). A systematic review of the effectiveness of occupational health and safety training. *Scandinavian Journal of Work, Environment & Health*, 193–208. 10.5271/sjweh.3259 22045515

[cl21234-bib-0243] Rogers, B. , & Goodno, L. (2000). Evaluation of interventions to prevent needlestick injuries in health care occupations. American Journal of Preventive Medicine, 18(4), 90–98. 10793285 10.1016/s0749-3797(00)00145-8

[cl21234-bib-0244] Rogers, E. M. (1995). Diffusion of innovations 4th ed. The Free Press.

[cl21234-bib-0245] Rothschild, M. L. (2000). Carrots, sticks, and promises: A conceptual framework for the management of public health and social issue behaviors. Social Marketing Quarterly, 6(4), 86–114.

[cl21234-bib-0246] Rundmo, T. (2000). Safety climate, attitudes and risk perception in Norsk Hydro. Safety Science, 34, 47–59.

[cl21234-bib-0247] Runyan, C. W. , Zakocs, R. C. , & Zwerling, C. (2000). Administrative and behavioral interventions for workplace violence prevention. American Journal of Preventive Medicine, 18(4), 116–127. 10793287 10.1016/s0749-3797(00)00147-1

[cl21234-bib-0248] Saari, J. (1998). Safety interventions: International perspectives. In F. Anne‐Marie & A. Williamson (Eds.), Occupational injury: Risk, prevention and intervention (pp. 179–194). Taylor & Francis.

[cl21234-bib-0249] Sáchez‐Meca, J. , Márin‐Martínes, F. , & Chacón‐Moscoso, S. (2003). Effect‐size indices for dichotomized outcomes in meta‐analysis. Psychological Methods, 8(4), 448–467. 14664682 10.1037/1082-989X.8.4.448

[cl21234-bib-0250] Sadayappan, M. , & Moaued, F. A. (2011). Systematic review of feedback mechanism improving safety in dynamic and static industry. Journal of SH&E Research, 6, 1–25.

[cl21234-bib-0251] Schein, E. H. (2004). Organizational culture and leadership. Jossey‐Bass.

[cl21234-bib-0252] Schneider, B. , Ehrhart, M. G. , & Macey, W. H. (2013). Organizational climate and culture. Annual Review of Psychology, 64, 361–388. 10.1146/annurev-psych-113011-143809 22856467

[cl21234-bib-0253] Schneider, B. , & Reichers, A. E. (1983). On the etiology of climates. Personnel Psychology, 36, 19–39.

[cl21234-bib-0254] Scott Geller, E. (2011). Psychological science and safety. Current Directions in Psychological Science, 20, 109–114.

[cl21234-bib-0255] Seo, D. C. , Torabi, M. R. , Blair, E. H. , & Ellis, N. T. (2004). A cross‐validation of safety climate scale using confirmatory factor analytic approach. Journal of Safety Research, 35(4), 427–445. 15474546 10.1016/j.jsr.2004.04.006

[cl21234-bib-0256] Shah, A. , Blackhall, K. , Ker, K. , & Patel, D. (2009). Educational interventions for the prevention of eye injuries. Cochrane Database of Systematic Reviews, 2009(4), 006527. 10.1002/14651858.CD006527.pub3 PMC7388744 19821372

[cl21234-bib-0257] Shannon, H. S. , Robson, L. S. , & Sale, J. E. M. (2001). Creating safer and healthier workplaces: Role of organizational factors and job characteristics. American Journal of Industrial Medicine, 40, 319–334. 11598981 10.1002/ajim.1106

[cl21234-bib-0258] Shea, B. J. , Grimshaw, J. M. , Wells, G. A. , Boers, M. , Andersson, N. , Hamel, C. , Porter, A. C. , Tugwell, P. , Moher, D. , & Bouter, L. M. (2007). Development of AMSTAR: A measurement tool to assess systematic reviews. BMC Medical Research Methodology, 7, 10. 17302989 10.1186/1471-2288-7-10 PMC1810543

[cl21234-bib-0259] Skinner, B. F. (1969). Contingencies of reinforcement. Appleton‐Century‐Crofts.

[cl21234-bib-0260] Slavin, R. E. (1995). Best evidence synthesis: An intelligent alternative to meta‐analysis. Journal of Clinical Epidemiology, 48(1), 9–18. 7853053 10.1016/0895-4356(94)00097-a

[cl21234-bib-0261] Spangenberg, S. (2010). *Large construction projects and injury prevention* [Doctoral dissertation]. National Research Centre for the Working Environment and Aalborg University.

[cl21234-bib-0262] Spangenberg, S. , Mikkelsen, K. L. , Kines, P. , Dyreborg, J. , & Baarts, C. (2002). The construction of the Øresund link between Denmark and Sweden: The effect of a multifaceted safety campaign. Safety Science, 40, 457–465.

[cl21234-bib-0263] Stajkovic, A. D. , & Luthans, F. (2001). Differential effects of incentive motivators on work performance. Academy of Management Journal, 4, 580–590.

[cl21234-bib-0264] Stojanovic, M. D. , & Ostojic, S. M. (2012). Preventing ACL injuries in team‐sport athletes: A systematic review of training interventions. Research in Sports Medicine, 20(3‐4), 223–238. 22742077 10.1080/15438627.2012.680988

[cl21234-bib-0265] Tompa, E. , Dolinschi, R. , Oliveira, C. , & Irvin, E. (2007). A. systematic review of OHS interventions with economic evaluations—Volume 2—Appendices. Institute for Work & Health.

[cl21234-bib-0266] Tompa, E. , Trevithick, S. , & McLeod, C. (2007). Systematic review of the prevention incentives of insurance and regulatory mechanisms for occupational health and safety. Scandinavian Journal of Work, Environment and Health, 33(2), 85–95. 10.5271/sjweh.1111 17460796

[cl21234-bib-0267] Tuncel, S. , Lotlikar, H. , Salem, S. , & Daraiseh, N. (2006). Effectiveness of behaviour based safety interventions to reduce accidents and injuries in workplaces: Critical appraisal and meta‐analysis. Theoretical Issues in Ergonomics Science, 7(3), 191–209.

[cl21234-bib-0268] van der Molen, H. F. , Lehtola, M. M. , Lappalainen, J. , Hoonakker, P. L. T. , Hsiao, H. , Haslam, R. , Hale, A. R. , Verbeek, J. H. (2007). Interventions for preventing injuries in the construction industry (Review). The Cochrane Library, 2008, 1–25. 10.1002/14651858.CD006251.pub2 17943901

[cl21234-bib-0269] van der Molen, H. , Lehtola, M. M. , Lappalainen, J. , Hoonakker, P. L. T. , Hsiao, H. , Haslam, R. , Hale, A. R. , & Verbeek, J. H. (2013). Interventions for preventing injuries in the construction industry (Review). The Cochrane Library, 2013, 1–44. 10.1002/14651858.CD006251.pub2 17943901

[cl21234-bib-0270] Van Poppel, M. N. , Koes, B. W. , Smid, T. , & Bouter, L. M. (1997). A systematic review of controlled clinical trials on the prevention of back pain in industry. Occupational and Environmental Medicine, 54(12), 841–847. 9470890 10.1136/oem.54.12.841 PMC1128963

[cl21234-bib-0271] Watt, A. M. , Patkin, M. , Sinnott, M. J. , Black, R. J. , & Maddern, G. J. (2010). Scalpel safety in the operative setting: A systematic review. Surgery, 147(1), 98–106. 19828169 10.1016/j.surg.2009.08.001

[cl21234-bib-0272] Vaughan, D. (1996). The challenger launch decision: Risky technology, culture, and deviance at NASA. The University of Chicago Press.

[cl21234-bib-0273] Verbeek, J. , Salmi, J. , Pasternack, I. , Jauhiainen, M. , Laamanen, I. , Schaafsma, F. , Hulshof, C. , & van Dijk, F. (2005). A search strategy for occupational health intervention studies. Occupational and Environmental Medicine, 62, 682–687. 16169913 10.1136/oem.2004.019117 PMC1740874

[cl21234-bib-0274] Weick, K. E. (1987). Organizational culture as a source of high reliability. California Management Review, 29, 112–127.

[cl21234-bib-0275] Weick, K. E. (1993). *Sensemaking in organizations: Small structures with large consequences* (Vols. 2).

[cl21234-bib-0276] Weick, K. E. (1995). Sensemaking in organizations. Sage.

[cl21234-bib-0303] Wicker, A. W. (1969). Attitudes versus actions: The relationship of verbal and overt behavioral responses to attitude objects. Journal of Social issues, 25(4), 41–78.

[cl21234-bib-0277] Wickizer, T. M. , Kopjar, B. , Franklin, G. , & Joesch, J. (2004). Do drug‐free workplace programs prevent occupational injuries? Evidence from Washington State. Health Services Research, 39, 91–110. 14965079 10.1111/j.1475-6773.2004.00217.x PMC1360996

[cl21234-bib-0278] Williamson, A. M. , Feyer, A.‐M. , Cairns, D. , & Biancotti, D. (1997). The development of a measure of safety climate: The role of safety perceptions and attitudes. Safety Sciences, 25, 15–27.

[cl21234-bib-0279] Zohar, D. (1980). Safety climate in industrial organizations: Theoretical and applied implications. Journal of Applied Psychology, 65, 96–102. 7364709

[cl21234-bib-0280] Zohar, D. (2002). The effects of leadership dimensions, safety climate, and assigned priorities on minor injuries in work groups. Journal of Organizational Behavior, 23, 75–92.

[cl21234-bib-0281] Zohar, D. (2003). Safety climate: Conceptual and measurement issues. In Quick, J. C. & Tetrick, L. E. (Eds.), Handbook of occupational health psychology (pp. 123–142). American Psychological Association.

[cl21234-bib-0282] Zohar, D. (2010). Thirty years of safety climate research: Reflections and future directions. Accident Analysis & Prevention, 42, 1517–1522. 20538108 10.1016/j.aap.2009.12.019

[cl21234-bib-0283] Zohar, D. , & Luria, G. (2003). The use of supervisory practices as leverage to improve safety behavior: A cross‐level intervention model. Journal of Safety Research, 34, 567–577. 14733991 10.1016/j.jsr.2003.05.006

[cl21234-bib-0284] Zohar, D. , & Luria, G. (2004). Climate as a social‐cognitive construction of supervisory safety practices: Scripts as proxy of behavior patterns. Journal of Applied Psychology, 89, 322–333. 15065978 10.1037/0021-9010.89.2.322

[cl21234-bib-0285] Zohar, D. , & Hofmann, D. (2012). Organizational culture and climate. In S. Kozlowski (Ed.), Handbook of industrial and organizational psychology (pp. 643–666). Oxford University Press.

[cl21234-bib-0286] Zwetsloot, G. I. J. M. , Kines, P. , Ruotsala, R. , Drupsteen, L. , Merivirta, M. L. , & Bezemer, R. (2017). The importance of commitment, communication, culture and learning for the implementation of the zero accident vision in companies. Safety Science, 96, 22–32.

