# Supplementary material for: Safety interventions for the prevention of accidents at work: A systematic review
Source: Campbell Syst Rev. 2022 Jun 1;18(2):e1234. doi: 10.1002/cl2.1234 (PMC9159701; doi:10.1002/cl2.1234)
Supplement: Supplementary file 1 — Supporting information. [file CL2-18-e1234-s001.docx]

# Appendices

## Search strategy

**PubMed search strategy from 1966 to 4 March 2015 at U.S. National Library of Medicine**

| **Search number** | **Terms** | **Total** |
| --- | --- | --- |
| **#1** | injur*[Title/Abstract] OR accident*[Title/Abstract] OR harm[Title/Abstract] OR fall[Title/Abstract] OR falling[Title/Abstract] OR burn[Title/Abstract] OR slip[Title/Abstract] OR poison[Title/Abstract] OR fatal[Title/Abstract] |  |
| **#2** | occupational[Text Word] OR work[Text Word] |  |
| **#3** | accidental falls[MeSH Terms] |  |
| **#4** | injuries[MeSH Subheading] |  |
| **#5** | #1 AND #2 |  |
| **#6** | #3 AND #2 |  |
| **#7** | #4 AND #2 |  |
| **#8** | #5 OR #6 OR #7 |  |
| **#9** | accidents, occupational[MeSH Terms] |  |
| **#10** | #8 OR #9 |  |
| **#11** | Safety[MeSH] OR “Safety Management”[MeSH] OR “prevention and control”[MeSH Subheading] OR safet*[Text Word] OR prevent*[Text Word] OR control[Text Word] OR controll*[Title/Abstract] OR risk[Title/Abstract] OR “risk”[MeSH Term] OR “risk management”[MeSH Terms] OR “accident prevention”[MeSH Terms] |  |
| **#12** | #10 AND #11 |  |
| **#13** | (randomized controlled trial[pt] OR randomized controlled trials[MeSH Terms] OR random allocation[MeSH Terms] OR double-blind method[MeSH Terms] OR single-blind method[MeSH Terms] OR clinical trial[pt] OR clinical trials[MeSH Terms] OR “clinical trial”[Text Word] OR ((singl*[Text Word] OR doubl*[Text Word] OR trebl*[Text Word] OR tripl*[Text Word]) AND (mask*[Text Word] OR blind*[Text Word])) OR “latin square”[Text Word] OR placebos[MeSH Terms] OR placebo*[Text Word] OR random*[Text Word] OR research design[MeSH Terms:noexp] OR comparative study[MeSH Terms] OR evaluation studies[MeSH Terms] OR follow-up studies[MeSH Terms] OR prospective studies[MeSH Terms] OR cross-over studies[MeSH Terms] OR control[Text Word] OR controll*[Title/Abstract] OR prospectiv*[Text Word] OR volunteer*[Text Word]) NOT (animal[MeSH Terms] NOT human[MeSH Terms]) [noexp=not exploded] |  |
| **#14** | #12 AND #13 |  |

**EMBASE search strategy from 1980 to 30 April 2015 at OVID**

| **Search number** | **Terms** | **Total** |
| --- | --- | --- |
| **#1** | injur$.ti. OR injur$.ab. OR accident$.ti. OR accident$.ab. OR harm.ti. OR harm.ab. OR fall.ti. OR fall.ab. OR falling.ti. OR falling.ab. OR burn.ti. OR burn.ab. OR slip.ti. OR slip.ab. OR poison.ti. OR poison.ab. OR fatal.ti. OR fatal.ab. [ti=title] [ab=abstract] |  |
| **#2** | (occupational OR work).tw. [tw=textword] |  |
| **#3** | falling/ |  |
| **#4** | occupational accident/ |  |
| **#5** | #1 and #2 |  |
| **#6** | #2 and #3 |  |
| **#7** | #4 OR #5 OR #6 |  |
| **#8** | safety.sh. [subject heading] |  |
| **#9** | safety management.mp. [mp=title, original title, abstract, name of substance word, subject heading, unique identifier] |  |
| **#10** | occupational safety/ |  |
| **#11** | "prevention and control"/ |  |
| **#12** | safet$.tw. [tw=textword] |  |
| **#13** | prevent$.tw. [tw=textword] |  |
| **#14** | control.tw. OR controll$.ti. OR controll$.ab. [tw=textword] [ti=title] [ab=abstrac |  |
| **#15** | risk.sh. OR risk.ti. OR risk.ab. [subject heading] [ti=title] [ab=abstract] |  |
| **#16** | accident prevention/ |  |
| **#17** | #8 OR #9 OR #10 OR #11 OR #12 OR #13 OR #14 OR #15 OR #16 |  |
| **#18** | #7 and #17 |  |
| **#19** | randomized controlled trial/ |  |
| **#20** | randomization/ |  |
| **#21** | double blind procedure/ |  |
| **#22** | single blind procedure/ |  |
| **#23** | clinical trial/ OR "clinical trial (topic)"/ |  |
| **#24** | clinical trial.tw. [tw=textword] |  |
| **#25** | (singl$ OR doubl$ OR trebl$ OR tripl$).tw. [tw=textword] |  |
| **#26** | (mask$ OR blind$).tw. [tw=textword] |  |
| **#27** | #25 and #26 |  |
| **#28** | latin square design/ |  |
| **#29** | placebo/ |  |
| **#30** | (placebo$ or random$).tw. [tw=textword] |  |
| **#31** | methodology/ |  |
| **#32** | comparative study/ |  |
| **#33** | evaluation/ |  |
| **#34** | follow up/ |  |
| **#35** | prospective study/ |  |
| **#36** | crossover procedure/ |  |
| **#37** | control.tw. OR controll$.ti. OR controll$.ab. OR prospectiv$.tw. OR volunteer$.tw. [tw=textword] [ti=title] [ab=abstract] |  |
| **#38** | #19 OR #20 OR #21 OR #22 OR #23 OR #24 OR #27 OR #28 OR #29 OR #30 OR #31 OR #32 OR #33 OR #34 OR #35 OR #36 OR #37 |  |
| **#39** | #18 and #38 |  |
| **#40** | limit #39 to human |  |

**CINAHL search strategy from 1981 to July 2015 at EBSCO**

| **Search number** | **Terms** | **Total** |
| --- | --- | --- |
| **#1** | (TI injur* OR AB injur*) OR (TI accident* OR AB accident*) OR (TI harm OR AB harm) OR (TI fall OR AB fall) OR (TI falling OR AB falling) OR (TI burn OR AB burn) OR (TI slip OR AB slip) OR (TI poison OR AB poison) OR (TI fatal OR AB fatal) [TI=title, AB=abstract] |  |
| **#2** | (TX occupational OR TX work) [TX= All Text] |  |
| **#3** | (MM “Accidental Falls”) [MM=heading is major subject] |  |
| **#4** | (#2 AND #3) |  |
| **#5** | (#1 OR #4) |  |
| **#6** | (MM Accidents, Occupational) OR (MM “Occupational Safety”) OR (MM “Safety”) OR (safet* OR prevent* OR control*) OR (TI risk OR AB risk) OR (MM “Risk Assessment”) OR (MM “Risk Management”) [TI=title, AB=abstract] [MM=heading is major subject] |  |
| **#7** | (5 AND #6) |  |
| **#8** | (MM ”Randomized Controlled Trials”) OR (MM Random Assignment”) OR (MM “Double-Blind Studies” OR (MM ”Single-Blind Studies”) OR (MH “Clinical Trials+”) OR (“clinical trial”) OR ((singl* OR doubl* OR trebl* OR tripl*) AND (mask* OR blind*)) OR “latin square” OR (MM “Placebos”) OR (“placebo”) OR (“random”) OR (MH “Prospective Studies+”) OR (MM “Crossover Design”) OR (MM “Study Design) OR (MM “Comparative Studies”) OR (MM “Evaluation Research”) OR (MM “Prospective Studies”) OR (TX control) OR (TX controll*) OR (AB controll*) OR (TX perspective*) OR (TX volunteer*) [MH=exact subject heading, both major and minor] [MM=heading is major subject] [TX= All Text] |  |
| **#9** | (#7 AND #8) /limit human |  |

**OSH UPDATE search strategy from 1974/1977 to 24 April 2015**

| Search number | Terms | Total |
| --- | --- | --- |
| #1 | [TW{injur*} OR AB{injur*} OR TW{accident*} OR AB{accident*} OR TW{harm*} OR AB{harm*} OR TW{fall*} OR AB{fall*} OR TW{burn*} OR AB{burn*} OR TW{slip*} OR AB{slip*} OR TW{poison*} OR AB{poison*} OR TW{fatal*} OR AB{fatal*}](javascript:%20doSubmit('QD1')) |  |
| #2 | [GW{occupation*} OR GW{Work*}](javascript:%20doSubmit('QD2')) |  |
| #3 | [#1 AND #2](javascript:%20doSubmit('QD3')) |  |
| #4 | [GW{safet*} OR GW{prevent*} OR GW{control*} OR GW{risk*}](javascript:%20doSubmit('QD4')) |  |
| #5 | [#3 AND #4](javascript:%20doSubmit('QD5')) |  |
| #6 | [DC{OUHSEL} OR DC{OUCISD} OR DC{OUNIOC}](javascript:%20doSubmit('QD6')) |  |
| #7 | [#5 AND #6](javascript:%20doSubmit('QD7')) |  |
| #8 | [GW{clinical trial*} OR GW{latin square} OR GW{placebo*} OR GW{random*} OR GW{research design*} OR GW{comparative stud*} OR GW{evaluation stud*} OR GW{follow-up stud*} OR GW{cross-over stud*} OR GW{control*} OR GW{prospectiv*} OR GW{volunteer*}](javascript:%20doSubmit('QD8')) |  |
| #9 | [GW{doubl*} OR GW{singl*} OR GW{trebl*} OR GW{tripl*}](javascript:%20doSubmit('QD9')) |  |
| #10 | [GW{mask*} OR GW{blind*}](javascript:%20doSubmit('QD10')) |  |
| #11 | [#9 AND #10](javascript:%20doSubmit('QD11')) |  |
| #12 | [#8 OR #11](javascript:%20doSubmit('QD12')) |  |
| #13 | [#7 AND #12](javascript:%20doSubmit('QD13')) |  |
| #14 | [TW{REVIEW} OR DE{REVIEW}](javascript:%20doSubmit('QD14')) |  |
| #15 | [#13 NOT #14](javascript:%20doSubmit('QD15')) |  |
| #16 | [#13 NOT #15](javascript:%20doSubmit('QD16')) |  |
| #17 | [DO{journal*}](javascript:%20doSubmit('QD17')) |  |
| #18 | [#15 AND #17](javascript:%20doSubmit('QD18')) |  |

**PsycINFO search strategy from 1806 to 20 February 2015 at APA**

| **Search number** | **Terms** | **Total** |
| --- | --- | --- |
| **#1** | OUTCOME:  (Title:(injury OR accident OR harm OR fall OR falling OR burn OR slip OR poison OR fatal) OR Abstract:(injury OR accident OR harm OR fall OR falling OR burn OR slip OR poison OR fatal)) AND (AnyField:(occupational OR work)) OR (IndexTerms:(falls OR accidents OR injuries)) AND (AnyField:(occupational OR work)) OR (AnyField:(occupational OR work)) AND (AnyField:(safety behavior OR safety behaviour OR risk behavior OR risk behaviour)) |  |
| **#2** | INTERVENTION:  safety OR risk management OR accident prevention:Index Terms OR safet* OR prevent* OR control* OR controll* OR risk:Title OR safet* OR prevent* OR control* OR controll* OR risk:Abstract |  |
| **#3** | OUTCOME + INTERVENTION:  IndexTerms:(safety OR risk management OR accident prevention) OR Title:(safet* OR prevent* OR control* OR controll* OR risk) OR Abstract:(safet* OR prevent* OR control* OR controll* OR risk) AND ((Title:(injury OR accident OR harm OR fall OR falling OR burn OR slip OR poison OR fatal) OR Abstract:(injury OR accident OR harm OR fall OR falling OR burn OR slip OR poison OR fatal)) AND (AnyField:(occupational OR work))) OR ((IndexTerms:(falls OR accidents OR injuries)) AND (AnyField:(occupational OR work))) OR ((AnyField:(occupational OR work)) AND (AnyField:(safety behavior OR safety behaviour OR risk behavior OR risk behaviour))) |  |
| **#4** | STUDY DESIGN:  "clinical trials" OR placebo OR "experimental design":Index Terms OR "comparative stud*" OR "random allocation" OR "double blind" OR "single blind" OR "evaluation stud*" OR "crossover" OR control* OR prospectiv* OR volunteer*:Any Field (+ methodology limit function) |  |
| **#5** | OUTCOME + INTERVENTION + STUDY DESIGN:  (IndexTerms:("clinical trials" OR placebo OR "experimental design") OR AnyField:("comparative stud*" OR "random allocation" OR "double blind" OR "single blind" OR "evaluation stud*" OR "cross over" OR control* OR prospectiv* OR volunteer*)) AND ((IndexTerms:(safety OR risk management OR accident prevention) OR Title:(safet* OR prevent* OR control* OR controll* OR risk) OR Abstract:(safet* OR prevent* OR control* OR controll* OR risk)) AND (((Title:(injury OR accident OR harm OR fall OR falling OR burn OR slip OR poison OR fatal) OR Abstract:(injury OR accident OR harm OR fall OR falling OR burn OR slip OR poison OR fatal)) AND (AnyField:(occupational OR work))) OR ((IndexTerms:(falls OR accidents OR injuries)) AND (AnyField:(occupational OR work))) OR ((AnyField:(occupational OR work)) AND (AnyField:(safety behavior OR safety behaviour OR risk behavior OR risk behaviour))))) OR ((IndexTerms:(safety OR risk management OR accident prevention) OR Title:(safet* OR prevent* OR control* OR controll* OR risk) OR Abstract:(safet* OR prevent* OR control* OR controll* OR risk)) AND (((Title:(injury OR accident OR harm OR fall OR falling OR burn OR slip OR poison OR fatal) OR Abstract:(injury OR accident OR harm OR fall OR falling OR burn OR slip OR poison OR fatal)) AND (AnyField:(occupational OR work))) OR ((IndexTerms:(falls OR accidents OR injuries)) AND (AnyField:(occupational OR work))) OR ((AnyField:(occupational OR work)) AND (AnyField:(safety behavior OR safety behaviour OR risk behavior OR risk behaviour)))) AND MethodologyFilt:"Prospective Study" ) OR ((IndexTerms:(safety OR risk management OR accident prevention) OR Title:(safet* OR prevent* OR control* OR controll* OR risk) OR Abstract:(safet* OR prevent* OR control* OR controll* OR risk)) AND (((Title:(injury OR accident OR harm OR fall OR falling OR burn OR slip OR poison OR fatal) OR Abstract:(injury OR accident OR harm OR fall OR falling OR burn OR slip OR poison OR fatal)) AND (AnyField:(occupational OR work))) OR ((IndexTerms:(falls OR accidents OR injuries)) AND (AnyField:(occupational OR work))) OR ((AnyField:(occupational OR work)) AND (AnyField:(safety behavior OR safety behaviour OR risk behavior OR risk behaviour)))) AND MethodologyFilt:"Followup Study" ) OR ((IndexTerms:(safety OR risk management OR accident prevention) OR Title:(safet* OR prevent* OR control* OR controll* OR risk) OR Abstract:(safet* OR prevent* OR control* OR controll* OR risk)) AND (((Title:(injury OR accident OR harm OR fall OR falling OR burn OR slip OR poison OR fatal) OR Abstract:(injury OR accident OR harm OR fall OR falling OR burn OR slip OR poison OR fatal)) AND (AnyField:(occupational OR work))) OR ((IndexTerms:(falls OR accidents OR injuries)) AND (AnyField:(occupational OR work))) OR ((AnyField:(occupational OR work)) AND (AnyField:(safety behavior OR safety behaviour OR risk behavior OR risk behaviour)))) AND MethodologyFilt:"Treatment Outcome/Clinical Trial" ) |  |

**EconLit search strategy 1969 to 9 July 2015***

| **Search number** | **Terms** | **Total** |
| --- | --- | --- |
| **#1** | ti(injur* OR accident*) OR ab(injur* OR accident*) |  |
| **#2** | ti(occupational* OR work*) OR ab(occupational* OR work*) OR ti(workplace OR worksite) OR ab(workplace OR worksite) |  |
| **#3** | #1 AND #2 |  |
| **#4** | ti(safety) OR ab(safety) OR ti(prevent* OR risk*) OR ab(prevent* OR risk*) OR ti(control* OR hazard*) OR ab (control* OR hazard*) OR ti(accident prevent*) OR ab(accident prevent*) |  |
| **#5** | #3 AND #4 /limit *peer reviewed journal* |  |

*In this database it was not possible to include *“study design”* in the search strategy

**Web of Science search strategy 1969 to 18 March 2015**

| **Search number** | **Terms** | **Total** |
| --- | --- | --- |
| #1 | TS=(injury OR accident OR harm OR fall OR falling OR burn OR slip OR poison OR fatal)  *Indexes=SCI-EXPANDED, SSCI, A&HCI Timespan=2011-2015* |  |
| #2 | TS=(occupational OR work)  *Indexes=SCI-EXPANDED, SSCI, A&HCI Timespan=2011-2015* |  |
| #3 | TS=(accidental falls)  *Indexes=SCI-EXPANDED, SSCI, A&HCI Timespan=2011-2015* |  |
| #4 | TOPIC: (injuries)  *Indexes=SCI-EXPANDED, SSCI, A&HCI Timespan=2011-2015* |  |
| #5 | #2 AND #1  *Indexes=SCI-EXPANDED, SSCI, A&HCI Timespan=2011-2015* |  |
| #6 | #3 AND #2  *Indexes=SCI-EXPANDED, SSCI, A&HCI Timespan=2011-2015* |  |
| #7 | #4 AND #2  *Indexes=SCI-EXPANDED, SSCI, A&HCI Timespan=2011-2015* |  |
| #8 | #7 OR #6 OR #5  *Indexes=SCI-EXPANDED, SSCI, A&HCI Timespan=2011-2015* |  |
| #9 | TOPIC: ("occupational accidents")  *Indexes=SCI-EXPANDED, SSCI, A&HCI Timespan=2011-2015* |  |
| #10 | #9 OR #8  *Indexes=SCI-EXPANDED, SSCI, A&HCI Timespan=2011-2015* |  |
| #11 | TOPIC: (safety OR control OR risk) *OR* TOPIC: (safety management) *OR* TOPIC: (safet* OR prevent*) *OR* TOPIC: (controll*) *OR* TOPIC: ("risk management") *OR* TOPIC: ("accident prevention")  *Indexes=SCI-EXPANDED, SSCI, A&HCI Timespan=2011-2015* |  |
| #12 | #11 AND #10  *Indexes=SCI-EXPANDED, SSCI, A&HCI Timespan=2011-2015* |  |
| #13 | TOPIC: ("randomized controlled trial" OR "randomized controlled trials" OR "random allocation" OR "double-blind method" OR "single-blind method")  *Indexes=SCI-EXPANDED, SSCI, A&HCI Timespan=2011-2015* |  |
| #14 | TOPIC: ("clinical trial" OR "clinical trials")  *Indexes=SCI-EXPANDED, SSCI, A&HCI Timespan=2011-2015* |  |
| #15 | TOPIC: (singl* OR doubl* OR trebl* OR tripl*) *AND* TOPIC: (mask* OR blind*)  *Indexes=SCI-EXPANDED, SSCI, A&HCI Timespan=2011-2015* |  |
| #16 | TOPIC: ("latin square" OR placebos OR placebo* OR random* OR "research design" OR "comparative stud*" OR "evaluation stud*" OR "follow-up stud*" OR "prospective stud*" OR "cross-over stud*" OR control OR controll* OR prospectiv* OR volunteer*)  *Indexes=SCI-EXPANDED, SSCI, A&HCI Timespan=2011-2015* |  |
| #17 | #16 OR #15 OR #14 OR #13  *Indexes=SCI-EXPANDED, SSCI, A&HCI Timespan=2011-2015* |  |
| #18 | #17 AND #12  *Indexes=SCI-EXPANDED, SSCI, A&HCI Timespan=2011-2015* |  |
| #19 | TS=(retrospective stud*) OR TS="interrupted time series" OR TS=(group* OR before-after OR pre-post OR pre-test OR "post test")  *Indexes=SCI-EXPANDED, SSCI, A&HCI Timespan=2011-2015* |  |
| #20 | #19 OR #16  *Indexes=SCI-EXPANDED, SSCI, A&HCI Timespan=2011-2015* |  |
| #21 | #20 OR #15 OR #14 OR #13  *Indexes=SCI-EXPANDED, SSCI, A&HCI Timespan=2011-2015* |  |
| #22 | #21 AND #12  *Indexes=SCI-EXPANDED, SSCI, A&HCI Timespan=2011-2015* |  |

**ProQuest Dissertations and Theses Professional search terms accessed at 26 June 2015 at proquest.com/professional**

| **Search number** | **Terms** | **Total** |
| --- | --- | --- |
| **#1** | ti(injur* OR accident*) OR ab(injur* OR accident*) |  |
| **#2** | occupational* OR work* OR workplace OR worksite |  |
| **#3** | #1 AND #2 |  |
| **#4** | Diskw.exact(“Safety” OR “PREVENTION” OR “RISK” OR “Risk” OR “accident prevention” OR “accidents”) OR (safet* OR prevent* OR control* OR hazard*) OR ti(risk) OR ab(risk) |  |
| **#5** | #3 AND #4 |  |

**WHO search terms accessed 9 July 2015 at WHO.int**

| **Search number** | **Terms** | **Total** |
| --- | --- | --- |
| **#1** | (accident OR injury) |  |
| **#2** | (occupational OR work OR workplace) |  |
| **#3** | (intervention) |  |
| **#4** | (review OR meta-analysis) |  |
| **#5** | #1 AND #2 AND # 3 NOT # 4 |  |

**OSHA – Europe search terms accessed at 8 July 2015 at osha.europe.eu/da**

| **Search number** | **Terms** | **Total** |
| --- | --- | --- |
| **#1** | Tools & Publications [Heading] |  |
| **#2** | Publications [Heading] |  |
| **#3** | Topics [Heading] |  |
| **#4** | (accident prevention OR dangerous substances OR musculoskeletal disorders OR women and OSH OR emerging risks OR OSH and young people OR risk assessment OR construction OR mainstreaming OSH into education OR HORECA OR leadership OR maintenance OR statistics OR nanomaterials OR ageing and OSH OR noise OR workplace health promotion OR road transport OR agriculture OR cleaning workers OR worker participation OR migrant workers OR fisheries OR transport) |  |

**ILO – International Labour Organisation search terms accessed at 16 July 2015 at ilo.org**

| **Search number** | **Terms** | **Total** |
| --- | --- | --- |
| **#1** | Publications [Heading] |  |
| **#2** | Working Papers [Heading] |  |
| **#3** | (accident) |  |

| **Search number** | **Terms** | **Total** |
| --- | --- | --- |
| **#1** | Publications [Heading] |  |
| **#2** | Working Papers [Heading] |  |
| **#3** | (injury) |  |

| **Search number** | **Terms** | **Total** |
| --- | --- | --- |
| **#1** | Publications [Heading] |  |
| **#2** | Working Papers [Heading] |  |
| **#3** | (occupational) |  |

| **Search number** | **Terms** | **Total** |
| --- | --- | --- |
| **#1** | Publications [Heading] |  |
| **#2** | Books and Reports [Heading] |  |
| **#3** | (occupational safety and health) |  |

| **Search number** | **Terms** | **Total** |
| --- | --- | --- |
| **#1** | Publications [Heading] |  |
| **#2** | Magazines and Newsletters [Heading] |  |
| **#3** | (International Journal of Labour Research) |  |
| **#4** | (accident OR injury AND occupational OR work OR workplace AND intervention) |  |

**Safetylit search terms accessed at 13 July 2015 at safetylit.org**

| **Search number** | **Terms** | **Total** |
| --- | --- | --- |
| **#1** | Search [Heading] |  |
| **#2** | (accident) OR (injury) [Textword+Synonyms] |  |
| **#3** | (occupational) OR (work) OR (workplace) [Textword+Synonyms] |  |
| **#4** | (intervention) [Textword+Synonyms] |  |
| **#5** | (review) OR (meta analysis) OR (meta-analysis) [Textword+Synonyms] |  |
| **#6** | #2 AND #3 AND #4 NOT #5 |  |

**Eurofound search terms accessed at 15 July 2015 at eurofound.europe.eu**

| **Search number** | **Terms** | **Total** |
| --- | --- | --- |
| **#1** | Publication [Heading] |  |
| **#2** | Topic [Heading] |  |
| **#3** | (Health and Safety at Work) OR (Physical Work Hazards) |  |

**OECD search terms accessed at 9 July 2015 at OECD.org**

| **Search number** | **Terms** | **Total** |
| --- | --- | --- |
| **#1** | OECD-libarary [Heading] |  |
| **#2** | Browse by theme[Heading] |  |
| **#3** | Agriculture and Food [Heading] |  |
| **#4** | Papers [Heading] |  |
| **#5** | Working/Policy Papers [Heading] |  |
| **#6** | OECD Food, Agriculture and Fisheries Papers |  |

| **Search number** | **Terms** | **Total** |
| --- | --- | --- |
| **#1** | OECD-libarary [Heading] |  |
| **#2** | Browse by theme[Heading] |  |
| **#3** | Employment [Heading] |  |
| **#4** | Papers [Heading] |  |
| **#5** | Working/Policy Papers [Heading] |  |
| **#6** | OECD Social, Employment and Migration Working Papers |  |

**NIOSH search terms accessed at 15 July 2015 at cdc.gov/niosh/**

| **Search number** | **Terms** | **Total** |
| --- | --- | --- |
| **#1** | Publications and Products [Heading] |  |
| **#2** | Search NIOSHTIC-2 Research Database [Heading] |  |
| **#3** | (accident) OR (injury) [All Fields] |  |
| **#4** | (occupational) OR (work) OR (workplace) [All Fields] |  |
| **#5** | (intervention) [All Fields] |  |
| **#6** | (review) OR (meta analysis) OR (meta-analysis) [All Fields] |  |
| **#7** | #3 AND #4 AND #5 NOT #6 |  |

**Cochrane Central Register of Controlled Trials search terms accessed at 4 June 2015 at onlinelibrary.wiley.com/cochranelibrary/search/advanced**

| **Search number** | **Terms** | **Total** |
| --- | --- | --- |
| **#1** | injur* OR accident* OR harm OR harmful OR fall OR falls OR burn OR burns OR poison* OR slip OR slippery OR trip OR trips OR fatal*:ti,ab,kw (Word variations have been searched) |  |
| **#2** | occupation* OR work OR workplace OR worksite*:ti,ab,kw (Word variations have been searched) |  |
| **#3** | #1 AND #2 |  |
| **#4** | safet* OR prevent* OR risk OR accident* OR control* OR hazard*:ti,ab,kw (Word variations have been searched) |  |
| **#5** | #3 AND #4 |  |
| **#6** | “post test” OR “random allocation” OR placebo* OR quasi* OR “research design” OR control* OR retrospective* OR “randomized controlled trial” OR RCT OR “clinical trial” OR “comparative study” OR “evaluation study” OR “follow up” OR prospective or “interrupted time” OR “before-after” OR “pre-test” OR “post-test” OR “double-blind” OR “single-blind” |  |
| **#7** | #5 AND #6 |  |

**Perosh search terms accessed at 8 July 2015 at Persosh.eu**

| **Search number** | **Terms** | **Total** |
| --- | --- | --- |
| **#1** | Research projects [Heading] |  |
| **#2** | Safety Culture and Accidents [Heading] |  |

## Screening procedures

These are reported in the study protocol (Dyreborg J, et.al. 2015).

## Types of safety interventions

**Some coding instructions for included studies:** The following classification is used to classify types of safety intervention for included studies. The type of safety intervention that a study aims to evaluate should be coded. For example, in cases where the study evaluates the effect of the PPE as such, e.g., to what extend gloves technically can provide a barrier between the person and the harmful substances (code 2.2.4 Engineering controls), or to what extend a safety campaign increases the use of PPE and in turn reduces injuries (code 1.1.1.: Safety campaign). A safety intervention could include one or more of the following components (code e.g., 1.1.2 or 2.1.1 or other code, or in case of more components: e.g., 1.1.2 + 1.2.3, then code = 3.1; e.g., 2.1.2 + 2.2.3, then code 3.2; e.g., 1.1.1+1.2.2+2.2.4, then code=3.3):

1. **Main type of components directed at the individual level** (Not specified)

*1.1.0.: Attitude and belief modification*: attitudes or beliefs are modified by means of knowledge, information and persuasive messages in campaigns, leaflets, booklets, films, posters, direct mail, guidelines, or various counselling approaches etc. (Not specified)

1.1.1 Safety campaign, by use of various means (one way communication)

1.1.2 Counselling approaches (two way communication, including group discussions)

1.1.3 Teaching, education to increase knowledge and awareness (classroom, workplace etc. but not training (1.2.2) that is focusing on skills)

1.1.9 Other types of attitude modifications not listed above

Excludes, e.g., skill training focused on behaviour change, coded under 1.2.2.

*1.2.0.: Behavior modification*: behavior is modified through various approaches, such as training, incentives, goal setting, feedback, individual coaching etc. (Not specified)

1.2.1 Individual goal setting (implicit, assigned, participative or self-assigned)

1.2.2 Safety training, and other types of training in order to improve skills, proficiency, dexterity, and other types of accomplishments (e.g., skill training, not just attitudinal changes)

1.2.3 Incentives (economic, benchmarking, safety bonus and other individual level incentives)

1.2.4 Individual feedback or safety coaching (e.g., face-to-face, and self-feedback, such as completion of a checklist)

1.2.9 Other types of behavior modifications not listed above

**1.9 Other main types of components directed at the individual level,** not listed above

*1.3.0.: Physiological modification*: Human physiology is modified through various training methods, such as, endurance training; strength and resistance training; flexibility exercises; with the aim to reduce the risk of injury. Other methods could include weight loss. (Not further specified)

1.3.1 Mixed physical training methods (such as Endurance training and Strength and resistance training; Flexibility exercises and Endurance training; Strength and resistance training and Flexibility exercises, or combinations of all three main types of training methods)

1.3.2 Endurance training (running, cycling, swimming etc.)

1.3.3 Strength and resistance training (such as push-ups, pull-ups, weight training, interval training etc.)

1.3.4 Flexibility exercises

1.3.9 Other types of training methods, not listed above

**2. Main types of components at the group or organizational level** (Not further specified)

*2.1.0.: Culture and climate modifications*: climate, social norms and culture, may be changed through leadership-based interventions, introduction or modification of safety management approaches, goal-setting, safety-coaching or by the influence of sectorial- or societal-level changes and modification aimed at changes at the place of work. (Not specified).

2.1.1 Goal setting at group or organizational level (implicit, assigned, participative or self-assigned)

2.1.2 Safety-coaching – teams, departments etc. (mainly employee directed)

2.1.3 Safety-coaching, leaders (top-leaders, middle management and nearest leaders)

2.1.4 Competitions (benchmarking, safety bonus and other group or organizational level incentives)

2.1.5 Safety feedback to work groups, leaders and workplace in general (e.g., face-to-face, public feedback, briefing at toolbox meetings, self-feedback, such as completion of a checklist)

2.1.6 Safety feedback to stakeholders, or other persons or groups outside the workplace who can influence workplace safety, e.g., the owner or client of a construction project (Types include face-to-face, public feedback, briefing, meetings, or other types of safety performance measurements)

2.1.7 Leadership based safety interventions aiming at changing safety climate

2.1.8 Approaches aiming at changing the safety culture.

2.1.9 Other types of culture and climate modifications, not listed above

*2.2.0.: Structural modification*: contextual factors are changed through legislation, regulation, enforcement and economics. This also refers to changes in the organization of safety management systems, the physical environment, engineering, i.e., modification of equipment and products, and implementation of rules and regulation, workers’ rights and interests etc. (Not specified).

2.2.1 Legislative changes and introduction of specific parts of laws and regulation which makes firms comply (primary deterrence related with the institutional effects of legislation)

2.2.2 Economic incentives (incl. insurance premium cost reduction, and other types of insurance incentives)

2.2.3 Soft regulation (e.g., CSR, agreements between social partners or industry groups, benchmarking between companies, action plans, certification processes, and other approaches without legal binding)

2.2.4 Engineering controls, such as design, availability and maintenance of technology (e.g., modifications in physical environment, engineering, modification of equipment and products, such as safety guards on machines, availability of lifting devices and the like), includes also elimination and substitution of risk factors (such as asbestos or noise).

2.2.5 Administrative controls, such as introduction or modification of safety policies (e.g., new lifting policies), Safety Management Systems (e.g., ISO/OHSAS or other safety standards or guidelines), including modifications of monitoring, feedback & learning systems (e.g., procedures for incident, accident, dangerous situation reporting, audit systems, audit inspections etc.). See also soft law.

2.2.6 Employee participation or involvement is the introduction or modification of an environment in which people have an impact on decisions and actions that affect their jobs and their safety in these jobs. This include worker participation in the planning and selection of tools, procedures and goals, or by improving rights to participation in company decisions and to participation in e.g., Health and Safety boards, advisory committees etc.

2.2.7 Enforcement of laws and regulations, including inspection, issuing warnings, penalties, fines and ultimately close downs. Enforcement work by general primary deterrence (code 2.2.1), which makes firms comply after warnings, penalties, fines etc., have been issued (specific deterrence).

2.2.8 Social marketing and other approaches to diffuse knowledge through various channels (mass media).

2.2.9 Other types of structural modifications, not listed above

**2.9 Other main types of components directed at group or organizational level, not listed above**

**3. Multifaceted safety interventions** (combination of components not specified):

*3.1* Combination of components at the individual level.

3.2 Combination of components at the group or organizational level

3.3 Combination of components across levels (individual and group/organizational level)

**3.9** Combination of components, not listed above

**9. Main types of components not listed above,** *describe in free text:*

## Data extraction overview

**Table 12.1. Data extraction questions and research designs, overview.**

| Study design | RCT | CBA | B&A | ITS | Other |
| --- | --- | --- | --- | --- | --- |
| **Q1.** How was comparison/control group formed? | x | x |  |  |  |
| **Q2.** Random assignment, specify design: | x |  |  |  |  |
| **Q3.** How was random assignment performed? | x |  |  |  |  |
| **Q4:** How many different types of intervention groups are there? | x | x | x | x | x |
| **Q5:** How many relevant control groups are there? | x | x |  |  |  |
| **Q6**: Study sample size. Please complete form | x | x | x | x | x |
| Participants |  |  |  |  |  |
| **Q7:** Were participant inclusion and/or exclusion criteria mentioned? | x | x | x | x | x |
| **Q8:** Participant background characteristics. Please complete form | x | x | x | x | x |
| **Q9**: Were there any differences between intervention and comparison groups at baseline? | x | x |  |  |  |
| **Q10**: Were there any analyses of differences between completers and dropouts in the intervention group? | x | x | x | x | x |
| **Q11**: Was intention to treat analysis used? | x | x | x | x | x |
| Intervention |  |  |  |  |  |
| **Q12**: Describe the safety intervention | x | x | x | x | x |
| **Q13:** Record exact details of the duration of the safety intervention | x | x | x | x | x |
| **Q14:** Record exact details of the intensity of the safety intervention | x | x | x | x | x |
| **Q15**: If any, describe methods used to ensure the quality and implementation/fidelity of the safety intervention | x | x | x | x | x |
| **Q16**: Compliance with the safety intervention | x | x | x | x | x |
| **Q17**: Were there any co-interventions (contextual factors) not related to the safety intervention studied, which could influence outcome in the intervention and/or control group? | x | x | x | x | x |
| **Q18**: Type of control/comparison group: | x | x |  |  |  |
| Outcome measures |  |  |  |  |  |
| **Q19:** How many baseline measurements were carried out? | x | x | x | x | x |
| **Q20:** When were the first and last follow-up measurements done? | x | x | x | x | x |
| **Q21:** Was the outcome data collection blinded? | x | x | x | x | x |
| **Q22:** Were all data collected in the same manner for safety intervention and comparison group(s)? | x | x |  |  |  |
| **Q23:** Were all data before and after the safety intervention collected in the same manner? |  |  | x | x |  |
| **Q24:** In which country was the study conducted? | x | x | x | x | x |

## Risk of bias table

| Covering RCT, Quasi-experimental designs and controlled BA designs | | | |
| --- | --- | --- | --- |
| **Dimensions** | **Domains** | **Description** | **Reviewer author’s decision** |
| Selection Sample bias | Sequence generation | Describe the method used to generate the allocation sequence in sufficient detail to allow an assessment of whether it should produce comparable groups. | Was the random sequence generation adequate? (In the case of cluster randomized studies with small numbers, was stratified or pair-matched randomization used to generate cluster randomization?)  Low risk  High Risk  Unclear or not reported |
|  | Allocation concealment | Describe the method used to conceal the allocation sequence in sufficient detail to determine whether the intervention allocations could have been foreseen in advance of, or during, enrolment. | Was allocation adequately concealed? (For cluster RCTs - were individuals recruited prior to cluster randomization (if not, was the cluster adequately concealed prior to concealment)?  Low risk  High Risk  Unclear or not reported |
|  | Equivalent groups | Describe baselines differences between intervention and comparison groups. | Were baselines reported, checked and cases of imbalances adequately controlled for?  Low risk  High Risk  Unclear or not reported |
| Performance bias | Blinding of participants | Describe if participants were blinded. | Low risk  High Risk  Unclear or not reported |
|  |  |  |  |
| Detection bias | Blinding of outcome assessors  *(Assessments for each main outcome or class of outcomes).* | Describe if outcome assessors were blinded, or if outcome assessors had vested interests. | Was knowledge of the allocated intervention adequately prevented during the study? Outcome assessors were not blinded but the review authors judge that the outcome was not likely to be influenced by lack of blinding.  Low risk  High Risk  Unclear or not reported |
|  | Statistical analysis  *(Assessment for outcomes using time to event data)* | Censoring (also related to attrition bias – see below). Describes measures taken to account for censoring in time-to-event data.  Cluster and unit of analysis issues. | Censored data reported and adequately accounted for (i.e. censoring unlikely to introduce bias?)  (For cluster RCTs, were appropriate methods used to account for clustering?)  Low risk  High Risk  Unclear or not reported |
| Attrition bias | Incomplete outcome data  *(Assessments for each main outcome or class of outcomes).* | Describe the completeness of the sample and follow data for each main outcome, including whether attrition and exclusions were reported /and reasons given), and if any re-inclusions in analyses performed by the review authors, including the use of ITT. | Were incomplete outcome data adequately accounted for? (For cluster RCTs, were all clusters included in the outcome data and analysis?)  Low risk  High Risk  Unclear or not reported |
| Reporting bias | Selective reporting of outcome and results  *(Assessments for each main outcome or class of outcomes).* | If possible check that pre-specified primary outcomes have been reported. | Are reports of the study free of suggestion of selective outcome reporting?  Low risk  High Risk  Unclear or not reported |
| Other sources of bias | Other potential sources of bias. | Describe whether study authors have reported additional concerns regarding other potential sources of bias, such as history, placebo effect, industrial sponsorship etc., and whether they were adequately accounted for. | Was the study apparently free of other problems that could put it at high risk of bias?  Low risk  High Risk  Unclear or not reported |
|  |  |  |  |

| For For serial measures (ITS) we used the seven-standard risk of bias criteria for interrupted time series studies based on the Cochrane Effective Practice and Organization of Care (EPOC) Review group (EPOC, 2016). | |
| --- | --- |
| 1. History (maturation), Was the intervention independent of other changes? | Score “Low risk” if there are compelling arguments that the intervention occurred independently of other changes over time and the outcome was not influenced by other confounding variables/historic events during study period. *If Events/variables identified, note what they are.* Score “High risk” if reported that intervention was not independent of other changes in time. |
| 1. Shape of the intervention effect (pre-specified) | Score “Low risk” if point of analysis is the point of intervention OR a rational explanation for the shape of intervention effect was given by the author(s). Where appropriate, this should include an explanation if the point of analysis is NOT the point of intervention; Score “High risk” if it is clear that the condition above is not met. |
| 1. Intervention affect data collection, | Score “Low risk” if reported that intervention itself was unlikely to affect data collection (for example, sources and methods of data collection were the same before and after the intervention); Score “High risk” if the intervention itself was likely to affect data collection (for example, any change in source or method of data collection reported). |
| 1. Knowledge of the allocated interventions | Score “Low risk” if the authors state explicitly that the primary outcome variables were assessed blindly, or the outcomes are objective, e.g. length of hospital stay. Primary outcomes are those variables that correspond to the primary hypothesis or question as defined by the authors. Score “High risk” if the outcomes were not assessed blindly. Score “Unclear risk” if not specified in the paper. |
| 1. Incomplete outcome data (attrition), | Score “Low risk” if missing outcome measures were unlikely to bias the results (e.g. the proportion of missing data was similar in the pre- and post-intervention periods or the proportion of missing data was less than the effect size i.e. unlikely to overturn the study result). Score “High risk” if missing outcome data was likely to bias the results. Score “Unclear risk” if not specified in the paper (Do not assume 100% follow up unless stated explicitly). |
| 1. Selective outcome reporting (reporting bias) | Score “Low risk” if there is no evidence that outcomes were selectively reported (e.g. all relevant outcomes in the methods section are reported in the results section). Score “High risk” if some important outcomes are subsequently omitted from the results. Score “Unclear risk” if not specified in the paper. |
| 1. Other risk of bias | Score “Low risk” if there is no evidence of other risk of biases. e.g. should consider if seasonality is an issue (i.e. if January to June comprises the pre-intervention period and July to December the post, could the “seasons’ have caused a spurious effect). |
| Additional ROB criteria included (all study designs)   1. Intervention fidelity   (and/or exposure to other confounding factors) | Score “low risk” if measures is taken to secure intervention fidelity and/or exposure to other factors besides the intervention and comparison that may confound the results (or that a control comparison received the intervention). Score high risk if now measures are taken, otherwise unclear or not reported |
| 1. Intervention rationale   (Why should the intervention work) | Score “low risk” if reported, and a reasonable rationale is described, e.g., a sound theoretical framework or concepts used to support why the intervention should work (in this context). Score high risk if no sound rationale for the intervention is presented, otherwise unclear or not reported |
|  |  |
